# Versatile approach towards fully desymmetrized trehalose with a novel set of orthogonal protecting groups

**DOI:** 10.3389/fchem.2023.1332837

**Published:** 2024-01-11

**Authors:** Tomáš Vašíček, Benjamin Arensmeyer, Alessandro Monti, Alla Zamyatina

**Affiliations:** Department of Chemistry, Institute of Organic Chemistry, University of Natural Resources and Life Sciences, Vienna, Austria

**Keywords:** carbohydrates, glycochemistry, synthesis, protecting group manipulations, regioselectivity

## Abstract

Trehalose-containing glycans play an essential role in bacterial pathogenesis, host-pathogen interaction, and cell signaling. The investigation of trehalose uptake and metabolism in *Mycobacteria* using synthetic desymmetrized trehalose probes is an important approach for the development of diagnostic tools and potential therapeutics for tuberculosis. Trehalose-derived mycobacterial glycolipids activate the innate immune response through recognition by the C-type lectin Mincle, justifying efforts to develop novel trehalose-based Mincle-dependent adjuvants. The chemical synthesis of trehalose-based glycoconjugates, glycolipids, and small-molecule trehalose probes requires the challenging chemical desymmetrization of eight hydroxyl groups in a *C*_*2*_-symmetric disaccharide αGlc(1↔1)αGlc. Using a novel set of orthogonal protecting groups, we developed a flexible multiscale synthetic approach to a collection of differently and variably protected fully desymmetrized trehalose derivatives, ready for final chemical modification with relevant functional or reporter groups. Using a regioselective and site-specific protecting group strategy, we performed multiple symmetry-breaking operations, resulting in a library of trehalose-derived orthogonally protected building blocks as a versatile source for the synthesis of complex trehalose-containing glycans.

## 1 Introduction

The manipulation of protecting groups is a common and convenient way to achieve the synthesis of complex biomolecules, including carbohydrates. In carbohydrate chemistry, protecting groups are often applied to impart specific chemical properties to synthetic intermediates, including controlling stereoselectivity in glycosylation reactions and manipulating the reactivity of donor and acceptor molecules (Zhang et al., 1999; Jensen et al., 2004; Pedersen et al., 2007; Wu and Wong, 2011). One of the challenges in carbohydrate synthesis is the selection of multiple orthogonal protecting groups (Ágoston et al., 2016), that can ensure the regio- and chemo-selective deprotection of a single hydroxyl or amino group used as a branching point in the oligosaccharide synthesis (Boltje et al., 2010) or as a site for attachment of functional groups to assemble complex glycoconjugates. The orthogonally protected monosaccharide building blocks are commonly used as a starting point for the assembly of biomolecules, but naturally occurring disaccharides can also be subject to protective group manipulation to synthesize highly functionalized products. One of the most representative examples is a non-reducing non-mammalian disaccharide trehalose (αGlc(1↔1)αGlc).

Trehalose-containing glycans and glycolipids found in *Mycobacteria* (Nobre et al., 2014), fungi (Yang et al., 2012), and worms (Penkov et al., 2010) and more recently in *Salmonella* (Reinink et al., 2019) are important virulence factors (Thanna and Sucheck, 2016; Vanaporn and Titball, 2020) that play an essential role in host-pathogen interaction (Gilleron et al., 2004; Gilmore et al., 2012; Dai et al., 2020), cell signaling (Schoenen et al., 2014; Kodar et al., 2017), and bacterial pathogenesis (Kalscheuer et al., 2010) and are therefore important synthetic targets. The pattern-recognition receptor, monocyte-inducible C-type lectin (Mincle) expressed on macrophages and other immune cells, plays a key role in immunity to *Mycobacterium tuberculosis* which produces abundant trehalose-based glycolipids, such as trehalose dimycolate (TDM) (Ishikawa et al., 2009; Schoenen et al., 2010; Lee et al., 2012). Mincle has been identified as a specific receptor for TDM responsible for triggering the production of the pro-inflammatory mediators via the Syk/CARD9 signaling pathway (Yamasaki et al., 2008; Werninghaus et al., 2009; Ostrop et al., 2015). The structural basis for Mincle activation by trehalose glycolipids is a subject of intensive research (Feinberg et al., 2013; Furukawa et al., 2013; Feinberg et al., 2016) and the potential of Mincle to activate the innate immune response has opened up new opportunities for the development of novel trehalose-derived vaccine adjuvants (Desel et al., 2013; Decout et al., 2017; Tima et al., 2017; Desel et al., 2022). Synthetic mycobacterial sulfolipid antigens and analogs (sulfoglycolipids based on the unsymmetrically derivatised trehalose) may be important candidates for tuberculosis vaccines (Guiard et al., 2008; Seeliger et al., 2012). The study of the trehalose pathway and metabolism in *Mycobacteria*, an important approach to tuberculosis diagnosis and drug development, necessitates the synthetic preparation of various desymmetrized trehalose probes (Backus et al., 2011; Swarts et al., 2012; Dai et al., 2020; Banahene et al., 2023).

The chemical synthesis of trehalose derivatives as part of glycoconjugates and parasitic glycans or trehaloses equipped with reporter groups for biorthogonal chemistry require either a complicated chemical glycosylation to assemble the double anomeric α,α-1,1′-glycosidic linkage that is difficult to perform in a stereoselective manner or a challenging desymmetrization of some of the eight hydroxyl groups in a *C*_*2*_-symmetric non-reducing disaccharide (Lin et al., 2007; Geerdink and Minnaard, 2014). The latter approach was extensively exploited, allowing the synthesis of many important biomolecules such as mycobacterial sulfolipids (Geerdink and Minnaard, 2014; Sarpe and Kulkarni, 2014) and trehalose-containing lipids (Mishra et al., 2019; Jana and Kulkarni, 2020) as well as the preparation of trehaloses modified with biorthogonal or fluorescent moieties as probes for studying the mycobacterial trehalome (Swarts et al., 2012; Dai et al., 2020; Carlier et al., 2022). However, most of the synthetic routes were based on a partial desymmetrization by differentiation of specific hydroxyl groups or on a direct substitution of the particular positions with a specific functional group (phosphate, lipid chain, reporter group, etc.) to produce the desired biomolecule.

Given the continuous progress in the study of trehalose-dependent mycobacterial biology and the discovery of new trehalose-recognizing proteins (such as the LpqY-SugABC transporter) (Parker et al., 2020; Furze et al., 2021; Pohane et al., 2021), as well as the ongoing efforts to develop novel Mincle-dependent vaccine adjuvants, a practical, easily reproducible and scalable approach to the synthesis of multipurpose desymmetrized trehalose scaffolds is in great demand. With this in mind, we aimed to develop a straightforward and efficient multi-gram-scale synthetic approach towards fully desymmetrized orthogonally protected trehaloses, which can then be used as versatile building blocks in the assembly of complex trehalose-based glycans, or as scaffolds for the introduction of reporter groups for biorthogonal chemistry and the study of trehalose pathways in mycobacteria.

## 2 Results and discussion

**FIGURE 1:**
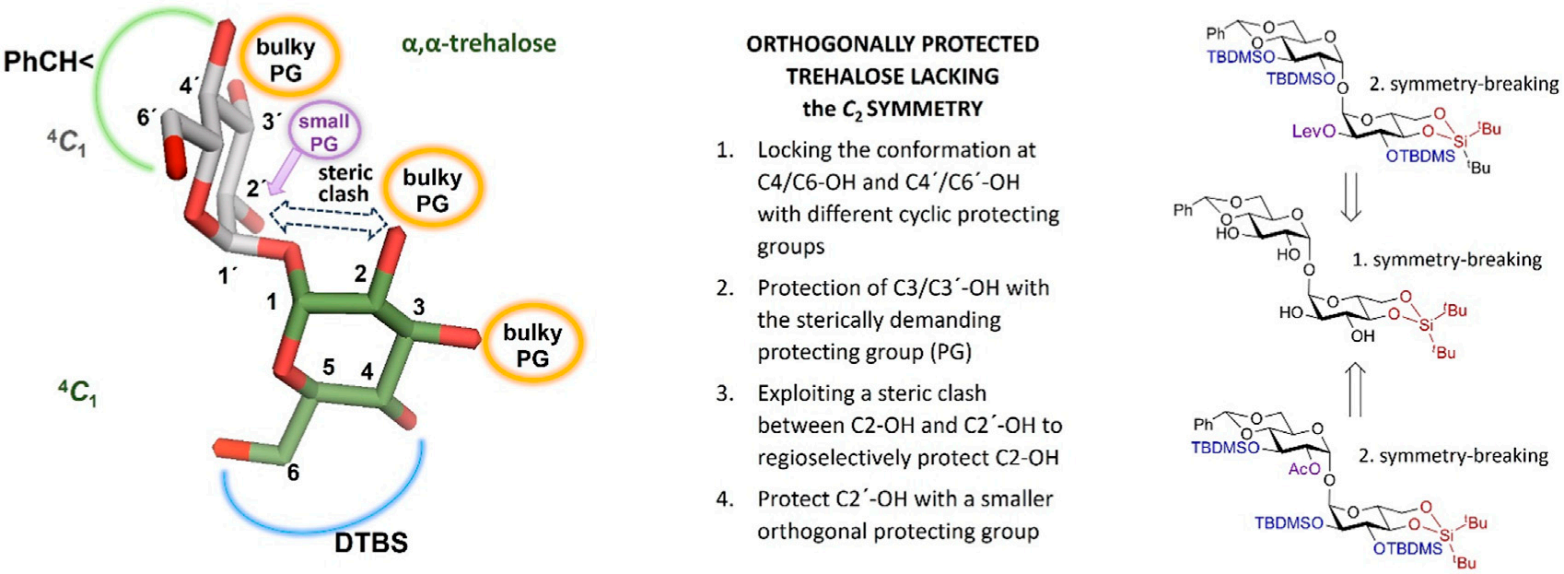
Desymmetrization design based on the conformational properties of α,α-trehalose.

To design the synthetic strategy, we considered both the chemical and structural properties of the non-reducing *C*_*2*_-symmetric disaccharide trehalose. Its tertiary structure, elucidated by X-ray- and molecular dynamics simulation studies, is governed by an exceptional rigidity of the α,α-(1↔1′) glycosidic linkage and very specific torsion angles around it (Brown et al., 1972; Dowd et al., 1992). A preferred gauche-gauche conformation with respect to the values of the torsion angles around the α,α-(1↔1′) glycosidic bond imposes a skewed relative orientation of two glucose rings and is largely dependent on the anomeric effect (Figure 1). (French et al., 2002; Nunes et al., 2010) This arrangement is valid for variably functionalized trehaloses, corresponds to a single conformational minimum, and is not influenced by the nature of the protecting or functional groups (Baddeley et al., 2003; Färnbäck et al., 2004). Therefore, the derivatization of multiple hydroxyl groups in trehalose with bulky substituents should not lead to significant deviations from a ^4^C_1_ conformation of the pyranose rings (as is characteristic of, e.g., triple TBDMS-substituted pyranoses (Pedersen et al., 2007; Bols and Pedersen, 2017)). Therefore, if the ^4^ *C*_1_ conformation of both Glc rings is additionally supported by a conformational lock provided by 4,6-*O*-cyclic protecting groups, the steric clash that would occur between bulky protecting groups on C2-OH and C2′-OH should allow the regioselective mono-substitution of either of positions 2/2′ (Figure 1).

Thus, our approach to trehalose desymmetrization was designed to rely on an initial C_2_-symmetry breaking operation by differentiating C-4/C-6 diols on both glucose units. To this end, we used two different cyclic protecting groups: benzylidene acetal to protect the C4´/C6′-OH on one glucose moiety and di-*tert*-butylsilylene (DTBS) protecting group to mask the C4/C6-OH groups on the other (Figure 1). The resulting partially desymmetrized intermediate **2** would then have two pairs of free hydroxyl groups, C2/C2′-OH and C3/C3′-OH, which would have to be differentiated by regioselective introduction of the orthogonal protecting groups. Considering the crystal structures of free- and differently substituted α,α-trehaloses (Brown et al., 1972; Baddeley et al., 2003; Färnbäck et al., 2004), we assumed that the application of bulky protecting groups would allow the differentiation of positions 2 and 2′ due to a likely steric clash between bulky substituents.

**SCHEME 1:**
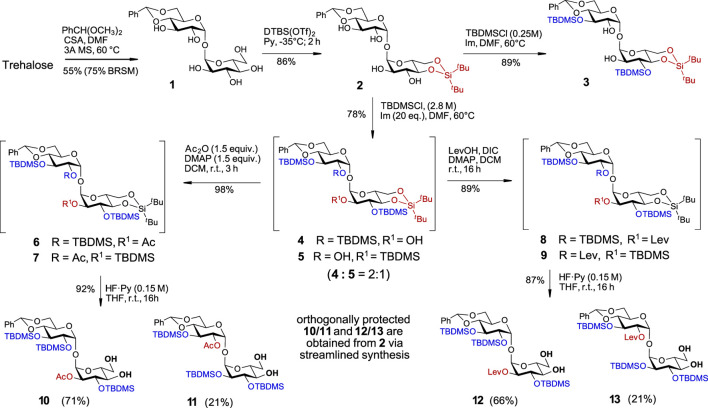
Initial symmetry-breaking transformations to differentiate C4,C6/C4′,C6′- and C2/C2′- hydroxyl groups.

Along these lines, the C-4/C-6 diol in one of the glucose moieties was initially protected by reaction with benzaldehyde dimethylacetate in the presence of camphor sulfonic acid to give monobenzylidene acetal **1** in 75% yield (BRSM), while the unreacted trehalose was fully recovered. The synthesis of trehalose 4,6-*O*-benzylidene acetal **1** starting from trehalose has been reported previously (Cheng, 1976), albeit in low yield and without indication of the purity and spectroscopic data of the isolated material. Alternatively, **1** was obtained as a by-product in the synthesis of symmetric trehalose 4,6/4′6′- dibenzylidene acetal or by partial hydrolysis or reductive opening of one of the benzylidene groups in 2,3/2′3′-protected trehalose dibenzylidene acetals (Richardson and Tarelli, 1971; Hadfield et al., 1978; Lin et al., 2007; Sarpe and Kulkarni, 2013). Here, we report the first targeted and highly efficient synthesis of trehalose 4,6-*O*-monobenzylidene acetal **1** on a 10 g scale (Supplementary Scheme 1). The second α-D-Glc unit in **1** was modified at the C4/C6 hydroxyl groups by the introduction of a di-*tert*-butylsilylene (DTBS) protecting group to generate **2** (Scheme 1). To further desymmetrize the molecule and to conform with the principles of orthogonality, we selected several temporary protecting groups with the intention to regioselectively protect either of positions C2/C3 and C2´/C3´ in compound **2**. Attempts to regioselectively introduce 2,2,2-trichloroethoxycarbonyl (Troc), 4-oxopentanoyl (Lev), triisopropylsilyl ether (TIPS), or 2-naphthylmethyl ether (Nap) protecting groups resulted in complex mixtures with a predominant formation of tetrasubstituted derivatives. It then became apparent that the use of a less reactive but more sterically hindered reagent could help to achieve the desired symmetry-breaking effect. Indeed, the reaction of **2** with *tert*-butyldimethysilyl chloride (TBDMSCl) in the presence of imidazole (Im) at 60°C led to the formation of the 3,3′-substituted disaccharide **3** in an excellent 89% yield (Scheme 1). As we aimed at the synthesis of trehalose derivatives lacking the *C*_2_-symmetry, the additional substitution of either C2-OH or C2′-OH groups was achieved by increasing the concentration of TBDMSCl in the reaction solution to 2.8 M, which afforded a mixture of tri-substituted TBDMS-protected regioisomers **4** and **5** in 78% yield in a ratio 2:1 (according to the ^1^H-NMR analysis). The remarkable regioselectivity of this transformation was confirmed by using a large excess of reagents (up to 20 equiv., i.e., 5 equiv. per OH group) and prolonged reaction times – even under these conditions, the tetrasubstituted product (2,2′,3,3′-*O*-TBDMS-protected) was not detected.

To streamline the synthesis, the mixture of partially TBDMS-protected disaccharides **4** and **5** was further derivatized without intermediate separation of individual regioisomers. The unsubstituted C2/C2′-OH groups in **4** and **5** were protected with sterically undemanding substituents - acetate or levulinate ester groups. To this end, a mixture **4 + 5** was treated with acetic anhydride/DMAP to give a mixture of regioisomers **6 + 7** or was subjected to *N,N′-*diisopropylcarbodiimide (DIC)/DMAP–promoted acylation with levulinic acid to afford C-2/C-2′ Lev-protected regioisomers **8** and **9**. In the next step, also performed with the mixtures of regioisomers **6 + 7** and **8 + 9**, we removed the conformational lock in a form of the 4,6-*O*-DTBS protecting group with the intention of 'relaxing’ the ^4^ *C*_1_ conformation on one side of the molecule to allow for further desymmetrization based on the different electronic and steric effects in a ^4^ *C*_1_-locked and “unlocked” Glc moieties. To this end, we studied the conditions for the chemoselective cleavage of 4,6-*O*-DTBS protecting group in the presence of three secondary TBDMS groups. Screening different reagents (HF·Py, Et_3_N‧3HF, TBAF) and reaction solvents (THF, DMF) revealed that using HF·Py in THF guarantees the desired chemoselectivity, while the concentration of the fluoride reagent in the reaction solution, rather than the number of equivalents of HF·Py, is decisive for the chemoselective cleavage of the 4,6-*O*-DTBS group while leaving three TBDMS groups intact. Accordingly, treatment of the regioisomeric mixture **6 + 7** with a diluted solution of HF·Py (*c* 0.15 M in the reaction solution) in THF led to the chemoselective cleavage of the DTBS group to afford 4,6-diols **10** and **11**, which were isolated in 71% and 21% yield, respectively (92% overall yield). Similarly, the mixture of levulinoyl derivatives **8 + 9** was treated with a diluted solution of HF·Py to generate the diols **12** and **13** in 66% and 21% isolated yields, respectively (87% overall yield) (Scheme 1). The excellent chemoselectivity of the DTBS group cleavage was confirmed by multigram upscaling of the transformation, where no TBDMS-deprotected or partially TBDMS-deprotected by-products were detected under the proposed reaction conditions.

**FIGURE 2:**
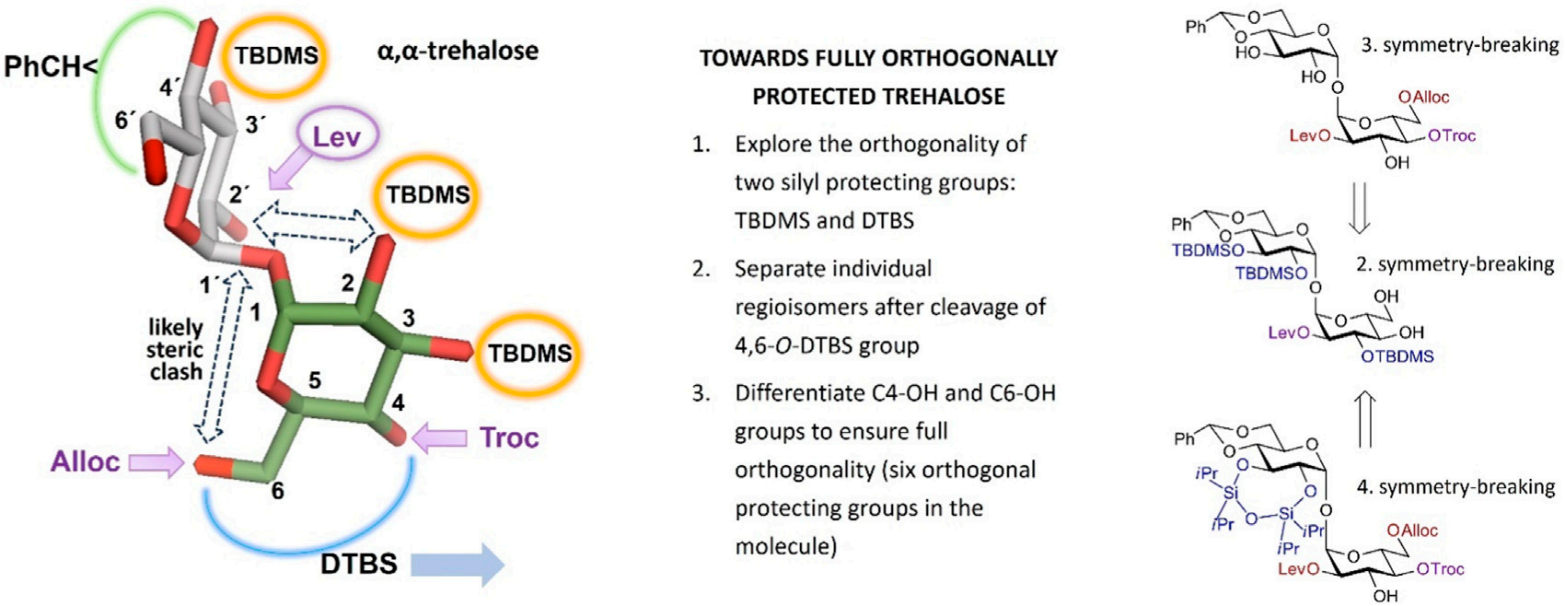
Toward orthogonally protected trehalose lacking the C_2_-symmetry, retrosynthetic scheme.

**SCHEME 2:**
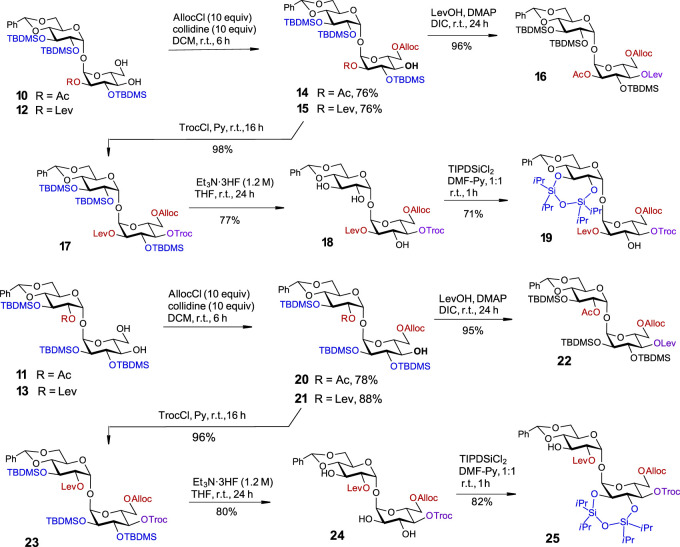
Third symmetry-breaking operation to fully differentiate two glucose moieties. Synthesis of partially desymmetrized orthogonally protected **17** and **23**. Synthesis of fully desymmetrized orthogonally protected trehalose building blocks **19** and **25**.

With four partially desymmetrized 4,6-diols **10, 11, 12,** and **13** in hand, we sought an efficient approach to regioselectively introduce an orthogonal protecting group onto the primary C6-OH. Due to a likely steric clash between the substituents at C2′ and C6 after the removal of the conformational lock provided by the 4,6-*O*-DTBS group (Figure 2), we had to rely on the use of sterically small substituents. However, the most reliable protocols for the differentiation of 4,6-diols include the use of bulky protecting groups which can be regioselectively attached to the primary C6-OH. To fulfil the criteria of orthogonality, we thought that a sterically small allyloxycarbonyl (Alloc) protecting group would be a good alternative and studied the possibilities for a regioselective protection of C6-OH using allyl chloroformate in the presence of several traditional hindered bases. Among bases tested, *sym*-collidine showed the best result, since the desired monosubstituted derivatives **14** and **15** were formed in 76% yields. Even when a large excess of AllocCl/collidine (5 eq. each) was applied, no detectable 4-*O*-Alloc-substituted by-products were formed confirming the true chemo- and regioselectivity of the transformation (Scheme 2). A similar result for the regioselective introduction of the allyloxycarbonyl group at the primary C6-OH was obtained from the reaction of 4,6-diols **11** and **13**, which gave the 6-*O*-Alloc derivatives **20** (78%) and **21** (88%), respectively. The remaining unsubstituted C4′-OH and C4-OH groups in the 2/2′-levulinated disaccharides **15** and **21** were reacted with TrocCl in pyridine to give the orthogonally protected regioisomers **17** and **23** in excellent yields of 98% and 96%, respectively. The 2/2′-acetylated compounds **14** and **20** were treated with levulinic acid under standard acylation conditions (DIC/DMAP) to furnish the fully orthogonally protected **16** and **22**. All transformations were performed in multigram scale to ensure high efficiency and reproducibility.

**SCHEME 3:**
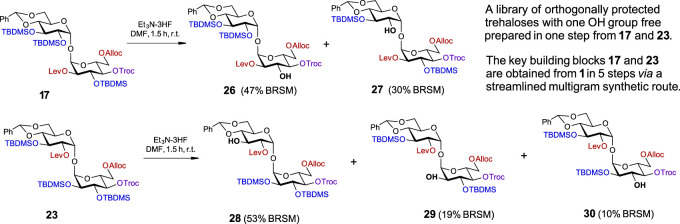
A one-step approach to a set of orthogonally protected trehaloses with one hydroxyl group free.

Given the propensity of the acetyl protecting group to migrate under a variety of chemical conditions (Lassfolk and Leino, 2023; Lassfolk and Leino, 2023), the orthogonally protected 2,2′-*O*-levulinoyl derivatives **17** and **23** were selected for the development of an advanced synthetic route. To pave the way for further desymmetrization, the TBDMS groups in **17** and **23** were removed, and the adjacent hydroxyl groups in **18** and **24** at C2´/C3´ and C2/C3, respectively, were protected as cyclic TIPDS ethers. To this end, the treatment of 2-*O*-Lev-protected **17** with a concentrated solution of Et_3_N‧3HF (*c* 1.2 M in the reaction solution) enabled the removal of all TBDMS groups and the formation of a triol **18** in 77% yield. A reaction of **18** with TIPSiCl_2_ in DMF-Py afforded the 2,3-*O*-TIPDS protected **19** in 71% yield. Similar transformations were performed with the 2′-*O*-Lev-protected regioisomer **23** which led to the formation of triol **24** in 80% yield. After the reaction of the latter with TIPSiCl_2_ in the presence of pyridine, the desymmetrized fully orthogonally protected trehalose **25** was obtained in 82% yield (Scheme 3).

**FIGURE 3:**
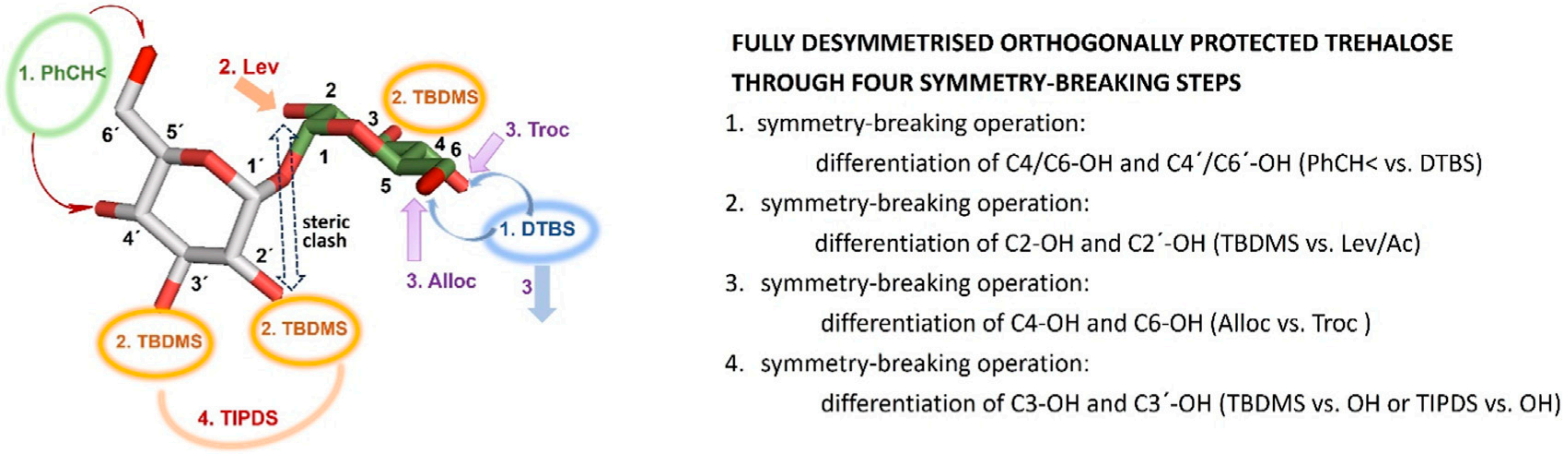
Synthetic steps to fully desymmetrized trehalose using four symmetry-breaking operations.

Using four major symmetry-breaking operations, we developed an uncomplicated, efficient, and easily reproducible synthetic route to a library of partially and fully desymmetrized orthogonally protected trehaloses (Figure 3). Both the fully orthogonally protected disaccharides **19** and **25** and the partially protected trehaloses **10/11**, **12/13**, **14/15**, **16/17**, **20/21**, and **22/23** in Schemes 1, 2 represent valuable building blocks that can be used in the assembly of complex trehalose-containing glycans and glycoconjugates, depending on the needs of a particular synthetic route.

In order to gain access to a series of orthogonally protected trehalose derivatives with a single free OH group at a specific position in one step, we explored the possibility of regioselective deprotection of the TBDMS groups in the sterically tensed **17** and **23** (Scheme 3). As we have already established for the use of HF·Py for the chemoselective deprotection of the cyclic 4,6-*O*-DTBS group (Scheme 1), the total concentration of the fluoride reagent in the reaction solution was crucial for the chemo- and regioselectivity of transformations. Since the removal of all three TBDMS groups in **17** required the use of a 1.2 M solution of [3HF·Et_3_N] in THF, the reagent concentration was reduced to 0.8 M and then to 0.25 M, which required longer reaction times (Supplementary Table S1). Changing the solvent to DMF and using 0.2 M [3HF·Et_3_N] accelerated the reaction rate and gave an easily separable mixture of partially protected compounds **26** and **27** with a free OH group in position 3 or 2′ in 47% and 30% isolated yields (BRSM), respectively (Scheme 3). The sites of attachment of silyl groups were confirmed by ^1^H-^29^Si HMBC-NMR. Similarly, the treatment of a fully protected **23** with low concentration [3HF·Et_3_N] (0.20 M) in DMF allowed for the synthesis of C3′-OH compound **28** as the major product (53% BRSM) along with C2-OH derivative **29** (19% BRSM) and C3-OH product **30** (10% BRSM), while the unreacted starting material was completely recovered (Supplementary Table S2). In this way, five different orthogonally protected trehalose building blocks were straightforwardly prepared from intermediates **17** and **23**.

In conclusion, using a novel set of orthogonal protecting groups, we developed a flexible multiscale synthetic route to a collection of differently and variably protected fully desymmetrized trehalose derivatives ready for final chemical derivatization with functional or reporter groups for biorthogonal chemistry. These trehalose building blocks serve as versatile precursors for a variety of biomolecules important for studying host-pathogen interactions, e.g., in *Mycobacteria* or other pathogens. The library of eleven desymmetrized variably orthogonally protected trehalose building blocks that can be routinely prepared via a streamlined synthetic route in multigram scale provides straightforward access to the synthesis of naturally occurring trehalose-based biomolecules and analogues thereof.

## 3 Experimental section

General synthetic methods. Reagents and solvents were purchased from commercial suppliers and used without further purification unless otherwise stated. Toluene was dried by distillation first over phosphorus pentoxide, then over calcium hydride, and was then stored over activated 4 Å molecular sieves (MS). Solvents were dried by storage over activated MS for at least 24 h prior to use (dichloromethane 4 Å, acetonitrile, and DMF 3 Å). Residual moisture was determined by coloumbometric titration on a Mitsubishi CA21 Karl Fischer apparatus and did not exceed 20 ppm. Reactions were monitored by TLC performed on silica gel 60 F254 HPTLC precoated glass plates with a 25 mm concentration zone (Merck). Spots were visualized by dipping into a sulfuric acid–*p*-anisaldehyde solution and subsequent charring at 250 °C. Solvents were removed under reduced pressure at ≤40 °C. Preparative HPLC was performed on a YMC Pack SIL-06 250 × 20 mm, S-5 μm, 6 nm column or on a YMC Pack SIL-06 250 × 10 mm, S-5 μm, 6 nm column. Preparative MPLC and column chromatography were performed using silica gel 60 (0.040–0.063 mm). NMR spectra were recorded on a Bruker Avance III 600 spectrometer (^1^H at 600.22 MHz; ^13^C at 150.93 MHz; ^31^P at 242.97 MHz) using standard Bruker NMR software. Chemical shifts are reported in ppm, where ^1^H NMR spectra recorded from samples in CDCl_3_ were referenced to internal TMS and ^13^C spectra were referenced to the corresponding solvent signal (77.16 ppm for CDCl_3_). NMR spectra recorded from samples in other solvents were referenced to residual solvent signals (for CD_3_OD 3.31 and 49.00 ppm; for CD_2_Cl_2_ 5.32 and 53.84 ppm; for DMSO-d_6_ 2.50 and 39.52 ppm; for ^1^H and ^13^C NMR, respectively). NMR spectra recorded in CDCl_3_-MeOD (4:1, v/v) were referenced to residual solvent signals of CDCl_3_ (7.26 ppm and 77.16 ppm; ^1^H and ^13^C NMR, respectively). NMR spectra recorded in CDCl_3_: MeOD (1:1 to 4:1, v/v) were referenced to residual solvent signals of MeOD (3.31 and 49.00 ppm, ^1^H and ^13^C NMR, respectively). ^31^P NMR spectra were referenced according to IUPAC recommendations from a referenced ^1^H-NMR spectrum. In all 1,1′-disaccharides, the NMR signals of the Glc ring on the left (benzylidene acetal-protected) is indicated by primes. Centrifugal partition chromatography (CPC) was performed on a SCPC-100 device with 100 mL column volume, a 3,000 rpm rotation speed, and 100 mg–1 g injection range (ARMEN, AlphaChrom). High-resolution mass spectrometry (HRMS) was carried out on acetonitrile solutions via LC-TOF MS (Agilent 1200SL HPLC and Agilent 6210 ESI-TOF, Agilent Technologies). Datasets were analyzed using Agilent Mass Hunter Software. MALDI-TOF MS was performed in negative-ion mode using a Bruker Autoflex Speed instrument with 6-aza-2-thiothymine (ATT) as matrix and ammonium sulfate as additive. Optical rotation was measured on an Anton Paar MCP 100 polarimeter featuring integrated Peltier temperature control. All [α]^D^_20_ values are reported in units of deg·dm^−1^ cm^3^ g^−1^; the corresponding concentrations are reported in g/100 mL.

**4,6-*O*-Benzylidene-α-D-glucopyranosyl-(1↔1)-α-D-glucopyranoside (1)**. Trehalose dihydrate (10.0 g, 26.44 mmol) was dissolved in dry DMF (264 mL, c = 0.100 M) and stirred with crushed molecular sieves (3Å; 1.00 g) at r. t. for 12 h. Benzaldehyde dimethyl acetal (DMT) (8.04 mL, 52.87 mmol) and camphor-10-sulfonic acid (CSA) (1.840 g, 7.93 mmol) were added and the reaction mixture was stirred at 60°C for 2 h. The reaction mixture was brought to r. t. and Et_3_N (4.2 mL, 29.08 mmol) was added dropwise. The stirring was continued for 15 min, the solids were removed by filtration over a pad of Celite, and the solution was concentrated. The residue was repeatedly co-evaporated from toluene/MeOH (110 mL, 10/1) to completely remove the reaction solvent DMF (the residue was taken up in MeOH (10 mL) and diluted with toluene (100 mL), and the solution was concentrated to dryness). The residue was partitioned between H_2_O/EtOAc (400 mL, 3/1), the phases were separated, and the organic phase was washed with H_2_O (3 × 50 mL). The combined aqueous phases were reextracted with EtOAc (2 × 50 mL), and concentrated. The residue was dissolved in MeOH/H_2_O (60 mL, 8/2) and purified by ion-exchange chromatography using AG 1-X8 resin (OH)^-^ - form (4 × 15 cm), eluent: MeOH/H_2_O (8/2) to remove the residual CSA. The fractions were collected and concentrated to dryness, and the residue was purified by *i*) column chromatography on silica gel or *ii*) centrifugal partition chromatography (CPC).

Purification by column chromatography on silica gel: the residue was taken up in (CH_2_Cl_2_-MeOH-NH_4_OH, 6/4/1) and loaded on a silica gel column (200 g, eluent: CH_2_Cl_2_-MeOH-NH_4_OH, 6/4/1 → 4/6/1) to afford **1** (5.9 g) and trehalose (2.9 g). The yield of **1** based on recovered starting material (BRSM) is 70%. R_f_ = 0.51 (CH_2_Cl_2_-MeOH-NH_4_OH, 6/4/0.8, v/v/v); R_f_ = 0.32 (*i*PrOH-H_2_O-NH_4_OH, 7/2/1, v/v/v); ${\left[\alpha \right]}_{D}^{20}$ = 120 (c = 1.4, MeOH), ^1^H NMR (600 MHz, MeOD): δ [ppm] = 7.51-7.48 (m, 2H, *Ph*CH), 7.36-7.32 (m, 3H, *Ph*CH), 5.57 (s, 1H, PhC*H*), 5.16 (d, 1H, ^3^ *J*_1′,2′_ = 3.9 Hz, H-1′), 5.10 (d, 1H, ^3^ *J*_1,2_ = 3.8 Hz, H-1), 4.22 (dd, 1H, ^2^ *J*_6′a,6′b_ = 10.1 Hz, ^3^ *J*_6′a,5′_ = 5.0 Hz, H-6′a), 4.10 (td, 1H, ^3^ *J*_5′,4′_ = ^3^ *J*_5′,6′a_ = 10.0 Hz, ^3^ *J*_5′,6′a_ = 4.9 Hz, H-5′), 4.01 (t, 1H, ^3^ *J*_3′,2′_ = ^3^ *J*_3′,4′_ = 9.4 Hz, H-3′), 3.86 (ddd, 1H, ^3^ *J*_5,4_ = 10.0 Hz, ^3^ *J*_5,6b_ = 5.4 Hz, ^3^ *J*_5,6a_ = 2.3 Hz, H-5), 3.81 (t, 1H, ^3^ *J*_3,2_ = ^3^ *J*_3,4_ = 9.2 Hz, H-3), 3.80 (dd, 1H, ^2^ *J*_6a,6b_ = 12.0 Hz, ^3^ *J*_6a,5_ = 2.5 Hz, H-6a), 3.72 (t, 1H, ^2^ *J*_6′a,6′b_ = ^3^ *J*_6′b,5′_ = 10.2 Hz, H-6′b), 3.68 (dd, 1H, ^2^ *J*_6b,6a_ = 11.9 Hz, ^3^ *J*_6b,5_ = 5.4 Hz, H-6b), 3.62 (dd, 1H, ^3^ *J*_2′,3′_ = 9.4 Hz, ^3^ *J*_2′,1′_ = 3.9 Hz, H-2′), 3.49 (dd, 1H, ^3^ *J*_2,3_ = 9.8 Hz, ^3^ *J*_2,1_ = 3.8 Hz, H-2), 3.48 (t, 1H, ^3^ *J*_4′,3′_ = ^3^ *J*_4′,5′_ = 9.5 Hz, H-4′), 3.34 (dd, 1H, ^3^ *J*_4,5_ = 9.9 Hz, ^3^ *J*_4,3_ = 9.0 Hz, H-4); ^13^C NMR (151 MHz, MeOD): δ [ppm] = 139.24 (*C*_*q*_, Ph), 129.89, 129.01, 127.54 (CH, Ph), 103.06 (Ph*C*H), 95.94 (C-1′), 95.51 (C-1), 83.10 (C-4′), 74.51 (C-3), 73.92 (C-5), 73.86 (C-2′), 73.17 (C-2), 71.87 (C-4), 71.57 (C-3′), 69.97 (C-6′), 64.09 (C-5′), 62.62 (C-6); HRMS (^+^ESI) m/z: calcd. for C_19_H_26_O_11_ [M + H]^+^ 431.1548, found 431.1555.

Purification by centrifugal partition chromatography (CPC). The biphasic liquid system (800 mL) was prepared by equilibration in the separation funnel the three-component solvent mixture EtOAc/MeOH/H_2_O (20/7/20). The separation was performed in descending mode with the upper (organic) phase serving as the stationary phase and the lower (aqueous) phase serving as the mobile phase. The column was initially conditioned by pumping the upper phase in descending mode at a rotation speed of 500 rpm and a flow rate of 30 mL min^−1^. The 5 mL sample (9 g of crude material [**1 + Tre**] was dissolved in 45 mL of the solvent mixture (EtOAc-MeOH-H_2_O, 20/7/20, v/v/v; 23 mL of upper phase and 22 mL of lower phase; injection volume 5 mL/1 g) was injected into the CPC column. The separation was performed at a rotation speed of 2,000 rpm and a mobile phase (aqueous phase) flow rate of 6 mL min^−1^. Samples [**1 + Tre**] of 1 g (5 mL) each were injected repeatedly into the equilibrated CPC column to give, after separation, 1 (5.92 g) and trehalose (3,1 g). The yield of **1** based on recovered starting material (BRSM) is 75%.

**4,6-*O*-Benzylidene-α-D-glucopyranosyl-(1↔1)-4,6-*O*-di-*tert*-butylsilylene-α-D-glucopyranoside** **(2)**. To a stirred solution of **1** (5.0 g; 11.62 mmol) in dry pyridine (116 mL) powdered activated molecular sieves (MS 3Å; 330 mg) were added and the suspension was stirred for 2 h at r. t. in the atmosphere of Ar. The mixture was cooled to −35°C and di-*tert*-butylsilyl bis(trifluoromethanesulfonate) (568 mL; 17.42 mmol) was added dropwise. The reaction mixture was stirred for 3.5 h at −35°C, then triethylamine (4,2 mL) and MeOH (8,4 mL) were successively added, and the mixture was stirred for 10 min. The reaction mixture was brought to r. t., the solids were removed by filtration over a pad of Celite, and the filtrate was concentrated. The residue was dissolved in EtOAc (350 mL), washed with sat. aq. NaHCO_3_ (3 × 100 mL), and brine (100 mL), dried over MgSO_4_, filtered, and concentrated. The residue was purified by column chromatography on silica gel (toluene-EtOAc, 1/1 → 2/8) to afford **2** (5.7g, 86%) as a solid. R_f_ = 0.92 (CHCl_3_-MeOH-NH_4_OH, 8/2/0.25, v/v/v); R_f_ = 0.44 (EtOAc–toluene, 6/4, v/v); ${\left[\alpha \right]}_{D}^{20}$ = 82 (c = 0.7, MeOH); ^1^H NMR (600 MHz, MeOD): δ [ppm] = 7.51-7.47 (m, 2 H, *Ph*CH), 7.36-7.32 (m, 3 H, *Ph*CH), 5.57 (s, 1 H, PhC*H*), 5.09 (d, 1 H, ^3^ *J*_1′,2′_ = 4.0 Hz, H-1′), 5.05 (d, 1 H, ^3^ *J*_1,2_ = 3.8 Hz, H-1), 4.21 (dd, 1 H, ^2^ *J*_6′a,6′b_ = 10.1 Hz, ^3^ *J*_6′a,5′_ = 5.0 Hz, H-6′a), 4.16 (td, 1 H, ^3^ *J*_5,4_ = ^3^ *J*_5,6b_ = 10.0 Hz, ^3^ *J*_5,6a_ = 5.0 Hz, H-5), 4.09 (dd, 1 H, ^2^ *J*_6a,6b_ = 9.8 Hz, ^3^ *J*_6a,5_ = 5.0 Hz, H-6a), 4.06 (td, 1 H, ^3^ *J*_5′,4′_ = ^3^ *J*_5′,6′b_ = 9.9 Hz, ^3^ *J*_5′,6′a_ = 4.9 Hz, H-5′), 4.03 (t, 1 H, ^3^ *J*_3′,2′_ = ^3^ *J*_3′,4′_ = 9.4 Hz, H-3′), 3.85 (dd, 1 H, ^3^ *J*_3,2_ = 9.5 Hz, ^3^ *J*_3,4_ = 9.0 Hz, H-3), 3.81 (t, 1 H, ^2^ *J*_6b,6a_ = ^3^ *J*_6b,5_ = 10.1 Hz, H-6b), 3.72 (t, 1 H, ^2^ *J*_6′b,6′a_ = ^3^ *J*_6′b,5′_ = 10.3 Hz, H-6′b), 3.66 (dd, 1 H, ^3^ *J*_4,5_ = 9.6 Hz, ^3^ *J*_4,3_ = 8.9 Hz, H-4), 3.61 (dd, 1 H, ^3^ *J*_2′,3′_ = 9.4 Hz, ^3^ *J*_2′,1′_ = 4.0 Hz, H-2′), 3.53 (dd, 1 H, ^3^ *J*_2,3_ = 9.7 Hz, ^3^ *J*_2,1_ = 3.8 Hz, H-2), 3.47 (t, 1 H, ^3^ *J*_4′,3′_ = ^3^ *J*_4′,5′_ = 9.5 Hz, H-4′), 1.07, 1.02 (2xs, 18H, 2x [C*H*_3_]_3_C, DTBS); ^13^C NMR (151 MHz, MeOD): δ [ppm] = 139.24 (*C*_*q*_, Ph), 129.88, 129.01, 127.54 (CH, Ph), 103.05 (Ph*C*H), 95.87 (C-1′), 95.50 (C-1), 83.02 (C-4′), 79.28 (C-4), 74.29 (C-3), 73.63 (C-2′), 73.15 (C-2), 71.54 (C-3′), 69.96 (C-6′), 67.96 (C-6), 67.72 (C-5), 64.20 (C-5′), 27.95, 27.71 ([*C*H_3_]_3_C, DTBS), 23.60, 20.93 ([CH_3_]_3_ *C*, DTBS); HRMS (^+^ESI) m/z: calcd. for C_27_H_43_O_11_Si [M + H]^+^ 571.2569, found 571.2577.

**4,6-*O*-Benzylidene-3-*O*-*tert*-butyldimethylsilyl-α-D-glucopyranosyl-(1↔1)-4,6-*O*-di-*tert*-butylsilylene-3-*O*-*tert*-butyldimethylsilyl-α-D-glucopyranoside (3).** To a stirred solution of **2** (3.11 g, 5.45 mmol) in dry DMF (100 mL) powdered 3Å molecular sieves (300 mg), imidazole (2.24 g, 32.90 mmol) and *tert*-butyldimethylsilyl chloride (4.18 g, 27.7 mmol) were added under the atmosphere of Ar. The reaction mixture was stirred at 60°C for 90 min, then diluted with EtOAc (250 mL) and washed with aq. satd. NaHCO_3_ (2 × 50 mL) and brine (2 × 50 mL). The organic layer was dried over Na_2_SO_4_, filtered, and concentrated. The residue was purified by column chromatography on silica gel (toluene - EtOAc, 95/5 → 90/10) to afford **3** (3.66 g, 84%) as a solid. R_f_ = 0.42 (toluene–EtOAc, 4/1, v/v); R_f_ = 0.70 (toluene–EtOAc, 3/2, v/v); ${\left[\alpha \right]}_{D}^{20}$ = 57 (c = 1.3, CHCl_3_); ^1^H NMR (600 MHz, CDCl_3_): δ [ppm] = 7.52-7.48 (m, 2H, *Ph*CH), 7.39-7.33 (m, 3H, *Ph*CH), 5.51 (s, 1 H, PhC*H*), 5.19 (d, 1 H, ^3^ *J*_1′,2′_ = 3.7 Hz, H-1′), 5.12 (d, 1 H, ^3^ *J*_1,2_ = 3.7 Hz, H-1), 4.26 (dd, 1 H, ^2^ *J*_6′a,6′b_ = 10.3 Hz, ^3^ *J*_6′a,5′_ = 5.0 Hz, H-6′a), 4.13 (m, 1 H, ^3^ *J*_5,4_ = ^3^ *J*_5,6b_ = 10.0 Hz, ^3^ *J*_5,6a_ = 4.9 Hz, H-5), 4.08 (dd, 1 H, ^2^ *J*_6a,6b_ = 9.8 Hz, ^3^ *J*_6a,5_ = 4.9 Hz, H-6a), 4.03 (td, 1 H, ^3^ *J*_5′,4′_ = ^3^ *J*_5′,6′b_ = 9.9 Hz, ^3^ *J*_5′,6′a_ = 4.9 Hz, H-5′), 3.98 (t, 1 H, ^3^ *J*_3′,2′_ = ^3^ *J*_3′,4′_ = 9.1 Hz, H-3′), 3.83 (t, 1 H, ^2^ *J*_6b,6a_ = ^3^ *J*_6b,5_ = 10.1 Hz, H-6b), 3.78 (t, 1 H, ^3^ *J*_3,2_ = 8.9 Hz, ^3^ *J*_3,4_ = 8.9 Hz, H-3), 3.70 (t, 1 H, ^2^ *J*_6′b,6′a_ = ^3^ *J*_6′b,5′_ = 10.3 Hz, H-6′b), 3.69 (t, 1 H, ^3^ *J*_4,5_ = 9.6 Hz, ^3^ *J*_4,3_ = 9.6 Hz, H-4), 3.67 (dd, 1 H, ^3^ *J*_2′,3′_ = 9.6 Hz, ^3^ *J*_2′,1′_ = 3.9 Hz, H-2′), 3.57 (m, 1H, ^3^ *J*_1,2_ = 3.7 Hz, ^3^ *J*_2,3_ = 9.0 Hz, H-2), 3.46 (t, 1 H, ^3^ *J*_4′,3′_ = ^3^ *J*_4′,5′_ = 9.4 Hz, H-4′), 2.02 (d, 1H, C2′-O*H*, 1.89 (d, 1H, C2-O*H*), 1.05, 0.99, 0.95, 0.89 (4xs, 36H, 4x [C*H*_3_]_3_C], DTBS), 0.18, 0.17, 0.10, 0.06 (4xs, 12H, 4xC*H*_3_, 2xTBDMS); ^13^C NMR (151 MHz, CDCl_3_): δ [ppm] = 137.25 (*C*_*q*_ *,* Ph), 128.90, 128.09, 126.20 (*C*H, Ph), 101.68 (Ph*C*H), 94.71 (C-1), 94.62 (C-1′), 81.57 (C-4′), 77.53 (C-4), 75.44 (C-3), 73.18 (C-2′), 72.68 (C-2), 72.43 (C-3′), 68.93 (C-6′), 67.16 (C-5), 66.69 (C-6), 63.80 (C-5′), 27.46, 26.99, 25.98, 25.85 (4x [*C*H_3_]_3_C], 2xTBDMS, DTBS), 22.68, 19.94, 18.34, 18.33 (4x [CH_3_]_3_ *C,* 2xTBDMS, 2xDTBS), −4.03, −4.26, −4.60, −4.91 (4x*C*H_3_, 2xTBDMS); HRMS (^+^ESI) m/z: calcd. for C_27_H_43_O_11_Si [M + H]^+^ 571.2569, found 571.2577.

**4,6-*O*-Benzylidene-2,3-di-*O*-*tert-*butyldimethylsilyl-α-D-glucopyranosyl-(1↔1)-3-*O*-*tert*-butyldimethylsilyl-4,6-*O*-di-*tert*-butylsilylene-α-D-glucopyranoside (4)** and **4,6-*O*-Benzylidene-3-*O*-*tert*-butyldimethylsilyl-α-D-glucopyranosyl-(1↔1)-2,3-di-*O*-*tert*-butyldimethylsilyl-4,6-*O*-di-*tert*-butylsilylene-α-D-glucopyranoside (5).** To a stirred solution of **2** (7.98 g, 13.98 mmol) in dry DMF (100 mL) imidazole (8.1 g, 279.6 mmol), *tert*-butyldimethylsilyl chloride (42.14 g, 279.6 mmol) and powdered molecular sieves (3Å, 800 mg, 10%) were added under atmosphere of Ar. The reaction mixture was stirred for 22 h at 60°C under atmosphere of Ar, diluted with EtOAc (500 mL), and washed with aq. satd. NaHCO_3_ (3 × 100 mL) and brine (2 × 100 mL). The organic layer was dried over Na_2_SO_4,_ filtered, and concentrated. The residue was purified by column chromatography on silica gel (toluene -EtOAc, 98/2 → 80/20) to afford a mixture of **4** and **5** (2:1 according to the ^1^H-NMR analysis), 9.91 g, 78%, as a solid. R_f_ = 0.43 and 0.38, respectively, (toluene–EtOAc, 95/5, v/v); R_f_ = 0.67 and 0.62 (hexane–EtOAc, 4/1, v/v); ^1^H NMR (chemical shifts for compound **4** are indicated by superscript ^m^) (600 MHz, CDCl_3_): δ [ppm] = 7.47-7.33 (m, *Ph*CH), 5.47, 5.42 (2xs, PhC*H*), 5.18 (d, ^3^ *J*_1,2_ = 3.6 Hz, H-1), 5.10 (d, 1H, ^3^ *J*_1,2_ = 3.5 Hz, H-1)^m^, 5.03 (d, 1H, ^3^ *J*_1′,2′_ = 3.0 Hz, H-1′)^m^, 5.03 (d, ^3^ *J*_1′,2′_ = 3.1 Hz, H-1′), 4.41-3.36 (4xH-3, 4xH-4, 4xH-5, 4xH-6), 3.67 (dd, ^3^ *J*_1′,2′_ = 3.5 Hz, ^3^ *J*_2′,3′_ = 8.7 Hz, H-2′)^m^, 3.65 (dd, ^3^ *J*_1′,2′_ = 3.7 Hz, ^3^ *J*_2′,3′_ = 8.7 Hz, H-2′), 3.59 (dd, ^3^ *J*_1′,2′_ = 3.1 Hz, ^3^ *J*_2′,3′_ = 8.9 Hz, H-2′), 3.58 (dd, ^3^ *J*_1,2_ = 3.7 Hz, ^3^ *J*_2,3_ = 9.4 Hz, H-2)^m^, 2.11 (d, 1H, C2-O*H*)^m^, 2.03 (d, 1H, C2′-O*H*), 1.06, 1.05, 1.02, 0.99, 0.97, 0.96, 0.94, 0.87, 0.80 (9xs, [C*H*_3_]_3_C*,* 3xTBDMS, 1xDTBS), 0.21, 0.17, 0.15, 0.13, 0.08, 0.00 (6xs, 12×C*H*_3_, 6xTBDMS); ^13^C NMR (151 MHz, CDCl_3_): δ [ppm] = 137.54, 137.42 (*C*_*q*_, Ph), 128.95, 128.07, 128.00, 126.57, 126.42 (PhCH), 102.45, 102.14 (Ph*C*H), 98.65, 96.62, 95.46, 94.16 (4xC-1), 82.50, 81.79, 78.58, 77.65, 74.70, 74.48, 73.78, 73.77, 73.28, 72.83, 72.24, 71.55, 68.08, 67.12, 63.86, 63.68 (4xC-2, 4xC-3, 4xC-4, 4xC-5), 69.10, 69.07, 66.96, 66.92 (4xC-6), 27.63, 27.61, 27.21, 27.12, 26.64, 26.58, 26.34, 26.13, 26.00, 25.87 (10x [*C*H_3_]_3_C, 6xTBDMS, 2xDTBS), 22.81, 22.78, 20.03, 19.86, 18.45, 18.29 ([CH_3_]_3_ *C*, 6xTBDMS, 2xDTBS), −2.87, −3.25, −3.77, −3.90, −3.93, −4.04, −4.27, −4.42, −4.42, −4.54, −4.66, −4.95 (12x*C*H_3_, 6xTBDMS); HRMS (^+^ESI) m/z: calcd. for C_45_H_85_O_11_Si_4_ [M + H]^+^ 930.5429, found 930.5434. HRMS (^+^ESI) m/z: calcd. for C_45_H_85_O_11_Si_4_ [M + H]^+^: 930.5429, found 930.5434.

**4,6-*O*-Benzylidene-2,3-di-*O*-*tert*-butyldimethylsilyl-α-D-glucopyranosyl-(1↔1)-3-*O*-*tert*-butyldimethylsilyl-4,6-*O*-di-*tert*-butylsilylene-2-*O*-(4-oxopentanoyl)-α-D-glucopyranoside (8)** and **4,6-*O*-Benzylidene-3-*O*-*tert*-butyldimethylsilyl-2-*O*-(4-oxopentanoyl)-α-D-glucopyranosyl-(1↔1)-2,3-di-*O*-*tert*-butyldimethylsilyl-4,6-*O*-di-*tert*-butylsilylene-α-D-glucopyranoside (9).** To a stirred solution of [**4 + 5**] (9.91 g, 9.80 mmol) in dry CH_2_Cl_2_ (23 mL, c = 0.472 M), levulinic acid (3.33 mL, 29.40 mmol), *N,N*′-diisopropylcarbodiimide (6.72 mL, 39.19 mmol), and 4-(dimethylamino)-pyridine (5.30 g, 39.19 mmol) were added successively. The reaction mixture was stirred for 16 h, then diluted with EtOAc (500 mL) and washed with aq. satd. NaHCO_3_ (4 × 100 mL), aq. satd. CuSO_4_ (3 × 50 mL), and brine (3 × 100 mL). The organic layer was dried over Na_2_SO_4,_ filtered, and concentrated. The residue was purified by column chromatography on silica gel (toluene -EtOAc, 95/5 → 80/20) to afford [**8 + 9**] (9.73 g, 89%) as a mixture. R_f_ = 0.55 and 0.47 (hexane-EtOAc, 4/1, v/v, both regio-isomers); ^1^H NMR (the chemical shifts for compound **8** are indicated by superscript ^m^) (600 MHz, CDCl_3_): δ [ppm] = 7.48-7.32 (m, *Ph*CH), 5.49, 5.43^m^ (2xs, PhC*H*), 5.19 (d, 1H, ^3^ *J*_1,2_ = 3.6 Hz, H-1)^m^, 5.15 (d, ^3^ *J*_1′,2′_ = 3.4 Hz, H-1′), 5.05 (d, 1H, ^3^ *J*_1′,2′_ = 2.8 Hz, H-1′)^m^, 4.96 (d, ^3^ *J*_1,2_ = 3.0Hz, H-1), 4.91 (dd, ^3^ *J*_2,3_ = 9.6 Hz, ^3^ *J*_1,2_ = 3.4 Hz, H-2), 4.79 (dd, 1H, ^3^ *J*_2,3_ = 9.8 Hz, ^3^ *J*_1,2_ = 3.6 Hz, H-2)^m^, 4.30-3.36 (4xH-3, 4xH-4, 4xH-5, 4xH-6), 3.66 (dd, ^3^ *J*_2′,3′_ = 8.9 Hz, ^3^ *J*_1′,2′_ = 2.7 Hz, H-2′)^m^, 3.58 (dd, ^3^ *J*_2,3_ = 9.1 Hz, ^3^ *J*_1,2_ = 3.1 Hz, H-2), 2.77- 2.62 (m, 2xC*H*_2_, Lev), 2.19, 2.02 (s, C*H*_3_, Lev), 1.07, 1.07, 1.02, 1.01, 0.97, 0.95, 0.94, 0.93 0.81, 0.81 (10×s, [C*H*_3_]_3_C*,* 6xTBDMS, 2xDTBS), 0.19, 0.18, 0.18, 0.17, 0.16, 0.16, 0.13, 0.04, 0.03, 0.03 (10×s, 12×C*H*_3_, 6xTBDMS); ^13^C NMR (151 MHz, CDCl_3_): δ [ppm] = 205.95^m^, 205.66 (*C*H_3_C = O, Lev), 172.38^m^, 172.33 (C=O, Lev), 137.35, 137.32^m^ (Ph-*C*_*q*_), 129.04^m^, 128.97, 128.08^m^, 128.04, 2 × 126.46 (CH, Ph), 102.60^m^, 102.33 (Ph*C*H), 97.69, 97.69, 94.94, 93.42^m^ (4xC-1), 82.69, 82.56, 78.51, 78.06, 74.32, 73.92, 73.80, 73.70, 73.48 71.42, 71.19, 69.01, 68.23, 67.57, 64.18, 63.47 (4xC-2, 4xC-3, 4xC-4, 4xC-5), 69.12, 69.07^m^, 66.88, 66.75^m^ (4xC-6), 37.69, 37.53^m^ (CH_2_, Lev), 29.82, 29.66^m^ (CH_3_, Lev), 27.84^m^, 27.80 (CH_2_, Lev), 27.90, 27.67, 27.24, 27.21, 26.72, 26.65, 26.41, 26.14, 25.80, 25.66 (10x [*C*H_3_]_3_C], 6xTBDMS, 2xDTBS), 22.90, 22.84, 19.80, 19.80, 18.86, 18.82, 18.47, 18.39, 18.14, 18.11 (10x [CH_3_]_3_ *C,* 6xTBDMS, 2xDTBS), −2.73, −3.16, −3.33, −3.87, −3.92, −3.98, 4.05, −4.42, −4.48, −4.55, −4.74, −4.95 (12x*C*H_3_, 6xTBDMS); HRMS (^+^ESI) m/z: calcd. for C_50_H_91_O_13_Si_4_ [M + H]^+^: 1011.5531, found 1011.5536; calcd. for C_50_H_94_NO_13_Si_4_ [M + NH_4_]^+^: 1028.5797, found 1028.5814; calcd. for C_50_H_90_KO_13_Si_4_ [M + K]^+^ 1049.5090, found 1049.5089.

**4,6-*O*-Benzylidene-2,3-di-*O*-*tert*-butyldimethylsilyl-α-D-glucopyranosyl-(1↔1)-3-*O*-*tert*-butyldimethylsilyl-2-*O*-(4-oxopentanoyl)-α-D-glucopyranoside (12)** and **4,6-*O*-Benzylidene-3-*O*-*tert*-butyldimethylsilyl-2-*O*-(4-oxopentanoyl)-α-D-glucopyranosyl-(1↔1)-2,3-di-*O*-*tert*-butyldimethylsilyl-α-D-glucopyranoside (13).** To a stirred solution of [**8 + 9**] (2.94 g, 2.91 mmol) in dry THF (12 mL) in a PTFE reaction vessel, a solution of HF·pyridine (240 μL, 0.152 M) was added at 0°C. The reaction mixture was stirred for 16 h at r. t. and concentrated. The residue was dissolved in EtOAc (250 mL), washed with aq. satd. NaHCO_3_ (3 × 50 mL), aq. satd. CaCl_2_ (3 × 30 mL), and brine (2 × 50 mL). The organic layer was dried over Na_2_SO_4,_ filtered, and concentrated. The residue was purified by column chromatography on silica gel (toluene -EtOAc, 98/2 → 75/25) to afford **12** (1.670 g, 66%) as a solid and **13** (0.520 g, 21%) as a solid.

**12**: R_f_ = 0.56 (toluene–EtOAc, 3/2, v/v); ${\left[\alpha \right]}_{D}^{20}$ = 79 (c = 1.6, CHCl_3_); ^1^H NMR (600 MHz, CDCl_3_): δ [ppm] = 7.40-7.31 (m, 5H, *Ph*CH), 5.42 (s, 1H, PhC*H*), 5.34 (d, 1H, ^3^ *J*_1,2_ = 3.4 Hz, H-1), 5.02 (d, 1H, ^3^ *J*_1′,2′_ = 3.1 Hz, H-1′), 4.69 (dd, 1H, ^3^ *J*_2,3_ = 9.9 Hz, ^3^ *J*_1,2_ = 3.5 Hz, H-2), 3.65 (dd, 1H, ^2^ *J*_6a,6b_ = 11.9 Hz, ^3^ *J*_5,6a_ = 5.4 Hz, H-6a), 4.13 (t, 1H, ^3^ *J*_2′,3′_ = ^3^ *J*_3′,4′_ = 9.1 Hz, H-3′), 4.12 (t, 1H, ^3^ *J*_2,3_ = ^3^ *J*_3,4_ = 9.0 Hz, H-3), 3.94 (dt, 1H, ^3^ *J*_4,5_ = ^3^ *J*_5_,_6b_ = 9.9 Hz, ^3^ *J*_5,6a_ = 3.6 Hz, H-5), 3.82 (d, 2H, ^3^ *J*_5,6_ = 3.4 Hz, H-6a, H-6b), 3.68-3.59 (m, 3H, H-4, H-5′, H-6′b), 3.62 (dd, 1H, ^3^ *J*_2′,3′_ = 9.5 Hz, ^3^ *J*_1′,2′_ = 3.2 Hz, H-2′), 3.41 (t, 1H, ^3^ *J*_2′,3′_ = ^3^ *J*_3′,4′_ = 9.0 Hz, H-4′), 2.82-2.62 (m, 4H, 2xC*H*_2_, Lev), 2.24 (d, 1H, C6*-*O*H*), 1.94 (s, 3H, C*H*_3_, Lev), 1.92 (s, 1H, C4*-*O*H*), 0.93, 0.92, 0.82 (3×s, 27H, 3×[C*H*_3_]_3_C*,* 3xTBDMS), 0.18, 0.17, 0.13, 0.10, 0.03, 0.03 (6×s, 18H, 6×C*H*_3_, 3xTBDMS); ^13^C NMR (151 MHz, CDCl_3_): δ [ppm] = 206.03 (*C*H_3_C = O, Lev), 172.53 (C=O, Lev), 137.17 (*C*_*q*_, Ph), 129.09, 128.12, 126.36 (CH, Ph), 102.49 (Ph*C*H), 94.86 (C-1′), 90.59 (C-1), 82.43 (C-4′), 73.89 (C-2′), 73.65 (C-2), 72.02 (C-3), 71.47 (C-3′), 71.46 (C-4), 71.43 (C-5), 68.81 (C-6′), 63.74 (C-5′), 62.13 (C-6), 37.57 (CH_2_, Lev), 29.58 (CH_3_, Lev), 27.89 (CH_2_, Lev), 26.43, 26.18, 25.78 ([*C*H_3_]_3_C, 3xTBDMS), 18.46, 18.15, 18.15 ([CH_3_]_3_ *C,* 3xTBDMS), −3.03, −3.95, −4.19, −4.38, −4.38, −4.41 (6xCH_3_, 3xTBDMS); HRMS (^+^ESI) m/z: calcd. for C_42_H_78_NO_13_Si_3_ [M + NH_4_]^+^ 888.4775, found 888.4788.

**13**: R_f_ = 0.33 (toluene–EtOAc, 3/2, v/v); ${\left[\alpha \right]}_{D}^{20}$ = 68 (c = 1.6, CHCl_3_); ^1^H NMR (600 MHz, CDCl_3_): δ [ppm] = 7.46-7.42 (m, 2H, *Ph*CH), 7.37-7.34 (m, 3H, *Ph*CH), 5.48 (s, 1H, PhC*H*), 5.32 (d, 1H, ^3^ *J*_1′,2′_ = 3.7 Hz, H-1′), 5.03 (d, 1H, ^3^ *J*_1,2_ = 3.0 Hz, H-1), 4.81 (dd, 1H, ^3^ *J*_2′,3′_ = 9.4 Hz, ^3^ *J*_1′,2′_ = 3.7 Hz, H-2′), 4.25 (t, 1H, ^3^ *J*_2′,3′_ = ^3^ *J*_3′,4′_ = 9.3 Hz, H-3′), 4.22 (t, 1H, ^2^ *J*_6′a,6′b_ = 10.4 Hz, ^3^ *J*_5′,6′b_ = 4.9 Hz, H-6′a), 4.07 (t, 1H, ^3^ *J*_2,3_ = ^3^ *J*_3,4_ = 8.7 Hz, H-3), 4.01 (td, 1H, ^3^ *J*_4′,5′_ = ^3^ *J*_5′,6′b_ = 10.0 Hz, ^3^ *J*_5′,6′a_ = 4.9 Hz, H-5′), 3.78 (m, 2H, H-6a, H-6b), 3.72 (t, 1H, ^2^ *J*_6′a,6′b_ = ^3^ *J*_5′,6′a_ = 10.4 Hz, H-6′b), 3.61-3.57 (m, 1H, H-5), 3.59 (dd, 1H, ^3^ *J*_2,3_ = 9.0 Hz, ^3^ *J*_1,2_ = 3.3 Hz, H-2), 3.51 (t, 1H, ^3^ *J*_3′,4′_ = ^3^ *J*_4′,5′_ = 9.4 Hz, H-4′), 3.41 (m, 1H, ^3^ *J*_4,5_ = ^3^ *J*_5_,_6b_ = 9.0 Hz, ^3^ *J*_5,6a_ = 5.7 Hz, H-5), 3.08 (d, 1H, O*H*), 3.00-2.43 (m, 4H, 2xC*H*_2_, Lev), 2.21 (s, 3H, C*H*_3_, Lev), 0.98, 0.95, 0.81 (3×s, 27H, 3×[C*H*_3_]_3_C*,* 3xTBDMS), 0.16, 0.16, 0.15, 0.11, 0.05, −0.03 (6×s, 18H, 6×C*H*_3_, 3xTBDMS); ^13^C NMR (151 MHz, CDCl_3_): δ [ppm] = 208.65 (*C*H_3_C = O, Lev), 172.34 (C=O, Lev), 137.22 (*C*_*q*_, Ph), 128.98 (CH, Alloc), 128.03, 126.44, 102.26 (Ph*C*H), 93.39 (C-1), 90.73 (C-1′), 81.89 (C-4′), 74.31 (C-2′), 74.28 (C-3), 73.33 (C-4), 73.02 (C-2), 72.35 (C-5), 69.27 (C-3′), 68.80 (C-6′), 63.46 (C-6), 63.01 (C-5′), 37.57 (CH_2_, Lev), 29.99 (CH_3_, Lev), 27.71 (CH_2_, Lev), 26.56, 26.34, 25.67 ([*C*H_3_]_3_C, 3xTBDMS), 18.51, 18.20, 18.15 ([CH_3_]_3_ *C,* 3xTBDMS), −3.07, −3.76, −4.01, −4.41, −4.43, −4.92 (6xCH_3_ *,* 3xTBDMS); HRMS (^+^ESI) m/z: calcd. for C_42_H_78_NO_13_Si_3_ [M + NH_4_]^+^ 888.4775, found 888.4795.

**4,6-*O*-Benzylidene-2,3-di-*O*-*tert*-butyldimethylsilyl-α-D-glucopyrano-syl-(1↔1)-6-*O*-allyloxycarbonyl-3-*O*-*tert*-butyldimethylsilyl-2-*O*-(4-oxopentanoyl)-α-D-glucopyranoside (15).** To a stirred solution of **12 (**4.930 g, 5.66 mmol c = 0.250M) in dry CH_2_Cl_2_ (22 mL), *sym*-collidine (7.50 mL, 56.6 mmol) and allyloxycarbonyl chloride (6.04 mL, 56.6 mmol) were added successively at 0 °C. The reaction mixture was stirred for 6 h at r. t., diluted with toluene (20 mL), and concentrated. The residue was redissolved in EtOAc (500 mL) and washed with aq. satd. NaHCO_3_ (3 × 150 mL), aq. satd. CuSO_4_ (3 × 50 mL), and brine (2 × 100 mL). The organic layer was dried over Na_2_SO_4,_ filtered, and concentrated. The residue was purified by column chromatography on silica gel (toluene -EtOAc, 90/10 → 80/20) to afford **15** (4.13 g, 76%) as a syrup. R_f_ = 0.22 (toluene–EtOAc, 9/1, v/v); ${\left[\alpha \right]}_{D}^{20}$ = 66 (c = 0.2, CHCl_3_); ^1^H NMR (600 MHz, CDCl_3_): δ [ppm] = 7.42-7.32 (m, 5H, *Ph*CH), 5.93 (m, 1H, C*H*, Alloc), 5.41 (s, 1H, PhC*H*), 5.39-5.26 (m, 2H, C*H*_2_, Alloc), 5.33 (d, 1H, ^3^ *J*_1,2_ = 3.5 Hz, H-1), 5.03 (d, 1H, ^3^ *J*_1′,2′_ = 3.1 Hz, H-1′), 4.73 (dd, 1H, ^3^ *J*_2,3_ = 9.9 Hz, ^3^ *J*_1,2_ = 3.5 Hz, H-2), 4.64 (m, 2H, C*H*_2_, Alloc), 4.45 (dd, 1H, ^2^ *J*_6a,6b_ = 11.8 Hz, ^3^ *J*_5,6a_ = 4.0 Hz, H-6a), 4.35 (dd, 1H, ^2^ *J*_6a,6b_ = 11.8 Hz, ^3^ *J*_5,6b_ = 2.2 Hz, H-6b), 4.17 (t, 1H, ^2^ *J*_6′a,6′b_ = ^3^ *J*_5′,6′a_ = 10.4 Hz, H-6′a), 4.12 (d, 1H, ^3^ *J*_2,3_ = ^3^ *J*_3,4_ = 9.1 Hz, H-3), 4.11 (t, 1H, ^3^ *J*_2′,3′_ = ^3^ *J*_3′,4′_ = 9.0 Hz, H-3′), 4.06 (dt, 1H, ^3^ *J*_4,5_ = ^3^ *J*_5_,_6b_ = 10.0 Hz, ^3^ *J*_5,6a_ = 6.1 Hz, H-5), 3.69-3.63 (m, 2H, H-5′, H-6′b), 3.64 (dd, 1H, ^3^ *J*_2′,3′_ = 9.0 Hz, ^3^ *J*_1′,2′_ = 3.2 Hz, H-2′), 3.56 (td, 1H, ^3^ *J*_3,4_ = ^3^ *J*_4,5_ = 9.4 Hz, H-4), 3.40 (t, 1H, ^3^ *J*_4′,5′_ = ^3^ *J*_3′,4′_ = 9.0 Hz, H-4′), 2.81-2.63 (m, 4H, 2xC*H*_2_, Lev), 2.34 (d, 1H, C4-O*H*), 1.93 (s, 3H, C*H*_3_, Lev), 0.93, 0.91, 0.81 (3×s, 27H, 3×[C*H*_3_]_3_C*,* 3xTBDMS), 0.17, 0.16, 0.13, 0.10, 0.03, 0.02 (6×s, 18H, 6×C*H*_3_, 3xTBDMS); ^13^C NMR (151 MHz, CDCl_3_): δ[ppm] = 206.01 (*C*H_3_C = O, Lev), 172.40 (C=O, Lev), 155.28 (C=O, Alloc), 137.17 (*C*_*q*_, Ph), 131.37 (CH, Alloc), 129.08, 128.11, 126.37 (CH, Ph), 119.03 (*C*H_2_, Alloc), 102.51 (Ph*C*H), 94.99 (C-1′), 90.67 (C-1), 82.44 (C-4′), 73.90 (C-2′), 73.42 (C-2), 71.91 (C-3), 71.45 (C-3′), 70.65 (C-4), 69.98 (C-5′), 68.81 (C-6′), 68.70 (*C*H_2_, Alloc), 66.19 (C-6), 63.72 (C-5′), 37.57 (CH_2_, Lev), 29.58 (CH_3_, Lev), 27.87 (CH_2_, Lev), 26.40, 26.18, 25.78 ([*C*H_3_]_3_C, 3xTBDMS), 18.46, 18.15, 18.15 ([CH_3_]_3_ *C,* 3xTBDMS), −3.01, −4.01, −4.19, −4.33, −4.38, −4.46 (6xCH_3_, 3xTBDMS); HRMS (^+^ESI) m/z: calcd. for C_46_H_82_O_15_Si_3_ [M + NH_4_]^+^ 972.4987, found 972.4991; calcd. for C_46_H_78_KO_15_Si_3_ [M + K]^+^ 993.4280, found 993.4295.

**4,6-*O*-Benzylidene-2,3-di-*O*-*tert*-butyldimethylsilyl-α-D-glucopyrano-syl-(1↔1)-6-*O*-allyloxycarbonyl-3-*O*-*tert*-butyldimethylsilyl-2-*O*-(4-oxopentanoyl)-4-*O*-(2,2,2-trichloroethoxycarbonylamino)-α-D-glucopyranoside (17).** To a stirred solution of **15** (0.800 g, 0.837 mmol) in dry CH_2_Cl_2_ (8 mL, c = 0.110 M), pyridine (500 μL, 5.86 mmol) and 2,2,2-trichloroethyl chloroformate (600 μL, 4.19 mmol) were added successively at 0°C. The reaction mixture was stirred for 16 h at r. t., then diluted with toluene (20 mL) and concentrated. The residue was dissolved in EtOAc (200 mL) and washed with aq. satd. NaHCO_3_ (3 × 50 mL), aq. satd. CuSO_4_ (3 × 50 mL), and brine (2 × 50 mL). The organic layer was dried over Na_2_SO_4,_ filtered, and concentrated. The residue was purified by column chromatography on silica gel (toluene-EtOAc, 98/2 → 75/25) to afford **17** (0.927 g, 98%) as a solid. R_f_ = 0.58 (toluene–EtOAc, 90/10, v/v); ${\left[\alpha \right]}_{D}^{20}$ = 69 (c = 1.0, CHCl_3_); ^1^H NMR (600 MHz, CDCl_3_): δ [ppm] = 7.38-7.32 (m, 5 H, *Ph*CH), 5.91 (m, 1H, C*H*, Alloc), 5.41 (s, 1H, PhC*H*), 5.38-5.24 (m, 2H, C*H*_2_, Alloc), 5.37 (d, 1H, ^3^ *J*_1,2_ = 3.1 Hz, H-1), 5.01 (d, 1H, ^3^ *J*_1′,2′_ = 2.9 Hz, H-1′), 4.90 (t, 1H, ^3^ *J*_3,4_ = ^3^ *J*_4,5_ = 9.5 Hz, H-4), 4.88-4.60 (m, 2H, C*H*_2_, Troc), 4.82 (dd, 1H, ^3^ *J*_2,3_ = 9.8 Hz, ^3^ *J*_1,2_ = 3.3 Hz, H-2), 4.61 (m, 2H, C*H*_2_, Alloc), 4.36 (t, 1H, ^3^ *J*_2,3_ = ^3^ *J*_3,4_ = 9.4 Hz, H-3), 4.28-4.20 (m, 3H, H-5, H-6a, H-6b), 4.17 (m, 1H, H-6′a), 4.12 (t, 1H, ^3^ *J*_2′,3′_ = ^3^ *J*_3′,4′_ = 8.8 Hz, H-3′), 3.69-3.63 (m, 2H, H-5, H-6′b), 3.65 (dd, 1H, ^3^ *J*_3′,2′_ = 8.8 Hz, ^3^ *J*_1′,2′_ = 2.9 Hz, H-2′), 3.41 (t, 1H, ^3^ *J*_3′,4′_ = ^3^ *J*_4′,5′_ = 8.7 Hz, H-4′), 2.82-2.61 (m, 4H, 2xC*H*_2_, Lev), 1.94 (s, 3H, C*H*_3_, Lev), 0.93, 0.87, 0.81 (3×s, 27H, 3×[C*H*_3_]_3_C*,* 3xTBDMS), 0.16, 0.14, 0.12, 0.09, 0.04, 0.03 (6×s, 18H, 6×C*H*_3_, 3xTBDMS); ^13^C NMR (151 MHz, CDCl_3_): δ [ppm] = 205.87 (*C*H_3_C = O, Lev), 172.27 (C=O, Lev), 154.64 (C=O, Alloc), 153.29 (C=O, Troc), 137.10 (*C*_*q*_, Ph), 131.38 (*C*H, Alloc), 129.12, 128.12, 126.36 (CH, Ph), 118.93 (CH_2_, Alloc), 102.56 (Ph*C*H), 95.11 (C-1′), 94.05 (*C*Cl_3_, Troc), 90.26 (C-1), 82.37 (C-4′), 77.08 (*C*H_2_, Troc), 76.00 (C-4), 73.82 (C-2′), 73.34 (C-2), 71.44 (C-3′), 69.10 (C-3), 68.75 (CH_2_, Alloc), 68.70 (C-6′), 67.83 (C-5), 65.35 (C-6), 63.84 (C-5′), 37.53 (CH_2_, Lev), 29.55 (CH_3_, Lev), 27.81 (CH_2_, Lev), 26.35, 26.19, 25.55 ([*C*H_3_]_3_C, 3xTBDMS), 18.46, 18.07, 17.91 ([CH_3_]_3_ *C,* 3xTBDMS), −2.96, −4.04, −4.18, −4.34, −4.52, −4.84 (6xCH_3_, 3xTBDMS); HRMS (^+^ESI) m/z: calcd. for C_49_H_79_Cl_3_KO_17_Si_3_ [M + K]^+^: 1167.3322, found 1167.3330; calcd. for C_49_H_83_Cl_3_NO_17_Si_3_ [M + NH_4_]^+^ 1146.4029, found 1146.4044.

**4,6-*O*-Benzylidene-α-D-glucopyranosyl-(1↔1)-6-*O*-allyloxycarbonyl-2-*O*-(4-oxopentanoyl)-4-*O*-(2,2,2-trichloroethoxycarbonylamino)-α-D-glucopyranoside (18).** To a stirred solution of triethylamine trihydrofluoride 3HF·Et_3_N (200 μL, 0.908 M) in dry DMF (200 µL), a solution of **22** (50.0 mg, 0.044 mmol) in dry DMF (100 µL) was added dropwise at 0°C (the final concentration of 3HF·Et_3_N in the reaction solution 1.2 M). The reaction mixture was stirred for 9 h at r. t. The reaction mixture was diluted with EtOAc (50 mL) and washed with aq. satd. NaHCO_3_ (2 × 10 mL), aq. satd. CaCl_2_ (2 × 10 mL), and brine (2 × 10 mL). The organic layer was dried over Na_2_SO_4,_ filtered, and concentrated. The residue was purified by column chromatography on silica gel (toluene-EtOAc, 80/20 → 40/60) to afford **18** (26.8 mg, 77%) as a syrup. R_f_ = 0.12 (toluene–EtOAc, 1:1, v/v); ${\left[\alpha \right]}_{D}^{20}$ = 85 (c = 0.6, CHCl_3_); ^1^H NMR (600 MHz, MeOD): δ [ppm] = 7.51-7.47 (m, 2 H, *Ph*CH), 7.38-7.34 (m, 3 H, *Ph*CH), 5.97 (m, 1H, C*H*, Alloc), 5.60 (s, 1H, PhC*H*), 5.40-5.25 (m, 2H, C*H*_2_, Alloc), 5.36 (d, 1H, ^3^ *J*_1,2_ = 3.4 Hz, H-1), 5.11 (d, 1H, ^3^ *J*_1′,2′_ = 3.8 Hz, H-1′), 4.86 (t, 1H, ^3^ *J*_3,4_ = ^3^ *J*_4,5_ = 9.4 Hz, H-4), 4.96-4.83 (m, 2H, C*H*_2_, Troc), 4.81 (dd, 1H, ^3^ *J*_2,3_ = 10.1 Hz, ^3^ *J*_1,2_ = 3.7 Hz, H-2), 4.63 (m, 2H, C*H*_2_, Alloc), 4.42 (dt, 1H**,** ^3^ *J*_5,4_ = ^3^ *J*_6b,5_ = 10.2 Hz, ^3^ *J*_5,6a_ = 3.4 Hz, H-5), 4.31 (t, ^3^ *J*_2,3_ = ^3^ *J*_3,4_ = 10.1 Hz, H-3), 4.32-4.27 (m, 2H, H-6a, H-6b), 4.25 (dd, 1H, ^2^ *J*_6′a,6′b_ = 10.1 Hz, ^3^ *J*_5′,6′a_ = 4.8 Hz, H-6′a), 3.92 (t, 1H, ^3^ *J*_3′,2′_ = ^3^ *J*_3′,4′_ = 9.4 Hz, H-3′), 3.84 (td, 1H, ^3^ *J*_5′,4′_ = ^3^ *J*_6′b,5′_ = 9.8 Hz, ^3^ *J*_5′,6′a_ = 4.6 Hz, H-5′), 3.77 (t, 1H, ^2^ *J*_6′a,6′b_ = ^3^ *J*_5′,6′b_ = 10.3 Hz, H-6′b), 3.64 (dd, 1H, ^3^ *J*_3′,2′_ = 9.4 Hz, ^3^ *J*_1′,2′_ = 3.8 Hz, H-2′), 3.53 (t, 1H, ^3^ *J*_3′,4′_ = ^3^ *J*_4′,5′_ = 9.4 Hz, H-4′), 2.85-2.65 (m, 4H, 2xC*H*_2_, Lev), 2.05 (s, 3H, C*H*_3_, Lev); ^13^C NMR (151 MHz, MeOD): δ [ppm] = 209.08 (CH_3_ *C* = O, Lev), 173.82 (C=O, Lev), 156.16 (C=O, Alloc), 155.06 (C=O, Troc), 139.09 (*C*_*q*_, Ph), 133.16 (*C*H, Alloc), 129.96, 129.10, 127.47 (CH, Ph), 118.94 (*C*H_2_, Alloc), 103.00 (Ph*C*H), 96.67 (C-1′), 95.90 (*C*Cl_3_, Troc), 92.68 (C-1), 82.59 (C-4′), 78.07 (*C*H_2_, Troc), 76.85 (C-4), 74.29 (C-2), 73.37 (C-2′), 71.76 (C-3′), 69.44 (C-5), 68.83 (C-6′), 68.70 (CH_2_, Alloc), 68.87 (C-5), 66.73 (C-6), 64.81 (C-5′), 38.52 (CH_2_, Lev), 29.64 (CH_3_, Lev), 28.85 (CH_2_, Lev); HRMS (+ESI) m/z: calcd. for C_31_H_38_Cl_3_O_17_ [M + H]^+^ 789.1147, found 789.1152; calcd. for C_31_H_41_Cl_3_NO_17_ [M + NH_4_]^+^ 806.1413, found 806.1412.

**4,6-*O*-Benzylidene-2,3-*O*-(tetra*iso*propyldisiloxane-1,3-diyl)-α-D-glucopyranosyl-(1↔1)-6-*O*-allyloxycarbonyl-2-*O*-(4-oxopentanoyl)-4-*O*-(2,2,2-trichloroethoxycarbonyl)-α-D-glucopyranoside (19).** To a stirred solution of **18** (130 mg, 0.165 mmol) in dry pyridine/DMF (500 μL, 1:1), 1,3-dichloro-1,1,3,3-tetra*iso*propyldisiloxane (100 μL, 0.52M) was added dropwise. The reaction mixture was stirred for 60 min, diluted with hexane (1.0 mL), and purified by column chromatography on silica gel (hexane-acetone, 90/10 → 75/25) to afford **19** (121 mg, 71%) as a transparent sirup. R_f_ = 0.24 (hexane–acetone, 3:1, v/v); R_f_ = 0.42 (toluene–EtOAc, 4:1, v/v); R_f_ = 0.74 (toluene -MeOH, 90:10, v/v); ${\left[\alpha \right]}_{D}^{20}$ = 52 (c = 1.7, CHCl_3_); ^1^H NMR (600 MHz, CDCl_3_): δ [ppm] = 7.47-7.43 (m, 2H, *Ph*CH), 7.37-7.31 (m, 3H, *Ph*CH), 5.91 (m, 1H, C*H*, Alloc), 5.55 (s, 1H, PhC*H*), 5.38-5.26 (m, 2H, C*H*_2_, Alloc), 5.31 (d, 1H, ^3^ *J*_1,2_ = 3.6 Hz, H-1), 5.11 (d, 1H**,** ^3^ *J*_1′,2′_ = 4.0 Hz, H-1′), 4.93 (dd, 1H, ^3^ *J*_2,3_ = 9.9 Hz, ^3^ *J*_1,2_ = 4.0 Hz, H-2), 4.91 (t, 1H, ^3^ *J*_3,4_ = ^3^ *J*_4,5_ = 10.0 Hz, H-4), 4.88-4.70 (m, 2H, C*H*_2_, Troc), 4.43 (m, 1H, ^3^ *J*_5,4_ = ^3^ *J*_6b,5_ = 10.2 Hz, ^3^ *J*_5,6a_ = 4.5 Hz, H-5), 4.31-4.20 (m, 4H, H-3, H-6a, H-6b, H-6′a), 4.11 (t, 1H**,** ^3^ *J*_2′,3′_ = ^3^ *J*_3′,4′_ = 8.7 Hz, H-3′), 3.88 (dd, 1H, ^3^ *J*_3′,2′_ = 8.5 Hz, ^3^ *J*_1′,2′_ = 4.0 Hz, H-2′), 3.85 (td, 1H, ^3^ *J*_5′,4′_ = ^3^ *J*_6′b,5′_ = 10.0 Hz, ^3^ *J*_5′,6′a_ = 4.8 Hz, H-5′), 3.72 (t, 1H, ^2^ *J*_6′a,6′b_ = ^3^ *J*_5′,6′b_ = 10.4 Hz, H-6′b), 3.53 (t, 1H, ^3^ *J*_3′,4′_ = ^3^ *J*_4′,5′_ = 9.3 Hz, H-4′), 2.79 (d, 1H, C3*-*O*H*), 2.74-2.54 (m, 4H, 2xC*H*_2_, Lev), 2.00 (s, 3H, C*H*_3_, Lev), 1.15-0.90 (m, 28H, TIPDS); ^13^C NMR (151 MHz, CDCl_3_): δ [ppm] = 207.16 (*C*H_3_C = O, Lev), 172.21 (C=O, Lev), 154.72 (C=O, Alloc), 153.66 (C=O, Troc), 137.57 (*C*_*q*_, Ph), 131.36 (*C*H, Alloc), 128.69, 128.06, 125.82 (CH, Ph), 119.03 (*C*H_2_, Alloc), 101.07 (Ph*C*H), 94.51 (C-1′), 94.31 (*C*Cl_3_, Troc), 91.16 (C-1), 81.02 (C-4′), 76.95 (*C*H_2_, Troc), 75.07 (C-2′), 75.01 (C-4), 73.43 (C-3′), 72.74 (C-2), 69.64 (C-3), 68.75 (C-6′, CH_2_, Alloc), 67.33 (C-5), 65.20 (C-6), 62.94 (C-5′), 38.04 (CH_2_, Lev), 29.49 (CH_3_, Lev), 28.01 (CH_2_, Lev), 17.39-17.01 (8xCH_3_, TIPDS), 12.73, 12.69, 12.20, 11.93 (4xCH, TIPDS). HRMS (^+^ESI) m/z: calcd. for C_43_H_69_Cl_3_NO_18_Si_2_ [M + NH_4_]^+^ 1048.3119, found 1048.2952.

**4,6-*O*-Benzylidene-3-*O*-*tert*-butyldimethylsilyl-2-*O*-(4-oxopentanoyl)-α-D-glucopyranosyl-(1↔1)-6-*O*-allyloxycarbonyl-2,3-di-*O*-*tert*-butyldimethylsilyl-α-D-glucopyranoside (21).** To a stirred solution of **13** (2.164 g, 2.48 mmol) in dry CH_2_Cl_2_ (10 mL, c = 0.250M), *sym*-collidine (3.30 mL, 24.8 mmol) and allyloxycarbonyl chloride (2.70 mL, 5.23 mmol) were added successively at 0°C. The reaction mixture was stirred for 6 h at r. t., then diluted with toluene (20 mL) and concentrated. The residue was dissolved in EtOAc (400 mL) and washed with aq. satd. NaHCO_3_ (3 × 100 mL), aq. satd. CuSO_4_ (3 × 50 mL), and brine (2 × 100 mL). The organic layer was dried over Na_2_SO_4,_ filtered, and concentrated. The residue was purified by column chromatography on silica gel (toluene-EtOAc, 90/10 → 80/20) to afford **21** (2.09 g, 88%) as a transparent syrup. R_f_ = 0.75 (toluene–EtOAc, 3/2, v/v); R_f_ = 0.17 (toluene–EtOAc, 90/10, v/v); ${\left[\alpha \right]}_{D}^{20}$ = 69 (c = 0.5, CHCl_3_); ^1^H NMR (600 MHz, CDCl_3_): δ [ppm] = 7.46-7.42 (m, 2H, *Ph*CH), 7.37-7.33 (m, 3H, *Ph*CH), 5.94 (m, 1H, C*H*, Alloc), 5.48 (s, 1H, PhC*H*), 5.39-5.24 (m, 2H, C*H*_2_, Alloc), 5.34 (d, 1H, ^3^ *J*_1′,2′_ = 3.7 Hz, H-1′), 5.03 (d, 1H, ^3^ *J*_1,2_ = 3.1 Hz, H-1), 4.72 (dd, 1H, ^3^ *J*_2′,3′_ = 9.4 Hz, ^3^ *J*_1′,2′_ = 3.8 Hz, H-2′), 4.64 (m, 2H, C*H*_2_, Alloc), 4.38-4.35 (m, 2H, H-6b, H-6a), 4.23 (t, 1H, ^3^ *J*_2′,3′_ = ^3^ *J*_3′,4′_ = 9.0 Hz, H-3′), 4.21 (t, 1H, ^2^ *J*_6′a,6′b_ = 10.4 Hz, ^3^ *J*_5′,6′a_ = 4.8 Hz, H-6′a), 4.06 (t, 1H, ^3^ *J*_2,3_ = ^3^ *J*_3,4_ = 8.3 Hz, H-3), 3.97 (td, 1H, ^3^ *J*_4′,5′_ = ^3^ *J*_5′,6′b_ = 10.0 Hz, ^3^ *J*_5′,6′a_ = 4.9 Hz, H-5′), 3.71 (t, 1H, ^2^ *J*_6′a,6′b_ = ^3^ *J*_5′,6′b_ = 10.4 Hz, H-6′b), 3.64 (m, 1H, ^3^ *J*_4,5_ = ^3^ *J*_5_,_6b_ = 9.2 Hz, ^3^ *J*_5,6a_ = 3.1 Hz, H-5), 3.61 (dd, 1H, ^3^ *J*_2,3_ = 8.7 Hz, ^3^ *J*_1,2_ = 3.1 Hz, H-2), 3.50 (t, 1H, ^3^ *J*_3′,4′_ = ^3^ *J*_4′,5′_ = 9.4 Hz, H-4′), 3.39 (t, 1H, ^3^ *J*_3,4_ = ^3^ *J*_4,5_ = 8.3 Hz, H-4), 2.96-2.43 (m, 4H, 2xC*H*_2_, Lev), 2.20 (s, 3H, C*H*_3_, Lev), 0.97, 0.94, 0.81 (3×s, 27H, 3×[C*H*_3_]_3_C*,* 3xTBDMS), 0.15, 0.14, 0.11, 0.05, 0.03 (6×s, 18H, 6×C*H*_3_, 3xTBDMS); ^13^C NMR (151 MHz, CDCl_3_): δ [ppm] = 207.97 (*C*H_3_C = O, Lev), 172.33 (C=O, Lev), 155.12 (C=O, Alloc), 137.22 (*C*_*q*_, Ph), 131.63 (CH, Alloc), 128.97, 128.03, 126.42 (CH, Ph), 118.89 (CH_2_, Alloc), 102.24 (Ph*C*H), 92.96 (C-1), 90.65 (C-1′), 81.78 (C-4′), 74.60 (C-2′), 74.31 (C-3), 72.91 (C-2), 71.26 (C-4), 71.27 (C-5), 69.33 (C-3′), 68.76 (C-6′), 68.62 (*C*H_2_, Alloc), 66.67 (C-6), 62.88 (C-5′), 37.65 (CH_2_, Lev), 29.81 (CH_3_, Lev), 27.70 (CH_2_, Lev), 26.53, 26.28, 25.68 ([*C*H_3_]_3_C, 3xTBDMS), 3 × 18.11 ([CH_3_]_3_ *C,* 3xTBDMS), −3.15, −3.74, 2x-4.06, −4.49, −4.89 (6xCH_3_, 3xTBDMS); HRMS (^+^ESI) m/z: calcd. for C_46_H_79_O_15_Si_3_ [M + H]^+^: 955.4721, found 955.4720; calcd. for C_46_H_78_KO_15_Si_3_ [M + K]^+^ 993.4280, found 993.4294.

**4,6-*O*-Benzylidene-3-*O*-*tert*-butyldimethylsilyl-2-*O*-(4-oxopentanoyl)-α-D-glucopyranosyl-(1↔1)-6-*O*-allyloxycarbonyl-2,3-di-*O*-*tert*-butyldimethylsilyl-4-*O*-(2,2,2-trichloroethoxycarbonylamino)-α-D-glucopyranoside (23).** To a stirred solution of **21** (2.09 g, 2.40 mmol) in dry CH_2_Cl_2_ (4 mL, c = 0.126 M), pyridine (1.25 mL, 15.29 mmol) and 2,2,2-trichloroethyl chloroformate (1.50 mL, 10.92 mmol) were added successively at 0°C. The reaction mixture was stirred for 16 h at r. t., then diluted with toluene (30 mL) and concentrated. The residue was dissolved in EtOAc (400 mL) and washed with aq. satd. NaHCO_3_ (3 × 100 mL), aq. satd. CuSO_4_ (2 × 50 mL), and brine (3 × 50 mL). The organic layer was dried over Na_2_SO_4,_ filtered, and concentrated. The residue was purified by column chromatography on silica gel (stepwise gradient elution, toluene-EtOAc, 98/2 → 75/25) to afford **23** (2.32 g, 96%) as a solid. R_f_ = 0.28 (toluene–EtOAc, 90/10, v/v); ${\left[\alpha \right]}_{D}^{20}$ = 56 (c = 1.2, MeOH); ^1^H NMR (600 MHz, MeOD): δ [ppm] = 7.46-7.42 (m, 2H, *Ph*CH), 7.38-7.33 (m, 3H, *Ph*CH), 5.93 (m, 1H, C*H*, Alloc), 5.49 (s, 1H, PhC*H*), 5.39-5.25 (m, 2H, C*H*_2_, Alloc), 5.24 (d, 1H, ^3^ *J*_1′,2′_ = 3.5 Hz, H-1′), 5.11 (d, 1H, ^3^ *J*_1,2_ = 2.8 Hz, H-1), 4.92 (dd, 1H**,** ^3^ *J*_3′,2′_ = 9.5 Hz, ^3^ *J*_1′,2′_ = 3.5 Hz, H-2′), 4.88-4.54 (m, 2H, C*H*_2_, Troc), 4.80 (t, 1H, ^3^ *J*_3,4_ = ^3^ *J*_4,5_ = 9.4 Hz, H-4), 4.62 (m, 2H, C*H*_2_, Alloc), 4.31-4.25 (m, 3H, H-3, H-6a, H-6b), 4.24 (t, 1H, ^3^ *J*_2′,3′_ = ^3^ *J*_3′,4′_ = 9.3 Hz, H-3′), 4.21 (t, 1H, ^2^ *J*_6′a,6′b_ = 10.5 Hz, ^3^ *J*_5′,6′a_ = 4.8 Hz, H-6′a), 4.04 (td, 1H, ^3^ *J*_5′,4′_ = ^3^ *J*_6′b,5′_ = 9.9 Hz, ^3^ *J*_5′,6′a_ = 4.8 Hz H-5′), 3.98 (m, 1H, ^3^ *J*_5,4_ = ^3^ *J*_6b,5_ = 9.8 Hz, ^3^ *J*_5,6a_ = 3.7 Hz, H-5), 3.72 (dd, 1H, ^3^ *J*_2,3_ = 9.0 Hz, ^3^ *J*_1,2_ = 2.9 Hz, H-2), 3.72 (t, 1H, ^2^ *J*_6′a,6′b_ = ^3^ *J*_5′,6′b_ = 10.2 Hz, H-6′b), 3.52 (t, 1H, ^3^ *J*_3′,4′_ = ^3^ *J*_4′,5′_ = 9.4 Hz, H-4′), 2.84-2.56 (m, 4H, 2xC*H*_2_, Lev), 2.15 (s, 3H, C*H*_3_, Lev), 0.98, 0.89, 0.82 (3×s, 27H, 3×[C*H*_3_]_3_C*,* 3xTBDMS), 0.16, 0.15, 0.14, 0.14, 0.05, −0.03 (6×s, 18H, 6×C*H*_3_, 3xTBDMS); ^13^C NMR (151 MHz, MeOD): δ [ppm] = 205.61 (*C*H_3_C = O, Lev), 172.10 (C=O, Lev), 154.64 (C=O, Alloc)), 153.43 (C=O, Troc), 137.22 (*C*_*q*_, Ph), 131.58 (*C*H, Alloc), 129.00, 128.05, 126.45 (CH, Ph), 119.01 (*C*H_2_, Alloc), 102.31 (Ph*C*H), 94.12 (*C*Cl_3_, Troc), 93.62 (C-1), 91.43 (C-1′), 81.90 (C-4′), 77.08 (*C*H_2_, Troc), 76.65 (C-4), 73.59 (C-2′), 73.00 (C-2), 71.09 (C-3), 69.36 (C-3′), 68.81 (C-6′), 68.72 (CH_2_, Alloc), 68.60 (C-5), 66.03 (C-6), 63.41 (C-5′), 37.51 (CH_2_, Lev), 29.74 (CH_3_, Lev), 27.76 (CH_2_, Lev), 26.63, 26.03, 25.66 ([*C*H_3_]_3_C, 3xTBDMS), 18.39, 18.15, 18.07 ([CH_3_]_3_ *C,* 3xTBDMS), −3.04, −3.81, −4.21, −4.39, −4.47, −4.92 (6xCH_3_, 3xTBDMS); HRMS (^+^ESI) m/z: calcd. for C_49_H_79_Cl_3_OK_17_Si_3_ [M + K]^+^ 1169.3309, found 1169.3317.

**4,6-*O*-Benzylidene-2-*O*-(4-oxopentanoyl)-α-D-glucopyranosyl-(1↔1)-6-*O*-allyloxycarbonyl-4-*O*-(2,2,2-trichloroethoxycarbonyl)-α-D-gluco-pyranoside (24).** To a stirred solution of triethylamine trihydrofluoride 3HF·Et_3_N (200 µL) in dry DMF (200 µL), a solution of **23** (50 mg, 0.044 mmol) in dry DMF (100 µL) was added dropwise at 0 °C. The reaction mixture was stirred for 9 h at r. t. under atmosphere of Ar, diluted with EtOAc (30 mL), and washed with aq. satd. NaHCO_3_ (2 × 10 mL), aq. satd. CaCl_2_ (2 × 10 mL), and brine (2 × 10 mL). The organic layer was dried over Na_2_SO_4,_ filtered, and concentrated. The residue was purified by column chromatography on silica gel (toluene-EtOAc, 80/20 → 40/60) to afford **24** (28.0 mg, 80%) as a transparent solid. R_f_ = 0.12 (toluene-EtOAc, 1:1, v/v); R_f_ = 0.02 (toluene-EtOAc, 8:2, v/v); ${\left[\alpha \right]}_{D}^{20}$ = 86 (c = 0.2, MeOH); ^1^H NMR (600 MHz, MeOD): δ [ppm] = 7.51-7.47 (m, 2H, *Ph*CH), 7.37-7.32 (m, 3H, *Ph*CH), 5.96 (m, 1H, C*H*, Alloc), 5.60 (s, 1H, PhC*H*), 5.39-5.24 (m, 2H, C*H*_2_, Alloc), 5.22 (d, 1H, ^3^ *J*_1′,2′_ = 3.8 Hz, H-1′), 5.13 (d, 1H, ^3^ *J*_1,2_ = 3.7 Hz, H-1), 4.94-4.82 (m, 2H, C*H*_2_, Troc), 4.88 (dd, 1H, ^3^ *J*_3′,2′_ = 9.7 Hz, ^3^ *J*_1′,2′_ = 3.8 Hz, H-2′), 4.74 (t, 1H, ^3^ *J*_3′,4′_ = ^3^ *J*_4′,5′_ = 9.7 Hz, H-4′), 4.61 (m, 2H, C*H*_2_, Alloc), 4.28 (m, 2H, ^3^ *J*_5,6b_ = 3.4 Hz, H-6a, H-6b), 4.25 (dd, 1H, ^2^ *J*_6′a,6′b_ = 9.9 Hz, ^3^ *J*_5′,6′a_ = 5.0 Hz, H-6′a), 4.22 (t, 1H, ^3^ *J*_3′,2′_ = ^3^ *J*_3′,4′_ = 9.4 Hz, H-3′), 4.18 (td, 1H, ^3^ *J*_5′,4′_ = ^3^ *J*_6′b,5′_ = 9.9 Hz, ^3^ *J*_5′,6′a_ = 5.0 Hz, H-5′), 4.11 (dt, 1H**,** ^3^ *J*_5,4_ = ^3^ *J*_6b,5_ = 10.2 Hz, ^3^ *J*_5,6a_ = 3.9 Hz, H-5), 3.98 (t, ^3^ *J*_2,3_ = ^3^ *J*_3,4_ = 9.5 Hz, H-3), 3.78 (t, 1H, ^2^ *J*_6′a,6′b_ = ^3^ *J*_5′,6′b_ = 10.1 Hz, H-6′b), 4.81 (dd, 1H, ^3^ *J*_2,3_ = 9.7 Hz, ^3^ *J*_1,2_ = 3.6 Hz, H-2), 3.60 (t, 1H, ^3^ *J*_3,4_ = ^3^ *J*_4,5_ = 9.5 Hz, H-4), 2.90-2.63 (m, 4H, 2xC*H*_2_, Lev), 2.17 (s, 3H, C*H*_3_, Lev); ^13^C NMR (151 MHz, MeOD): δ [ppm] = 208.96 (CH_3_ *C* = O, Lev), 173.86 (C=O, Lev), 156.14 (C=O, Alloc), 155.13 (C=O, Troc), 139.07 (*C*_*q*,_ Ph), 133.19 (*C*H, Alloc), 129.94, 129.02, 127.53 (CH, Ph), 118.92 (*C*H_2_, Alloc), 103.10 (Ph*C*H), 95.46 (C-1′), 93.68 (C-1), 82.57 (C-4′), 78.10 (*C*H_2_, Troc), 77.58 (C-4), 74.68 (C-2), 72.61 (C-2′), 72.00 (C-3′), 69.68 (C-6′), 69.67 (CH_2_, Alloc), 69.36 (C-5), 67.40 (C-6), 64.26 (C-5′), 39.52 (CH_2_, Lev), 29.80 (CH_3_, Lev), 28.77 (CH_2_, Lev); HRMS (+ESI) m/z: calcd. For C_31_H_38_Cl_3_O_17_ [M + H]^+^ 789.1147, found 789.1152; calcd. For C_31_H_41_Cl_3_NO_17_ [M + NH_4_]^+^ 806.1413, found 806.1412.

**4,6-*O*-Benzylidene-2-*O*-(4-oxopentanoyl)-α-D-glucopyranosyl-(1↔1)-6-*O*-allyloxycarbonyl-2,3-*O*-(tetra*iso*propyldisiloxane-1,3-diyl)-4-*O*-(2,2,2-trichloroethoxycarbonyl)-α-D-glucopyranoside (25).** To a stirred solution of **24** (20 mg, 0.025 mmol) in dry pyridine/DMF (200 μL, 1:1) 1,3-dichloro-1,1,3,3-tetra*iso*propyldisiloxane (40 μL, 0.52M) was added dropwise. The reaction mixture was stirred for 60 min, diluted with hexane (500 µL), and purified by column chromatography on silica gel (hexane-acetone, 90/10 → 75/25) to give **25** (10.8 mg, BRSM 82%) as transparent oil. Unreacted **24** (10.2 mg) was recovered. R_f_ = 0.22 (hexane–acetone, 3:1, v/v); R_f_ = 0.58 (toluene–MeOH, 90:10, v/v); ${\left[\alpha \right]}_{D}^{20}$ = 72 (c = 1.1, CHCl_3_); ^1^H NMR (600 MHz, CDCl_3_): δ [ppm] = 7.49-7.45 (m, 2H, *Ph*CH), 7.39-7.34 (m, 3 H, *Ph*CH), 5.93 (m, 1H, CH, Alloc), 5.53 (s, 1H, PhC*H*), 5.39-5.26 (m, 2H, CH_2_, Alloc), 5.22 (d, 1H, ^3^ *J*_1′,2′_ = 3.7 Hz, H-1′), 5.15 (d, 1H, ^3^ *J*_1,2_ = 3.8 Hz, H-1), 5.00 (dd, 1H, ^3^ *J*_3′,2′_ = 9.7 Hz, ^3^ *J*_1′,2′_ = 3.7 Hz, H-2′), 4.89 (t, 1H, ^3^ *J*_3,4_ = ^3^ *J*_4,5_ = 9.6 Hz, H-4), 4.89 (m, 2H, CH_2_, Troc), 4.62 (m, 2H, CH_2_, Alloc), 4.35 (dd, 1H, ^2^ *J*_6a,6b_ = 12.2 Hz, ^3^ *J*_5,6a_ = 4.8 Hz, H-6a), 4.28 (dd, 1H, ^2^ *J*_6a,6b_ = 12.0 Hz, ^3^ *J*_5,6b_ = 2.6 Hz, H-6b), 4.24-4.19 (m, 4H, H-3, H-3′, H-5′, H-6′a), 4.17 (m, 1H, ^3^ *J*_5,4_ = ^3^ *J*_6b,5_ = 10.5 Hz, ^3^ *J*_5,6a_ = 4.7 Hz, H-5), 3.90 (dd, 1H, ^3^ *J*_2,3_ = 8.9 Hz, ^3^ *J*_1,2_ = 3.9 Hz, H-2), 3.74 (t, 1H, ^2^ *J*_6a,6′b_ = ^3^ *J*_5′,6′b_ = 11.8 Hz, H-6′b), 3.60 (t, 1H, ^3^ *J*_3′,4′_ = ^3^ *J*_4′,5′_ = 9.2 Hz, H-4′), 2.88-2.61 (m, 4H, 2xC*H*_2_, Lev), 2.70 (d, 1H, C3′-O*H*), 2.18 (s, 3H, C*H*_3_, Lev), 1.12-0.97 (m, 28H, [C*H*_3_]_3_C*,* TIPDS); ^13^C NMR (151 MHz, CDCl_3_): δ [ppm] = 206.62 (*C*H_3_C = O, Lev), 172.17 (C=O, Lev), 154.71 (C=O, Alloc), 153.31 (C=O, Troc), 137.03 (*C*_*q*,_ Ph), 131.47 (*C*H, Alloc), 129.25, 128.24, 126.45 (CH, Ph), 119.08 (*C*H_2_, Alloc), 102.28 (Ph*C*H), 93.78 (C-1), 92.53 (C-1′), 81.31 (C-4′), 77.12 (*C*H_2_, Troc), 75.80 (C-4), 74.23 (C-3), 73.99 (C-2), 72.93 (C-2′), 68.80 (CH_2_, Alloc), 68.80 (C-6′), 68.73 (C-3′), 67.34 (C-5), 65.79 (C-6), 62.57 (C-5′), 37.98 (CH_2_, Lev), 29.72 (CH_3_, Lev), 27.97 (CH_2_, Lev), 17.35–17.07 (8xCH_3_, TIPDS), 12.78, 12.66, 12.06, 11.73 (4xCH, TIPDS). HRMS (^+^ESI) m/z: calcd. for C_43_H_69_Cl_3_NO_18_Si_2_ [M + NH_4_]^+^ 1048.3119, found 1048.2949.

**4,6-*O*-Benzylidene-2,3-di-*O*-*tert*-butyldimethylsilyl-α-D-glucopyranosyl-(1↔1)-6-*O*-allyloxycarbonyl-2-*O*-(4-oxopentanoyl)-4-*O*-(2,2,2-trichloroethoxycarbonyl)-α-D-glucopyranosid (26)** and **4,6-*O*-Benzylidene-3-*O*-*tert*-butyldimethylsilyl-α-D-glucopyranosyl-(1↔1)-6-*O*-allyloxycarbonyl-3-*O*-*tert*-butyldimethylsilyl-2-*O*-(4-oxopentanoyl)-4-*O*-(2,2,2-trichloroethoxycarbonyl)-α-D-glucopyrano-side (27).** To a stirred solution of triethylamine trihydrofluoride 3HF·Et_3_N (500 µL) in dry DMF (2500 µL) in a PTFE reaction vessel, a solution of **17** (500 mg, 0.442 mmol) in dry DMF (2000 µL) was added dropwise to reach the final concentration of HF·Et_3_N (0.2 M) in the reaction solution (pH = 3.5). The reaction mixture was stirred for 70 min at r. t. under atmosphere of Ar, then diluted with EtOAc (150 mL) and washed with aq. satd. CaCl_2_ (2 × 30 mL), aq. satd. NaHCO_3_ (3 × 30 mL), and brine (2 × 30 mL). The organic layer was dried over Na_2_SO_4,_ filtered, and concentrated. The residue was purified by column chromatography on silica gel (toluene-EtOAc, 100/0 → 40/60) to give **26** (104 mg, 47%) and **27** (67 mg, 30%), overall yield 77%, the unreacted starting material **17** was fully recovered (256 mg).

**26:** R_f_ = 0.61 (toluene-EtOAc, 1/1, v/v); R_f_ = 0.32 (toluene-EtOAc, 4/1, v/v); ${\left[\alpha \right]}_{D}^{20}$ = 67 (c = 1.4, CHCl_3_); ^1^H NMR (600 MHz, CDCl_3_): δ [ppm] = 7.43-7.38 (m, 2H, *Ph*CH), 7.37-7.32 (m, 3H, *Ph*CH), 5.92 (m, 1H, C*H*, Alloc), 5.41 (s, 1H, PhC*H*), 5.39-5.25 (m, 2H, C*H*_2_, Alloc), 5.31 (d, 1H, ^3^ *J*_1,2_ = 3.7 Hz, H-1), 5.11 (d, 1H, ^3^ *J*_1′,2′_ = 3.2 Hz, H-1′), 5.00 (t, 1H, ^3^ *J*_3,4_ = ^3^ *J*_4,5_ = 9.4 Hz, H-4), 4.97 (dd, 1H, ^3^ *J*_2,3_ = 10.0 Hz, ^3^ *J*_1,2_ = 3.8 Hz, H-2), 4.84-4.77 (m, 2H, C*H*_2_, Troc), 4.62 (m, 2H, C*H*_2_, Alloc), 4.35 (m, 1H, ^3^ *J*_4,5_ = ^3^ *J*_5_,_6b_ = 9.8 Hz, ^3^ *J*_5,6a_ = 4.7 Hz, H-5), 4.34 (m, 1H, H-6a), 4.34 (d, 1H, ^3^ *J*_2,3_ = ^3^ *J*_3,4_ = 9.7 Hz, H-3), 4.26 (dd, 1H, ^2^ *J*_6a,6b_ = 13.4 Hz, ^3^ *J*_5,6b_ = 3.8 Hz, H-6b), 4.13 (t, 1H, ^2^ *J*_6′a,6′b_ = 10.3 Hz, ^3^ *J*_5′,6′a_ = 4.7 Hz, H-6′a), 4.02 (t, 1H, ^3^ *J*_2′,3′_ = ^3^ *J*_3′,4′_ = 8.9 Hz, H-3′), 3.89 (td, 1H, ^3^ *J*_4′,5′_ = ^3^ *J*_5′,6′b_ = 10.1 Hz, ^3^ *J*_5′,6′a_ = 4.7 Hz, H-5′), 3.67 (t, 1H, ^2^ *J*_6′a,6′b_ = ^3^ *J*_5′,6′b_ = 10.4 Hz, H-6′b), 3.67 (dd, 1H, ^3^ *J*_2′,3′_ = 8.9 Hz, ^3^ *J*_1′,2′_ = 3.3 Hz, H-2′), 3.40 (t, 1H, ^3^ *J*_3′,4′_ = ^3^ *J*_4′,5′_ = 9.2 Hz, H-4′), 2.84 (d, 1H, C3-O*H*), 2.80-2.60 (m, 4H, 2xC*H*_2_, Lev), 2.07 (s, 3H, C*H*_3_, Lev), 0.92, 0.80 (2×s, 18H, 2×[C*H*_3_]_3_C*,* 2xTBDMS), 0.13, 0.10, 0.07, 0.03 (4×s, 12H, 4×C*H*_3_, 3xTBDMS); ^13^C NMR (151 MHz, CDCl_3_): δ [ppm] = 207.30 (*C*H_3_C = O, Lev), 172.18 (C=O, Lev), 154.69 (C=O, Alloc), 153.52 (C=O, Troc), 137.13 (*C*_*q*_, Ph), 131.36 (*C*H, Alloc), 129.16, 128.12, 126.39 (CH, Ph), 118.98 (*C*H_2_, Alloc), 102.54 (Ph*C*H), 96.38 (C-1′), 94.24 (*C*Cl_3_, Troc), 91.54 (C-1), 82.59 (C-4′), 77.05 (*C*H_2_, Troc), 74.91 (C-4), 73.87 (C-2′), 72.69 (C-2), 71.51 (C-3′), 69.42 (C-5), 68.86 (C-6′), 68.78 (CH_2_, Alloc), 67.63 (C-3), 65.07 (C-6), 63.38 (C-5′), 38.18 (CH_2_, Lev), 29.64 (CH_3_, Lev), 28.01 (CH_2_, Lev), 26.33, 26.11 ([*C*H_3_]_3_C, 2xTBDMS), 18.37, 18.24 ([CH_3_]_3_ *C,* 2xTBDMS), −2.78, −4.11, −4.25, −4.30 (4xCH_3_, 2xTBDMS); HRMS (^−^ESI) m/z: calcd. for C_43_H_65_Cl_4_O_17_Si_2_ [M + Cl]^-^ 1049.2520, found 1049.2514.

**27**: R_f_ = 0.73 (toluene-EtOAc, 1/1, v/v); R_f_ = 0.58 (toluene-EtOAc, 4/1, v/v); ${\left[\alpha \right]}_{D}^{20}$ = 77 (c = 1.0, CHCl_3_); ^1^H NMR (600 MHz, CDCl_3_): δ [ppm] = 7.44-7.40 (m, 2H, *Ph*CH), 7.36-7.32 (m, 3H, *Ph*CH), 5.93 (m, 1H, *CH,* Alloc), 5.48 (s, 1H, PhC*H*), 5.39 (d, 1H, ^3^ *J*_1,2_ = 3.6 Hz, H-1), 5.38-5.25 (m, 2H, C*H*_2_, Alloc), 5.15 (d, 1H, ^3^ *J*_1′,2′_ = 3.7 Hz, H-1′), 4.88 (t, 1H, ^3^ *J*_3,4_ = ^3^ *J*_4,5_ = 9.6 Hz, H-4), 4.86-4.63 (m, 2H, C*H*_2_, Troc), 4.79 (dd, 1H, ^3^ *J*_2,3_ = 9.6 Hz, ^3^ *J*_1,2_ = 3.6 Hz, H-2), 4.62 (m, 2H, C*H*_2_, Alloc), 4.33 (m, 1H, ^3^ *J*_4,5_ = ^3^ *J*_5_,_6b_ = 10.3 Hz, ^3^ *J*_5,6a_ = 3.7 Hz, H-5), 4.30 (dd, 2H, ^2^ *J*_6a,6b_ = 9.4 Hz, ^3^ *J*_5,6_ = 2.7 Hz, H-6a, H-6b), 4.22 (dd, 1H, ^2^ *J*_6′a,6′b_ = 10.5 Hz, ^3^ *J*_5′,6′a_ = 5.6 Hz, H-6′a), 4.24 (t, 1H, ^3^ *J*_2,3_ = ^3^ *J*_3,4_ = 9.2 Hz, H-3), 3.96 (t, 1H, ^3^ *J*_2′,3′_ = ^3^ *J*_3′,4′_ = 9.0 Hz, H-3′), 3.73-3.64 (m, 3H, H-5′, H-6′b, H-2′), 3.46 (t, 1H, ^3^ *J*_3′,4′_ = ^3^ *J*_4′,5′_ = 9.0 Hz, H-4′), 2.77-2.59 (m, 4H, 2xC*H*_2,_ Lev), 2.15 (s, 1H, C2′-O*H*), 1.97 (s, 3H, C*H*_3_, Lev), 0.89, 0.86 (2×s, 18H, 2×[C*H*_3_]_3_C*,* 2xTBDMS), 0.13, 0.13, 0.12, 0.05 (4×s, 12H, 4×C*H*_3_, 3xTBDMS); ^13^C NMR (151 MHz, CDCl_3_): δ [ppm] = 205.86 (*C*H_3_C = O, Lev), 172.09 (C=O, Lev), 154.65 (C=O, Alloc), 153.25 (C=O, Troc), 137.15 (*C*_*q*_, Ph), 131.43 (*C*H, Alloc), 129.00, 128.12, 126.14 (CH, Ph), 119.10 (*C*H_2_, Alloc), 101.82 (Ph*C*H), 94.21 (C-1′), 93.90 (*C*Cl_3_, Troc), 90.98 (C-1), 81.41 (C-4′), 77.24 (*C*H_2_, Troc), 76.30 (C-4), 73.59 (C-2), 72.92 (C-2′), 72.46 (C-3′), 69.37 (C-3), 68.78 (CH_2_, Alloc), 68.67 (C-6′), 67.36 (C-5), 65.60 (C-6), 63.94 (C-5′), 37.51 (CH_2_, Lev), 29.54 (CH_3_, Lev), 27.80 (CH_2_, Lev), 25.89, 25.53 ([*C*H_3_]_3_C, 2xTBDMS), 18.31, 17.91 ([CH_3_]_3_ *C,* 2xTBDMS), −4.18, −4.51, −4.80, −4.82 (4xCH_3_, 2xTBDMS); HRMS (^−^ESI) m/z: calcd. for C_43_H_65_Cl_4_O_17_Si_2_ [M + Cl]^-^ 1049.2520, found 1049.2514.

**4,6-*O*-Benzylidene-2-*O*-(4-oxopentanoyl)-α-D-glucopyranosyl-(1↔1)-6-*O*-allyloxycarbonyl-2,3-di-*O*-*tert*-butyldimethylsilyl-4-*O*-(2,2,2-trichloroethoxycarbonyl)-α-D-glucopyranoside (28)** and **4,6-*O*-Benzylidene-3-*O*-*tert*-butyldimethylsilyl-2-*O*-(4-oxopentanoyl)-α-D-glucopyranosyl-(1↔1)-6-*O*-allyloxycarbonyl-3-*O*-*tert*-butyldimethylsilyl-4-*O*-(2,2,2-trichloroethoxycarbonyl)-α-D-glucopyranoside (29)** and **4,6-*O*-Benzylidene-3-*O*-*tert*-butyldimethyl-silyl-2-*O*-(4-oxopentanoyl)-α-D-glucopyranosyl-(1↔1)-6-*O*-allyloxycarbonyl-3-*O*-*tert*-butyldimethylsilyl-4-*O*-(2,2,2-trichloroethoxycarbonyl)-α-D-glucopyranoside (30)**. To a stirred solution of triethylamine trihydrofluoride 3HF·Et_3_N (500 µL) in dry DMF (2500 µL) in a PTFE reaction vessel, a solution of **23** (500 mg, 0.442 mmol) in dry DMF (2000 µL) was added dropwise to reach the final concentration of HF·Et_3_N (0.2 M) in the reaction solution (pH = 3.5). The reaction mixture was stirred for 70 min at r. t. under atmosphere of Ar, then diluted with EtOAc (150 mL) and washed with aq. satd. CaCl_2_ (2 × 30 mL), aq. satd. NaHCO_3_ (3 × 30 mL), and brine (2 × 30 mL). The organic layer was dried over Na_2_SO_4,_ filtered, and concentrated. The residue was purified by column chromatography on silica gel (toluene-EtOAc, 100/0 → 40/60) to afford **28** (80 mg, 53%), **29** (29 mg, 19%), and **30** (16 mg, 10%), the overall yield being 82%. The unreacted starting material **23** was fully recovered (331 mg).

**28:** R_f_ = 0.67 (toluene–EtOAc, 1/1, v/v); R_f_ = 0.25 (toluene–EtOAc, 4/1, v/v); ${\left[\alpha \right]}_{D}^{20}$ = 66 (c = 0.8, CHCl_3_); ^1^H NMR (600 MHz, CDCl_3_): δ [ppm] = 7.50-7.46 (m, 2H, *Ph*CH), 7.40-7.35 (m, 3H, *Ph*CH), 5.92 (m, 1H, C*H*, Alloc), 5.55 (s, 1H, PhC*H*), 5.39-5.26 (m, 2H, C*H*_2_, Alloc), 5.22 (d, 1H, ^3^ *J*_1′,2′_ = 3.8 Hz, H-1′), 5.11 (d, 1H, ^3^ *J*_1,2_ = 2.9 Hz, H-1), 5.02 (dd, 1H, ^3^ *J*_2′,3′_ = 9.6 Hz, ^3^ *J*_1′,2′_ = 3.8 Hz, H-2′), 4.90-4.54 (m, 2H, C*H*_2_, Troc), 4.76 (t, 1H, ^3^ *J*_3,4_ = ^3^ *J*_4,5_ = 9.3 Hz, H-4), 4.62 (m, 2H, C*H*_2_, Alloc), 4.27-4.21 (m, 3H, H-6′a, H-6a, H-6b), 4.23 (t, 1H, ^3^ *J*_2′,3′_ = ^3^ *J*_3′,4′_ = 9.5 Hz, H-3′), 4.20 (d, 1H, ^3^ *J*_2,3_ = ^3^ *J*_3,4_ = 8.9 Hz, H-3), 4.11 (td, 1H, ^3^ *J*_4′,5′_ = ^3^ *J*_5′,6′b_ = 9.9 Hz, ^3^ *J*_5′,6′a_ = 4.8 Hz, H-5′), 4.05 (dt, 1H, ^3^ *J*_4,5_ = ^3^ *J*_5_,_6b_ = 10.0 Hz, ^3^ *J*_5,6a_ = 4.2 Hz, H-5), 3.73 (t, 1H, ^2^ *J*_6′a,6′b_ = ^3^ *J*_5′,6′b_ = 10.6 Hz, H-6′b), 3.72 (dd, 1H, ^3^ *J*_2,3_ = 9.2 Hz, ^3^ *J*_1,2_ = 3.1 Hz, H-2), 3.62 (t, 1H, ^3^ *J*_3′,4′_ = ^3^ *J*_4′,5′_ = 9.5 Hz, H-4′), 2.87-2.61 (m, 4H, 2xC*H*_2_, Lev), 2.17 (s, 3H, C*H*_3_, Lev), 0.98, 0.88 (2×s, 18H, 2×[C*H*_3_]_3_C*,* 2xTBDMS), 0.17, 0.17, 0.15, 0.15 (4×s, 12H, 4×C*H*_3_, 2xTBDMS); ^13^C NMR (151 MHz, CDCl_3_): δ [ppm] = 206.93 (*C*H_3_C = O, Lev), 172.05 (C=O, Lev), 154.58 (C=O, Alloc), 153.38 (C=O, Troc), 136.93 (*C*_*q*_, Ph), 131.43 (*C*H, Alloc), 129.34, 128.33, 126.47 (CH, Ph), 119.16 (*C*H_2_, Alloc), 102.39 (Ph*C*H), 94.73 (C-1), 94.03 (*C*Cl_3_, Troc), 92.08 (C-1′), 81.16 (C-4′), 77.26 (C-4), 77.09 (*C*H_2_, Troc), 72.98 (C-2), 72.91 (C-2′), 71.15 (C-3), 68.83 (C-6′), 68.78 (CH_2_, Alloc), 68.73 (C-3′), 68.26 (C-5), 66.10 (C-6), 63.03 (C-5′), 37.92 (CH_2_, Lev), 29.74 (CH_3_, Lev), 27.91 (CH_2_, Lev), 26.58, 25.91 ([*C*H_3_]_3_C], 2xTBDMS), 18.49, 18.00 ([CH_3_]_3_ *C,* 2xTBDMS), −2.79, −4.00, −4.12, −4.57 (4xCH_3_, 2 x TBDMS); HRMS (^+^ESI) m/z: calcd. for C_43_H_65_Cl_3_KO_17_Si_2_ [M + K]^+^ 1055.2441, found 1055.2443; calcd. for C_43_H_69_Cl_3_NO_17_Si_2_ [M + NH_4_]^+^ 1034.3148, found 1034.3167.

**29:** R_f_ = 0.88 (toluene–EtOAc, 1/1, v/v); R_f_ = 0.49 (toluene–EtOAc, 4/1, v/v); ${\left[\alpha \right]}_{D}^{20}$ = 83 (c = 0.4, CHCl_3_); ^1^H NMR (600 MHz, CDCl_3_): δ [ppm] = 7.50-7.47 (m, 2H, *Ph*CH), 7.38-7.33 (m, 3H, *Ph*CH), 5.92 (m, 1H, C*H*, Alloc), 5.51 (s, 1H, PhCH), 5.38-5.24 (m, 2H, C*H*_2_, Alloc), 5.25 (d, 1H, ^3^ *J*_1′,2′_ = 3.8 Hz, H-1′), 5.17 (d, 1H, ^3^ *J*_1,2_ = 3.7 Hz, H-1), 4.90 (dd, 1H, ^3^ *J*_2′,3′_ = 9.4 Hz, ^3^ *J*_1′,2′_ = 3.8 Hz, H-2′), 4.86-4.62 (m, 2H, C*H*_2_, Troc), 4.83 (t, 1H, ^3^ *J*_3,4_ = ^3^ *J*_4,5_ = 9.7 Hz, H-4), 4.61 (m, 2H, C*H*_2_, Alloc), 4.32 (dd, 1H, ^2^ *J*_6a,6b_ = 12.2 Hz, ^3^ *J*_5,6a_ = 4.3 Hz, H-6a), 4.27 (dd, 1H, ^2^ *J*_6a,6b_ = 12.1 Hz, ^3^ *J*_5,6b_ = 3.1 Hz, H-6b), 4.25 (dd, 1H, ^2^ *J*_6′a,6′b_ = 10.3 Hz, ^3^ *J*_5′,6′a_ = 5.0 Hz, H-6′a), 4.15 (t, 1H, ^3^ *J*_2′,3′_ = ^3^ *J*_3′,4′_ = 9.3 Hz, H-3′), 4.09 (t, 1H, ^3^ *J*_2,3_ = ^3^ *J*_3,4_ = 9.2 Hz, H-3), 4.09-4.04 (m, 2H, H-5, H-5′), 3.71 (t, 1H, ^2^ *J*_6′a,6′b_ = ^3^ *J*_5′,6′b_ = 10.3 Hz, H-6′b), 3.69 (dd, 1H, ^3^ *J*_2,3_ = 9.2 Hz, ^3^ *J*_1,2_ = 3.7 Hz, H-2), 3.53 (t, 1H, ^3^ *J*_3′,4′_ = ^3^ *J*_4′,5′_ = 9.4 Hz, H-4′), 2.86-2.56 (m, 4H, 2xC*H*_2_, Lev), 2.16 (s, 3H, C*H*_3_, Lev), 1.99 (1H, C2-O*H*), 0.90, 0.84 (2×s, 18H, 2×[C*H*_3_]_3_C*,* 2xTBDMS), 0.18, 0.14, 0.08, 0.02 (4×s, 12H, 4×C*H*_3_, 2xTBDMS)); ^13^C NMR (151 MHz, CDCl_3_): δ [ppm] = 205.76 (*C*H_3_C = O, Lev), 172.14 (C=O, Lev), 154.61 (C=O, Alloc), 153.29 (C=O, Troc), 137.09 (*C*_*q*_, Ph), 131.48 (*C*H, Alloc), 128.97, 128.07, 126.24 (CH, Ph), 118.99 (*C*H_2_, Alloc), 101.86 (Ph*C*H), 93.98 (*C*Cl_3_, Troc), 93.86 (C-1), 92.75 (C-1′), 81.84 (C-4′), 77.16 (*C*H_2_, Troc), 76.04 (C-4), 73.62 (C-2′), 72.43 (C-3), 72.20 (C-2), 69.41 (C-3′), 68.70 (CH_2_, Alloc), 68.70 (C-6′), 68.33 (C-5), 65.82 (C-6), 63.14 (C-5′), 37.54 (CH_2_, Lev), 29.71 (CH_3_, Lev), 27.76 (CH_2_, Lev), 25.70, 25.65 ([*C*H_3_]_3_C, 2xTBDMS), 2 × 18.10 ([CH_3_]_3_ *C,* 2xTBDMS), −4.33, −4.36, −4.83, −4.91 (4xCH_3_, 2xTBDMS); HRMS (^+^ESI) m/z: calcd. for C_43_H_65_Cl_3_KO_17_Si_2_ [M + K]^+^ 1055.2441, found 1055.2441; calcd. for C_43_H_69_Cl_3_NO_17_Si_2_ [M + NH_4_]^+^ 1034.3148, found 1034.3154.

**30:** R_f_ = 0.7 (toluene–EtOAc, 3/1, v/v); ${\left[\alpha \right]}_{D}^{20}$ = 68 (c = 1.1, CHCl_3_); ^1^H NMR (600 MHz, CDCl_3_): δ [ppm] = 7.45-7.41 (m, 2H, Ph), 7.36-7.33 (m, 3H, Ph), 5.94 (m, 1H, C*H*, Alloc), 5.48 (s, 1H, PhCH), 5.39-5.25 (m, 2H, C*H*_2_, Alloc), 5.23 (d, 1H, ^3^ *J*_1′,2′_ = 3.7 Hz, H-1′), 5.07 (d, 1H, ^3^ *J*_1,2_ = 3.5 Hz, H-1), 4.92 (dd, 1H, ^3^ *J*_2′,3′_ = 9.4 Hz, ^3^ *J*_1′,2′_ = 3.7 Hz, H-2′), 4.89 (t, 1H, ^3^ *J*_3,4_ = ^3^ *J*_4,5_ = 9.8 Hz, H-4), 4.83-4.76 (m, 2H, C*H*_2_, Troc), 4.63 (m, 2H, C*H*_2_, Alloc), 4.37 (dd, 1H, ^2^ *J*_6a,6b_ = 12.2 Hz, ^3^ *J*_5,6a_ = 4.1 Hz, H-6a), 4.31 (dd, 1H, ^2^ *J*_6a,6b_ = 12.2 Hz, ^3^ *J*_5,6b_ = 2.6 Hz, H-6b), 4.21 (dd, 1H, ^2^ *J*_6′a,6′b_ = 10.3 Hz, ^3^ *J*_5′,6′a_ = 4.8 Hz, H-6′a), 4.17 (t, 2H, ^3^ *J*_2′,3′_ = ^3^ *J*_3′,4′_ = ^3^ *J*_2,3_ = ^3^ *J*_3,4_ = 9.3 Hz, H-3′, H-3), 4.09 (m, 1H, ^3^ *J*_4,5_ = ^3^ *J*_5_,_6b_ = 10.1 Hz, ^3^ *J*_5,6a_ = 3.3 Hz, H-5), 4.02 (td, 1H, ^3^ *J*_4′,5′_ = ^3^ *J*_5′,6′b_ = 10.0 Hz, ^3^ *J*_5′,6′a_ = 4.8 Hz, H-5′), 3.74 (dd, 1H, ^3^ *J*_2,3_ = 9.4 Hz, ^3^ *J*_1,2_ = 3.5 Hz, H-2), 3.71 (t, 1H, ^2^ *J*_6′a,6′b_ = ^3^ *J*_5′,6′b_ = 10.4 Hz, H-6′b), 3.50 (t, 1H, ^3^ *J*_3′,4′_ = ^3^ *J*_4′,5′_ = 9.4 Hz, H-4′), 2.87-2.52 (m, 4H, 2xC*H*_2_, Lev), 2.17 (s, 3H, C*H*_3_, Lev), 2.23 (s, 1H, C3-O*H*), 0.96, 0.80 (2×s, 18H, 2×[C*H*_3_]_3_C*,* 2xTBDMS), 0.17, 0.14, 0.04, −0.05 (4×s, 12H, 4×C*H*_3_, 2xTBDMS); ^13^C NMR (151 MHz, CDCl_3_): δ [ppm] = 205.74 (*C*H_3_C = O, Lev), 172.14 (C=O, Lev), 154.70 (C=O, Alloc), 153.69 (C=O, Troc), 137.15 (*C*_*q*_, Ph), 131.52 (*C*H, Alloc), 129.03, 128.04, 126.46 (CH, Ph), 119.00 (*C*H_2_, Alloc), 102.35 (Ph*C*H), 94.29 (*C*Cl_3_, Troc), 93.28 (C-1), 91.99 (C-1′), 82.11 (C-4′), 77.06 (*C*H_2_, Troc), 75.18 (C-4), 73.54 (C-2′), 72.57 (C-2), 71.56 (C-3), 69.31 (C-3′), 68.82 (C-6′), 68.76 (CH_2_, Alloc), 67.78 (C-5), 65.66 (C-6), 62.99 (C-5′), 37.59 (CH_2_, Lev), 29.75 (CH_3_, Lev), 27.85 (CH_2_, Lev), 25.90, 25.67 ([*C*H_3_]_3_C, 2xTBDMS), 18.14, 18.10 ([CH_3_]_3_ *C,* 2xTBDMS), −3.96, −4.32, −4.85, −4.93 (4xCH_3_, 2xTBDMS); HRMS (^+^ESI) m/z: calcd. for C_43_H_65_Cl_3_KO_17_Si_2_ [M + K]^+^ 1055.2441, found 1055.2441; calcd. for C_43_H_69_Cl_3_NO_17_Si_2_ [M + NH_4_]^+^ 1034.3148, found 1034.3154.

## Data Availability

The original contributions presented in the study are included in the article/Supplementary Material, further inquiries can be directed to the corresponding author.
