# Supplementary material for: Versatile approach towards fully desymmetrized trehalose with a novel set of orthogonal protecting groups
Source: Front Chem. 2024 Jan 11;11:1332837. doi: 10.3389/fchem.2023.1332837 (PMC10808579; doi:10.3389/fchem.2023.1332837)

Supplementary Material

Versatile approach towards fully desymmetrized trehalose with a novel set of orthogonal protecting groups

Tomáš Vašíček, Benjamin Arensmeyer, Alessandro Monti, Alla Zamyatina*

Content

Synthesis of (1) and (2): alternative preparation schemes and purification protocols 2

Experimental procedures 4

Optimisation of reaction conditions for regioselective cleavage of TBDMS groups in (22) and (28) 12

^{1}H-, ^{13}C-NMR spectra of synthetic compounds 18

Synthesis of (1) and (2): alternative preparation schemes and purification approaches.

The reaction of trehalose with benzaldehyde dimethylacetate (DMT) under acidic catalysis generally results in the formation of symmetrically substituted *bis*-4,6-*O*-benzilydene acetal **S1**, while a monoprotected **1** is usually obtained in minor amounts as a by-product. The synthesis of trehalose 4,6-*O*-benzylidene acetal **1** starting from trehalose has been reported previously,[(1)] albeit in low yield and without indication of the purity and spectroscopic data of the isolated material. In accordance with numerous reported synthetic procedures, the camphor sulfonic acid (CSA) - promoted reaction of trehalose with DMT afforded in our hands the symmetrically substituted **S1** as the major product. Careful optimisation of the reaction conditions allowed for predominant formation of **1** (55-65%) together with **S1** (10%), while unreacted trehalose (25-35%) was still present in the mixture (when the reaction was terminated before complete conversion). After removal of the reaction solvent (DMF), the reaction mixture formed a poorly soluble polymer-like jelly, so that the isolation of the individual products by column chromatography was highly inefficient due to the very different polarities and solubilities of the constituents. Therefore, the polar, water-soluble components [**Tre** + **1**] were first separated from **S1** by partitioning the reaction mixture between EtOAc and water, providing [**Tre** + **1**] in the aqueous phase (90%) and **S1** in the organic phase (10%). The pH of the aqueous phase containing **Tre** and **1** had to be continuously adjusted by the addition of an inorganic, water-soluble base to prevent the loss of the benzylidene acetal group due to the presence of residual CSA. Although the CSA was neutralised by the addition of Et_3N to terminate the reaction, the latter leached into the organic phase during the extraction, rendering the aqueous phase highly acidic and leading to hydrolysis of benzylidene acetal group in **1**. Therefore, in the next step, the remaining CSA was removed by passing the aqueous solution of [**Tre** + **1**] through an anion exchange resin column (OH^--form) which afforded **1** in a mixture with unreacted trehalose (SI-Scheme 1).

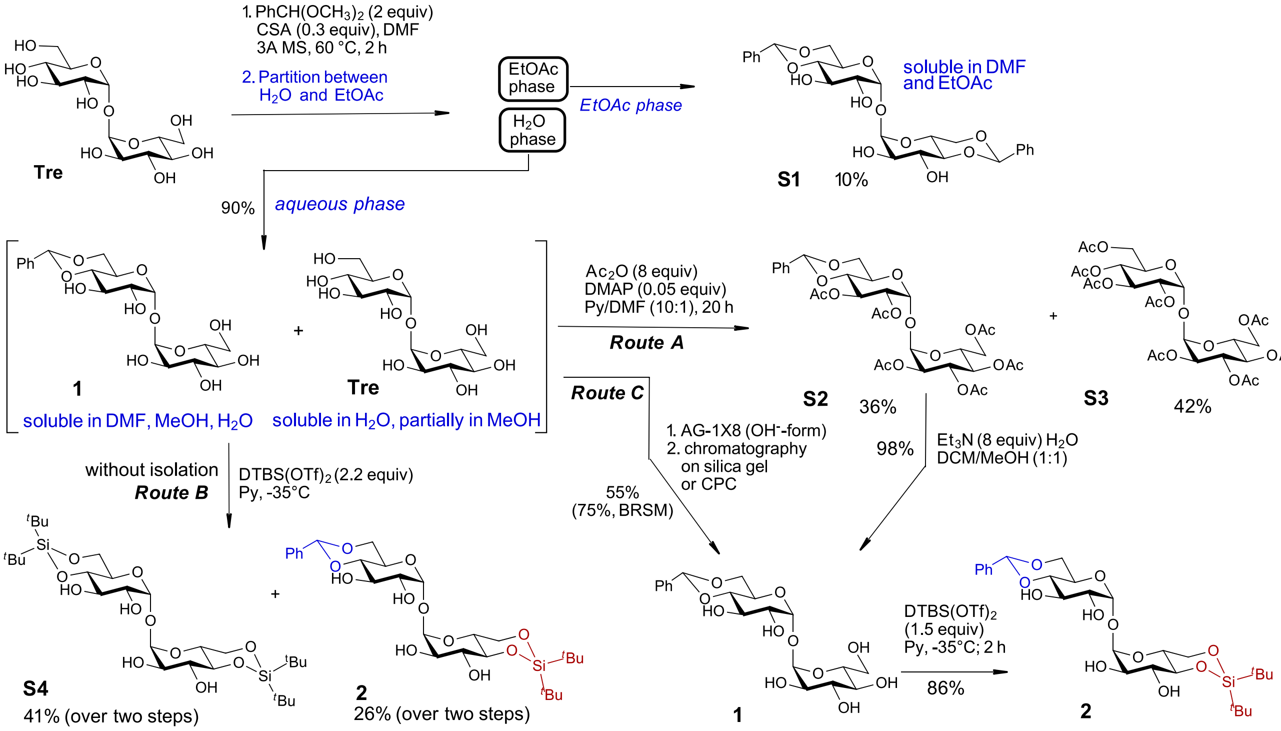

SI-Scheme 1. Synthesis of 4,6-O-monobenzylidene protected trehalose **1** and desymmetrised trehalose derivative **2**.

Considering the use of preparative quantities of materials (10 g scale), the high polarity and poor solubility of the amphiphilic **1**, we sought an efficient way to either proceed to DTBS-protected **2** using a mixture [**Tre**+**1**] or to separate **1** in pure form at low cost. Accordingly, the mixture [**Tre**+**1**] was peracetylated to give **S2** and **S3**[(2,3)], and these less polar products were readily separated by conventional silica gel chromatography yielding the target **S2** in only 36% yield (SI-Scheme 1, *Route A*). Alternatively, the mixture [**Tre**+**1**] was directly reacted with DTBS(OTf)$_2$ in pyridine to give a symmetrical *bis*-DTBS-protected derivative **S4** and the target non-symmetrically protected **2** which was readily isolated, albeit in an unsatisfactory yield of 26% (SI-Scheme 1, *Route B*). Initial attempts to separate the amphiphilic **1** from the unprotected trehalose using silica gel chromatography (EtOAc/MeOH, 10:1→10:3) resulted in low yields and incomplete separation. Switching to a classical solvent mixture for lipid purification (DCM/MeOH/aq. NH_3, 6/4/1) allowed for a clean isolation of **1** in 52% yield and a recovery of trehalose.

An even more facile isolation of **1** was achieved by using centrifugal partition chromatography (CPC). CPC, which is based on the differential partitioning of analytes between two immiscible liquid phases, is a preparative, support-free liquid-liquid chromatography technique. One of the liquid phases is held stationary in the column (the rotor) by a constant field of centrifugal force, while the other liquid phase is pumped through the column. CPC offers many advantages over conventional chromatography in that there is no solid support, which is replaced by a large volume of liquid stationary phase, and is characterized by high capacity, low solvent consumption and total recovery of the loaded sample. The biphasic liquid systems used in CPC usually consist of a mixture of three to four solvents and/or solutions: two immiscible solvents and up to two modifying solvents that can be mixed with both. The two phases of the same solvent system are in thermodynamic equilibrium in a rotating column and the analytes must be soluble in both phases obtained after equilibration of the biphasic liquid system. The separation of [**Tre+1**] by CPC was performed in 2 g portions in descending mode using ethyl acetate and water as two immiscible solvents and methanol as modifier (EA/MeOH/H_2O (20/7/20, 800 mL), resulting in a clean isolation of **1** (53%) and full recovery of unreacted trehalose thus providing **2** in 75% yield (BRSM). Next, **1** was subjected to a reaction with DTBS(OTf)$_2$ in pyridine at -40° C leading to the formation of 4,6-*O*-di-*tert*-butylsilylene (DTBS) protected **2** in 86% yield (SI-Scheme 1; *Route C*). The synthetic steps [**Tre→1→2**] were performed in a multigram scale (starting from 10 g of trehalose) and carefully optimised to achieve the highest reproducibility.

References.

1. Cheng, K. L. (1976) Synthesis of α-D-glucopyranosyl α-D-galoctopyranoside. *Carbohydr. Res. 50,* 152-157.

2. Hsieh, H. W., Schombs, M. W., Witschi, M. A., and Gervay-Hague, J. (2013) Regioselective Silyl/Acetate Exchange of Disaccharides Yields Advanced Glycosyl Donor and Acceptor Precursors, In *J. Org. Chem.*, pp 9677-9688.

3. Färnbäck, M., Eriksson, L., and Widmalm, G. (2004) Octa-*O*-acetyl-α,α-trehalose ethanol disolvate. *Acta Crystallogr. Sect. E 60*, 1483-1485.

Experimental procedures

General methods:

Reagents and solvents were purchased from commercial suppliers and used without further purification unless otherwise stated. Dichloromethane was distilled from CaH_2 and stored over activated molecular sieves (4Å). Other solvents were dried by storage over activated molecular sieves for at least 48 h prior to use [3Å for DMF]. Residual moisture was determined by colorimetric titration on a Mitsubishi CA-21 Karl Fischer apparatus and did not exceed 10 ppm for dry solvents. All reactions were monitored by TLC performed on silica gel 60 F254 HPTLC pre-coated glass plates with a 25 mm concentration zone (Merck). Spots were visualized by UV-light followed by dipping into H_2SO_4-*p*-anisaldehyde solution and subsequent charring at 250°C. Organic solvents were removed under reduced pressure at 30°C. NMR spectra were recorded at 298 K on a Bruker Avance III 600 spectrometer (^{1}H at 600.22 MHz; ^{13}C at 150.92 MHz) and/or on Bruker AC 300 spectrometer (^{1}H at 300.13 MHz; ^{13}C at 75.49 MHz) using standard Bruker NMR software. Chemical shifts are reported in ppm. ^{1}H-NMR, ^{13}C-NMR are referenced to residual solvent signals (for MeOD: 3.31 ppm, for $CDCl_3$: 7.26 ppm). The Glc-ring with benzylidene acetal protecting group (the upper left ring in the structures) is indicated by prime. High-resolution mass spectrometry (HRMS) was carried out from 1–10 mg/L acetonitrile solutions via LC-TOF MS (Agilent 1200SL HPLC and Agilent 6210 ESI–TOF, Agilent Technologies). The mass spectrometer was tuned with Agilent Mass Hunter Software. Optical rotation was measured on a Perkin Elmer 243 B polarimeter, equipped with a Haake water circulation bath and a Haake D1 immersion circulator for temperature control of the measuring cell. $[\alpha]_D^{20}$ values are given in units of $deg \cdot dm^{-1} \cdot cm^3 \cdot g^{-1}$.

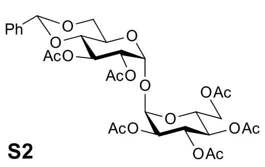

S2

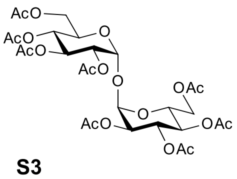

S3

Route A

4,6-*O*-Benzylidene-2,3-di-*O*-acetyl-α-D-glucopyranosyl-(1↔1)-2,3,4,6-tetra-*O*-acetyl-α-D-glucopyranoside (S2) and **2,3,4,6-tetra-*O*-acetyl-α-D-glucopyranosyl-(1↔1)-2,3,4,6-tetra-*O*-acetyl-α-D-glucopyranoside (S3)**

Trehalose dihydrate (3.30 g, 8.723 mmol) was dissolved in dry DMF (87 mL) and stirred with freshly dried powdered molecular sieves (3Å) for 1 h at r.t. Camphor-10-sulfonic acid (CSA; 0.608 g, 2.62 mmol) and benzaldehyde dimethyl acetal (DMT; 2.62 mL, 17.4 mmol) we added succesively and the reaction mixture was stirred at 60° C for 2 h. The reaction was quenched by dropwise addition of Et_3N (0.3 mL). Solids were removed by filtration over a pad of Celite, and the filtrate was concentrtaed. The residue was partitined between H_2O/EA (3/1, 200 mL) and the organic phases was washed with H_2O (3 x 50 mL), the aqueous phases were combined and reextracted with EA (2 x 50 mL). The combined aqueous phases were concentrated. The residue was dissolved in dry DMF (100 mL), and pyridine (6.67 mL, 82.8 mmol), DMAP (0.060 g, 0.52 mmol) and Ac_2O (7.8 mL, 82.8 mmol) were added successively and the reaction mixture was stirred at r.t. for 20 h. MeOH (15 mL) was added and the reaction mixture was stirred for 15 min. at r.t. and concentrated. The residue was purified

by column chromatography on silica gel (toluene − EtOAc, 1/1) to afford **S2** (2.396 g, 36%) and **S3**[(2,3)] (2.75 g, 42%) as solids.

S2: R_f = 0.48 (toluene – EtOAc, 1/1, v/v); $[\alpha]_D^{20}$ = 145 (c = 0.5, $CHCl_3$); ^{1}H NMR (600 MHz, $CDCl_3$): δ [ppm] = 7.44-7.41 (m, 2H, *Ph*CH), 7.38-7.34 (m, 3H, *Ph*CH), 5.60 (t, 1H, $^3J_{3',2'}$ = $^3J_{3',4'}$ = 9.8 Hz, H-3′), 5.50 (dd, 1H, $^3J_{3,2}$ = 10.1 Hz, $^3J_{3,4}$ = 9.5 Hz, H-3), 5.49 (s, 1H, PhC*H*), 5.37 (d, 1H, $^3J_{1,2}$ = 4.0 Hz, H-1), 5.27 (d, 1H, $^3J_{1',2'}$ = 4.0 Hz, H-1′), 5.05 (dd, 1H, $^3J_{4,5}$ = 10.4 Hz, $^3J_{4,3}$ = 9.5 Hz, H-4), 5.05 (dd, 1H, $^3J_{2',3'}$ = 9.9 Hz, $^3J_{2',1'}$ = 4.0 Hz, H-2′), 5.00 (dd, 1H, $^3J_{2,3}$ = 10.3 Hz, $^3J_{2,1}$ = 4.0 Hz, H-2), 4.25 (dd, 1H, $^2J_{6a,6b}$ = 12.2 Hz, $^3J_{6a,5}$ = 5.6 Hz, H-6a), 4.17 (dd, 1H, $^2J_{6'a,6'b}$ = 10.5 Hz, $^3J_{6'\alpha,5}$ = 4.9 Hz, H-6′a), 4.09 (ddd, 1H, $^3J_{5,4}$ = 10.3 Hz, $^3J_{5,6a}$ = 5.6 Hz, $^3J_{5,6b}$ = 2.1 Hz, H-5), 4.01 (dd, 1H, $^2J_{6b,6a}$ = 12.2 Hz, $^3J_{6b,5}$ = 2.2 Hz, H-6b), 3.97 (td, 1H, $^3J_{5',4'}$ = $^3J_{5',6'b}$ = 14.9 Hz, $^3J_{5',6'a}$ = 4.9 Hz, H-5′), 3.75 (t, 1H, $^2J_{6'b,6'a}$ = $^3J_{6'b,5'}$ = 10.5 Hz, H-6′b), 3.68 (t, 1H, $^3J_{4',3'}$ = $^3J_{4',5'}$ = 9.7 Hz , H-4′), 2.12, 2.10, 2.08, 2.08, 2.05, 2.03 (6 x s, 18H, C*H*$_3$CO(Ac)); ^{13}C NMR (151 MHz, $CDCl_3$): δ [ppm] = 170.57, 170.03, 169.85, 169.76, 169.60, 169.57 (CH_3*C*O, Ac), 136.72 (C_q, Ph), 129.13, 128.22, 126.16 (CH, Ph), 101.81 (Ph*C*H), 93.32 (C-1′), 92.18 (C-1), 79.01 (C-4′), 70.64 (C-2′), 70.10 (C-2), 70.02 (C-3), 68.93 (C-3′), 68.56 (C-4), 68.56 (C-6′), 68.13 (C-5), 63.18 (C-5′), 61.79 (C-6), 20.82, 20.64, 20.59 (*C*H$_3$CO).

To a stirred solution of **S2** (0.875 g, 1.282 mmol) in DCM/MeOH (5 mL, 1/1), Et_3N (4.5 mL, 32.05 mmol) and H_2O (2.8 mL, 153.82 mmol) were added. The reaction mixture was stirred for 24 h at r.t., diluted with toluene (20mL) and concentrated to dryness. The residue was dissolved in H_2O (100 mL), extracted with EtOAc (2 x 10 mL) and the aqueous phase was concentrated to afford **1** (0.540 g, 98%).

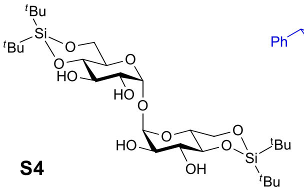

S4

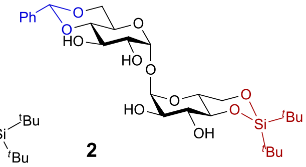

2

Route B

4,6-*O*-Benzylidene-α-D-glucopyranosyl-(1↔1)-4,6-*O*-di-*tert*-butylsilylene-α-D-glucopyranoside (**2**) and **4,6-*O*-di-*tert*-butylsilylene-α-D-glucopyranosyl-(1↔1)-4,6-*O*-di-*tert*-butylsilylene-α-D-glucopyranoside** (**S4**)

Trehalose dihydrate (3.117 g, 8.239 mmol) was dissolved in dry DMF (80 mL) and stirred for 2 h with powdered molecular sieves (3Å) at 60° C. Benzaldehyde dimethyl acetal (2.45 mL, 16.3 mmol) and camphor-10-sulfonic acid (0.575 g, 2.45 mmol) were added successively, and the reaction mixture was stirred at 60° C for 2 h. The reaction was quenched by addition of Et_3N (0.2 mL), the solids were removed by filtration over a pad of Celite, and the filtrate was concentrated. The residue was partitioned between H_2O/EA (60 mL, 3/1), the phases were separated and the organic phases was washed with H_2O (3 x 40 mL). The combined aqueous phases were reextracted with EtOAc (2 x 50 mL), and concentrated. The residue was dissolved in dry pyridine (80 mL) and powdered molecular sieves (3Å) were added. The mixture was brought to -35° C and trifluoromethanesulfonic acid di-*tert*-butylsilylene ester DTBS(OTf)$_2$ (5.56 mL, 17.12 mmol) was added dropwise under atmosphere of Ar. Reaction mixture was stirred for 30 min at -35° C under atmosphere of Ar, then a solution of triethylamine (3.2 mL) in MeOH (6 mL) was added dropwise and the stirring was continued for 10 min. The solids were

removed by filtration over a pad of Celite and the filtrate was concentrated. The residue was dissolved in EtOAc (300 mL) and washed with sat. aq. $NaHCO_3$ (2 x 100 mL) and brine (50 mL), dried over $MgSO_4$, filtered and concentrated. The residue was purified by column chromatography on silica gel (toluene – EtOAc, 1/1 → 2/8) to afford **2** (1.30 g, 26%) and **S4** (2.32 g, 41%) as solids.

S4: R_f = 0.44 (Tol – EtOAc, 1/1, v/v); $[\alpha]_D^{20}$ = 86 (c = 1.0, $CHCl_3$); ^{1}H NMR (600 MHz, $CDCl_3$): δ [ppm] = 5.14 (d, 2H, $^3J_{1,2}$ = 3.8 Hz, 2xH-1), 4.11 (dd, 2H, $^2J_{6a,6b}$ = 9.8 Hz, $^3J_{6a,5}$ = 5.0 Hz, H-6a), 4.01 (td, 2H, $^3J_{5,4}$ = $^3J_{5,6b}$ = 10.0 Hz, $^3J_{5,6a}$ = 5.0 Hz, H-5), 3.83 (t, 4H, $^3J_{3,2}$ = $^3J_{3,4}$ = $^3J_{6ax,5}$ = $^3J_{6b,6a}$ = 9.9 Hz, H-3, H-6b), 3.71 (dd, 2H, $^3J_{2,3}$ = 9.5 Hz, $^3J_{2,1}$ = 3.8 Hz, H-2), 3.66 (t, 2H, $^3J_{4,3}$ = $^3J_{4,5}$ = 9.2 Hz, H-4), 1.05, 0.98 (2xs, 36H, 4x[C*H*$_3$]$_3$C], DTBS); ^{13}C NMR (151 MHz, $CDCl_3$): δ [ppm] = 93.38 (C-1), 77.14 (C-4), 74.31 (C-3), 71.60 (C-2), 66.68 (C-5), 66.43 (C-6), 27.38, 26.95 ([*C*H$_3$]$_3$C], DTBS), 22.65, 20.00 ([CH$_3$]$_3$*C*, DTBS).

Route C for the synthesis of 2 is included in the manuscript.

Synthesis of **6** and **7**

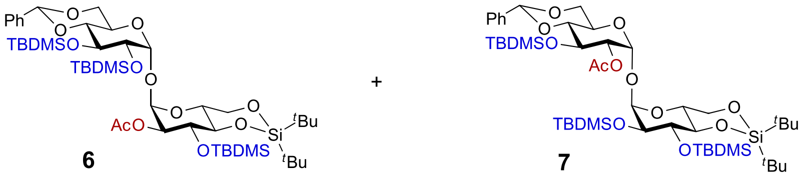

6 + **7**

4,6-*O*-Benzylidene-2,3-di-*O*-*tert*-butyldimethylsilyl-α-D-glucopyranosyl-(1↔1)-2-*O*-acetyl-3-*O*-*tert*-butyldimethylsilyl-4,6-*O*-di-*tert*-butylsilylene-α-D-glucopyranoside 6) and

4,6-*O*-Benzylidene-2-*O*-acetyl-3-*O*-*tert*-butyldimethylsilyl-α-D-glucopyranosyl-(1↔1)-2,3-di-*O*-*tert*-butyldimethylsilyl-4,6-*O*-di-*tert*-butylsilylene-α-D-glucopyranoside (7)

To a stirred solution of [**4 + 5**] (0.10 g, 0.109 mmol) in dry CH_2Cl_2 (500 µL) acetic anhydride (16 µL, 0.164 mmol) and 4-dimethylaminopyridine (20 mg, 0.164 mmol) were added successively under atmosphere of Ar. The reaction mixture was stirred for 3 h at r.t., then diluted with EtOAc (30 mL) and washed with aq. satd. $NaHCO_3$ (2 × 10 mL) aq. satd. $CuSO_4$ (2 × 10 mL) and brine (2 × 10 mL). The organic layer was dried over Na_2SO_4, filtered and concentrated. The residue was purified by column chromatography on silica gel (toluene − EtOAc, 100/0 → 97/3) to afford [**6 + 7**] as a mixture (0.10 g, 99%). R_f = 0.43, 0.38 (toluene – EtOAc, 95/5, v/v); R_f = 0.67, 0.62 (hexane – EtOAc, 80/20, v/v);

^{1}H NMR (the chemical shifts for compound **6** are with superscript ^{m}) (600 MHz, $CDCl_3$): δ [ppm] = 7.47-7.32 (m, *Ph*CH), 5.50, 5.43 (2xs, PhC*H*), 5.18 (d, 1H, $^3J_{1,2}$ = 3.4 Hz, H-1)^m, 5.18 (d, $^3J_{1',2'}$ = 3.6 Hz, H-1′), 5.02 (d, 1H, $^3J_{1',2'}$ = 2.9 Hz, H-1′)^m, 4.94 (d, $^3J_{1,2}$ = 3.1 Hz, H-1), 4.88 (dd, $^3J_{1',2'}$ = 3.6 Hz, $^3J_{2',3'}$ = 9.6 Hz, H-2′), 4.80 (dd, 1H, $^3J_{1,2}$ = 3.6 Hz, $^3J_{2,3}$ = 9.8 Hz, H-2)^m, 4.28-3.37 (4xH-3, 4xH-4, 4xH-5, 4xH-6), 3.66 (dd, $^3J_{1',2'}$ = 2.9 Hz, $^3J_{2',3'}$ = 9.1 Hz, H-2′)^m, 3.57 (dd, $^3J_{1',2'}$ = 3.1 Hz, $^3J_{2',3'}$ = 9.1 Hz, H-2′), 2.18, 2.13 (s, 3H, CH_3, Ac), 1.07, 1.07, 1.02, 0.97, 0.96, 0.94, 0.92 0.81, 0.81 ([C*H*$_3$]$_3$C, 3xTBDMS, 1xDTBS), 0.18, 0.18, 0.17, 0.17, 0.16, 0.15, 0.13, 0.03, 0.02, 0.02 (12×C*H*$_3$, 6xTBDMS); ^{13}C NMR (151 MHz, $CDCl_3$): δ [ppm] = 170.31, 170.23^m (C=O, Ac), 137.32

(C_q, Ph), 129.08^{m}, 128.97, 128.09^{m}, 128.05, 126.49, 126.44^{m} (PhCH), 102.66^{m}, 102.29 (Ph*C*H), 98.01^{m}, 97.20, 94.40, 93.73^{m} (4xC-1), 82.60^{m}, 82.42, 78.44, 78.05^{m}, 74.29^{m}, 73.85, 73.71, 73.48^{m}, 71.48, 71.20, 69.10, 68.09, 67.62, 64.09, 63.40 (4xC-2, 4xC-3, 4xC-4, 4xC-5), 69.12^{m}, 69.06, 66.88, 66.77^{m} (4xC-6), 27.90, 27.64, 27.23, 26.73, 26.64, 26.40, 26.14, 25.77, 25.64 (10x[CH_3]$_3$C], 6xTBDMS, 2xDTBS), 21.11^{m}, 21.05 (CH_3, Ac), 22.91, 22.84, 21.13, 19.82, 18.84, 18.70, 18.39, 18.16, 18.11 ([CH_3]$_3$*C*, 6xTBDMS, 2xDTBS), -2.71, -3.18, -3.34, -3.87, -4.04, -4.40, -4.49, -4.67, -4.77, -5.03 (CH_3, 6xTBDMS); HRMS ($^+$ESI) m/z: calcd for $C_{47}H_{87}O_{12}Si_4$ $[M+H]^+$ 955.5269, found 955.5263; calcd for $C_{47}H_{90}NO_{12}Si_4$ $[M+NH_4]^+$ 972.5535, found 972.5536.

Synthesis of **10** and **11**

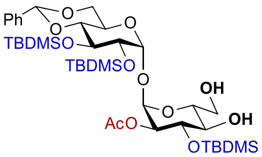

10

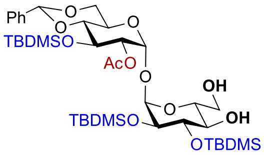

11

4,6-*O*-Benzylidene-2,3-di-*O*-*tert*-butyldimethylsilyl-α-D-glucopyranosyl-(1↔1)-2-*O*-acetyl-3-*O*-*tert*-butyldimethylsilyl-α-D-glucopyranoside (10) and

4,6-*O*-Benzylidene-2-*O*-acetyl-3-*O*-*tert*-butyldimethylsilyl-α-D-glucopyranosyl-(1↔1)-2,3-di-*O*-*tert*-butyldimethylsilyl-α-D-glucopyranoside (**11**)

To a stirred solution of [**6** + **7**] (0.820 g, 0.859 mmol) in dry THF (10 mL) placed in a PTFE reaction vessel a solution of HF·Py (200 µL, 20µL HF·Py/1 mL THF = 0.15 M) was added at 0° C. The reaction mixture was stirred for 16 h at r.t., diluted with EtOAc (150 mL), washed with aq. satd. $NaHCO_3$ (3 × 50 mL), aq. satd. $CaCl_2$ (2 × 30 mL) and brine (2 × 30 mL). The organic layer was dried over Na_2SO_4, filtered and concentrated. The residue was purified by column chromatography on silica gel (toluene − EtOAc, 98/2 → 75/25) to afford **10** (0.467 g, 71%) and **11** (0.153g, 21%) as solids.

10: R_f = 0.28 (toluene – EtOAc, 8/2, v/v); $[\alpha]_D^{20}$ = 85 (c = 1.5, $CHCl_3$); ^{1}H NMR (600 MHz, $CDCl_3$): δ [ppm] = 7.41-7.38 (m, 2H, *Ph*CH), 7.36-7.33 (m, 3H, *Ph*CH), 5.42 (s, 1H, PhC*H*), 5.31 (d, 1H, $^3J_{1,2}$ = 3.4 Hz, H-1), 5.01 (d, 1H, $^3J_{1',2'}$ = 3.1 Hz, H-1′), 4.72 (dd, 1H, $^3J_{2,3}$ = 9.9 Hz, $^3J_{1,2}$ = 3.5 Hz, H-2), 4.13 (t, 1H, $^3J_{2,3}$ = $^3J_{3,4}$ = 9.3 Hz, H-3), 4.13 (t, 1H, $^2J_{6'a,6'b}$ = $^3J_{6'a,5'}$ = 9.1 Hz, H-6′a), 3.95 (dt, 1H, $^3J_{5,4}$ = $^3J_{5,6b}$ = 9.8 Hz, $^3J_{5,6a}$ = 3.6 Hz, H-5), 3.82 (d, 2H, $^3J_{5,6a}$ = 3.6 Hz, H-6a,b), 3.72 (td, 1H, $^3J_{5',4'}$ = $^3J_{6'b,5'}$ = 15.0 Hz, $^3J_{5',6'a}$ = 4.6 Hz, H-5′), 3.67 (t, 1H, $^2J_{6'a,6'b}$ = $^3J_{5',6'b}$ = 10.4 Hz, H-6′b), 3.66 (t, 1H, $^3J_{3,4}$ = $^3J_{4,5}$ = 9.2 Hz, H-4), 3.65 (dd, 1H, $^3J_{3',2'}$ = 8.9 Hz, $^3J_{1',2'}$ = 3.1 Hz, H-2′), 3.41 (t, 1H, $^3J_{3',4'}$ = 9.3 = $^3J_{4',5'}$ = Hz, H-4′), 2.20 (s, 3H, CH_3, Ac), 0.94, 0.92, 0.81 (3×s, 27H, 3×[C*H*$_3$]$_3$C, 3xTBDMS), 0.17, 0.16, 0.13, 0.11, 0.02, 0.01 (6×s, 18H, 6×C*H*$_3$, 3xTBDMS); ^{13}C NMR (151 MHz, $CDCl_3$): δ [ppm] = 170.31 (C=O, Ac), 137.23 (C_q, Ph), 129.12, 128.10, 126.47 (CH, Ph), 102.68 (Ph*C*H), 95.43 (C-1), 91.16 (C-1′), 82.41 (C-4′), 73.96 (C-2′), 73.31 (C-2), 72.06 (C-3′), 71.51 (C-3), 71.50 (C-4), 71.49 (C-5), 68.90 (C-6′), 63.74 (C-5′), 62.16 (C-6), 26.42, 26.19, 25.76 (3x[*C*H$_3$]$_3$C], 3xTBDMS), 21.04 (*C*H$_3$, Ac), 18.48, 18.22,

18.15 (3x[CH_3]$_3$*C*, 3xTBDMS), -3.06, -4.01, -4.39 (3x*C*H_3, 3xTBDMS); HRMS ($^+$ESI) m/z: calcd. for $C_{39}H_{71}O_{12}Si_3$ [M+H]$^+$ 815.4248, found 815.4238; calcd. for $C_{39}H_{74}O_{12}Si_3$ [M+NH_4]$^+$ 832.4513, found 832.4516.

11: R_f = 0.17 (toluene – EtOAc, 8/2, v/v); $[\alpha]_D^{20}$ = 37 (c = 0.1, $CHCl_3$); ^{1}H NMR (600 MHz, $CDCl_3$): δ [ppm] = 7.47-7.43 (m, 2H, *Ph*CH), 7.38-7.34 (m, 3H, *Ph*CH), 5.50 (s, 1H, PhC*H*), 5.29 (d, 1H, $^3J_{1',2'}$ = 3.6 Hz, H-1′), 5.05 (d, 1H, $^3J_{1,2}$ = 2.8 Hz, H-1), 4.85 (dd, 1H, $^3J_{2',3'}$ = 9.5 Hz, $^3J_{1',2'}$ = 3.7 Hz, H-2′), 4.22 (t, 1H, $^3J_{2',3'}$ = $^3J_{3',4'}$ = 9.2 Hz, H-3′), 4.22 (t, 1H, $^2J_{6'a,6'b}$ = 10.4 Hz, $^3J_{5',6'a}$ = 5.2 Hz, H-6′a), 4.04 (m, 1H, H-5′), 4.03 (t, 1H, $^3J_{2,3}$ = $^3J_{3,4}$ = 8.3 Hz, H-3), 3.77 (d, 2H, $^3J_{5,6a}$ = 4.4 Hz, H-6a,b), 3.72 (t, 1H, $^2J_{6'a,6'b}$ = $^3J_{5',6'b}$ = 10.4 Hz, H-6′b), 3.62 (m, 1H, H-5), 3.60 (dd, 1H, $^3J_{2,3}$ = 8.9 Hz, $^3J_{1,2}$ = 2.8 Hz, H-2), 3.52 (t, 1H, $^3J_{3',4'}$ = $^3J_{4',5'}$ = 9.4 Hz, H-4′), 3.48 (t, $^3J_{3,4}$ = $^3J_{4,5}$ = 8.6 Hz, H-4), 2.11 (s, 3H, CH_3, Ac), 0.97, 0.95, 0.82 (3×s, 27H, 3×[C*H*$_3$]$_3$C, 3xTBDMS), 0.18, 0.17, 0.15, 0.14, 0.04, -0.02 (6×s, 18H, 6×C*H*$_3$, 3xTBDMS); ^{13}C NMR (151 MHz, $CDCl_3$): δ [ppm] = 170.40 (C=O, Ac), 137.31 (C_q, Ph), 128.97, 128.05, 126.40 (Ph*C*H), 102.19 (Ph*C*H), 94.47 (C-1), 91.91 (C-1′), 82.01 (C-4′), 74.02 (C-3), 73.77 (C-2′), 72.97 (C-5), 72.94 (C-2), 71.86 (C-4), 69.25 (C-3′), 68.90 (C-6′), 63.41 (C-5′), 62.41 (C-6), 26.53, 26.21, 25.63 (3x[*C*H$_3$]$_3$C], 3xTBDMS), 20.91 (*C*H$_3$, Ac), 18.42, 18.41, 18.16 (3x[CH_3]$_3$*C*, 3xTBDMS), -3.19, -3.93, -4.20, -4.32, -4.42, -5.01 (6x*C*H$_3$, 3xTBDMS); HRMS ($^+$ESI) m/z: calcd. for $C_{39}H_{74}O_{12}Si_3$ [M+NH_4]$^+$ 832.4513, found 832.4526.

Synthesis of **14**

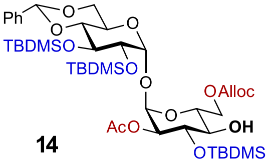

14

4,6-*O*-Benzylidene-2,3-di-*O*-*tert*-butyldimethylsilyl-α-D-glucopyranosyl-(1↔1)-2-*O*-acetyl-6-*O*-allyloxycarbonyl-3-*O*-*tert*-butyldimethylsilyl-α-D-glucopyranoside (14)

To a stirred solution of **10** (0.475 g, 0.528 mmol) in dry CH_2Cl_2 (15 mL) collidine (1.10 mL, 5.28 mmol) and allyloxycarbonyl chloride AllocCl (890 µL, 5.28 mmol) were added successively at 0° C. The reaction mixture was brought up to r.t. and stirred for 18 h. The reaction mixture was concentrated, the residue was dissolved in EtOAc (150 mL) and washed with aq. satd. $NaHCO_3$ (4 × 40 mL), aq. satd. $CuSO_4$ (4 × 25 mL) and brine (2 × 20 mL). The organic layer was dried over Na_2SO_4, filtered and concentrated. The residue was purified by column chromatography on silica gel (toluene − EtOAc, 90/10 → 80/20) to afford **14** (0.402 g, 76%) as a syrup. R_f = 0.73 (toluene – EtOAc, 8/2, v/v); R_f = 0.36 (toluene – EtOAc, 9/1, v/v); $[\alpha]_D^{20}$ = 5.0 (c = 0.2, $CHCl_3$); ^{1}H NMR (600 MHz, $CDCl_3$): δ [ppm] = 7.41-7.38 (m, 2H, *Ph*CH), 7.36-7.33 (m, 3H, *Ph*CH), 5.94 (m, 1H, C*H*, Alloc), 5.41 (s, 1H, PhC*H*), 5.40-5.27 (m, 2H, C*H*$_2$, Alloc), 5.30 (d, 1H, $^3J_{1,2}$ = 3.4 Hz, H-1), 5.02 (d, 1H, $^3J_{1',2'}$ = 3.1 Hz, H-1′), 4.77 (dd, 1H, $^3J_{2,3}$ = 9.9 Hz, $^3J_{1,2}$ = 3.5 Hz, H-2), 4.64 (dt, 2H, C*H*$_2$, Alloc), 4.47 (dd, 1H, $^2J_{6a,\,6b}$ = 11.9 Hz, $^3J_{6a,\,5}$ = 6.0 Hz, H-6a), 4.34 (dd, 1H, $^2J_{6a,\,6b}$ = 11.9 Hz, $^3J_{6b,\,5}$ = 2.2 Hz, H-6b), 4.34 (dd, 1H, H-6′a), 4.11 (d, 1H, $^3J_{2,3}$ = $^3J_{3,4}$ = 9.1 Hz, H-3), 4.11 (t, 1H, $^3J_{2',3'}$ = $^3J_{3',4'}$ = 9.3 Hz, H-3′), 4.07 (m, 1H, $^3J_{5,4}$ = $^3J_{6b,5}$ = 9.1 Hz, $^3J_{5,6a}$ = 4.3 Hz, H-5), 3.78 (td, 1H, $^3J_{5',4'}$ = $^3J_{6'b,5'}$ = 9.9 Hz, $^3J_{5',6'a}$ = 4.6 Hz, H-5′), 3.66 (t, 1H, $^2J_{6'a,6'b}$ = $^3J_{5',6'b}$ = 10.4 Hz, H-6′b), 3.65 (dd, 1H, $^3J_{3',2'}$ = 8.9 Hz, $^3J_{1',2'}$ = 3.1 Hz, H-2′), 3.58 (t, 1H, $^3J_{3,4}$ = $^3J_{4,5}$ = 9.5 Hz, H-4), 3.40 (t, 1H, $^3J_{3',4'}$ = $^3J_{4',5'}$ = 9.3 Hz, H-4′), 2.38 (d, 1H, C4-O*H*), 2.19 (s, 3H, C*H*$_3$, Ac), 0.93, 0.91, 0.80 (3×s, 27H,

3×[C*H*$_3$]$_3$C, 3xTBDMS), 0.16, 0.15, 0.13, 0.10, 0.02, 0.01 (6×s, 18H, 6×C*H*$_3$, 3xTBDMS)); ^{13}C NMR (151 MHz, CDCl$_3$): δ [ppm] = 170.15 (C=O, Ac), 155.34 (C=O, Alloc), 137.30 (C_q, Ph), 131.45 (*C*H, Alloc), 129.09, 128.08, 126.50 (Ph*C*H), 119.01 (CH$_2$, Alloc), 102.72 (Ph*C*H), 95.60 (C-1′), 91.30 (C-1), 82.49 (C-4′), 74.05 (C-2′), 73.14 (C-2), 71.98 (C-3), 71.52 (C-3′), 70.75 (C-4), 70.19 (C-5), 68.94 (C-6′), 68.70 (*C*H$_2$, Alloc), 66.25 (C-6), 63.74 (C-5′), 26.42, 26.21, 25.77 (3x[*C*H$_3$]$_3$C], 3xTBDMS), 21.02 (*C*H$_3$, Ac), 18.48, 18.22, 18.18 (3x[CH$_3$]$_3$*C*, 3xTBDMS), -3.02, -4.04, -4.22, -4.28, -4.35, -4.57 (6x*C*H$_3$, 3xTBDMS); HRMS ($^+$ESI) m/z: calcd. for $C_{43}H_{78}NO_{14}Si_3$ $[M+NH_4]^+$ 916.4725, found 916.4732.

Synthesis of **16**

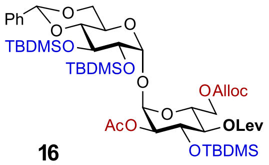

16

4,6-*O*-Benzylidene-2,3-di-*O*-*tert*-butyldimethylsilyl-α-D-glucopyranosyl-(1↔1)-2-*O*-acetyl-6-*O*-allyloxycarbonyl-3-*O*-*tert*-butyldimethylsilyl-4-*O*-(4-oxopentanoyl)-α-D-glucopyranoside (16)

To a stirred solution of **14** (0.27 g, 0.30 mmol) in dry CH_2Cl_2 (6.0 mL) levulinic acid (53 mg, 0.45 mmol), *N,N′*-diisopropylcarbodiimide (70 µL, 0.45 mmol) and DMAP (0.1 equiv., 0.03 mmol) were added successively. The reaction mixture was stirred for 24 h at r.t. and concentrated. The residue was redissolved in EtOAc (50 mL) and washed with aq. satd. $NaHCO_3$ (3×15 mL), and brine (2×15 mL). The organic layer was dried over Na_2SO_4, filtered and concentrated. The residue was purified by column chromatography on silica gel (toluene − EtOAc, 95/5 → 90/10) to afford **16** (0.287 g, 96%) as a syrup. R_f = 0.64 (toluene – EtOAc, 6/4, v/v); $[\alpha]_D^{20}$ = 70 (c = 0.5, CHCl$_3$); ^{1}H NMR (600 MHz, CDCl$_3$): δ [ppm] = 7.41-7.37 (m, 2H, *Ph*CH), 7.36-7.33 (m, 3H, *Ph*CH), 5.92 (m, 1H, C*H*, Alloc), 5.41 (s, 1H, PhC*H*), 5.38-5.23 (m, 2H, C*H*$_2$, Alloc), 5.33 (d, 1H, $^3J_{1,2}$ = 2.3 Hz, H-1), 5.06 (t, 1H, $^3J_{3,4}$ = $^3J_{4,5}$ = 9.4 Hz, H-4), 5.01 (d, 1H, $^3J_{1',2'}$ = 2.2 Hz, H-1′), 4.81 (dd, 1H, $^3J_{2,3}$ = 9.7 Hz, $^3J_{1,2}$ = 2.8 Hz, H-2), 4.61 (m, 2H, C*H*$_2$, Alloc), 4.31 (t, 1H, $^3J_{2,3}$ = $^3J_{3,4}$ = 9.4 Hz, H-3), 4.23 (dd, 1H, $^2J_{6a,6b}$ = 12.5 Hz, $^3J_{5,6a}$ = 5.1 Hz, H-6a), 4.19-4.06 (m, 4H, H-6b, H-5, H-3′, H-6′a), 3.75-3.61 (m, 3H, H-5′, H-2′, H-6′b), 3.41 (t, 1H, $^3J_{3',4'}$ = $^3J_{4',5'}$ = 9.0 Hz, H-4′), 2.88-2.50 (m, 4H, 2xC*H*$_2$, Lev), 2.19 (s, 3H, C*H*$_3$, Ac), 2.18 (s, 3H, C*H*$_3$, Lev), 0.95, 0.85, 0.81 (3×s, 27H, 3×[C*H*$_3$]$_3$C, 3xTBDMS), 0.14, 0.12, 0.10, 0.08, 0.02, 0.01 (6×s, 18H, 6×C*H*$_3$, 3xTBDMS); ^{13}C NMR (151 MHz, CDCl$_3$): δ [ppm] = 205.96 (CH$_3$*C*=O, Lev), 171.49 (*C*=O, Lev), 169.97 (*C*=O, Ac), 154.69 (*C*=O, Alloc), 137.17 (C_q, Ph), 131.57 (CH, Alloc), 129.10, 128.07, 126.44 (*C*H, Ph), 118.59 (CH$_2$, Alloc), 102.67 (Ph*C*H), 94.98 (C-1′), 90.21 (C-1), 82.26 (C-4′), 73.83 (C-2′), 73.33 (C-2), 71.51 (C-3′), 71.25 (C-4), 69.20 (C-3), 68.80 (C-6′), 68.52 (*C*H$_2$, Alloc), 68.47 (C-5), 65.70 (C-6), 63.73 (C-5′), 37.87 (*C*H$_2$, Lev), 29.72 (*C*H$_3$, Lev), 28.03 (*C*H$_2$, Lev), 26.40, 26.19, 25.52 (3x[*C*H$_3$]$_3$C], 3xTBDMS), 20.97 (*C*H$_3$, Ac), 18.47, 18.06, 17.88 (3x[CH$_3$]$_3$*C*, 3xTBDMS), -3.02, -4.00, -4.20, -4.43, -4.50, -4.55 (6x*C*H$_3$, 3xTBDMS); HRMS ($^+$ESI) m/z: calcd. for $C_{48}H_{81}NO_{16}Si_3$ $[M+H]^+$ 997.4827, found 997.4837; calcd. for $C_{48}H_{81}KO_{16}Si_3$ $[M+K]^+$ 1035.4386, found 1035.4404.

Synthesis of **20**

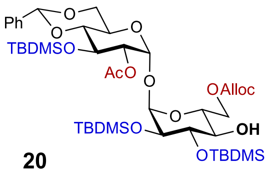

20

2-*O*-Acetyl-4,6-*O*-benzylidene-3-*O*-*tert*-butyldimethylsilyl-α-D-glucopyranosyl-(1↔1)-6-*O*-allyloxycarbonyl-2,3-di-*O*-*tert*-butyldimethylsilyl-α-D-glucopyranoside (20)

To a stirred solution of **11** (0.150 g, 0.167 mmol) in dry CH_2Cl_2 (4 mL) collidine (350 µL, 1.67 mmol) and allyloxycarbonyl chloride (280 µL, 1.67 mmol) were added successively at 0° C. The reaction mixture was stirred for 18 h at r.t., diluted with toluene (10 mL) and concentrated. The residue was redissolved in EtOAc (50 mL) and washed with aq. satd. $NaHCO_3$ (3×15 mL), aq. satd. $CuSO_4$ (5×10 mL) and brine (2 × 20 mL). The organic layer was dried over Na_2SO_4, filtered and concentrated. The residue was purified by column chromatography on silica gel (toluene − EtOAc, 90/10 → 80/20) to afford **20** (0.130 g, 78%) as a syrup. R_f = 0.76 (toluene – EtOAc, 6/4, v/v); R_f = 0.38 (toluene – EtOAc, 9/1, v/v); $[\alpha]_D^{20}$ = 73 (c = 1.5, $CHCl_3$); ^{1}H NMR (600 MHz, $CDCl_3$): δ [ppm] = 7.47-7.43 (m, 2H, *Ph*CH), 7.38-7.34 (m, 3H, *Ph*CH), 5.94 (m, 1H, C*H*, Alloc), 5.50 (s, 1H, PhC*H*), 5.40-5.26 (m, 2H, C*H*$_2$, Alloc), 5.27 (d, 1H, $^3J_{1',2'}$ = 3.8 Hz, H-1′), 5.06 (d, 1H, $^3J_{1,2}$ = 2.8 Hz, H-1), 4.88 (dd, 1H, $^3J_{2',3'}$ = 9.5 Hz, $^3J_{1',2'}$ = 3.7 Hz, H-2′), 4.65 (m, 2H, C*H*$_2$, Alloc), 4.44 (dd, 1H, $^2J_{6a,6b}$ = 11.8 Hz, $^3J_{5,6a}$ = 5.2 Hz, H-6a), 4.28 (dd, 1H, $^2J_{6a,6b}$ = 11.8 Hz, $^3J_{5,6b}$ = 2.5 Hz, H-6b), 4.21 (t, 1H, $^2J_{6'a,6'b}$ = 10.4 Hz, $^3J_{5',6'a}$ = 4.0 Hz, H-6′a), 4.21 (t, 1H, $^3J_{2',3'}$ = $^3J_{3',4'}$ = 9.4 Hz, H-3′), 4.03 (t, 1H, $^3J_{2,3}$ = $^3J_{3,4}$ = 8.3 Hz, H-3), 4.02 (td, 1H, $^3J_{4',5'}$ = $^3J_{5',6'b}$ = 10.0 Hz, $^3J_{5',6'a}$ = 4.8 Hz, H-5′), 3.77 (m, 1H, $^3J_{4,5}$ = $^3J_{5,6b}$ = 8.2 Hz, $^3J_{5,6a}$ = 2.6 Hz, H-5), 3.71 (t, 1H, $^2J_{6'a,6'b}$ = $^3J_{5',6'b}$ = 10.4 Hz, H-6′b), 3.61 (dd, 1H, $^3J_{2,3}$ = 8.6 Hz, $^3J_{1,2}$ = 2.7 Hz, H-2), 3.52 (t, 1H, $^3J_{3',4'}$ = $^3J_{4',5'}$ = 9.4 Hz, H-4′), 3.41 (t, 1H, $^3J_{3,4}$ = $^3J_{4,5}$ = 8.4 Hz, H-4), 2.57 (d, 1H, C4-O*H*), 2.10 (s, 3H, C*H*$_3$, Ac), 0.96, 0.95, 0.82 (3×s, 27H, 3×[C*H*$_3$]$_3$C, 3xTBDMS), 0.17, 0.17, 0.15, 0.14, 0.14, 0.04, -0.02 (6×s, 18H, 6×C*H*$_3$, 3xTBDMS); ^{13}C NMR (151 MHz, $CDCl_3$): δ [ppm] = 170.04 (C=O, Ac), 155.27 (C=O, Alloc), 137.37 (C_q, Ph), 131.51 (CH, Alloc), 128.94, 128.03, 126.43 (CH, Ph), 119.00 (CH_2, Alloc), 102.24 (Ph*C*H), 94.00 (C-1), 91.97 (C-1′), 82.08 (C-4′), 73.86 (C-3), 73.61 (C-2′),72.85 (C-2),71.81 (C-5), 70.91 (C-4), 69.38 (C-3′), 68.93 (C-6′), 68.75 (CH_2, Alloc), 66.42 (C-6), 63.40 (C-5′), 26.51, 26.23, 25.64 (3x[*C*H$_3$]$_3$C], 3xTBDMS), 20.84 (*C*H$_3$, Ac), 18.41, 18.36, 18.14 (3x[CH$_3$]$_3$*C*, 3xTBDMS), -3.28, -3.89, -4.17, -4.38, -4.41, -4.99 (6x*C*H$_3$, 3xTBDMS); HRMS ($^+$ESI) m/z: calcd. for $C_{43}H_{78}NO_{14}Si_3$ $[M+NH_4]^+$ 916.4725, found 916.4734; calcd. for $C_{43}H_{74}KO_{14}Si_3$ $[M+K]^+$ 937.4018, found 937.40.

Synthesis of **22**

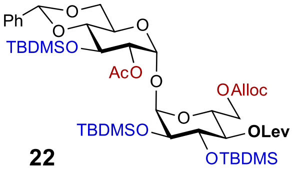

22

2-*O*-Acetyl-4,6-*O*-benzylidene-3-*O*-*tert*-butyldimethylsilyl-α-D-glucopyranosyl-(1↔1)-6-*O*-allyloxycarbonyl-2,3-di-*O*-*tert*-butyldimethylsilyl-4-*O*-(4-oxopentanoyl)-α-D-glucopyranoside (22)

To a stirred solution of **20** (0.107 g, 0.119 mmol) in dry CH_2Cl_2 (3 mL) levulinic acid (21 mg, 0.179 mmol), *N*,*N'*-diisopropylcarbodiimide (27 µL, 0.179 mmol) and catalytic amount of DMAP (0.1 equiv., 0.01 mmol) were added successively. The reaction mixture was stirred for 24 h at r.t. and then concentrated. The residue was dissolved in EtOAc (50 mL) and washed with aq. satd. $NaHCO_3$ (3×10 mL), and brine (2×10 mL). The organic layer was dried over Na_2SO_4, filtered and concentrated. The residue was purified by column chromatography on silica gel (toluene – EtOAc, 95/5 → 90/10) to afford **22** (0.113 g, 95%) as a syrup. R_f = 0.64 (toluene – EtOAc, 6/4, v/v); $[\alpha]_D^{20}$ = -1.3 (c = 0.2, $CHCl_3$); ^{1}H NMR (600 MHz, $CDCl_3$): δ [ppm] = 7.47-7.43 (m, 2H, *Ph*CH), 7.37-7.34 (m, 3H, *Ph*CH), 5.93 (m, 1H, C*H*, Alloc), 5.50 (s, 1H, PhC*H*), 5.38-5.23 (m, 2H, CH_2, Alloc), 5.22 (d, 1H, $^3J_{1',2'}$ = 3.5 Hz, H-1′), 5.08 (d, 1H, $^3J_{1,2}$ = 2.8 Hz, H-1), 4.92 (dd, 1H, $^3J_{3',2'}$ = 9.5 Hz, $^3J_{1',2'}$ = 3.6 Hz, H-2′), 4.88 (t, 1H, $^3J_{3,4}$ = $^3J_{4,5}$ = 9.3 Hz, H-4), 4.62 (d, 1H, CH_2, Alloc), 4.22 (t, 1H, *J* = 9.3 Hz, H-3´), 4.20 (t, 1H, *J* = 9.1 Hz, H-3), 4.25-4.18 (m, 1H, H6′a), 4.14-4.10 (m, 2H, H-6a,H-6b), 4.05 (m, $^3J_{5',4'}$ = $^3J_{6'b,5'}$ = 9.9 Hz, $^3J_{5',6'a}$ = 4.7 Hz, H-5′), 3.85 (m, 1H, $^3J_{5,4}$ = $^3J_{6b,5}$ = 9.3 Hz, $^3J_{5,6a}$ = 4.8 Hz, H-5), 3.71 (t, 1H, $^2J_{6'a,6'b}$ = $^3J_{5',6'b}$ = 10.3 Hz, H-6′b), 3.70 (dd, 1H, $^3J_{2,3}$ = 9.1 Hz, $^3J_{1,2}$ = 2.9 Hz, H-2), 3.53 (t, 1H, $^3J_{3',4'}$ = $^3J_{4',5'}$ = 9.4 Hz, H-4′), 2.89-2.52 (m, 4H, 2xCH_2, Lev), 2.18 (s, 3H, CH_3, Ac), 2.14 (s, 3H, CH_3, Lev), 0.96, 0.88, 0.83 (3×s, 27H, 3×[CH_3]$_3$C, 3xTBDMS), 0.15, 0.14, 0.13, 0.12, 0.04, 0.02 (6×s, 18H, 6×CH_3, 3xTBDMS); ^{13}C NMR (151 MHz, $CDCl_3$): δ [ppm] = 218.00 (CH_3*C*=O, Lev), 171.93 (C=O, Lev), 169.85 (C=O, Ac), 154.70 (C=O, Alloc), 137.36 (C_q, Ph), 131.69 (CH, Alloc), 128.97, 128.04, 126.45 (CH, Ph), 118.70 (CH_2, Alloc), 102.29 (Ph*C*H), 94.16 (C-1), 91.98 (C-1′), 82.05 (C-4′), 73.44 (C-2′), 73.26 (C-2), 72.06 (C-4), 71.16 (C-3), 69.41 (C-5, C-3′), 68.92 (C-6′), 68.63 (CH_2, Alloc), 66.44 (C-6), 63.59 (C-5′), 38.02 (CH_2, Lev), 29.69 (CH_3, Lev), 28.35 (CH_2, Lev), 26.63, 26.04, 25.65 (3x[*C*H_3]$_3$C], 3xTBDMS), 20.81 (*C*H_3C=O, Ac), 18.49, 18.17, 18.03 (3x[CH_3]$_3$*C*, 3xTBDMS), -2.98, -3.89, -4.11, -4.26, -4.39, -4.99 (6x*C*H_3, 3xTBDMS); HRMS ($^+$ESI) m/z: calcd. for $C_{48}H_{84}NO_{16}Si_3$ $[M+NH_4]^+$ 1014.5092, found 1014.5108; calcd. for $C_{48}H_{80}KO_{16}Si_3$ $[M+K]^+$ 1035.4386, found 1035.4388.

Optimisation of reaction conditions for regioselective cleavage of TBDMS groups in 22 and 28

The cleavage of the TBDMS groups in **17** was initially performed using a concentrated solution of 3HF·Et_3N, which resulted in the complete removal of all three TMDMS groups to afford **18** in 77% yield (SI-Table 1, entry 1).

Reducing the concentration of [3HF·Et_3N] in solution to 0.77 M and shortening the reaction time from 24 h to 8 h afforded **18** in only 14% yield and two partially deprotected compounds **26** and **S5**+**S6** (SI-Table 1, entry 2). Further decreasing the concentration of [3HF·Et_3N] in the reaction solution to 0.24 M and buffering the reaction with triethylamine to pH=7 afforded a complex mixture of partially TBDMS-protected compounds **26**, **27**, **S5-S7** and **18** within 18 h (SI-Table 1, entry 3). Changing the solvent to DMF significantly accelerated the reaction rate and resulted in the formation of **26** as the major product using 0.74 M [3HF·Et_3N] is solution (SI-Table 1, entry 4). Lowering the concentration of [3HF·Et_3N] in the reaction solution (DMF) to 0.25 M afforded **26** and **27** as the main products, while the unreacted starting material was fully recovered (SI-Table 1, entry 5). Partially TBDMS-protected derivatives were readily isolated in pure form by conventional chromatography on silica gel.

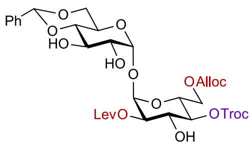

18

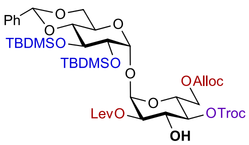

26

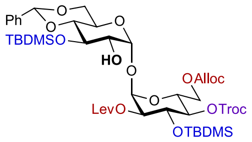

27

17 → 18

17 → 26 + 27

17 → 26 + S5 + S6 + S7

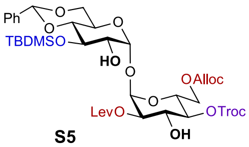

S5

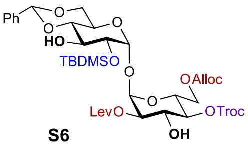

S6

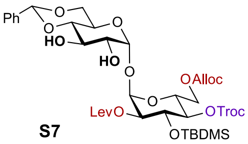

S7

SI-Table 1. Optimisation of reaction conditions for regioselective cleavage of TBDMS groups in **17**.

Entry	[3HF·Et_3N][b]	Solvent	React.time[c]	Conversion[g]	**27**[d]	**26**[d]	**S5**[d]	**S6**[d]	**S7**[d]	**18**[d]
1	1.2	THF	24	87	n.i.	6	5		1	77
2	0.8	THF	8	92	n.i.	17	13		5	14
3[a]	0.25	THF	18	80	12	6	10		6	20
4	0.75	DMF	1.5	88	9	19	21 (1:0.18)[e]		2	4
5	0.20	DMF	1.5	49	**30**	**47**	8		n.i	n.i

[a]Reaction was performed in neutral conditions (pH=7), all other transformations included in the SI-Table 1 were carried out in acidic conditions (pH=3)
[b]Final concentration of 3HF·Et_3N in the reaction mixture [M]
[c]Reaction time is given in [h], [d]Isolated yield based on reacted starting material, BRSM [%]
[e,f]The ratio of regio-isomers was determined by ^{1}H-NMR analysis of isolated mixture of **S5 + S6**
[g]Overall conversion [%], the yields for all isolated compounds are based on the recovered starting material (BRSM)
n.i. = not isolated;

Similarly, the use of THF as the reaction solvent for the partial deprotection of **23** promoted by [3HF·Et_3N] (0.24 M) required longer reaction times (24 h) and resulted in almost complete conversion of the starting **23**, giving a 1:2:1 mixture of partially TBDMS-deblocked **29**, **S8+S9** and fully TBDMS-deprotected **24** (SI-Table 2, entry 1). Shortening the reaction time to 6 h while using the same concentration of [3HF·Et_3N] buffered with triethylamine until pH = 7 resulted in a lower conversion of the starting material (48%) and again gave the complex mixture of positional isomers (SI-Table 2, entry 2). To accelerate the reaction rate, the solvent was changed to DMF and the concentration of [3HF·Et_3N] in the reaction solution was increased to 0.74 M. These conditions afforded a mixture of four compounds: **28** (17%), **S8+S9** (45%) and **24** (23%) (SI-Table 2, entry 3). To reduce the amount of fully TBDMS-deblocked **24**, the reaction was stopped after 1 h, yielding **28** and **S8+S9** as the main products (SI-Table 2, entry 4). Using a lower concentration of [3HF·Et_3N] in reaction solution (DMF) and reducing the reaction time to 1.2 h afforded **28** as the main product (53% BRSM) together with **29** (19% BRSM) and **30** (10% BRSM) (SI-Table 2, entry 5), all partially TBDMS-protected derivatives were readily isolated in pure form using conventional column chromatography on silica gel.

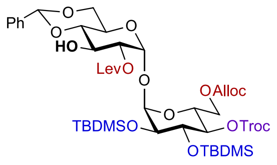

28

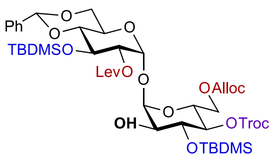

29

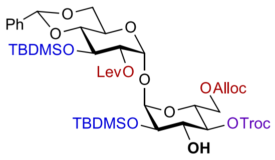

30

23 → 24
23 → 28 + 29 + 30
23 → 28 + S8 + S9 + 24

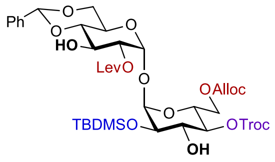

S8

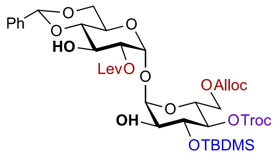

S9

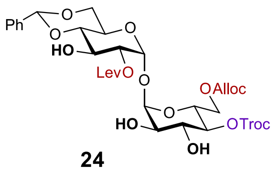

24

SI-Table 2. Optimisation of reaction conditions for regioselective cleavage of TBDMS groups in **23**

Entry	[HF·Et_3N][b]	solvent	React. time[c]	conversion[g]	**30**[d]	**29**[d]	**28**[d]	**S8**[d]	**S9**[d]	**24**[d]
1	0.25	THF	24	84	n.i.	22	6	50(1:0.18)[f]		25
2[a]	0.25	THF	6	48	n.i.	25	15	18		8
3	0.75	DMF	5	100	n.i.	<1	17	45		23
4	0.75	DMF	1	84	4	7	40	33		6
5	0.20	DMF	1.5	34	**10**	**19**	**53**	3		n.i

[a]Reaction was performed in neutral conditions (pH=7), all other transformations included in the SI-Table 2 were carried out in acidic conditions (pH=3)
[b]Final concentration of 3HF·Et_3N in the reaction mixture [M]
[c]Reaction time is given in [h]
[d]Isolated yield based on reacted starting material, BRSM [%]
[e,f]The ratio of regio-isomers was determined by ^{1}H-NMR analysis of isolated mixture of **S8** and **S9**
[g]Overall conversion [%]; the yields for all isolated compounds are based on the recovered starting material (BRSM)
n.i. = not isolated;

Synthesis of S5

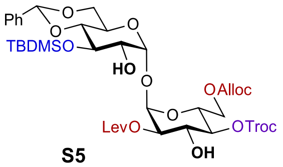

S5

4,6-*O*-Benzylidene-3-*O*-*tert*-butyldimethylsilyl-α-D-glucopyranosyl-(1↔1)-6-*O*-allyloxycarbonyl-2-*O*-(4-oxopentanoyl)-4-*O*-(2,2,2-trichloroethoxycarbonyl)-α-D-glucopyranoside (S5)

See SI-Table 1 for conditions. R_f = 0.50 (toluene – EtOAc, 1/1, v/v); R_f = 0.17 (toluene – EtOAc, 8/2, v/v); $[\alpha]_D^{20}$ = -8.0 (c = 0.3, $CHCl_3$); ^{1}H NMR (600 MHz, $CDCl_3$): δ [ppm] = 7.48-7.43 (m, 2H, *Ph*CH), 7.38-7.33 (m, 3H, *Ph*CH), 5.92 (m, 1H, C*H*, Alloc), 5.48 (s, 1H, PhC*H*), 5.38-5.25 (m, 2H, CH_2, Alloc), 5.27 (d, 1H, $^3J_{1,2}$ = 3.8 Hz, H-1), 5.15 (d, 1H, $^3J_{1',2'}$ = 3.8 Hz, H-1′), 4.98 (dd, 1H, $^3J_{2,3}$ = 10.0 Hz, $^3J_{1,2}$ = 3.8 Hz, H-2), 4.98 (t, 1H, $^3J_{3,4}$ = $^3J_{4,5}$ = 9.7 Hz, H-4), 4.83 (m, 2H, CH_2, Troc), 4.61 (m, 2H, CH_2, Alloc), 4.39-4.32 (m, 3H, H-5, H-6a, H-3), 4.23 (dd, 1H, $^2J_{6a,6b}$ = 11.6 Hz, $^3J_{5,6b}$ = 2.2 Hz, H-6b), 4.18 (t, 1H, $^2J_{6'a,6'b}$ = 10.3 Hz, $^3J_{5',6'a}$ = 4.8 Hz, H-6′a), 3.92 (t, 1H, $^3J_{2',3'}$ = $^3J_{3',4'}$ = 9.0 Hz, H-3′), 3.84 (td, 1H, $^3J_{4',5'}$ = $^3J_{5',6'b}$ = 10.1 Hz, $^3J_{5',6'a}$ = 4.8 Hz, H-5′), 3.70 (dd, 1H, $^3J_{2',3'}$ = 8.5 Hz, $^3J_{1',2'}$ = 4.0 Hz, H-2′), 3.70 (t, 1H, $^2J_{6'a,6'b}$ = $^3J_{5',6'b}$ = 10.4 Hz, H-6′b), 3.44 (t, 1H, $^3J_{3',4'}$ = $^3J_{4',5'}$ = 9.3 Hz, H-4′), 2.86-2.54 (m, 4H, 2xCH_2, Lev), 2.66 (s, 1H, C2′-OH), 2.10 (s, 3H, CH_3, Lev), 0.87 (s, 9H, ([CH_3]$_3$C, TBDMS), 0.14, 0.04 (2×s, 6H, 2×CH_3, TBDMS); ^{13}C NMR (151 MHz, MeOD): δ [ppm] = 207.59 (*C*H_3C=O, Lev), 172.08 (C=O, Lev), 154.71 (C=O, Alloc), 153.62 (C=O, Troc), 137.16 (C_q, Ph), 131.42 (*C*H, Alloc), 129.04, 128.12, 126.20 (CH, Ph), 119.08 (*C*H_2, Alloc), 101.88 (Ph*C*H), 95.08 (C-1′), 94.29 (*C*Cl_3,Troc), 92.06 (C-1), 81.56 (C-4′), 77.02 (*C*H_2, Troc), 75.10 (C-4), 72.99 (C-2′), 72.72 (C-2), 72.40 (C-3′), 69.15 (C-3), 68.79 (C-6′), 68.76 (CH_2, Alloc), 67.36 (C-5), 65.25 (C-6), 63.46 (C-5′), 37.99 (CH_2, Lev), 29.66 (CH_3, Lev), 27.80 (CH_2, Lev), 25.85 ([*C*H_3]$_3$C], TBDMS), 18.24 ([CH_3]$_3$*C*, TBDMS), -4.15, -4.70 (2xCH_3, TBDMS); HRMS (+ESI) m/z: calcd for $C_{37}H_{55}NO_{17}Si$ $[M+NH_4]^+$ 918.2299, found 918.2287.

Synthesis of S6

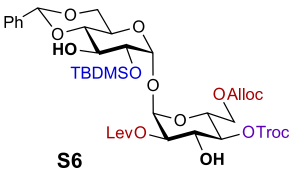

S6

4,6-*O*-Benzylidene-2-*O*-*tert*-butyldimethylsilyl-α-D-glucopyranosyl-(1↔1)-6-*O*-allyloxycarbonyl-2-*O*-(4-oxopentanoyl)-4-*O*-(2,2,2-trichloroethoxycarbonyl)-α-D-glucopyranoside (S6)

See SI-Table 1 for conditions. R_f = 0.50 (toluene – EtOAc, 1/1, v/v); R_f = 0.17 (toluene – EtOAc, 8/2, v/v); $[\alpha]_D^{20}$ = -8.0 (c = 0.3, $CHCl_3$); ^{1}H NMR (600 MHz, $CDCl_3$): δ [ppm] = 7.46-7.42 (m, 2H, *Ph*CH), 7.39-7.34 (m, 3H, *Ph*CH), 5.93 (m, 1H, C*H*, Alloc), 5.52 (s, 1H, PhC*H*), 5.39-5.26 (m, 2H, CH_2, Alloc), 5.30 (d, 1H, $^3J_{1,2}$ = 3.7 Hz, H-1), 5.01 (d, 1H, $^3J_{1',2'}$ = 3.5 Hz, H-1′), 4.97 (t, 1H, $^3J_{3,4}$ = $^3J_{4,5}$ = 9.7 Hz, H-4), 4.96 (dd, 1H, $^3J_{2,3}$ = 10.0 Hz, $^3J_{1,2}$ = 3.7 Hz, H-2), 4.84-4.74 (m, 2H, CH_2, Troc), 4.63 (m, 2H, CH_2, Alloc), 4.36-4.29 (m, 3H, H-5, H-6a,

H-3), 4.26 (dd, 1H, $^{2}J_{6a,6b}$ = 12.0 Hz, $^{3}J_{5,6b}$ = 2.4 Hz, H-6b), 4.21 (dd, 1H, $^{2}J_{6'a,6'b}$ = 10.3 Hz, $^{3}J_{5',6'a}$ = 4.8 Hz, H-6′a), 4.02 (t, 1H, $^{3}J_{2',3'}$ = $^{3}J_{3',4'}$ = 9.2 Hz, H-3′), 3.94 (td, 1H, $^{3}J_{4',5'}$ = $^{3}J_{5',6'b}$ = 10.0 Hz, $^{3}J_{5',6'a}$ = 4.8 Hz, H-5′), 3.74 (dd, 1H, $^{3}J_{2',3'}$ = 9.0 Hz, $^{3}J_{1',2'}$ = 3.5 Hz, H-2′), 3.71 (t, 1H, $^{2}J_{6'a,6'b}$ = $^{3}J_{5',6'b}$ = 10.4 Hz, H-6′b), 3.51 (t, 1H, $^{3}J_{3',4'}$ = $^{3}J_{4',5'}$ = 9.5 Hz, H-4′), 2.80-2.58 (m, 4H, 2xC*H*2, Lev), 2.08 (s, 3H, C*H*3, Lev), 0.92 (s, 9H, [C*H*3]3C, TBDMS), 0.17, 0.10 (2×s, 6H, 2×C*H*3, TBDMS); ^{13}C NMR (151 MHz, $CDCl_3$): δ [ppm] = 207.14 (*C*H3C=O, Lev), 172.21 (C=O, Lev), 154.67 (C=O, Alloc), 153.56 (C=O, Troc), 137.05 (C_q, Ph), 131.36 (*C*H, Alloc), 129.21, 128.29, 126.13 (CH, Ph), 119.00 (*C*H2, Alloc), 101.89 (Ph*C*H), 95.65 (C-1′), 94.26 (*C*Cl3, Troc), 91.67 (C-1), 81.10 (C-4′), 74.92 (C-4), 73.25 (C-2′), 72.67 (C-2), 70.78 (C-3′), 69.43 (C-3), 68.80 (C-6′), 68.77 (CH_2, Alloc), 67.53 (C-5), 65.10 (C-6), 63.10 (C-5′), 38.12 (CH_2, Lev), 29.61 (CH_3, Lev), 27.95 (CH_2, Lev), 25.81 ([*C*H3]3C], TBDMS), 18.21 ([CH3]3*C*, TBDMS), -4.31, -4.66 (2xCH_3, TBDMS); HRMS (+ESI) m/z: calcd for $C_{37}H_{55}NO_{17}Si$ $[M+NH_4]^+$ 918.2299, found 918.2287.

Synthesis of S7

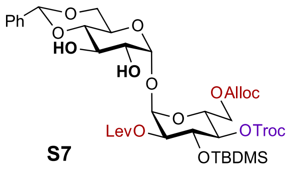

S7

4,6-*O*-Benzylidene-α-D-glucopyranosyl-(1↔1)-6-*O*-allyloxycarbonyl-2-*O*-(4-oxopentanoyl)-3-*O*-*tert*-butyldimethylsilyl-4-*O*-(2,2,2-trichloroethoxycarbonyl)-α-D-glucopyranoside (S7)

See SI-Table 1 for conditions. R_f = 0.20 (toluene – EtOAc, 1/1, v/v); R_f = 0.06 (toluene – EtOAc, 8/2, v/v); $[\alpha]_D^{20}$ = 81 (c = 0.5, $CHCl_3$); ^{1}H NMR (600 MHz, $CDCl_3$): δ [ppm] = 7.48-7.43 (m, 2H, *Ph*CH), 7.40-7.34 (m, 3H, *Ph*CH), 5.93 (m, 1H, C*H*, Alloc), 5.53 (s, 1H, PhC*H*), 5.40-5.26 (m, 2H, C*H*2, Alloc), 5.38 (d, 1H, $^{3}J_{1,2}$ = 3.3 Hz, H-1), 5.15 (d, 1H, $^{3}J_{1',2'}$ = 3.8 Hz, H-1′), 4.86-4.62 (m, 2H, C*H*2, Troc), 4.85 (t, 1H, $^{3}J_{3,4}$ = $^{3}J_{4,5}$ = 9.6 Hz, H-4), 4.81 (dd, 1H, $^{3}J_{2,3}$ = 9.6 Hz, $^{3}J_{1,2}$ = 3.8 Hz, H-2), 4.63-4.61 (m, 2H, C*H*2, Alloc), 4.36 (dt, 1H, $^{3}J_{4,5}$ = $^{3}J_{5,6b}$ = 10.3 Hz, $^{3}J_{5,6a}$ = 3.9 Hz, H-5), 4.31 (dd, 1H, $^{2}J_{6'a,6'b}$ = 10.1 Hz, $^{3}J_{5',6'a}$ = 4.5 Hz, H-6′a), 4.26 (m, 2H, $^{2}J_{6a,6b}$ = 12.0 Hz, $^{3}J_{5,6}$ = 2.6 Hz, H-6a, H-6b), 4.24 (t, 1H, $^{3}J_{2,3}$ = $^{3}J_{3,4}$ = 9.1 Hz, H-3), 4.01 (t, 1H, $^{3}J_{2',3'}$ = $^{3}J_{3',4'}$ = 9.3 Hz, H-3′), 3.79 (td, 1H, $^{3}J_{4',5'}$ = $^{3}J_{5',6'b}$ = 10.1 Hz, $^{3}J_{5',6'a}$ = 4.5 Hz, H-5′), 3.76 (dd, 1H, $^{3}J_{2',3'}$ = 8.9 Hz, $^{3}J_{1',2'}$ = 4.1 Hz, H-2′), 3.73 3.72 (t, 1H, $^{2}J_{6'a,6'b}$ = $^{3}J_{5',6'b}$ = 10.4 Hz, H-6′b), 3.53 (t, 1H, $^{3}J_{3',4'}$ = $^{3}J_{4',5'}$ = 9.3 Hz, H-4′), 2.79-2.62 (m, 4H, 2xC*H*2, Lev), 2.67 (d, 1H, C3′-OH), 2.41 (d, 1H, C2′-OH), 2.06 (s, 3H, C*H*3, Lev), 0.84 (s, 9H, [C*H*3]3C, TBDMS), 0.12, 0.11 (2×s, 6H, 2×C*H*3, TBDMS); ^{13}C NMR (151 MHz, $CDCl_3$): δ [ppm] = 205.80 (*C*H3C=O, Lev), 172.05 (C=O, Lev), 154.68 (C=O, Alloc), 153.27 (C=O, Troc), 136.91 (C_q, Ph), 131.41 (*C*H, Alloc), 129.34, 128.38, 126.18 (CH, Ph), 119.20 (*C*H2, Alloc), 101.99 (Ph*C*H), 94.64 (C-1′), 91.65 (C-1), 80.91 (C-4′), 76.51 (C-4), 73.48 (C-2), 72.10 (C-3′), 71.20 (C-2′), 69.44 (C-5), 68.83 (C-6′), 68.70 (CH_2, Alloc), 67.25 (C-3), 65.76 (C-6), 63.47 (C-5′), 37.53 (CH_2, Lev), 29.66 (CH_3, Lev), 27.78 (CH_2, Lev), 25.50 ([*C*H3]3C], TBDMS), 17.88 ([CH3]3*C*, TBDMS), -4.36, -4.75 (2xCH_3, TBDMS); HRMS (+ESI) m/z: calcd for $C_{37}H_{55}NO_{17}Si$ $[M+K]^+$ 939.1593, found 939.1579.

Synthesis of S8

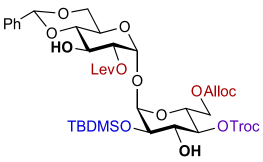

S8

4,6-*O*-Benzylidene-2-*O*-(4-oxopentanoyl)-α-D-glucopyranosyl-(1↔1)-6-*O*-allyloxycarbonyl-2-*O*-*tert*-butyldimethylsilyl-4-*O*-(2,2,2-trichloroethoxycarbonyl)-α-D-glucopyranoside (S8)

See SI-Table 2 for conditions. R_f = 0.16 (toluene – EtOAc, 8/2, v/v); $[\alpha]_D^{20}$ = 67 (c = 0.2, $CHCl_3$); ^{1}H NMR (600 MHz, $CDCl_3$): δ [ppm] = 7.48-7.44 (m, 2H, *Ph*CH), 7.40-7.35 (m, 3H, *Ph*CH), 5.93 (m, 1H, C*H*, Alloc), 5.53 (s, 1H, PhC*H*), 5.39-5.26 (m, 2H, CH_2, Alloc), 5.21 (d, 1H, $^3J_{1',2'}$ = 3.8 Hz, H-1′), 5.07 (d, 1H, $^3J_{1,2}$ = 3.4 Hz, H-1), 5.02 (dd, 1H, $^3J_{2',3'}$ = 9.7 Hz, $^3J_{1',2'}$ = 3.7 Hz, H-2′), 4.86 (t, 1H, $^3J_{3,4}$ = $^3J_{4,5}$ = 9.7 Hz, H-4), 4.82-4.77 (m, 2H, CH_2, Troc), 4.62 (m, 2H, CH_2, Alloc), 4.35 (dd, 1H, $^2J_{6a,6b}$ = 12.1 Hz, $^3J_{5,6a}$ = 4.7 Hz, H-6a), 4.27 (dd, 1H, $^2J_{6a,6b}$ = 12.1 Hz, $^3J_{5,6b}$ = 2.6 Hz, H-6b), 4.25 (t, 1H, $^2J_{6'a,6'b}$ = $^3J_{5',6'a}$ = 10.4 Hz, H-6′a), 4.23 (t, 1H, $^3J_{2',3'}$ = $^3J_{3',4'}$ = 10.0 Hz, H-3′), 4.19-4.14 (m, 1H, $^3J_{4,5}$ = $^3J_{5,6b}$ = 10.2 Hz, $^3J_{5,6a}$ = 4.0 Hz, H-5), 4.16 (t, 1H, $^3J_{2,3}$ = $^3J_{3,4}$ = 9.2 Hz, H-3), 4.11 (td, 1H, $^3J_{4',5'}$ = $^3J_{5',6'b}$ = 9.9 Hz, $^3J_{5',6'a}$ = 4.7 Hz, H-5′), 3.74 (t, 1H, $^2J_{6'a,6'b}$ = $^3J_{5',6'b}$ = 10.2 Hz, H-6′b), 3.72 (dd, 1H, $^3J_{2,3}$ = 9.5 Hz, $^3J_{1,2}$ = 3.5 Hz, H-2), 3.60 (t, 1H, $^3J_{3',4'}$ = $^3J_{4',5'}$ = 9.5 Hz, H-4′), 2.87-2.62 (m, 4H, 2xCH_2, Lev), 2.22 (d, 1H, C3-OH) 2.18 (s, 3H, CH_3, Lev), 0.97 (s, 9H, (CH_3)$_3$C, TBDMS), 0.18, 0.15 (2×s, 6H, 2×CH_3, TBDMS); ^{13}C NMR (151 MHz, $CDCl_3$): δ [ppm] = 206.67 (*C*H$_3$C=O, Lev), 172.15 (C=O, Lev), 154.69 (C=O, Alloc), 153.65 (C=O, Troc), 136.95 (C_q, Ph), 131.44 (*C*H, Alloc), 129.33, 128.30, 126.50 (CH, Ph), 119.08 (*C*H$_2$, Alloc), 102.46 (Ph*C*H), 94.16 (C-1), 92.49 (C-1′), 81.33 (C-4′), 77.06, (*C*H$_2$, Troc), 75.31 (C-4), 72.83 (C-2), 72.65 (C-2′), 71.51 (C-3), 68.84 (C-6′), 68.81 (CH_2, Alloc), 68.64 (C-3′), 67.81 (C-5), 65.72 (C-6), 62.94 (C-5′), 37.98 (CH_2, Lev), 29.71 (CH_3, Lev), 27.94 (CH_2, Lev), 25.89 ([*C*H$_3$]$_3$C], TBDMS), -4.04, -4.79 (2xCH_3, TBDMS); HRMS ($^+$ESI) m/z: calcd for $C_{37}H_{55}Cl_3NO_{17}Si$ $[M+NH_4]^+$ 920.2280, found 920.2281.

Synthesis of S9

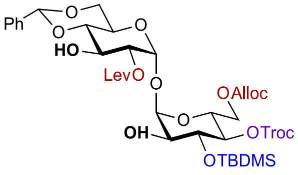

S9

4,6-*O*-Benzylidene-2-*O*-(4-oxopentanoyl)-α-D-glucopyranosyl-(1↔1)-6-*O*-allyloxycarbonyl-3-*O*-*tert*-butyldimethylsilyl-4-*O*-(2,2,2-trichloroethoxycarbonyl)-α-D-glucopyranoside (S9)

See SI-Table 2 for conditions. R_f = 0.58 (toluene – EtOAc, 1/1, v/v); R_f = 0.12 (toluene – EtOAc, 8/2, v/v); $[\alpha]_D^{20}$ = 148 (c = 0.3, $CHCl_3$); ^{1}H NMR (600 MHz, $CDCl_3$): δ [ppm] = 7.52-7.47 (m, 2H, *Ph*CH), 7.40-7.34 (m,

3H, *Ph*CH), 5.92 (m, 1H, C*H*, Alloc), 5.56 (s, 1H, PhC*H*), 5.38-5.25 (m, 2H, CH_2, Alloc), 5.24 (d, 1H, $^3J_{1',2'}$ = 3.8 Hz, H-1′), 5.17 (d, 1H, $^3J_{1,2}$ = 3.7 Hz, H-1), 4.99 (dd, 1H, $^3J_{2',3'}$ = 9.7 Hz, $^3J_{1',2'}$ = 3.8 Hz, H-2′), 4.86-4.62 (m, 2H, CH_2, Troc), 4.80 (t, 1H, $^3J_{3,4}$ = $^3J_{4,5}$ = 9.6 Hz, H-4), 4.61 (m, 2H, CH_2, Alloc), 4.29 (td, 2H, $^2J_{6a,6b}$ = 10.1 Hz, $^3J_{5,6}$ = 4.9 Hz, H-6a, H-6b), 4.23 (dd, 1H, $^2J_{6'a,6'b}$ = 12.0 Hz = $^3J_{5',6'a}$ = 3.0 Hz , H-6′a), 4.19 (t, 1H, $^3J_{2',3'}$ = $^3J_{3',4'}$ = 9.5 Hz, H-3′), 4.15-4.08 (m, 2H, H-5, H-5‘), 4.05 (d, 1H, $^3J_{2,3}$ = $^3J_{3,4}$ = 9.1 Hz, H-3), 3.74 (t, 1H, $^2J_{6'a,6'b}$ = $^3J_{5',6'b}$ = 10.3 Hz, H-6′b), 3.70 (dd, 1H, $^3J_{2,3}$ = 9.1 Hz, $^3J_{1,2}$ = 3.7 Hz, H-2), 3.62 (t, 1H, $^3J_{3',4'}$ = $^3J_{4',5'}$ = 9.5 Hz, H-4′), 2.85-2.60 (m, 4H, 2xCH_2, Lev), 2.18 (s, 3H, CH_3, Lev), 1.93 (d, 1H, C2-OH), 0.89 (s, 9H, [CH_3]$_3$C, TBDMS), 0.18, 0.14 (2×s, 6H, 2×CH_3, TBDMS); ^{13}C NMR (151 MHz, $CDCl_3$): δ [ppm] = 206.66 (*C*H_3C=O, Lev), 172.23 (C=O, Lev), 154.61 (C=O, Alloc), 153.31 (C=O, Troc), 136.92 (C_q, Ph), 131.45 (*C*H, Alloc), 129.27, 128.33, 126.28 (CH, Ph), 119.08 (*C*H_2, Alloc), 102.01 (Ph*C*H), 94.49 (C-1), 93.13 (C-1′), 81.04 (C-4′), 79.21 (*C*H_2, Troc), 76.24 (C-4), 72.90 (C-2), 72.64 (C-2′), 72.24 (C-3), 68.81 (C-3′), 68.76 (CH_2, Alloc), 68.70 (C-6′), 68.21 (C-5), 65.91 (C-6), 62.97 (C-5′), 37.99 (CH_2, Lev), 29.70 (CH_3, Lev), 27.89 (CH_2, Lev), 27.88 ([*C*H_3]$_3$C], TBDMS), 18.07 ([CH_3]$_3$*C*, TBDMS), -4.23, -4.77 (2xCH_3, TBDMS); HRMS ($^+$ESI) m/z: calcd for $C_{37}H_{55}Cl_3NO_{17}Si$ $[M+NH_4]^+$ 920.2280, found 920.2281.

1 ^{1}H-NMR (600 MHz, MeOD)

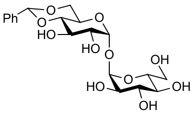

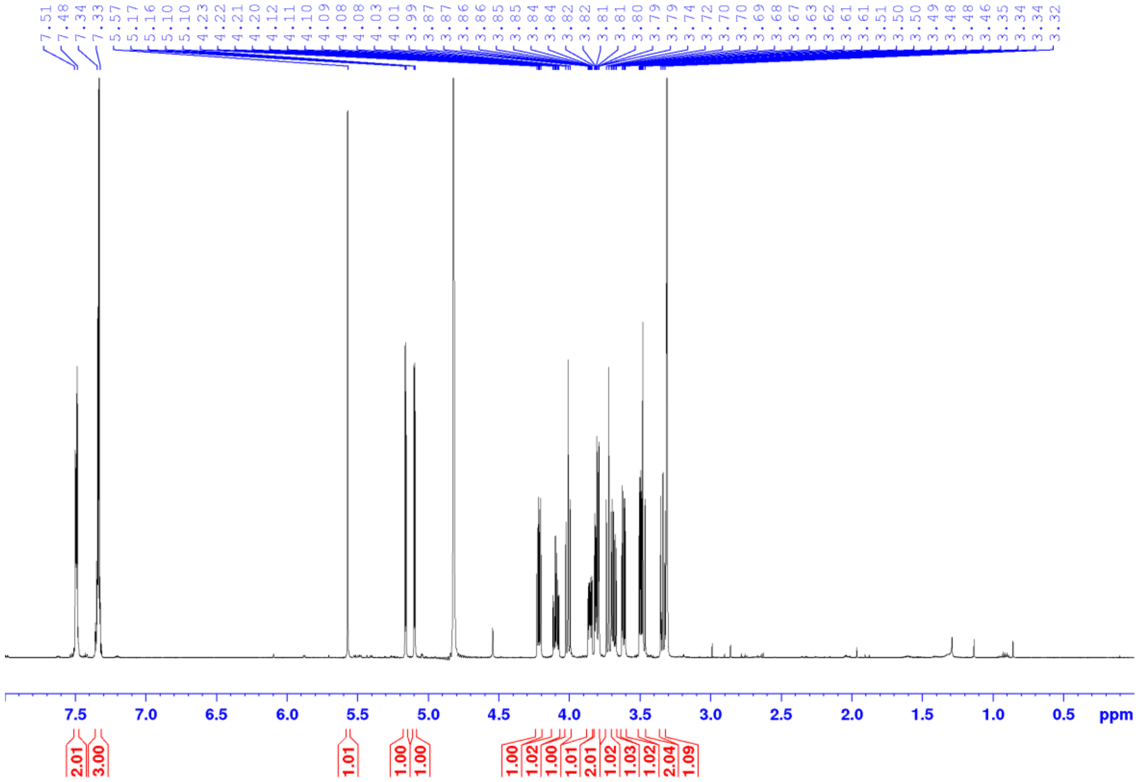

1 ^{13}C-NMR (151 MHz, MeOD)

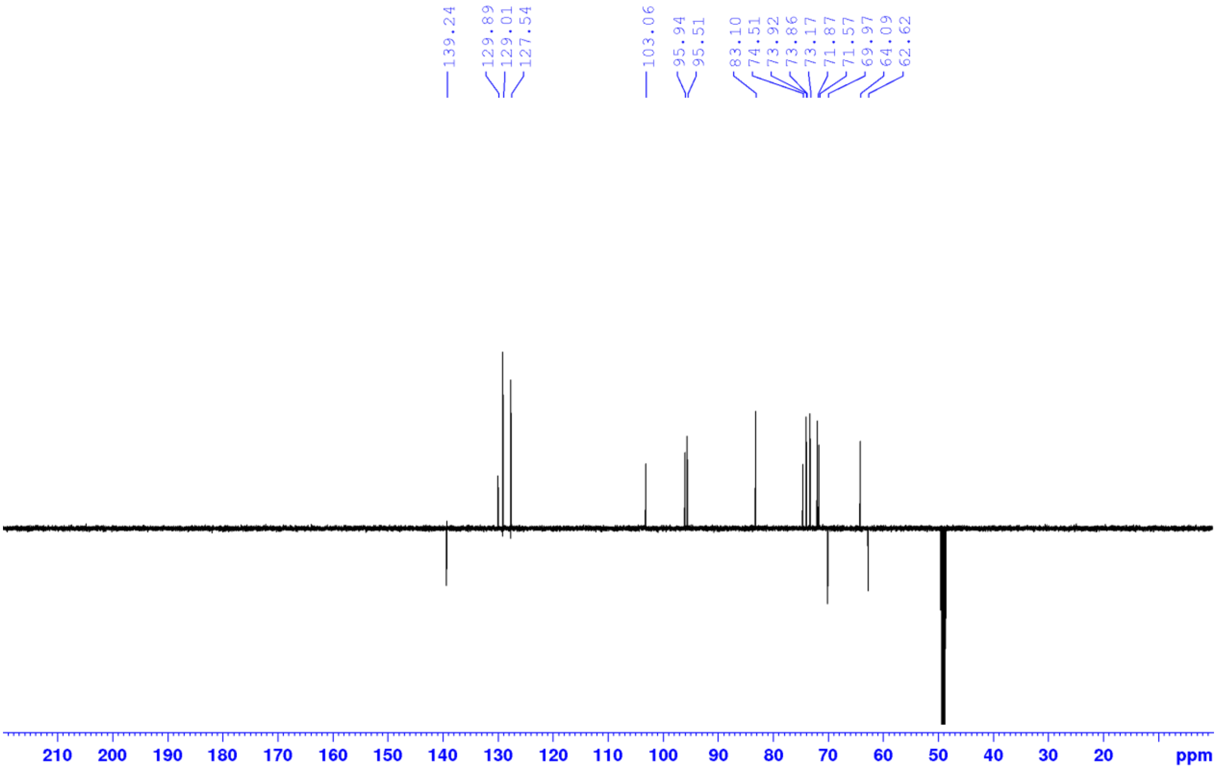

2 ^{1}H-NMR (600 MHz, MeOD)

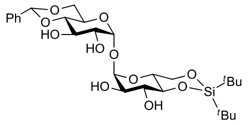

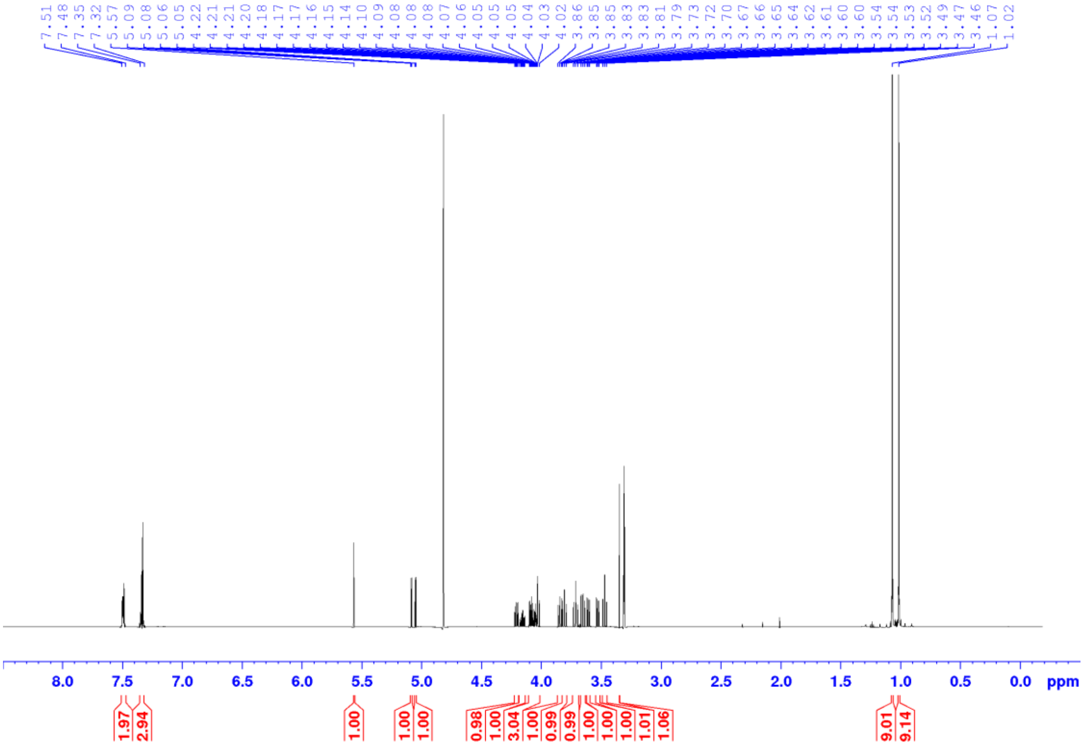

2 ^{13}C-NMR (151 MHz, MeOD)

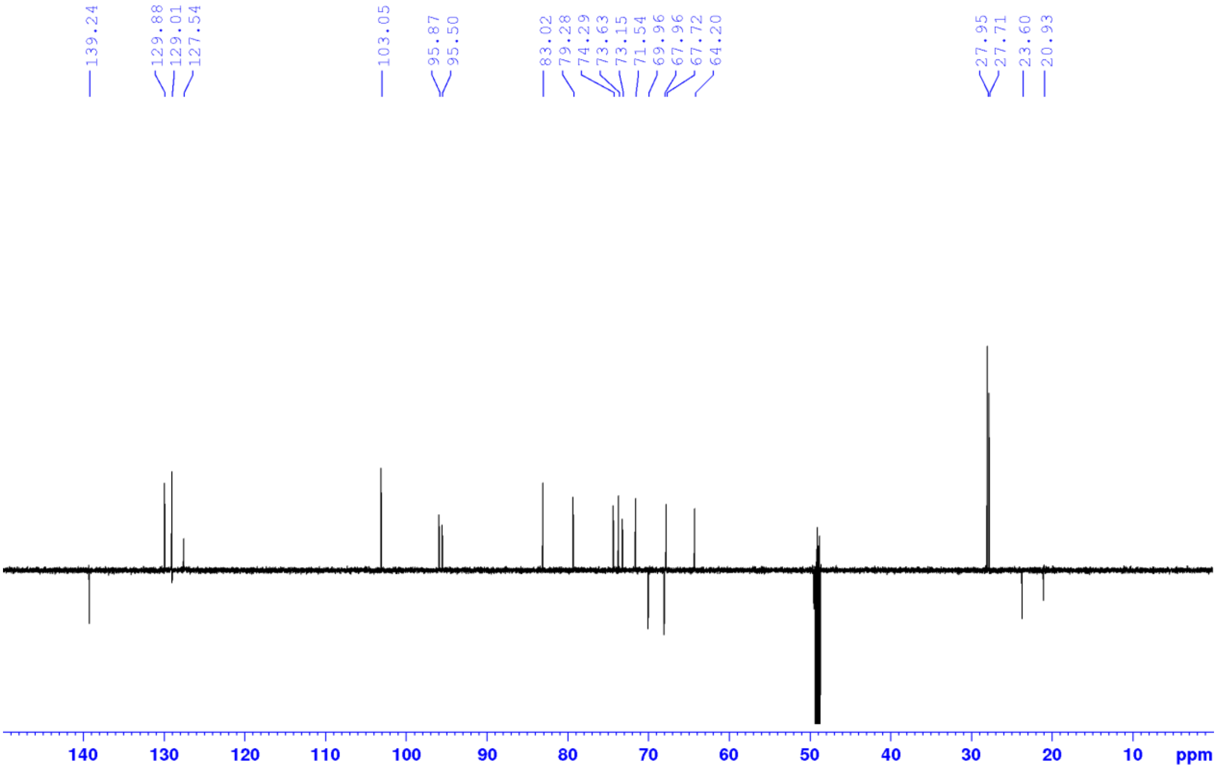

3 ^{1}H-NMR (600 MHz, $CDCl_3$)

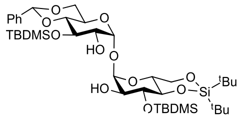

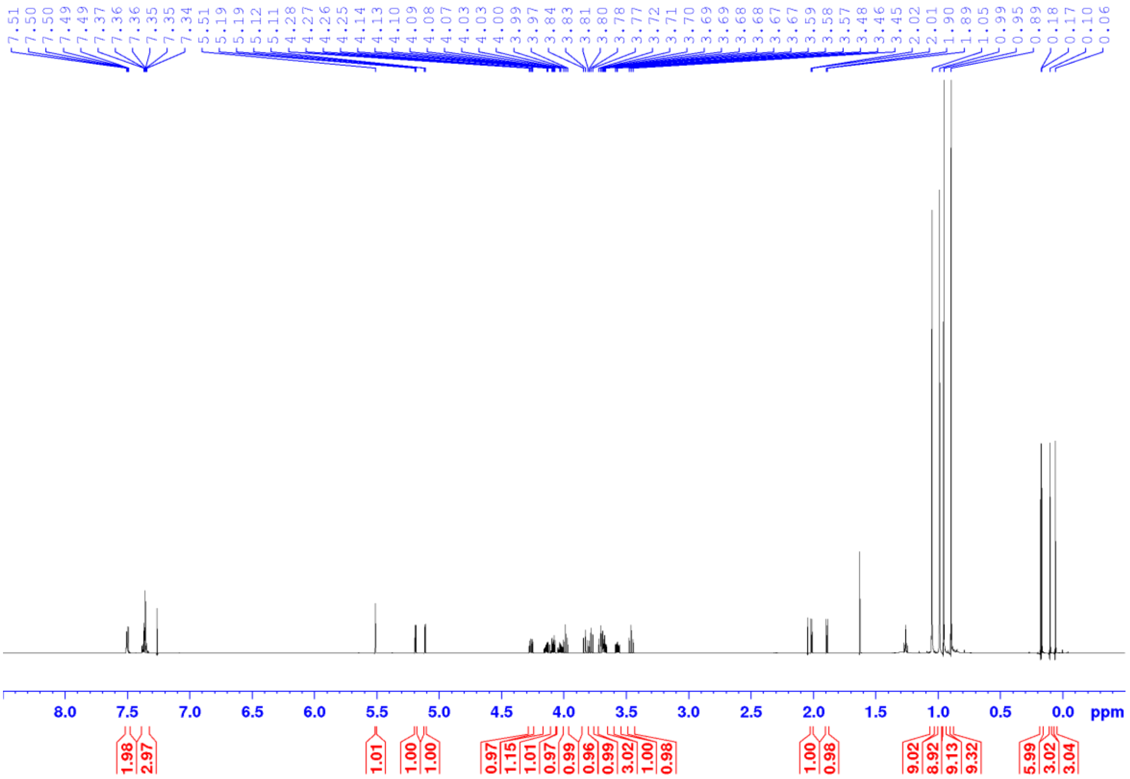

3 ^{13}C NMR (151 MHz, $CDCl_3$)

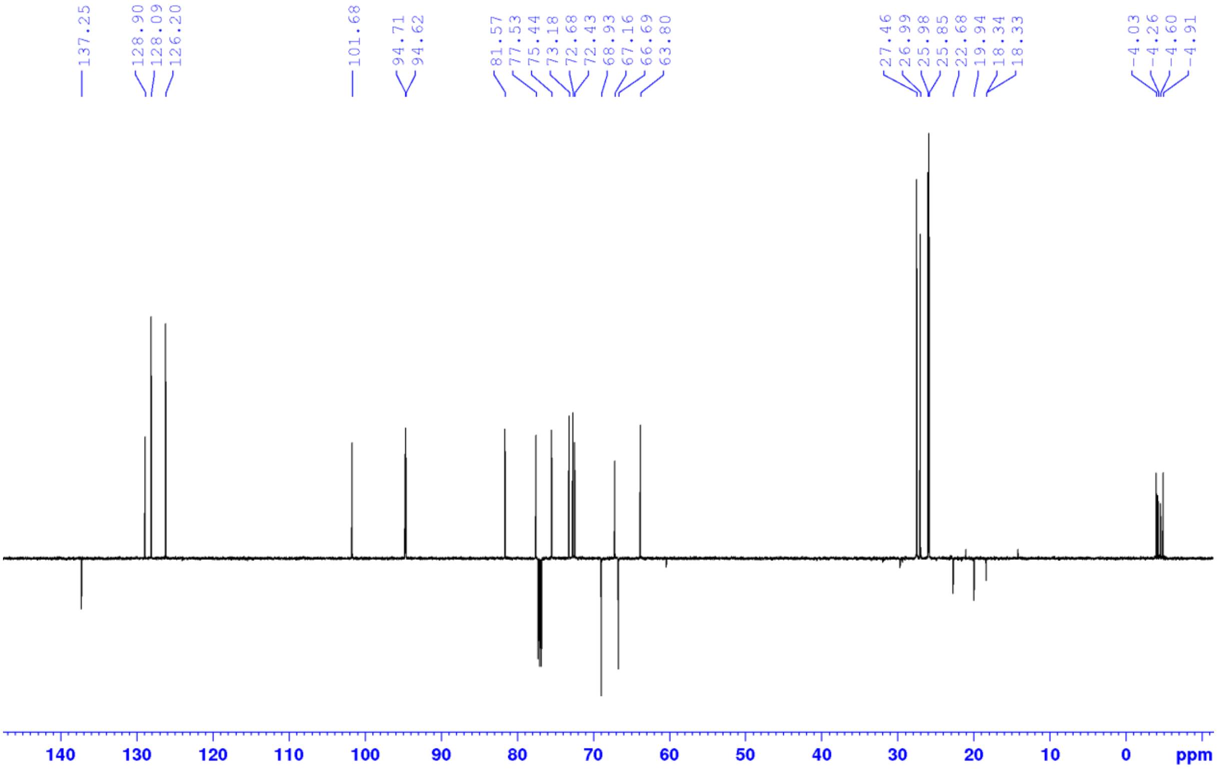

3 ^{1}H–^{29}Si HMBC ($CDCl_3$)

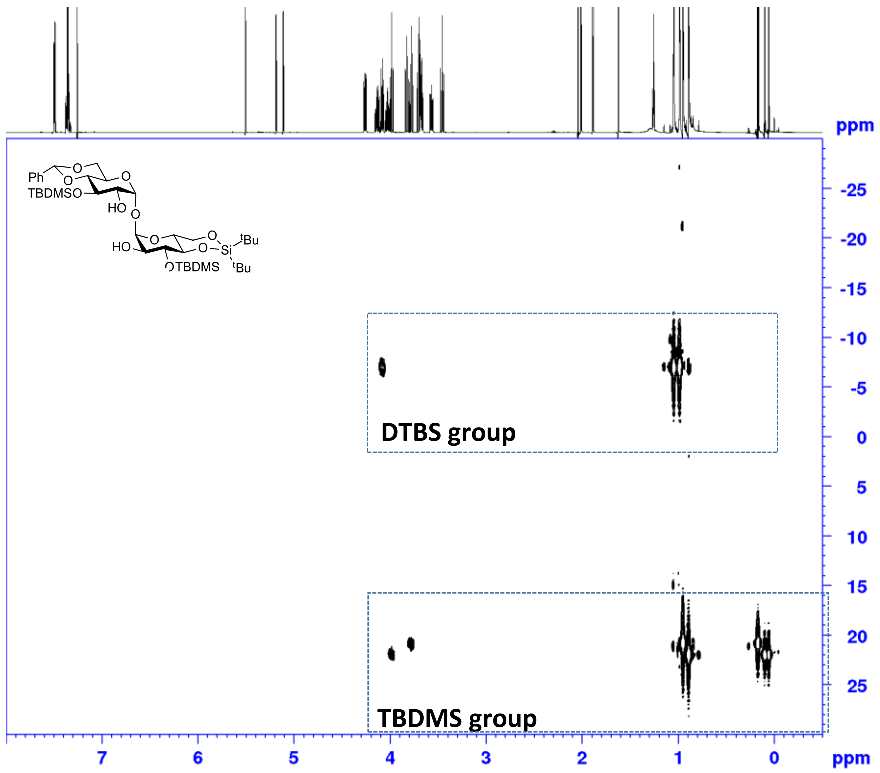

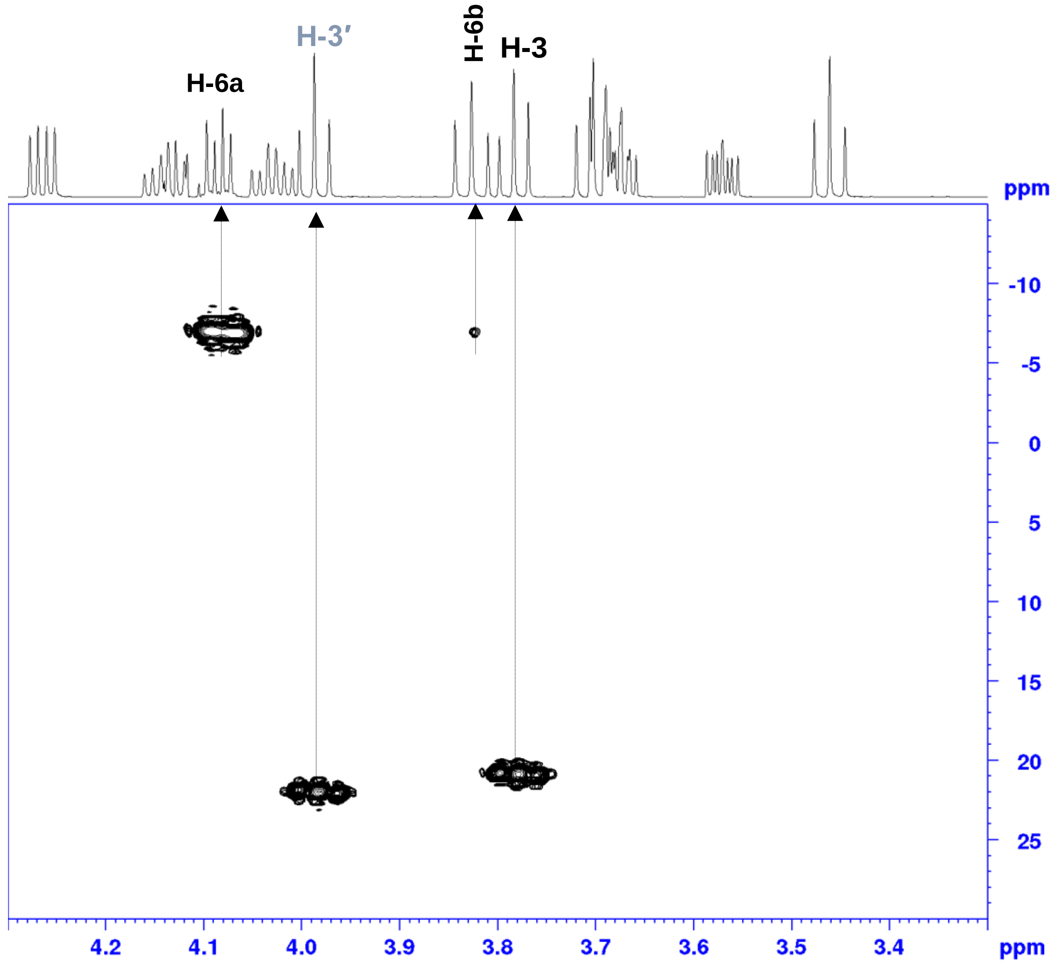

4 + 5 (mixture of regioisomers) ^{1}H-NMR (600 MHz, $CDCl_3$)

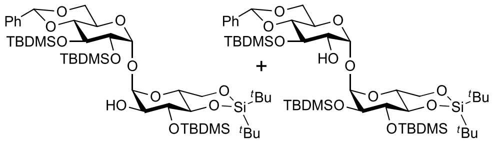

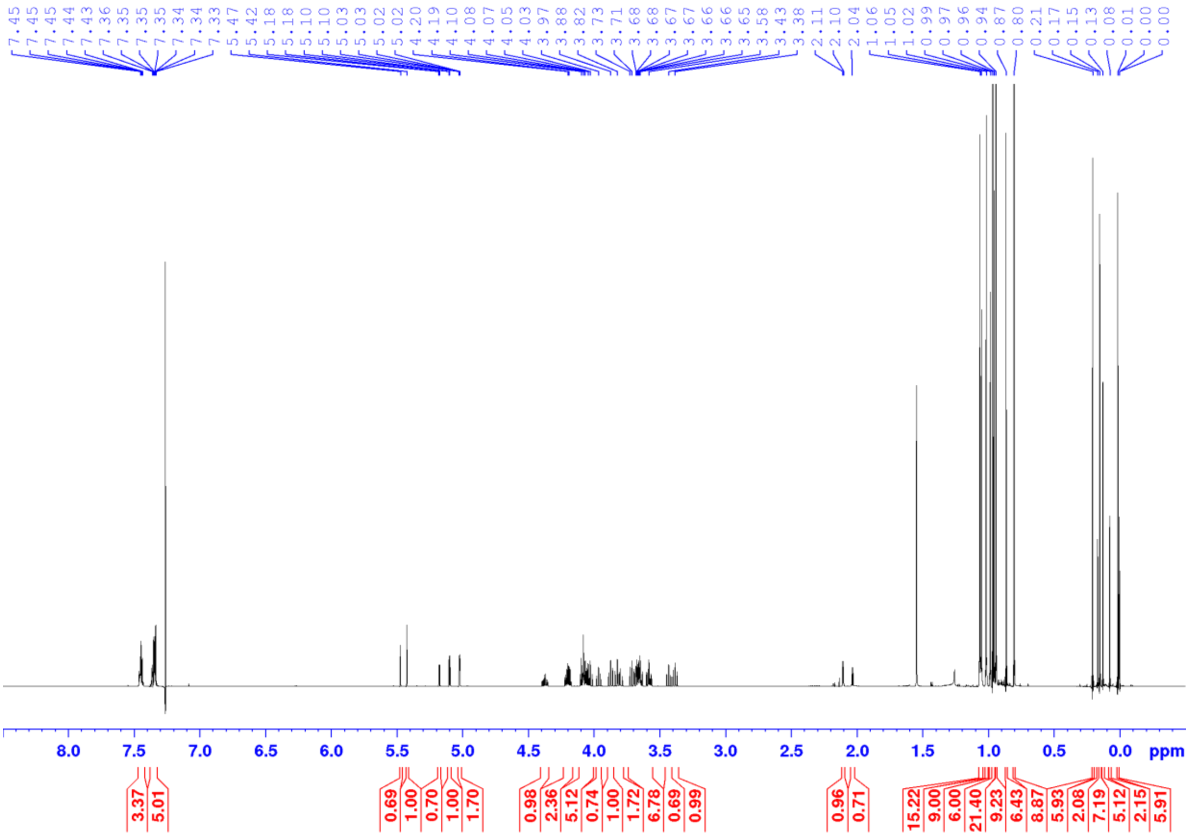

4 + 5 (mixture of regioisomers) ^{13}C-NMR (151 MHz, $CDCl_3$)

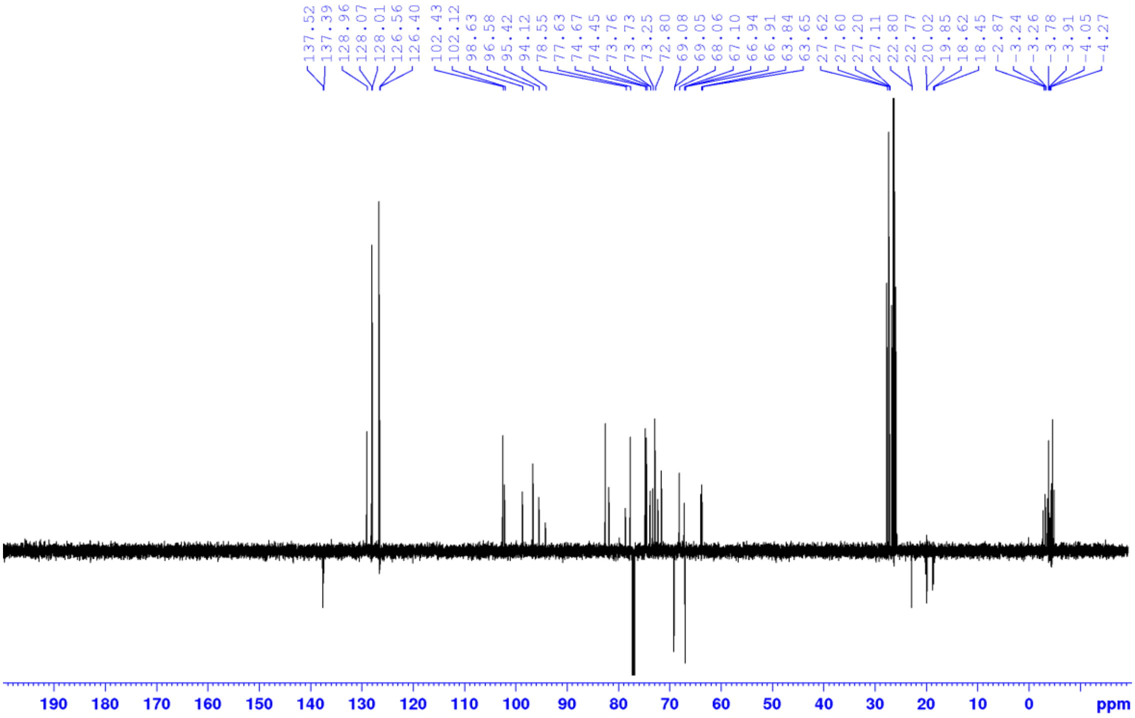

4 + 5 (mixture of regioisomers) ^{1}H–^{29}Si HMBC ($CDCl_3$)

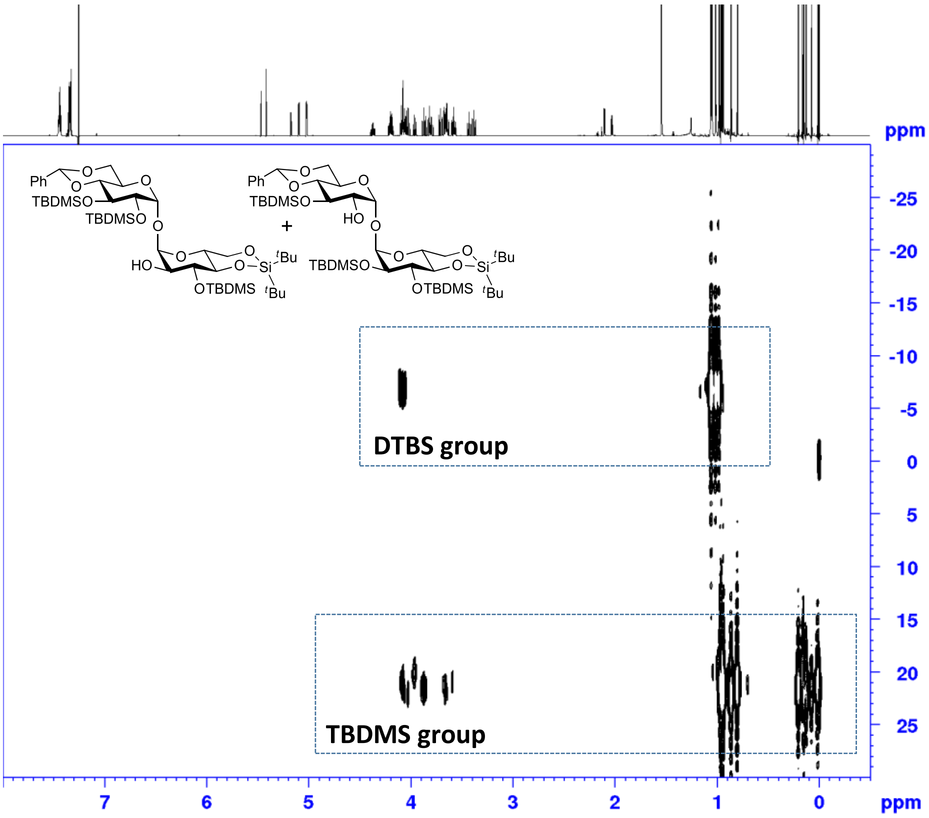

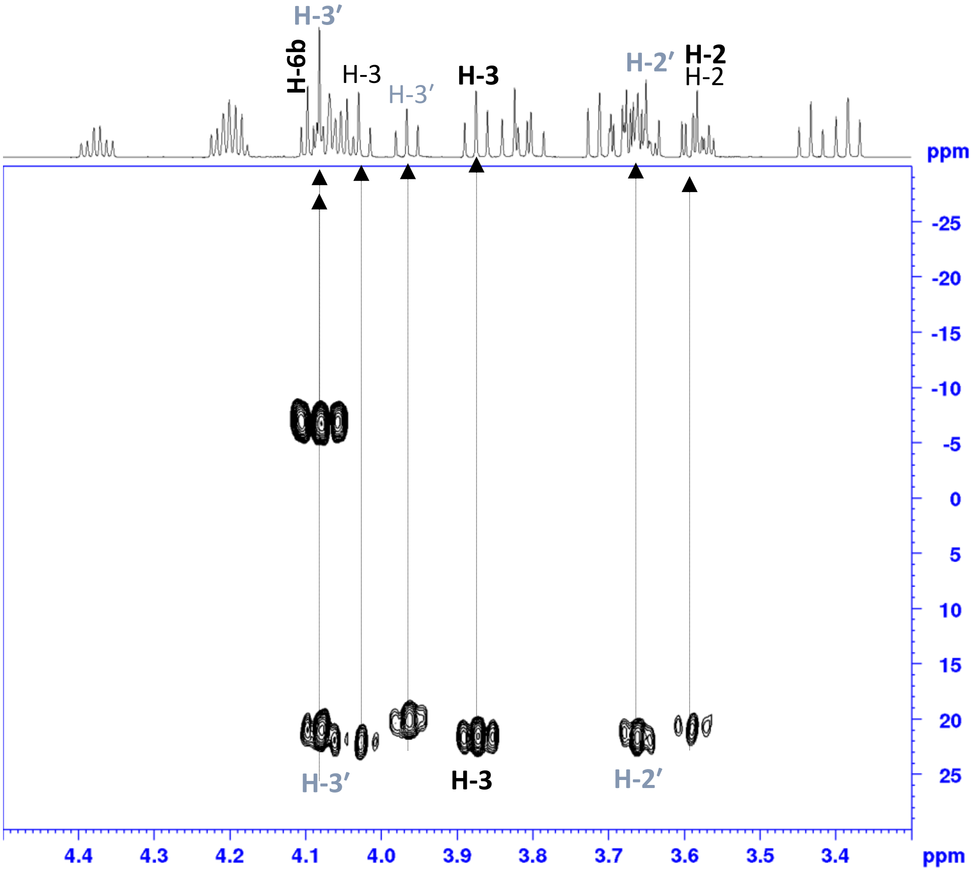

6 + 7 (mixture of regioisomers) ^{1}H-NMR (600 MHz, $CDCl_3$)

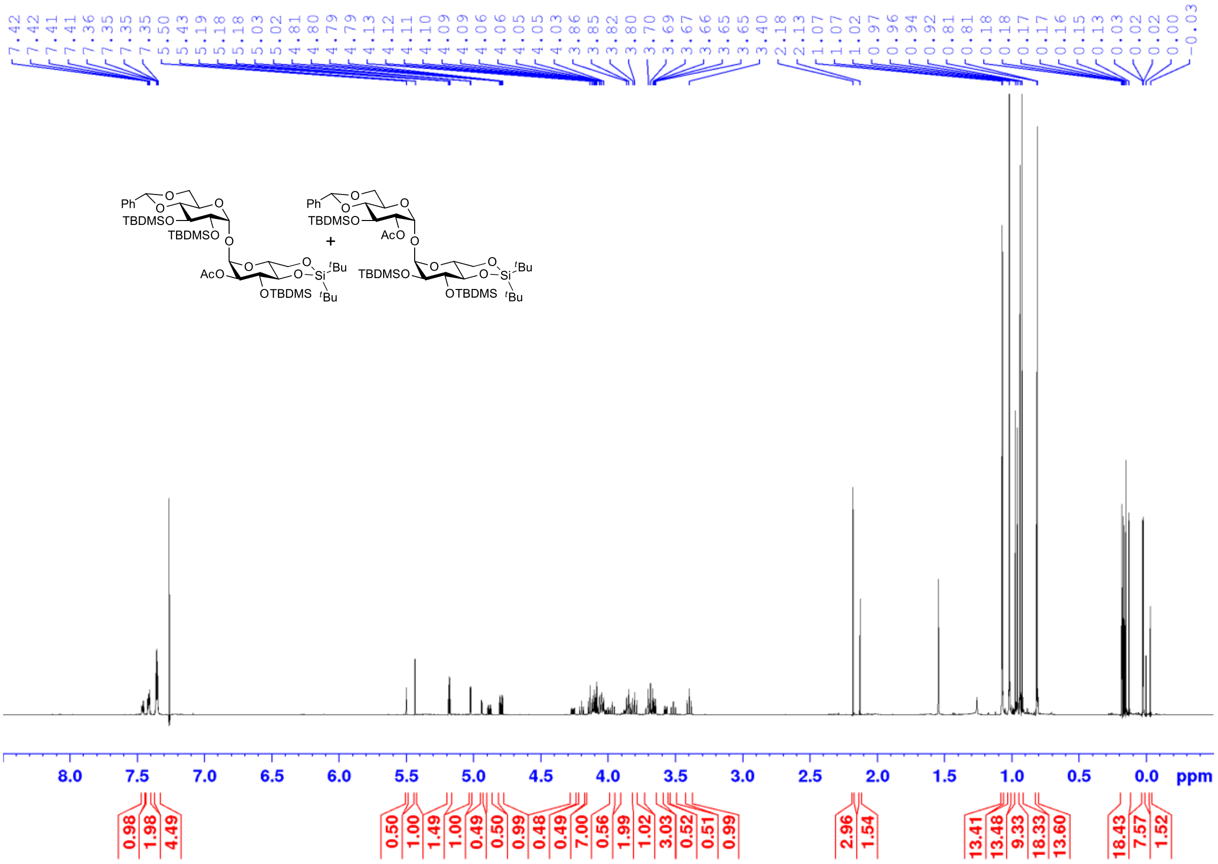

6 + 7 (mixture of regioisomers) ^{13}C NMR (151 MHz, $CDCl_3$)

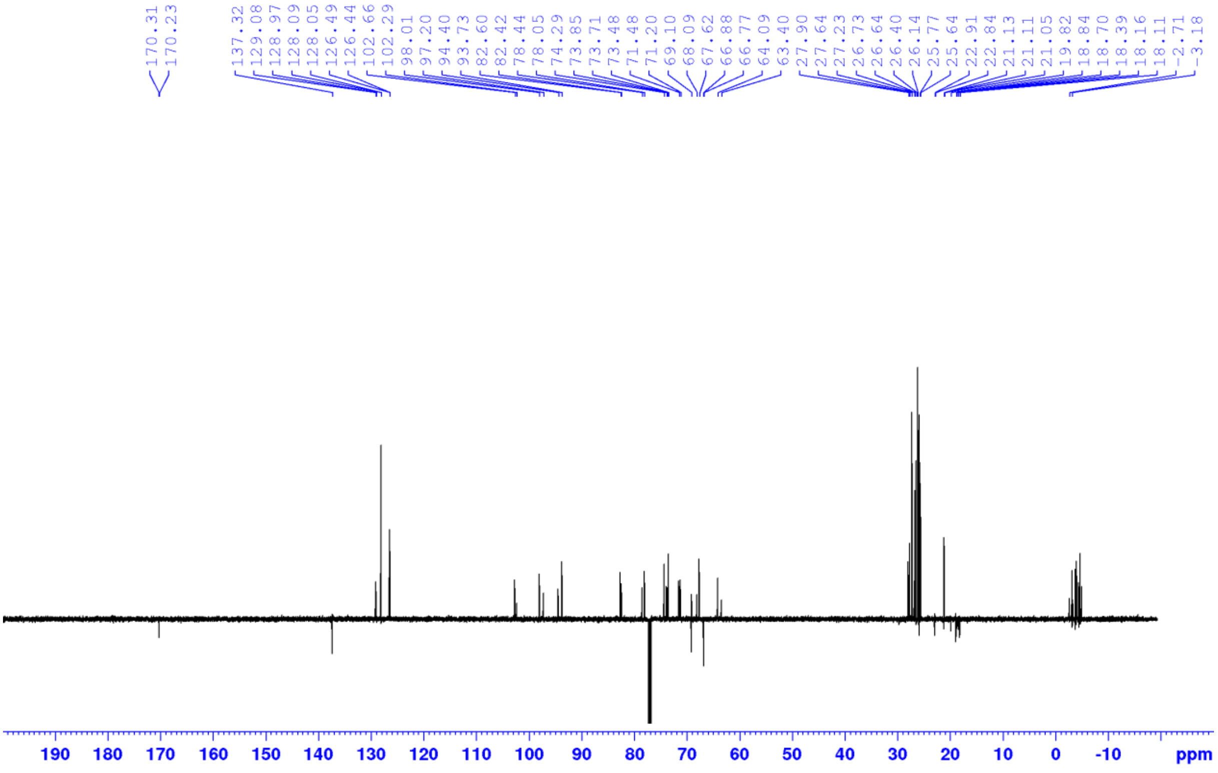

6 + **7** (mixture of regioisomers) ^{1}H–^{29}Si HMBC (CDCl$_3$) *

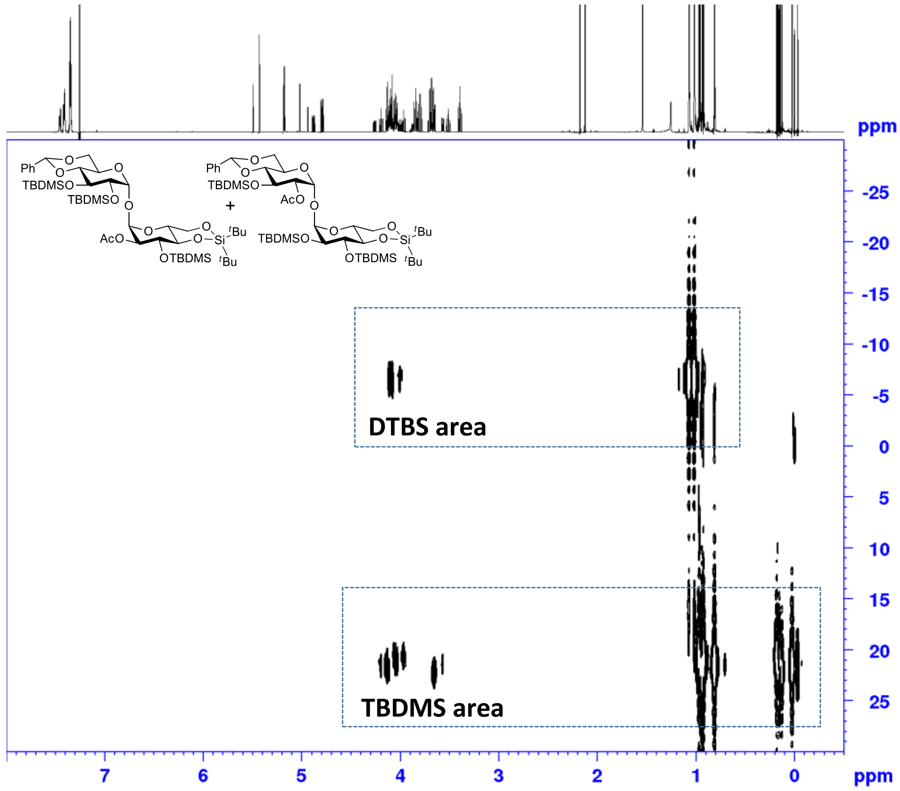

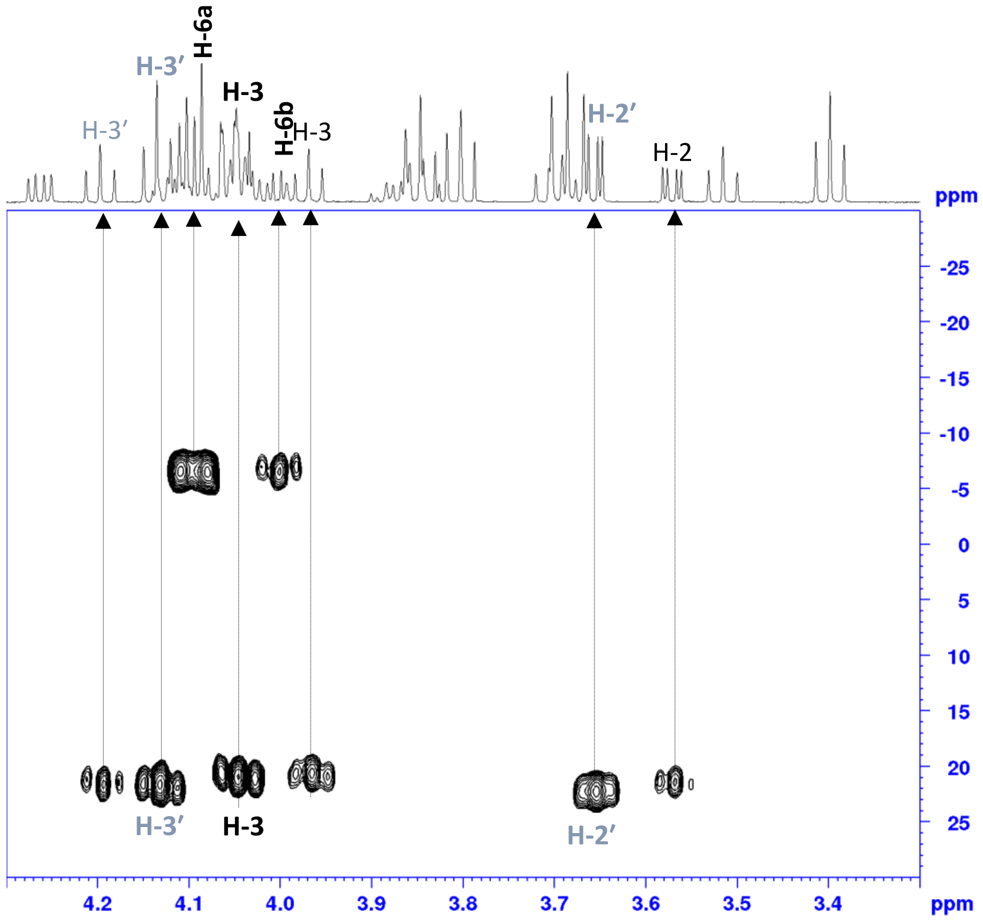

8 + 9 (mixture of regioisomers) ^{1}H-NMR (600 MHz, $CDCl_3$)

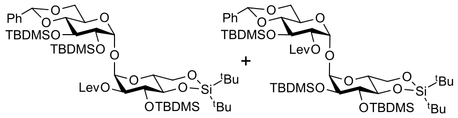

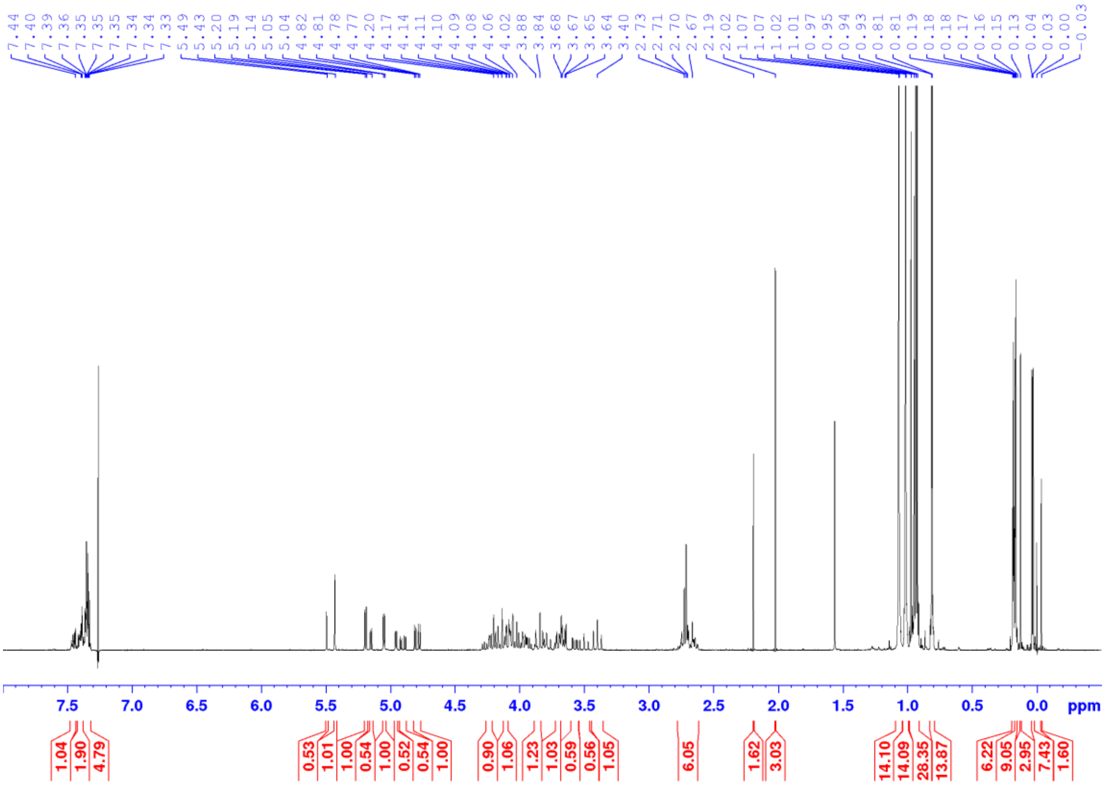

8 + 9 ^{13}C-NMR (151 MHz, $CDCl_3$)

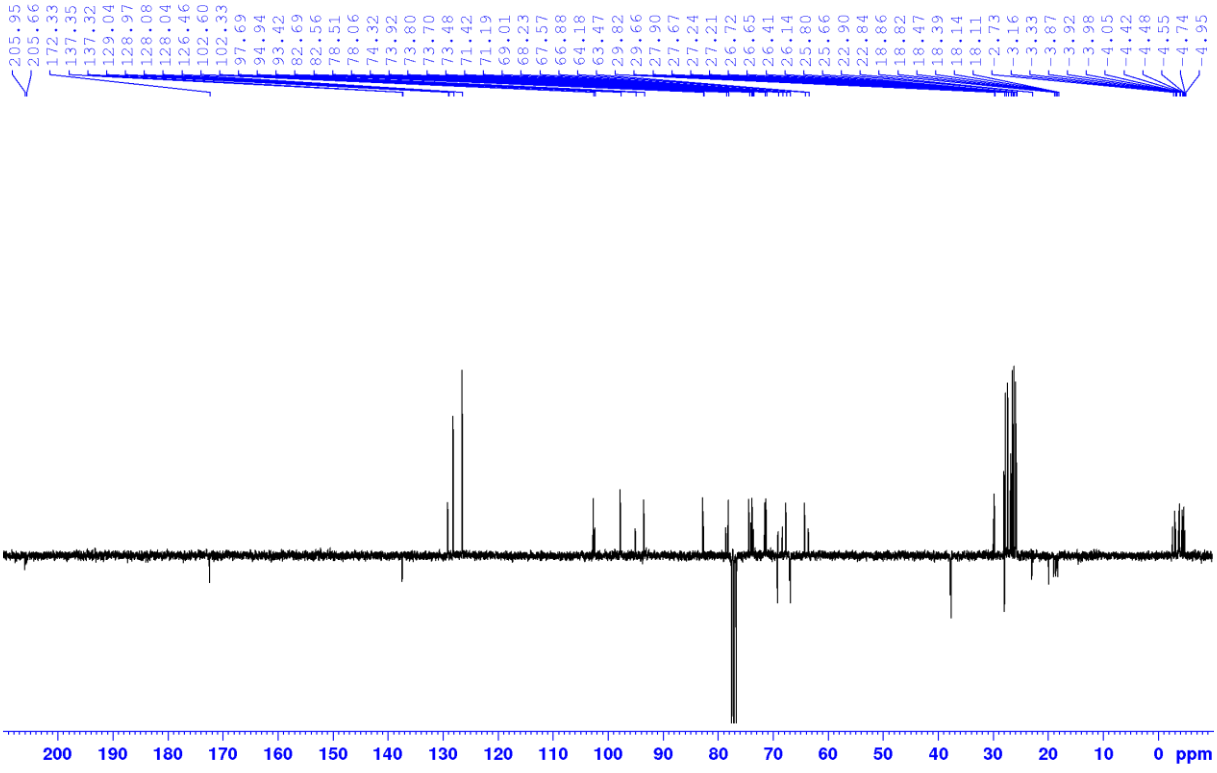

8+ 9 ^{1}H–^{29}Si HMBC ($CDCl_3$)

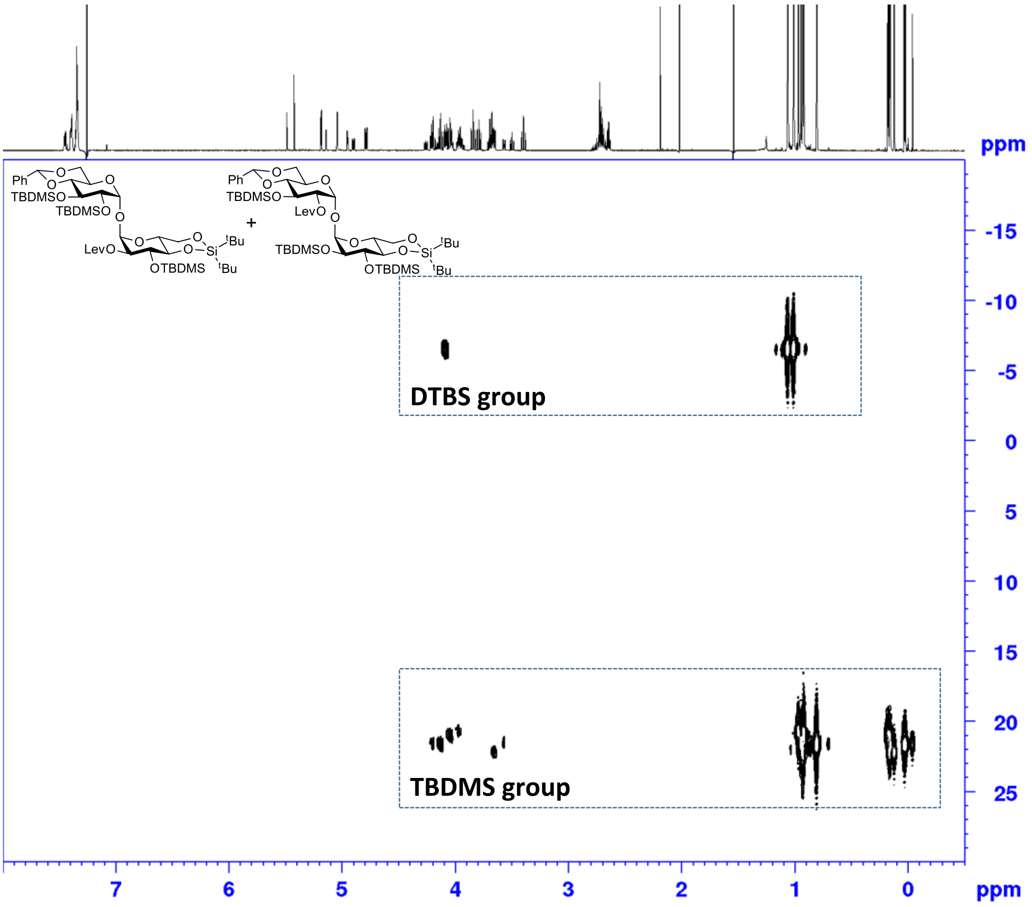

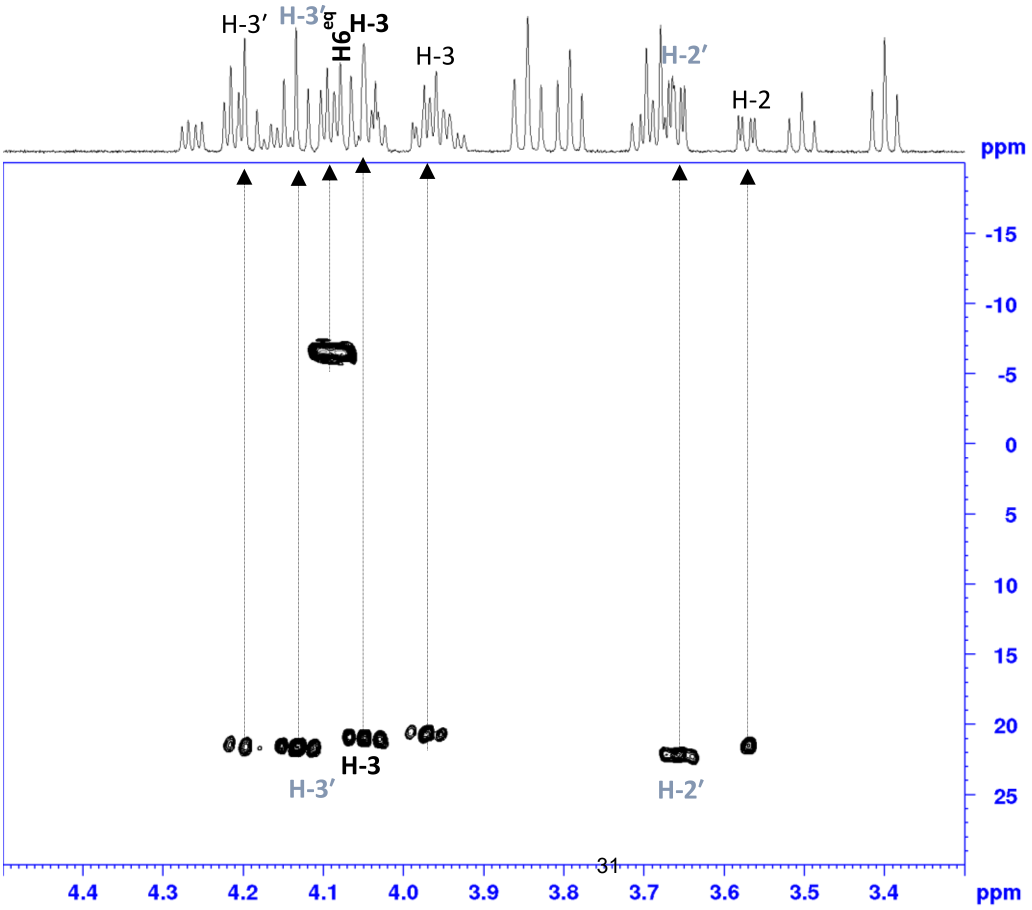

10 ^{1}H-NMR (600 MHz, $CDCl_3$)

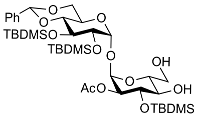

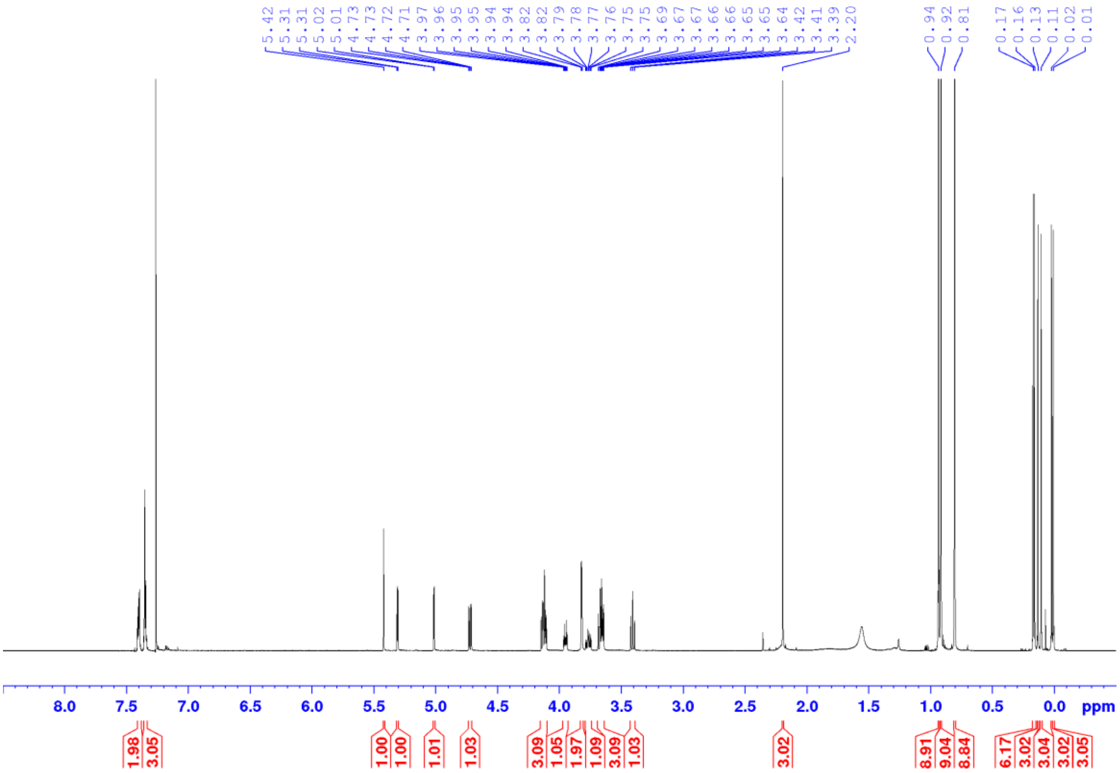

10 ^{13}C-NMR (151 MHz, $CDCl_3$)

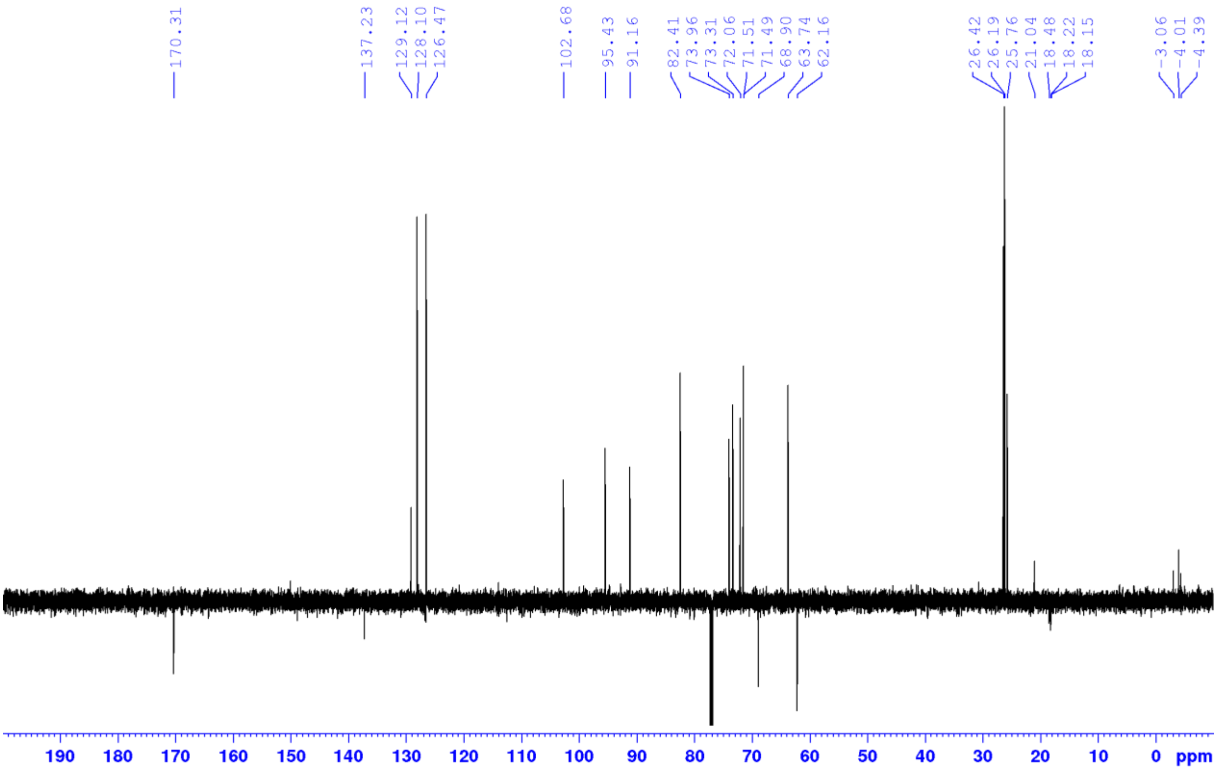

11 ^{1}H-NMR (600 MHz, $CDCl_3$)

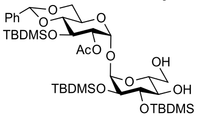

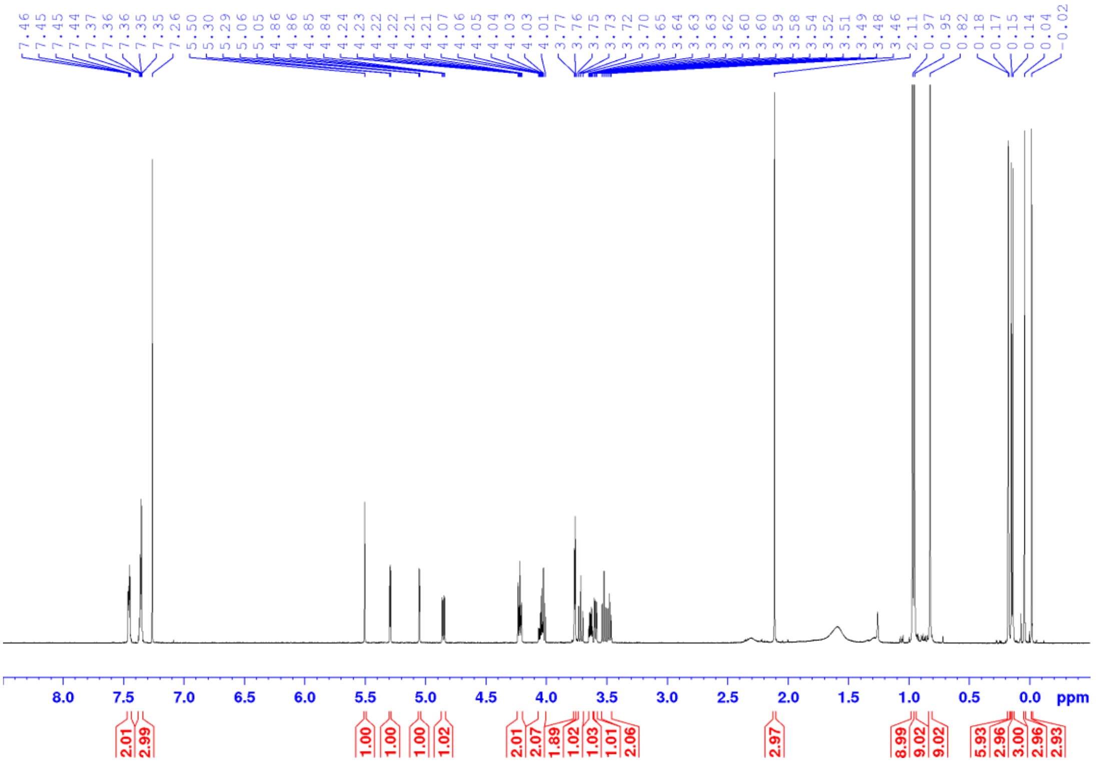

11 ^{13}C-NMR (151 MHz, $CDCl_3$)

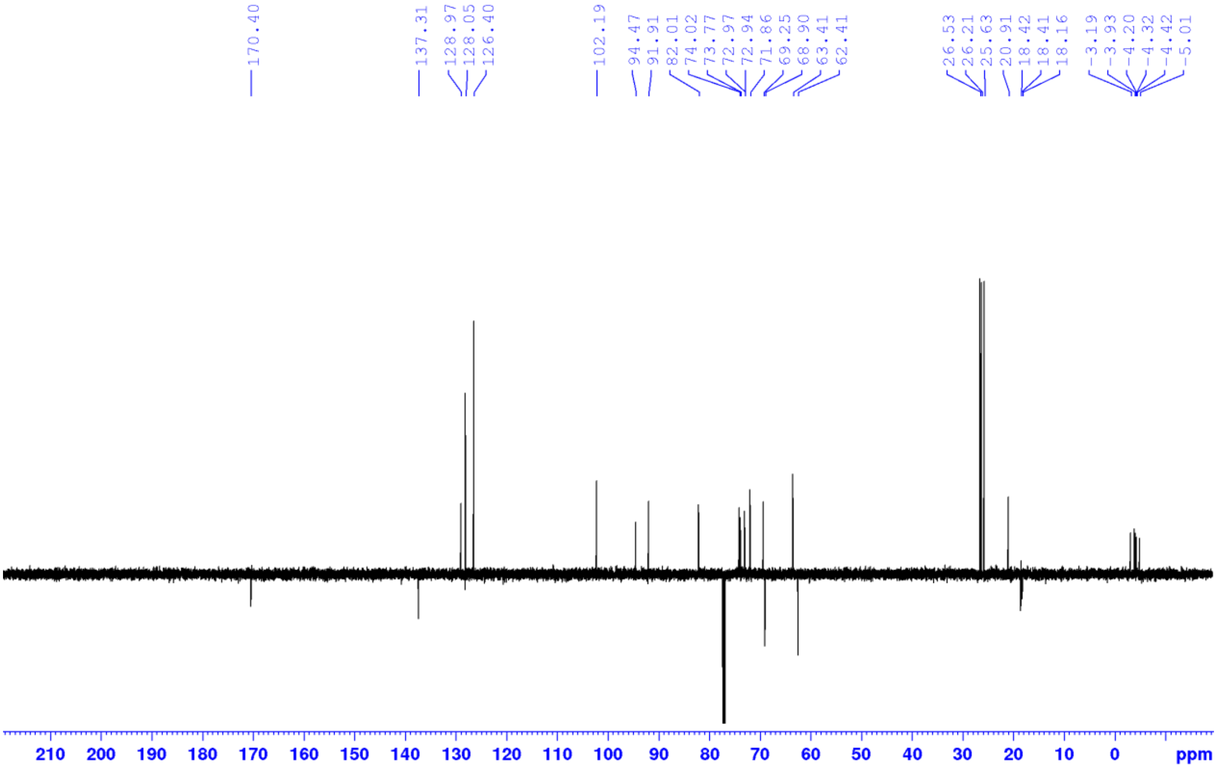

12 ^{1}H-NMR (600 MHz, $CDCl_3$)

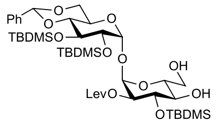

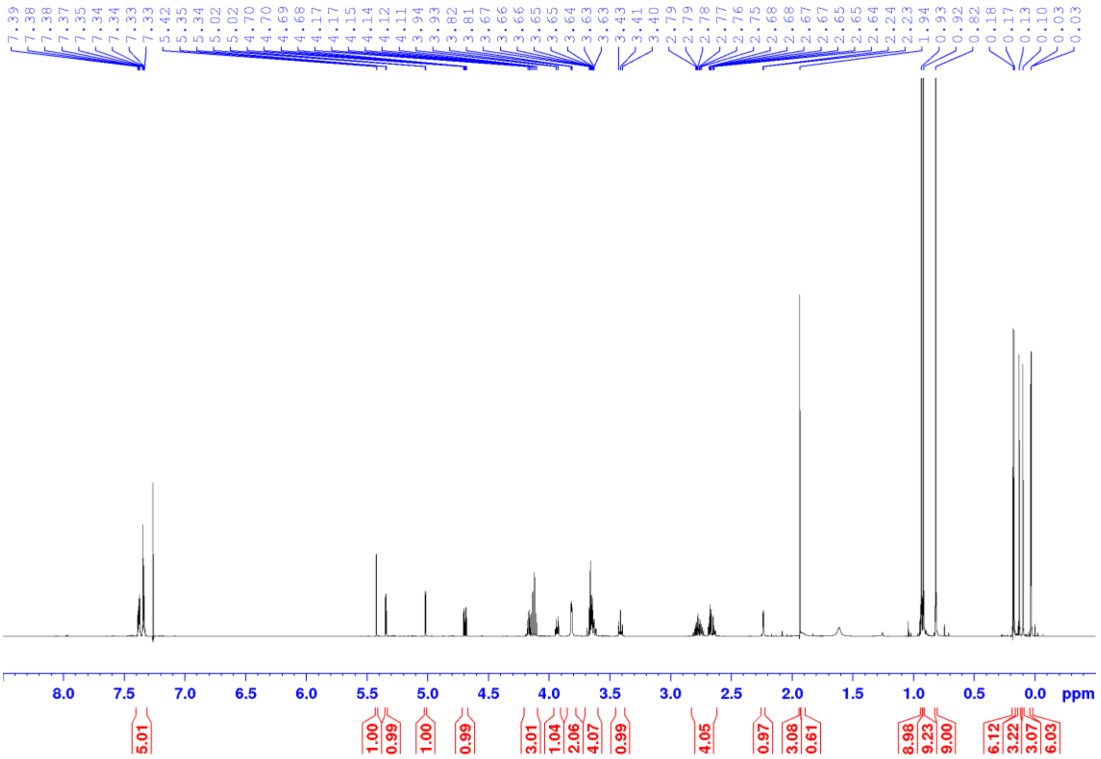

12 ^{13}C-NMR (151 MHz, $CDCl_3$)

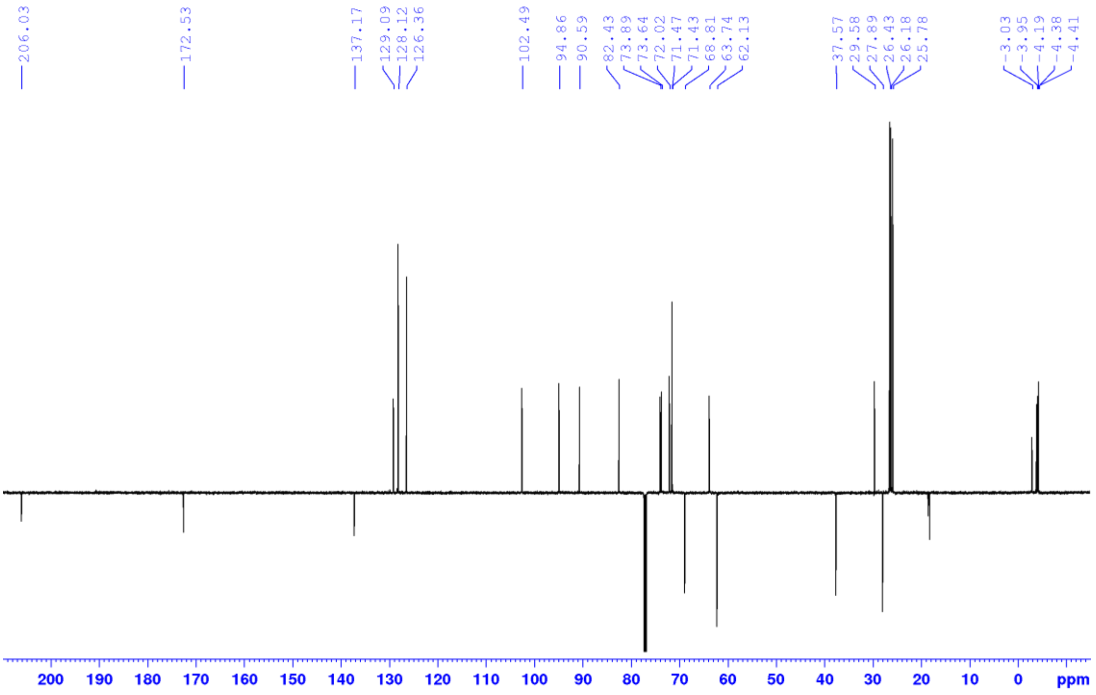

12 ^{1}H–^{29}Si HMBC ($CDCl_3$)

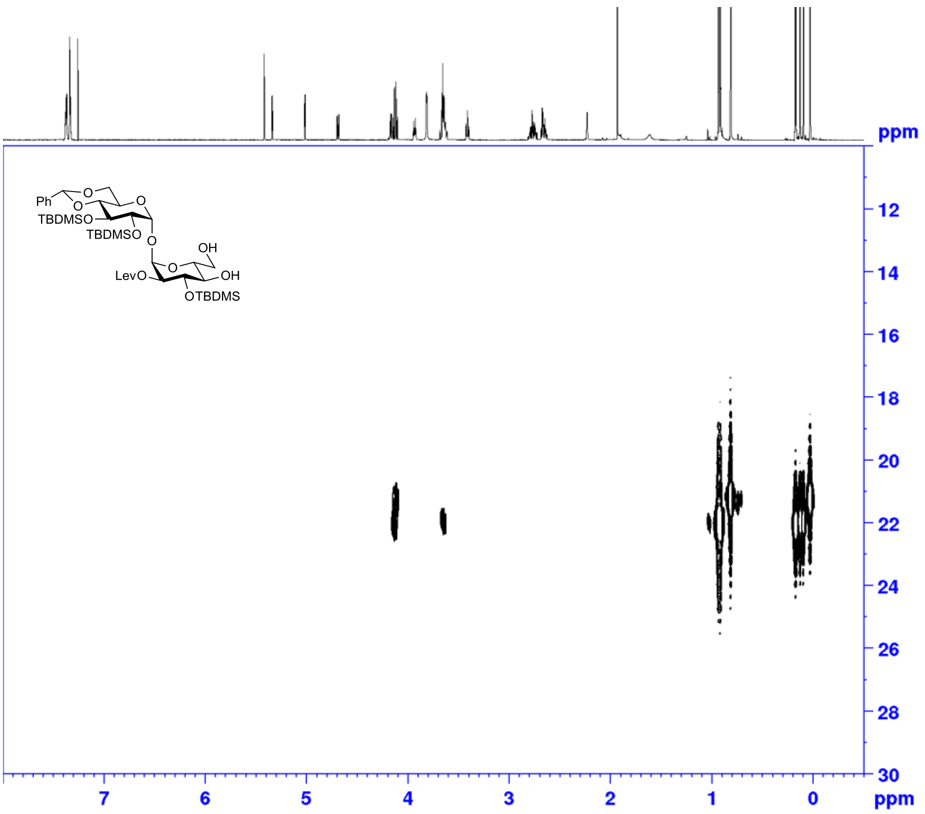

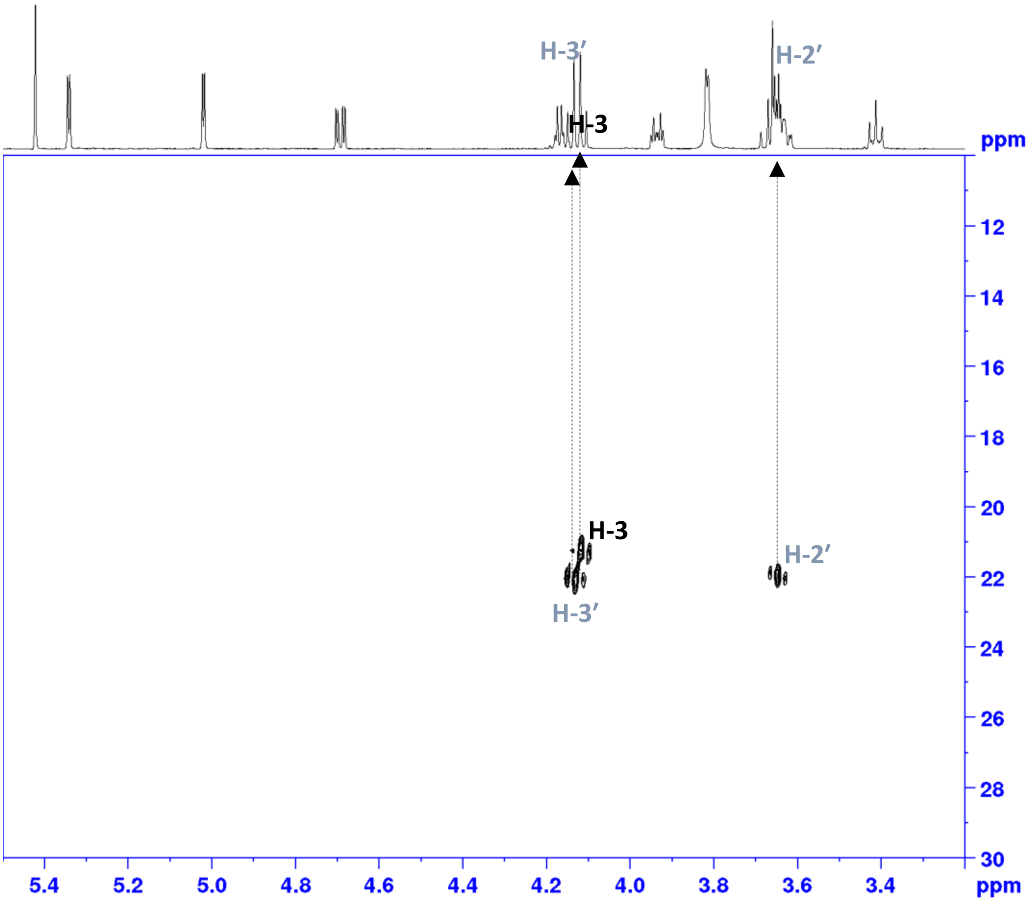

13 ^{1}H-NMR (600 MHz, $CDCl_3$)

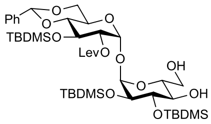

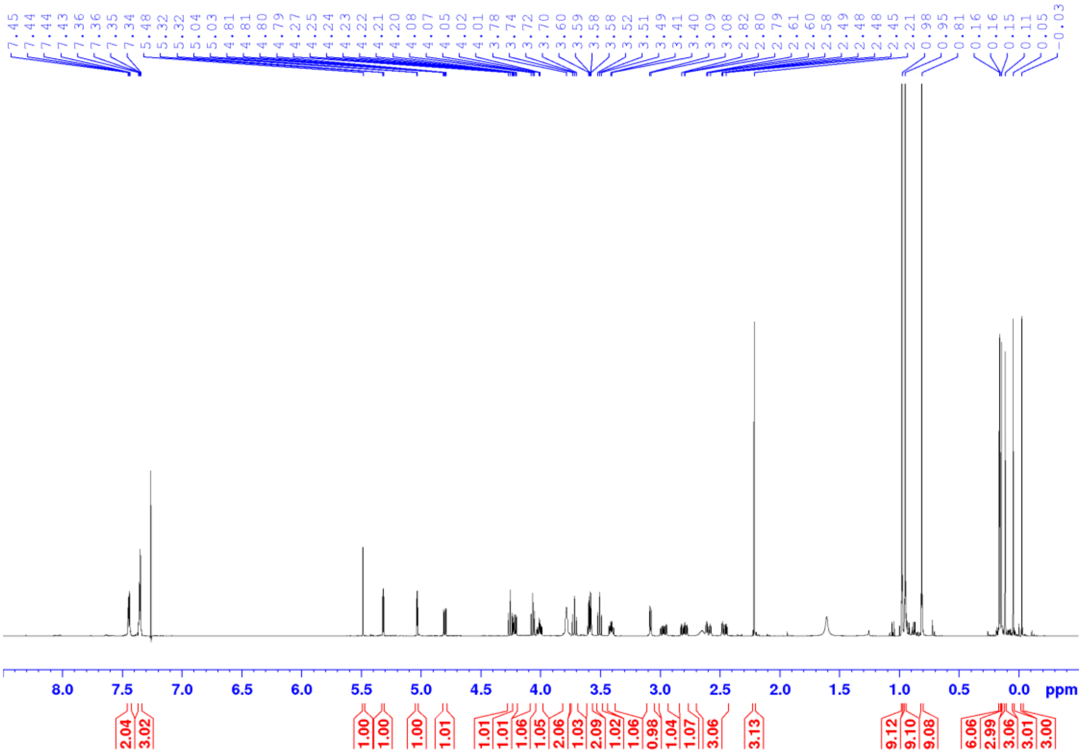

13 ^{13}C NMR (151 MHz, $CDCl_3$)

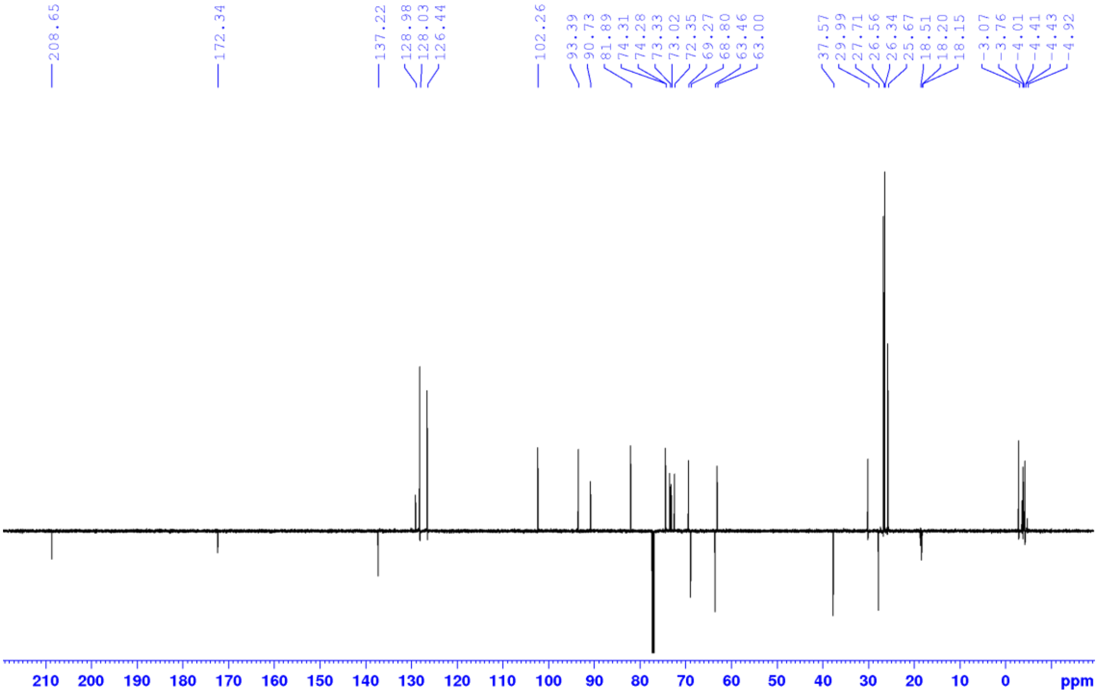

13 1H–29Si HMBC ($CDCl_3$)

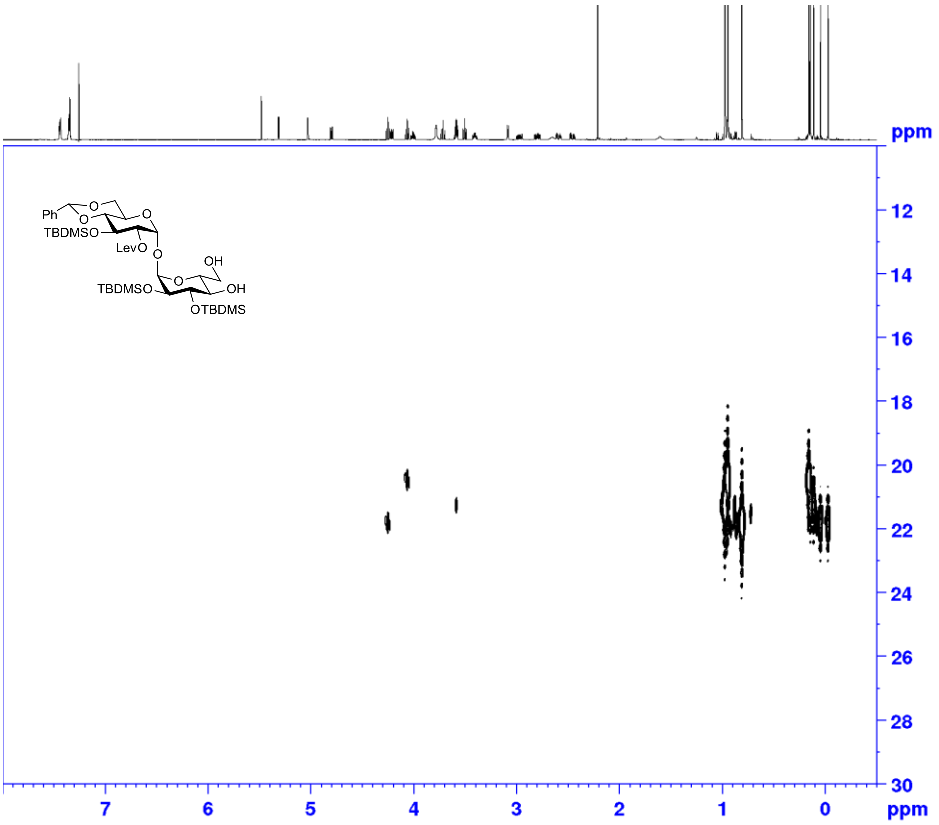

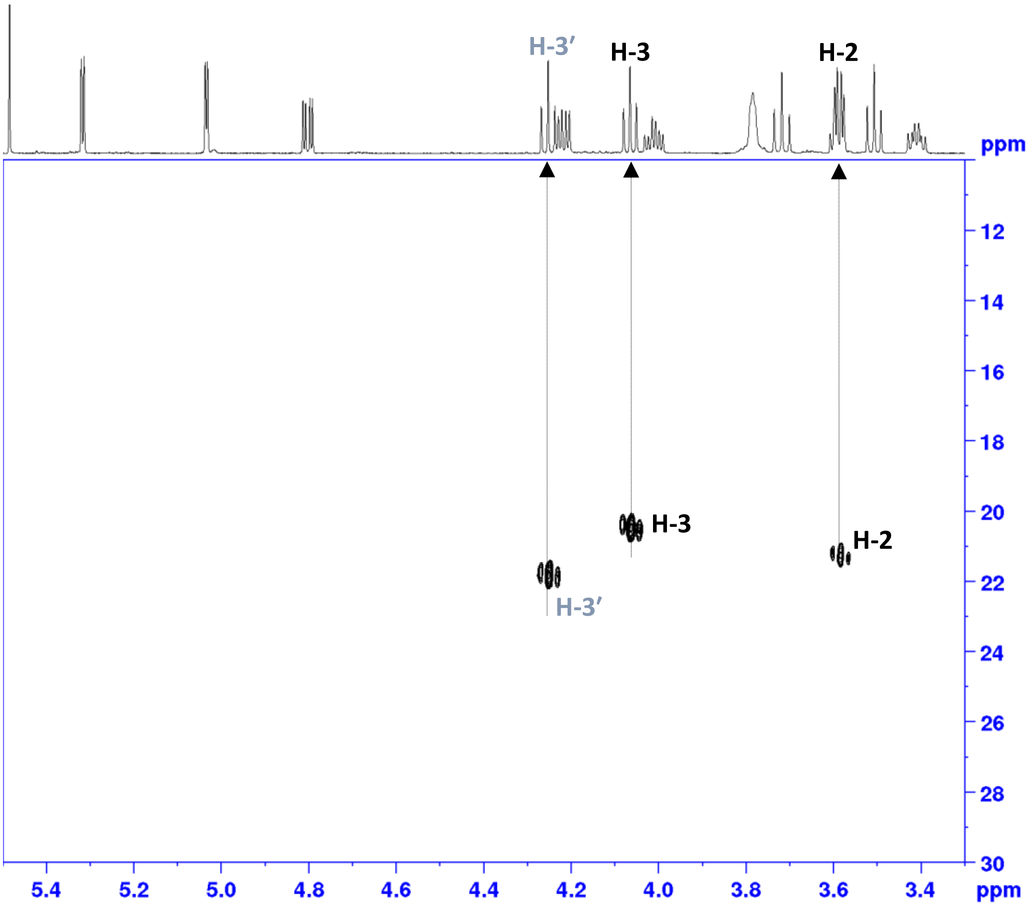

14 ^{1}H-NMR (600 MHz, $CDCl_3$)

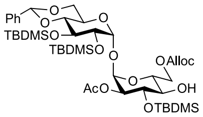

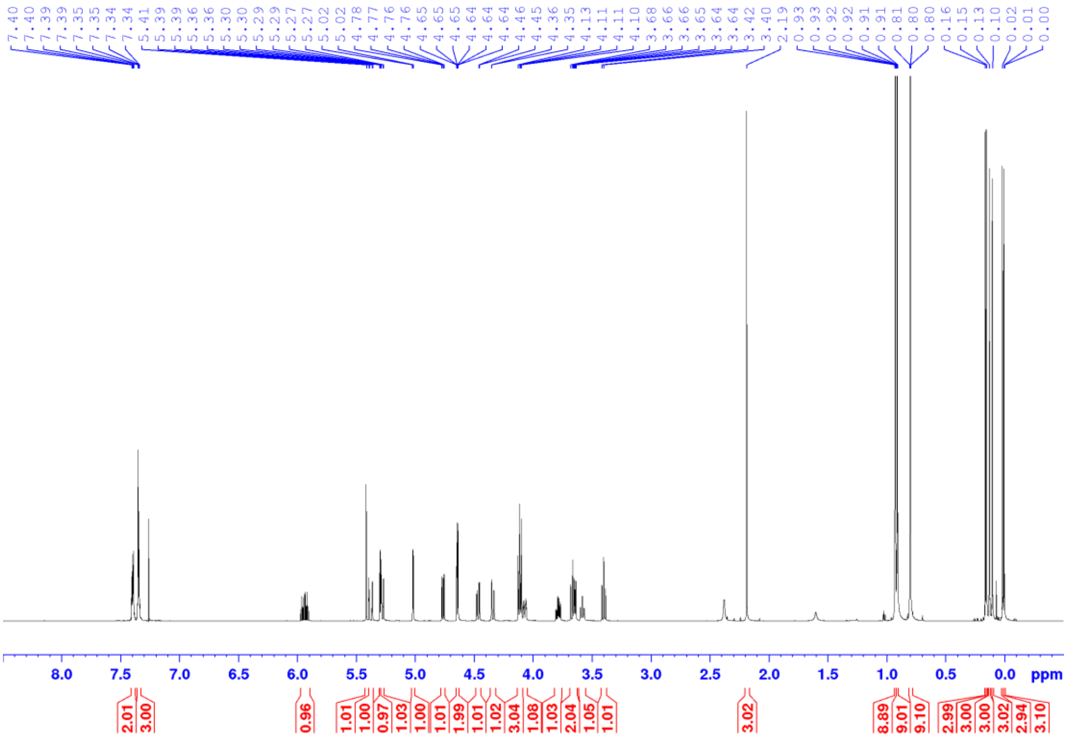

14 ^{13}C-NMR (151 MHz, $CDCl_3$)

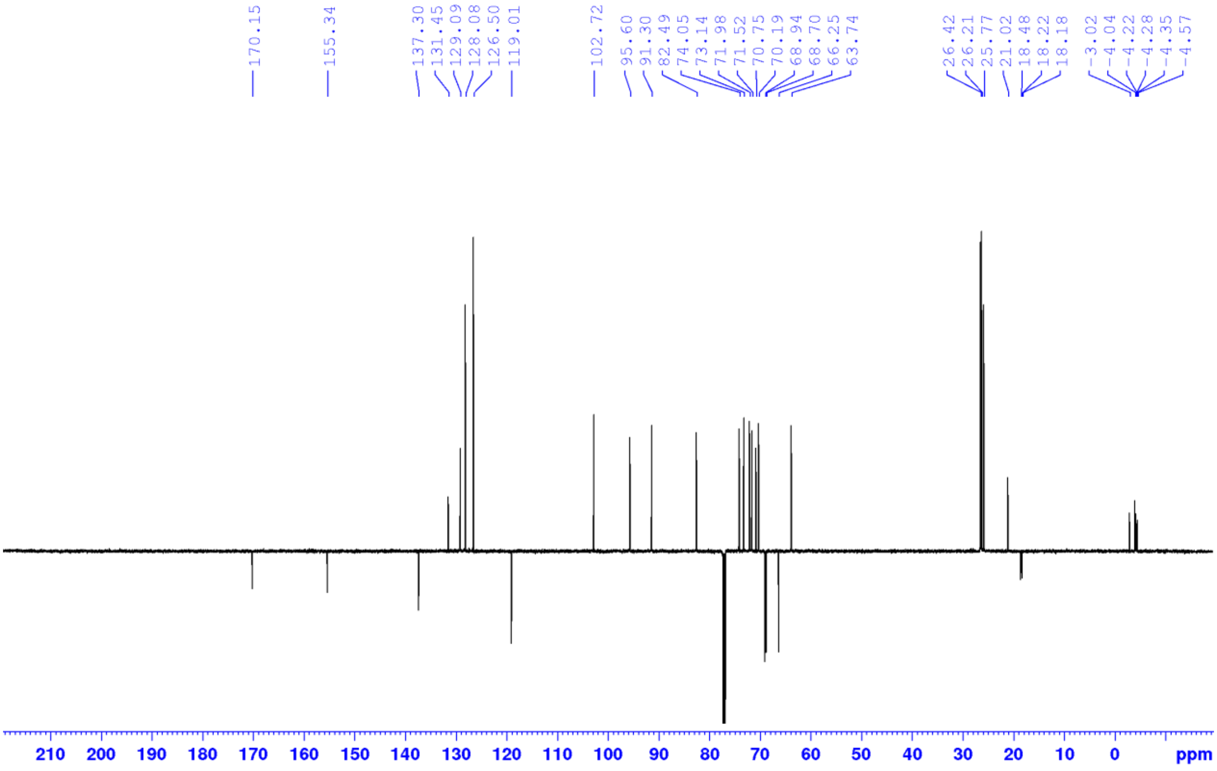

15 ^{1}H-NMR (600 MHz, $CDCl_3$)

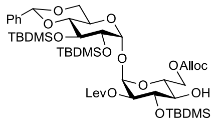

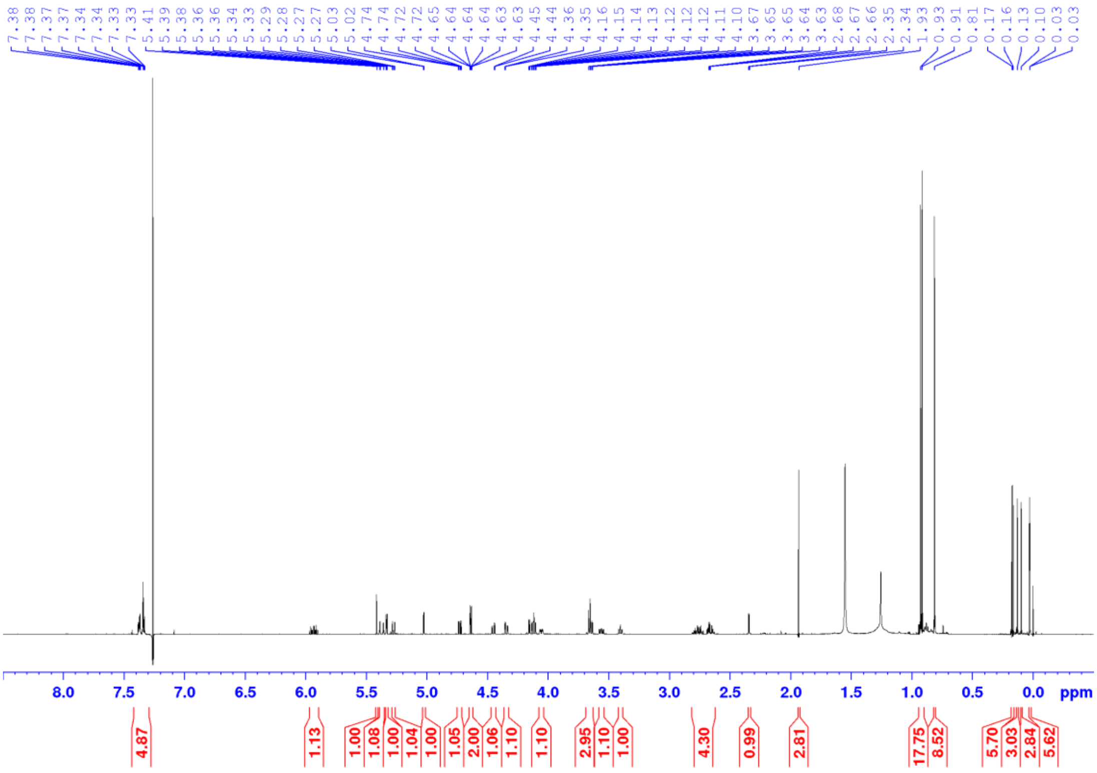

15 ^{13}C-NMR (151 MHz, $CDCl_3$)

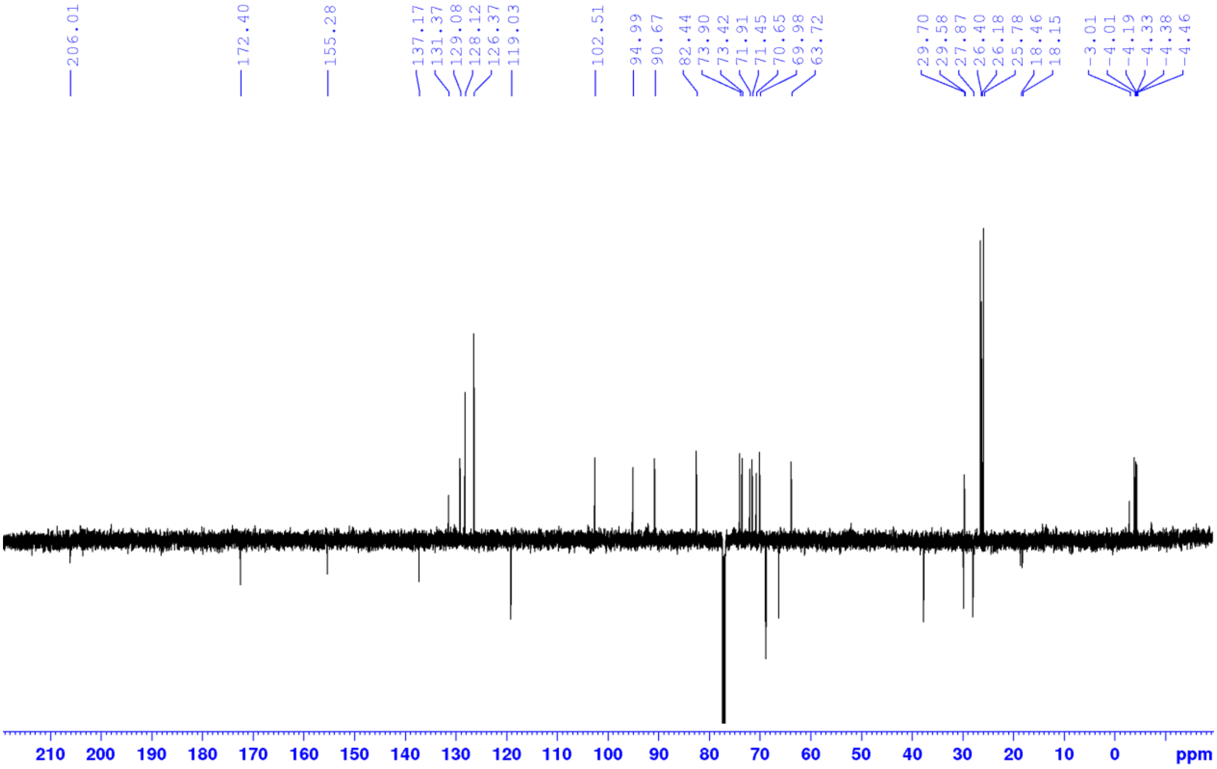

16 ^{1}H-NMR (600 MHz, $CDCl_3$)

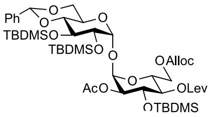

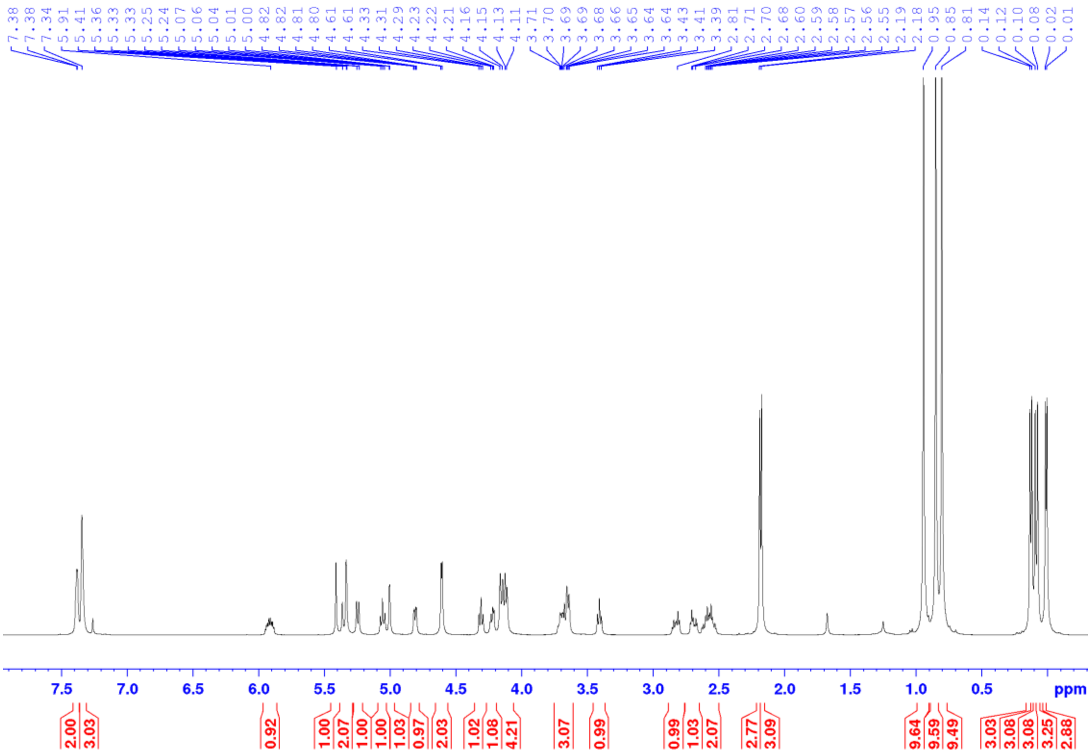

16 ^{13}C-NMR (151 MHz, $CDCl_3$)

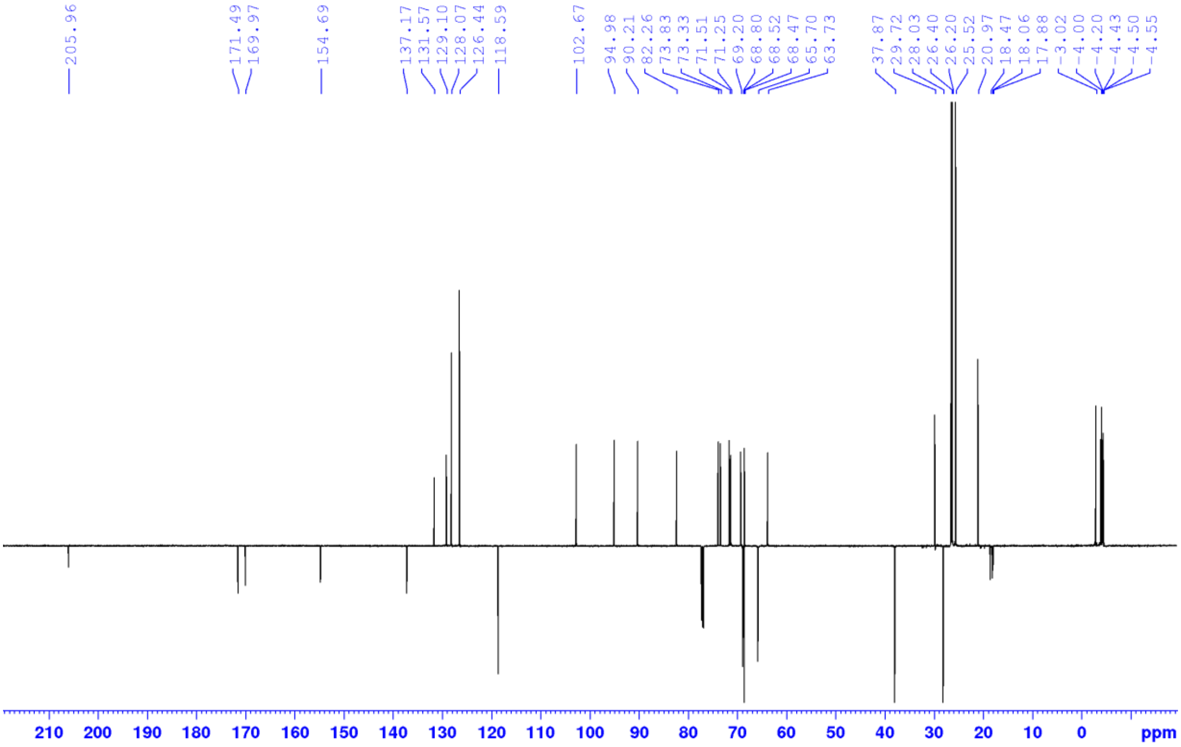

17 ^{1}H-NMR (600 MHz, $CDCl_3$)

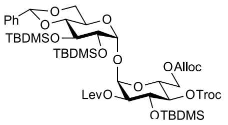

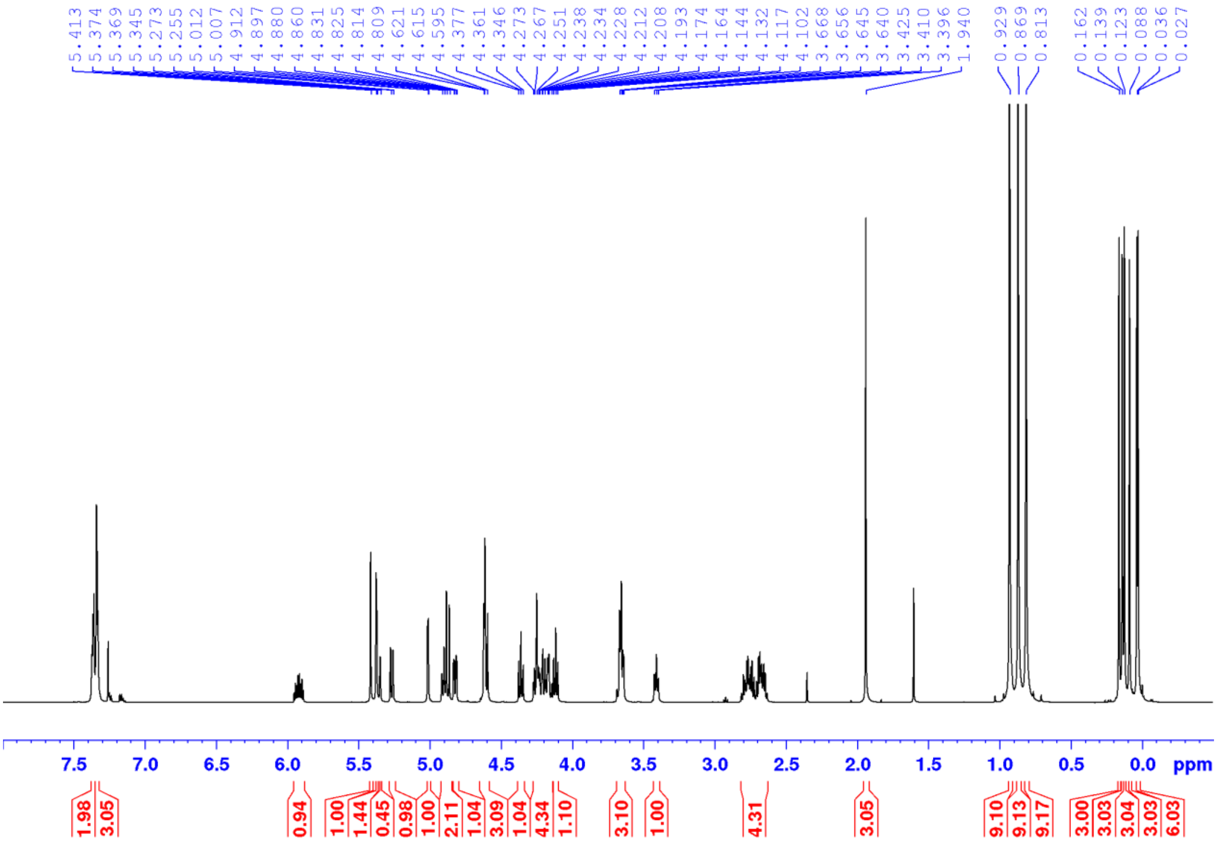

17 ^{13}C-NMR (151 MHz, $CDCl_3$)

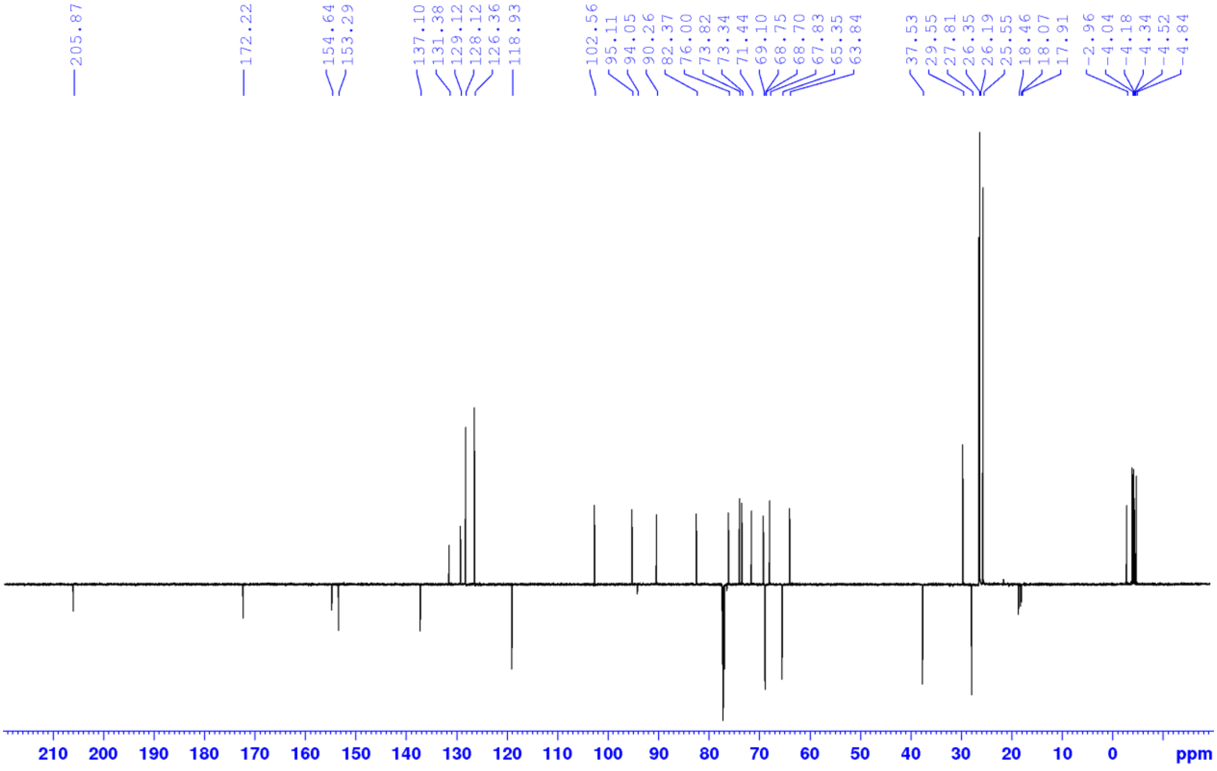

17 ^{1}H–^{29}Si HMBC ($CDCl_3$)

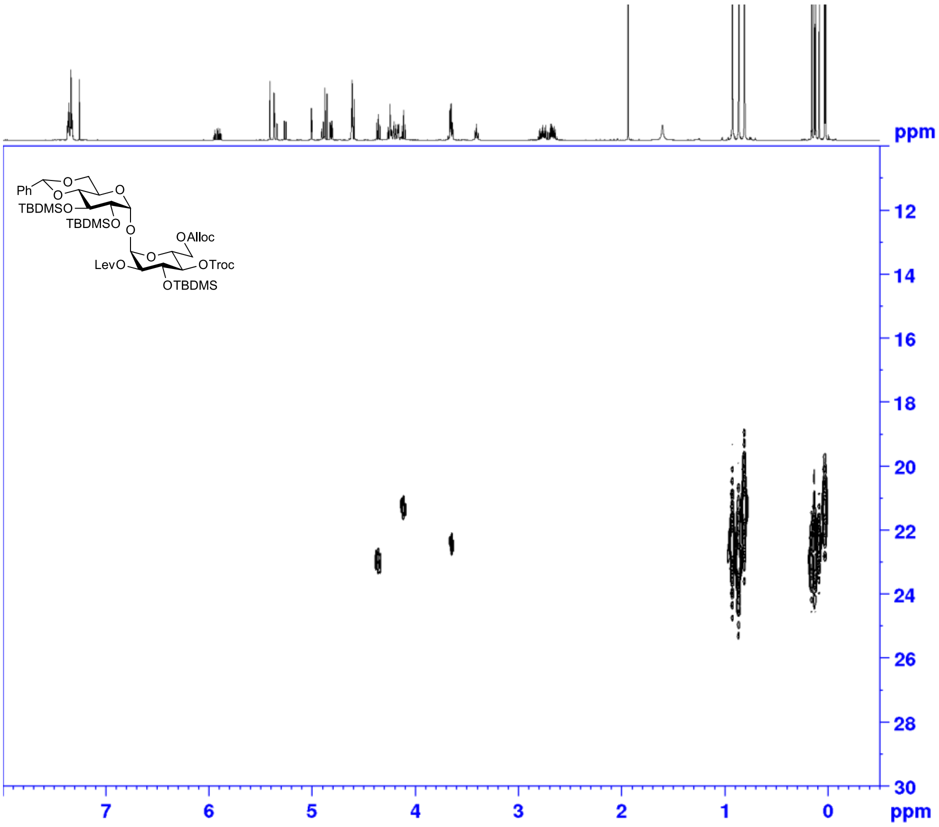

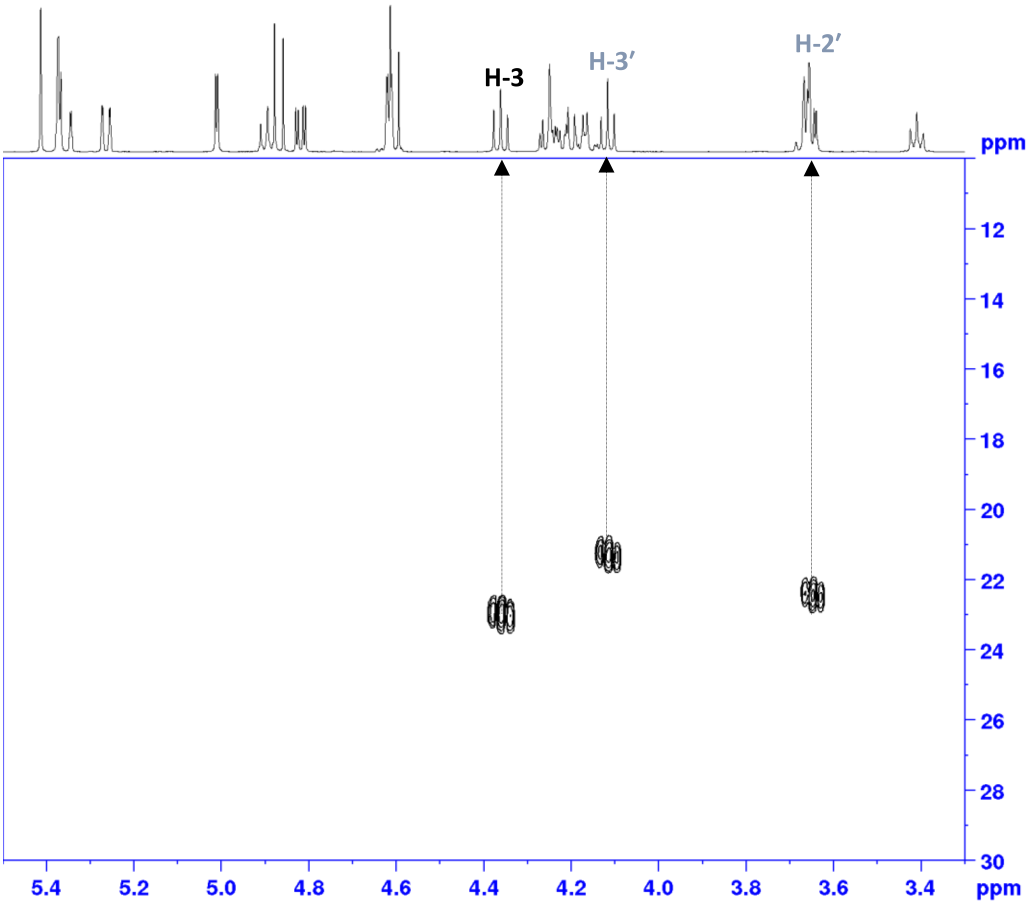

18 ^{1}H-NMR (600 MHz, CDCl$_3$)

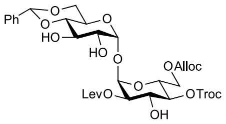

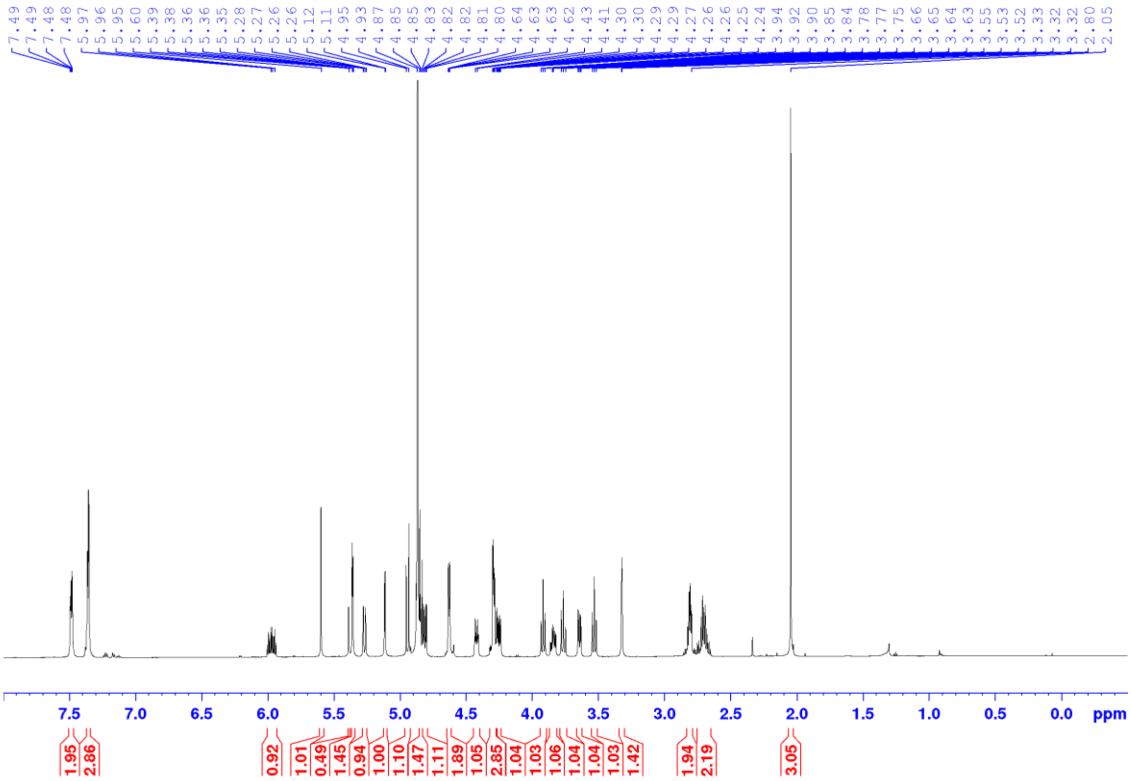

18 ^{13}C-NMR (151 MHz, CDCl$_3$)

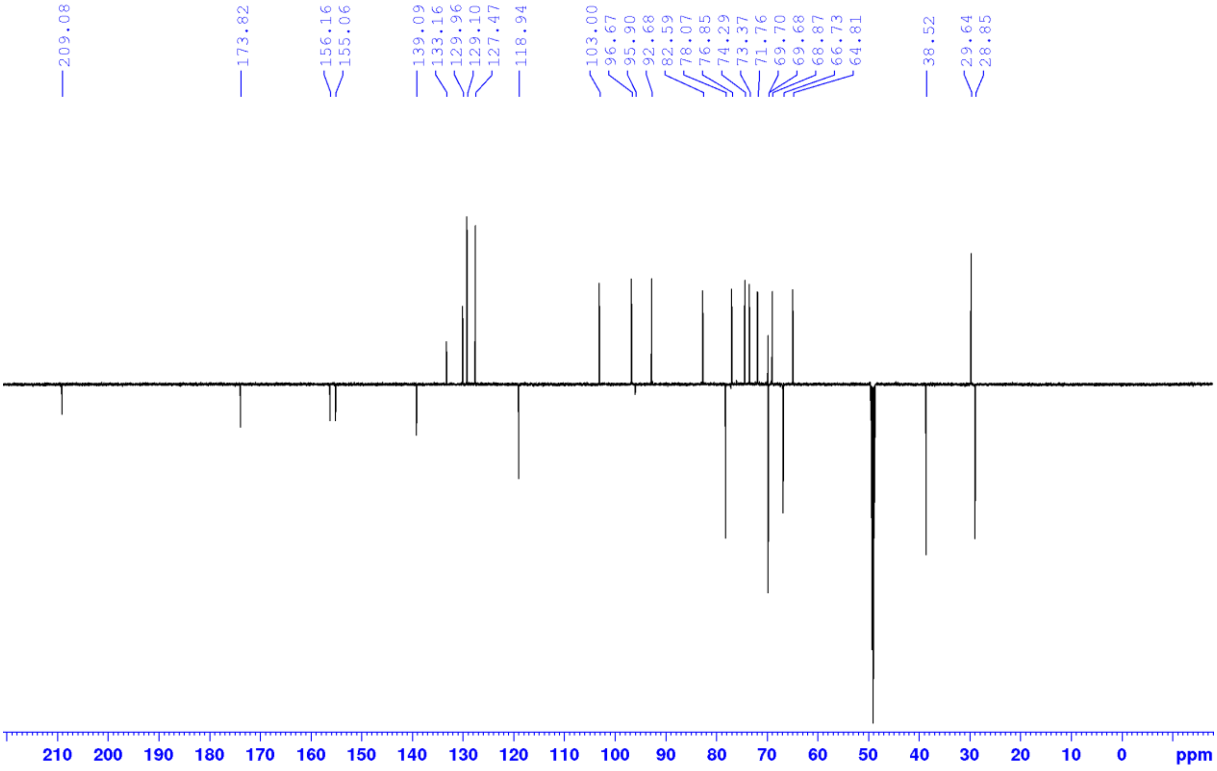

19 ^{1}H-NMR (600 MHz, $CDCl_3$)

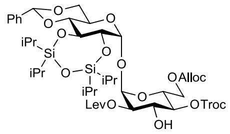

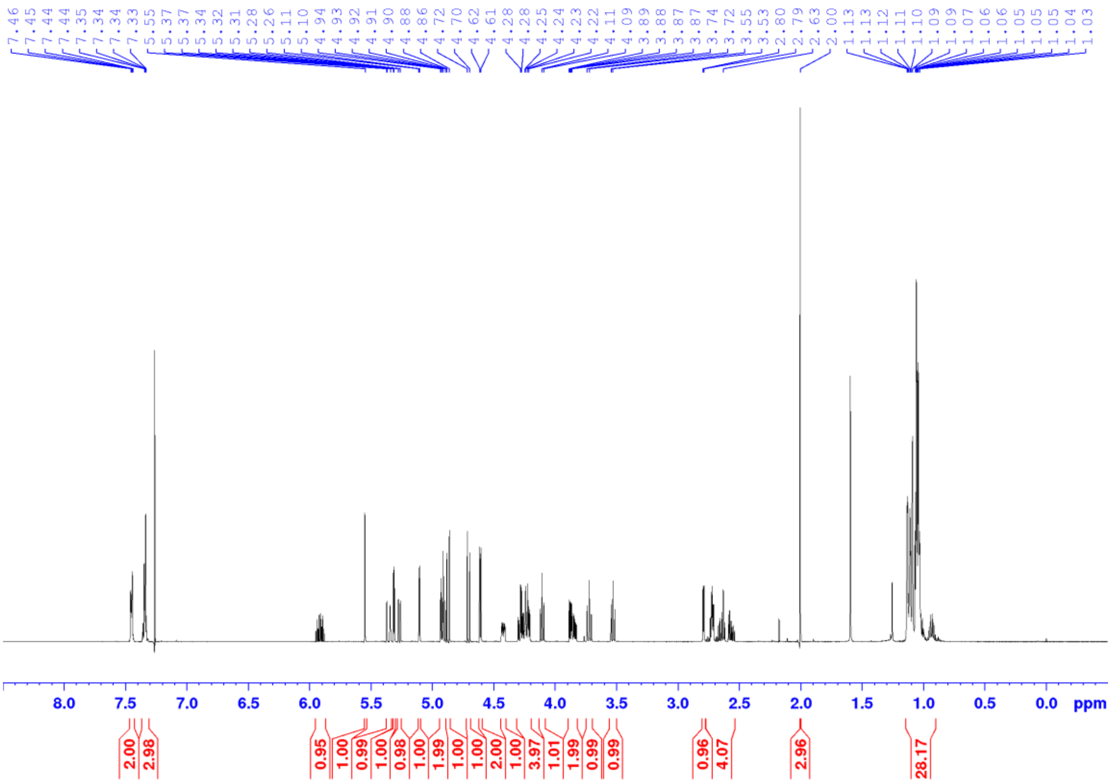

19 ^{13}C NMR (151 MHz, $CDCl_3$)

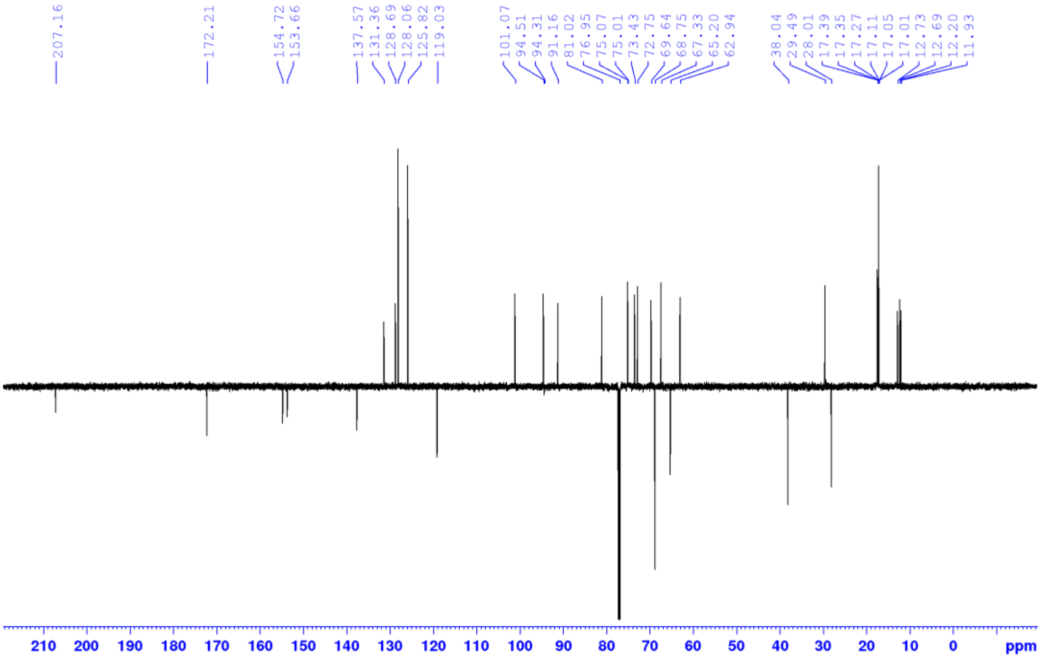

19 ^{1}H-^{29}Si NMR ($CDCl_3$)

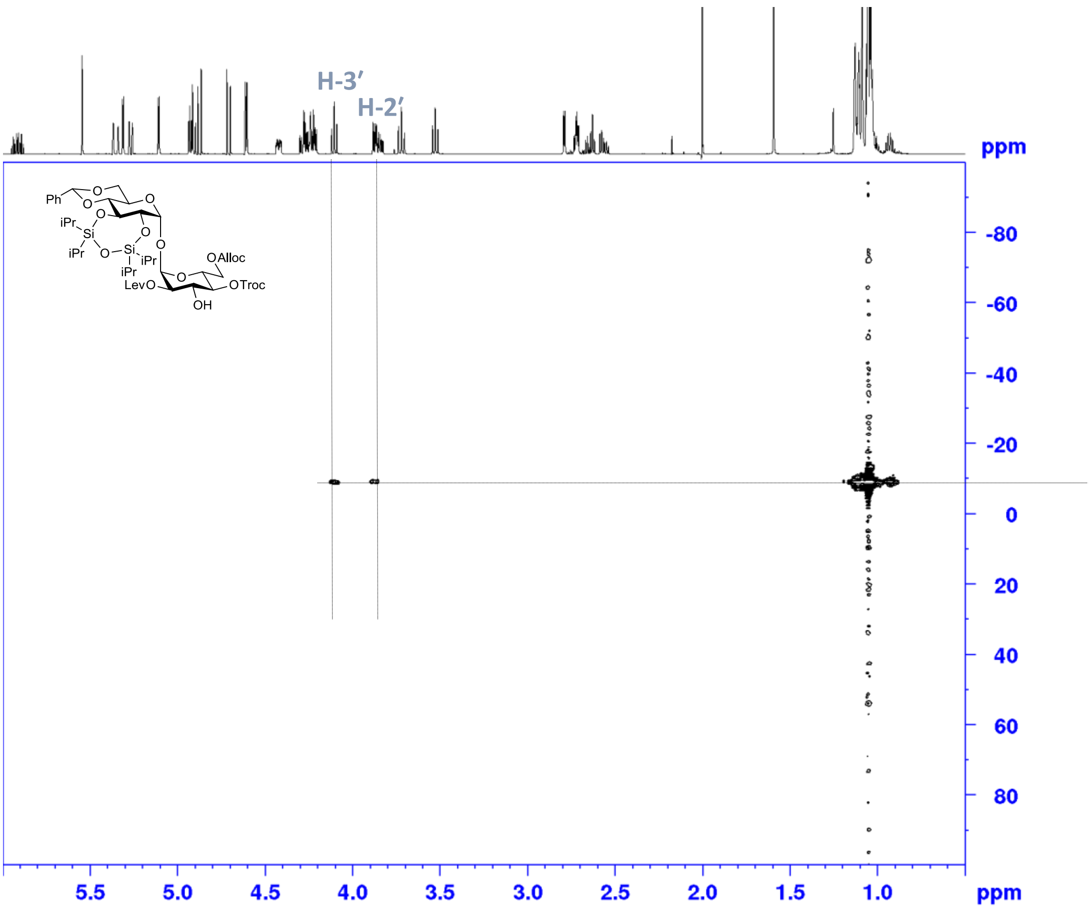

20 ^{1}H-NMR (600 MHz, $CDCl_3$)

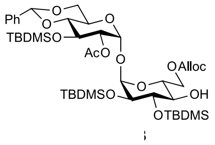

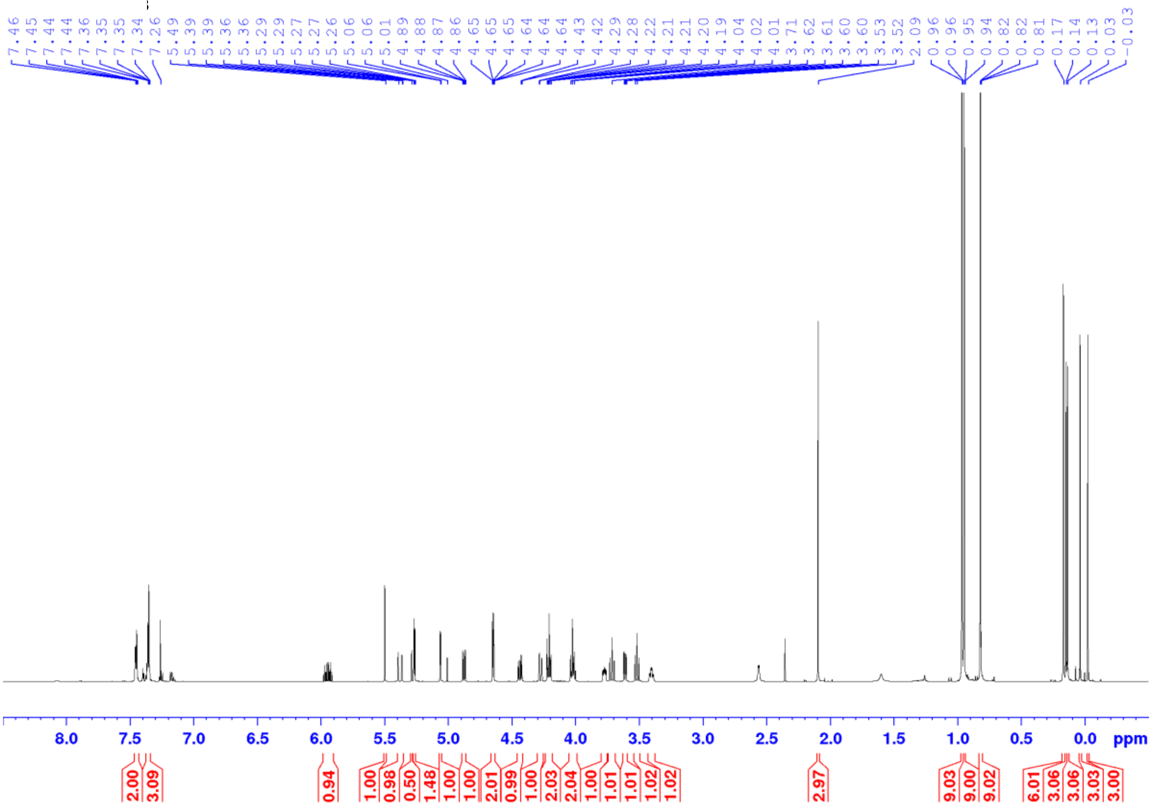

20 ^{13}C-NMR (151 MHz, $CDCl_3$)

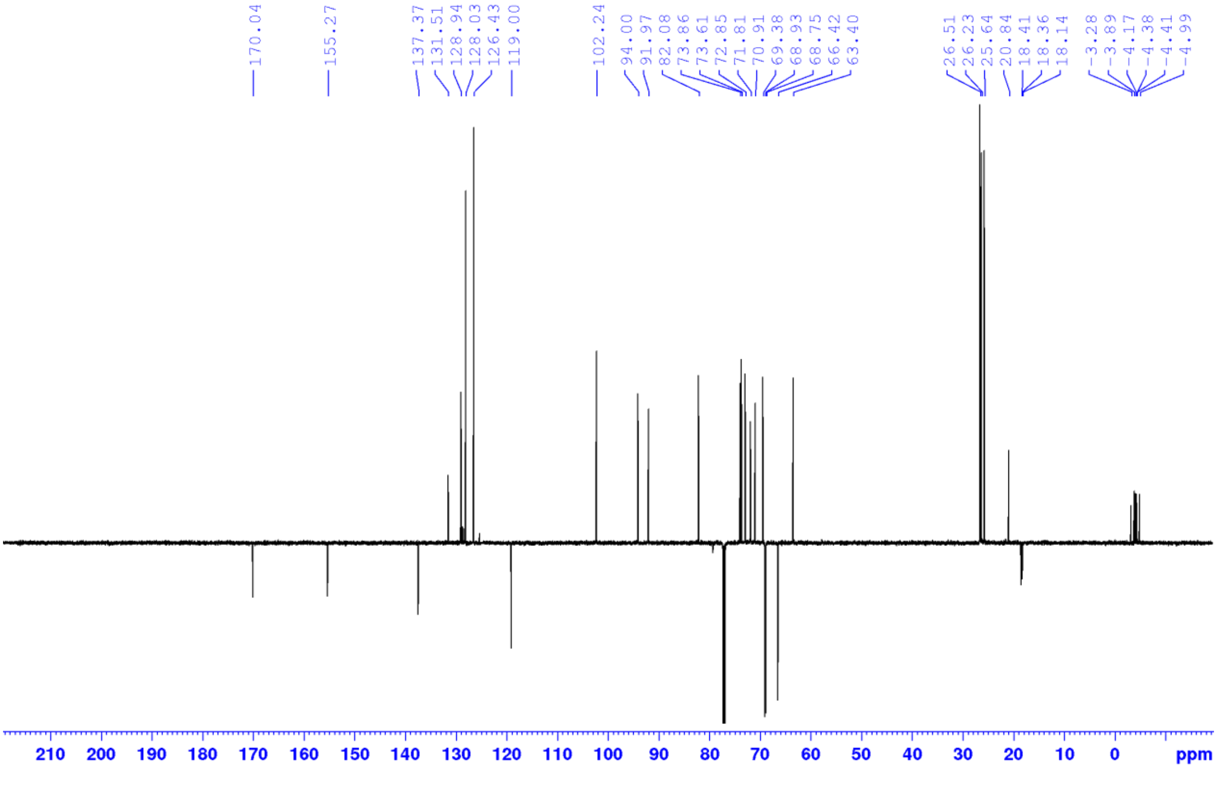

21 ^{1}H-NMR (600 MHz, $CDCl_3$)

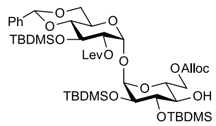

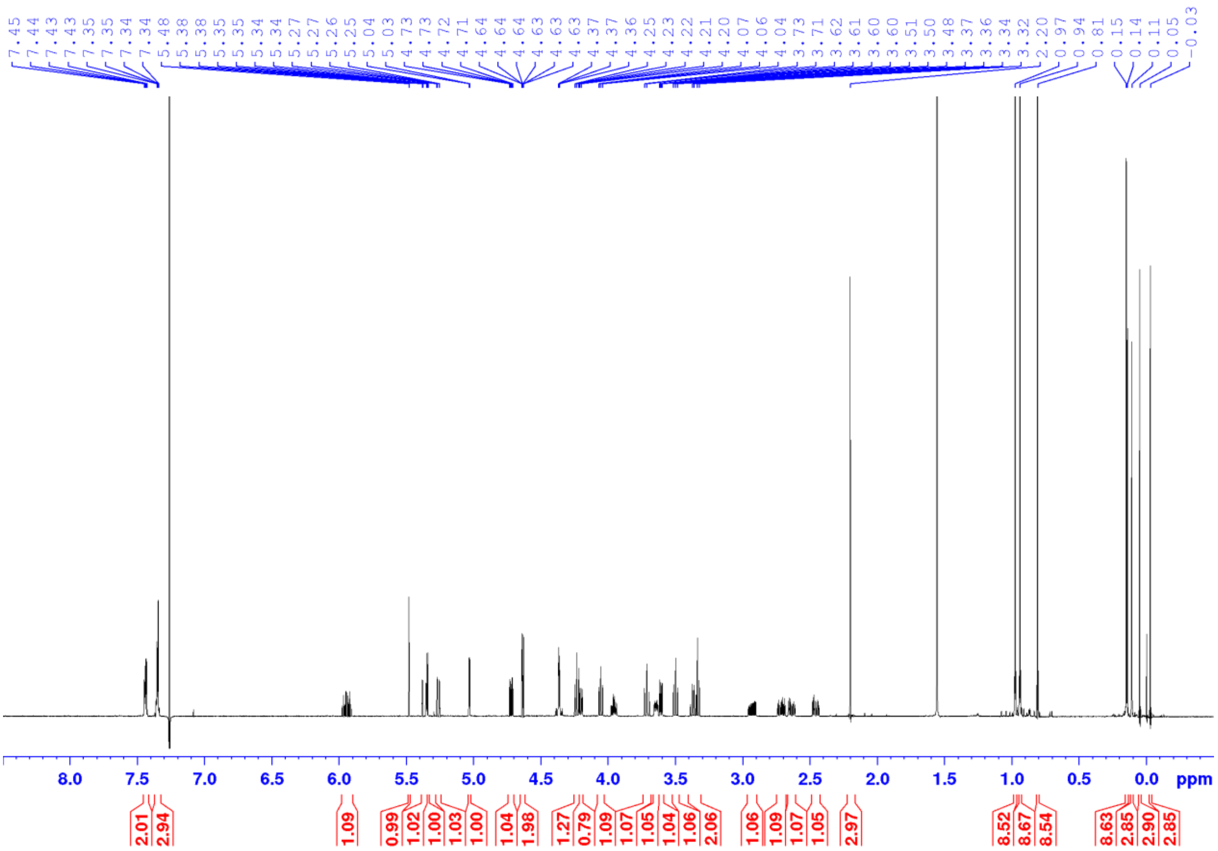

21 ^{13}C-NMR (151 MHz, $CDCl_3$)

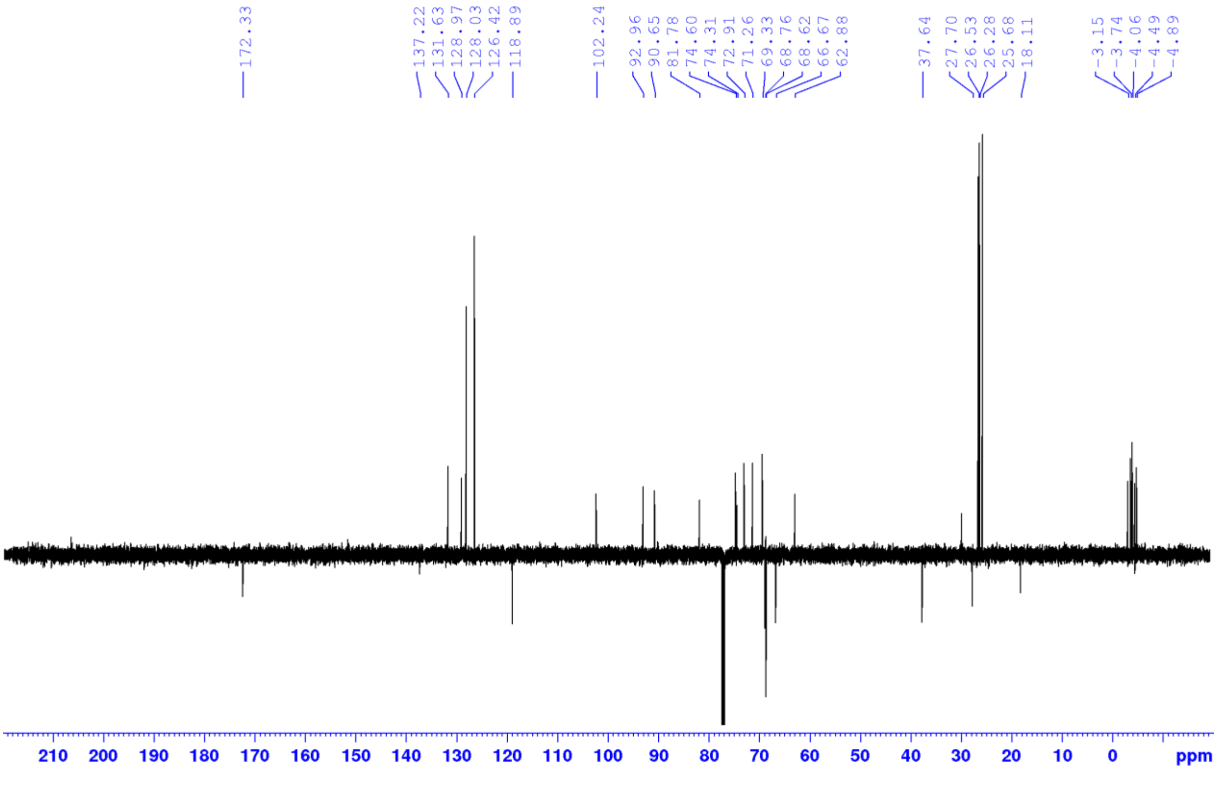

22 ^{1}H-NMR (600 MHz, $CDCl_3$)

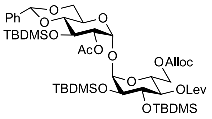

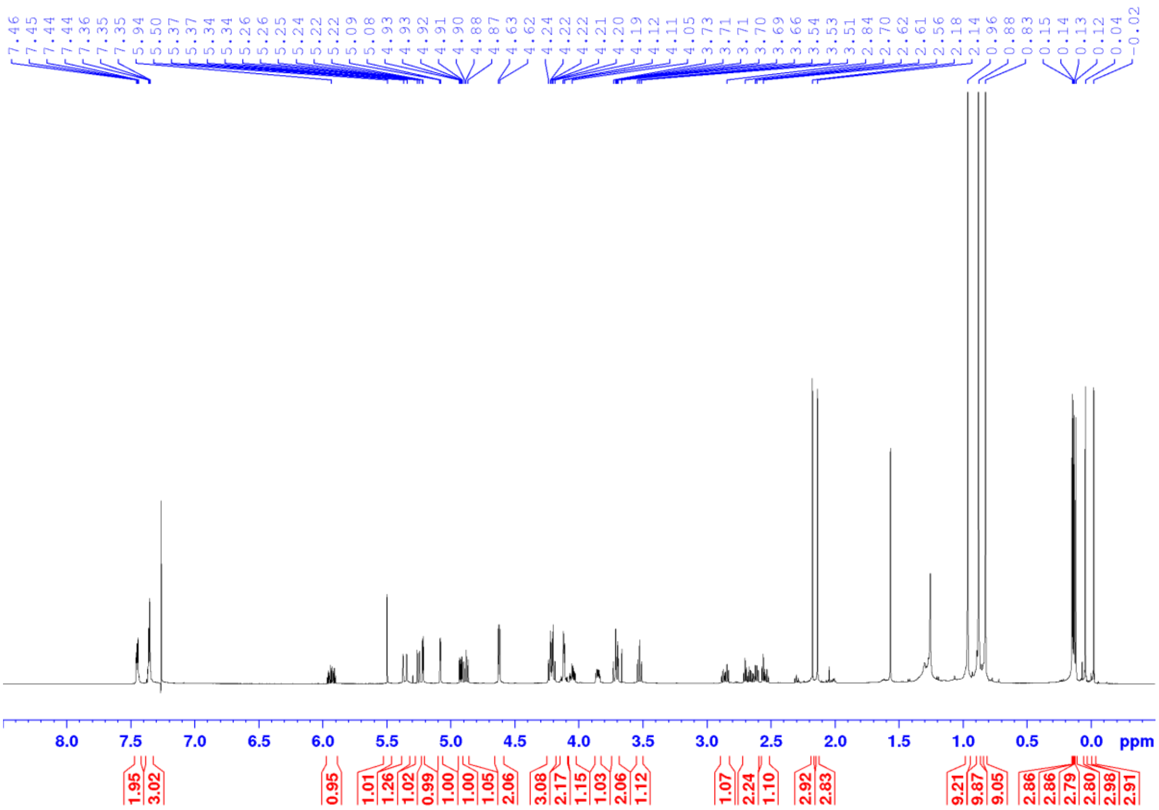

22 ^{13}C-NMR (151 MHz, $CDCl_3$)

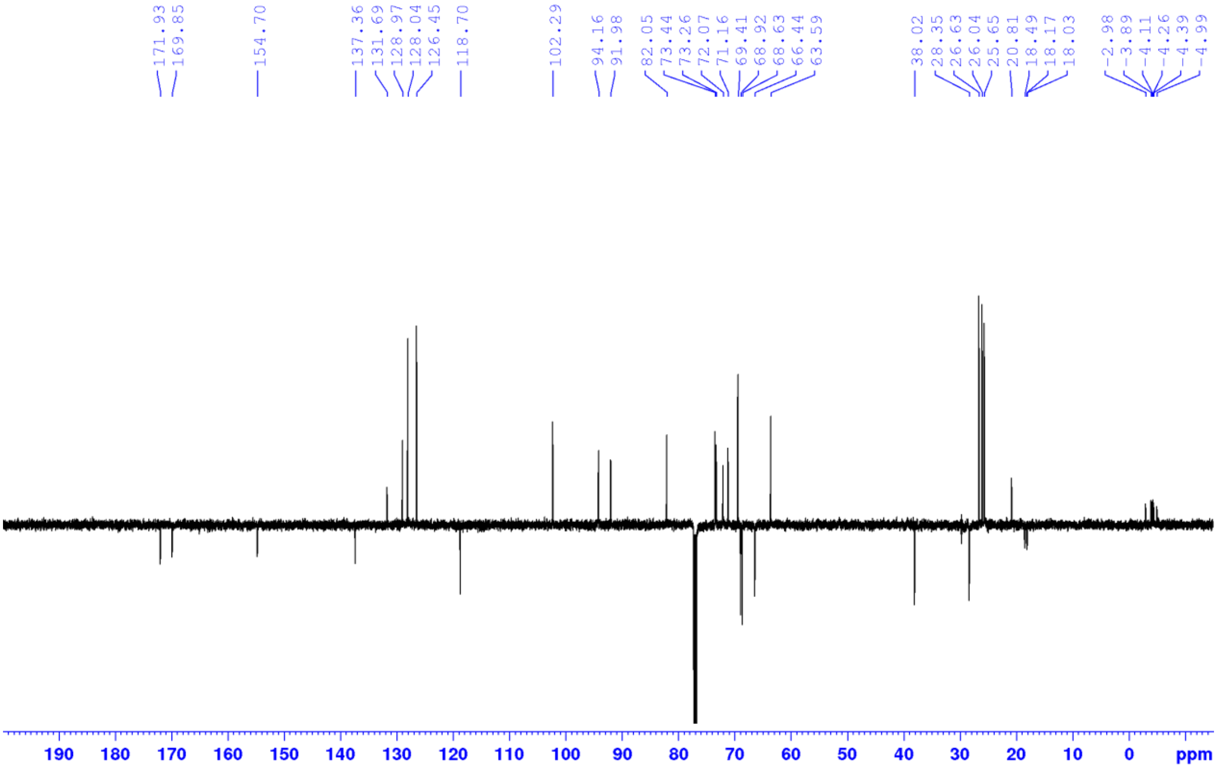

23 ^{1}H-NMR (600 MHz, $CDCl_3$)

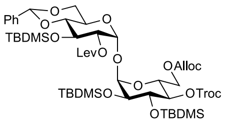

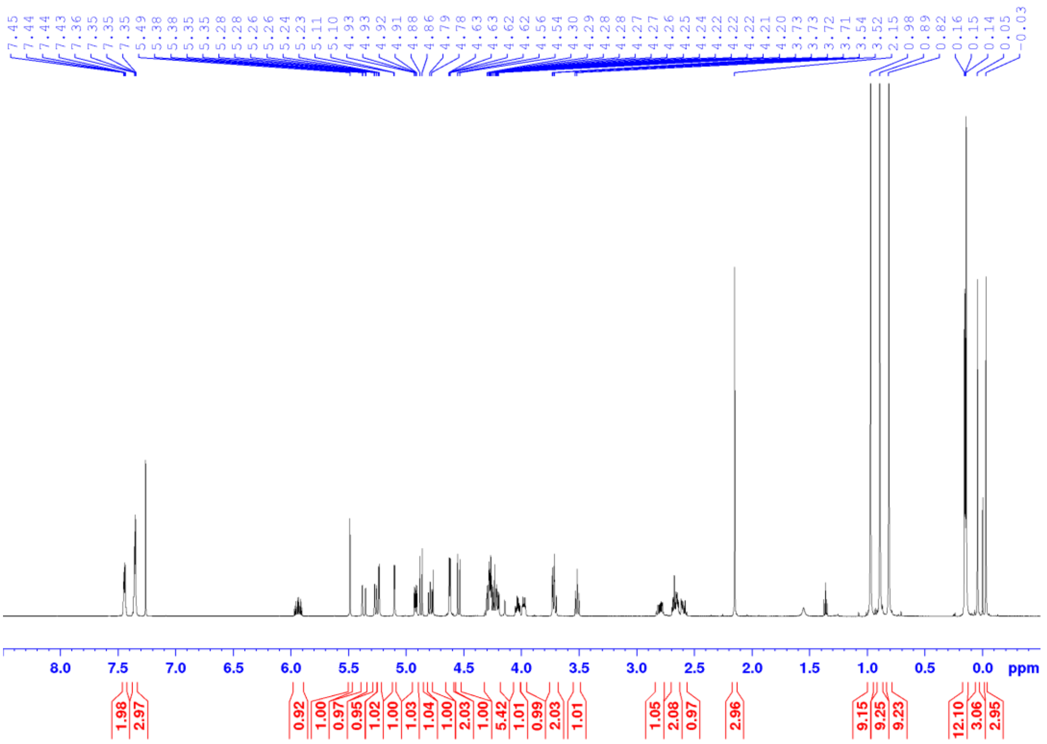

23 ^{13}C-NMR (151 MHz, $CDCl_3$)

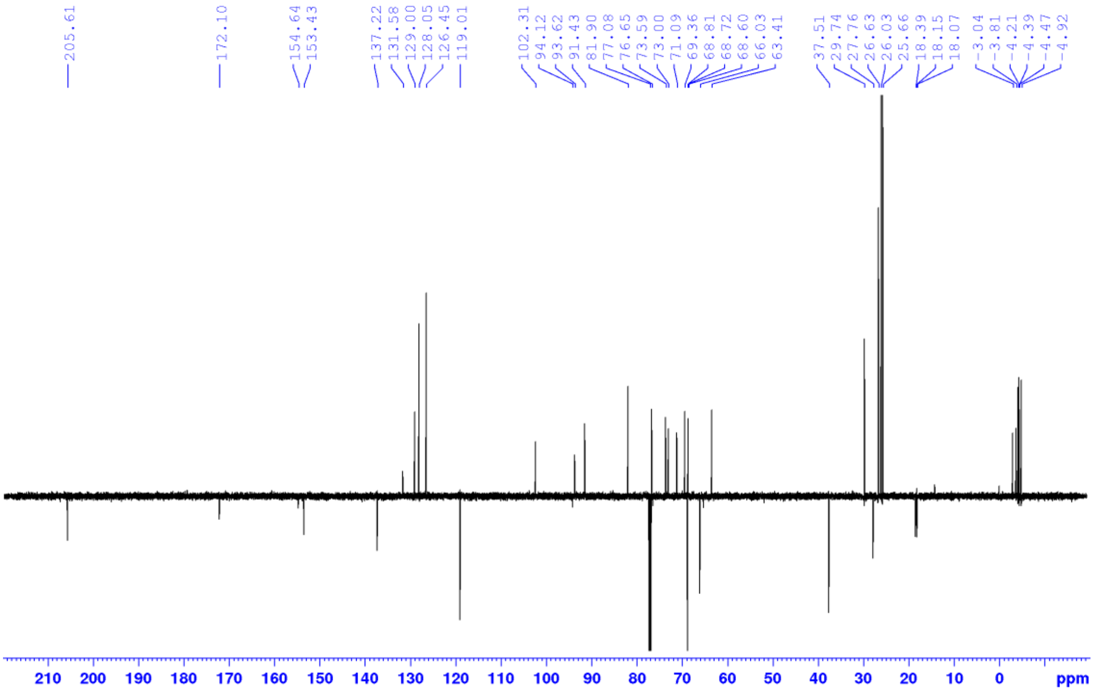

23 ^{1}H–^{29}Si HMBC ($CDCl_3$)

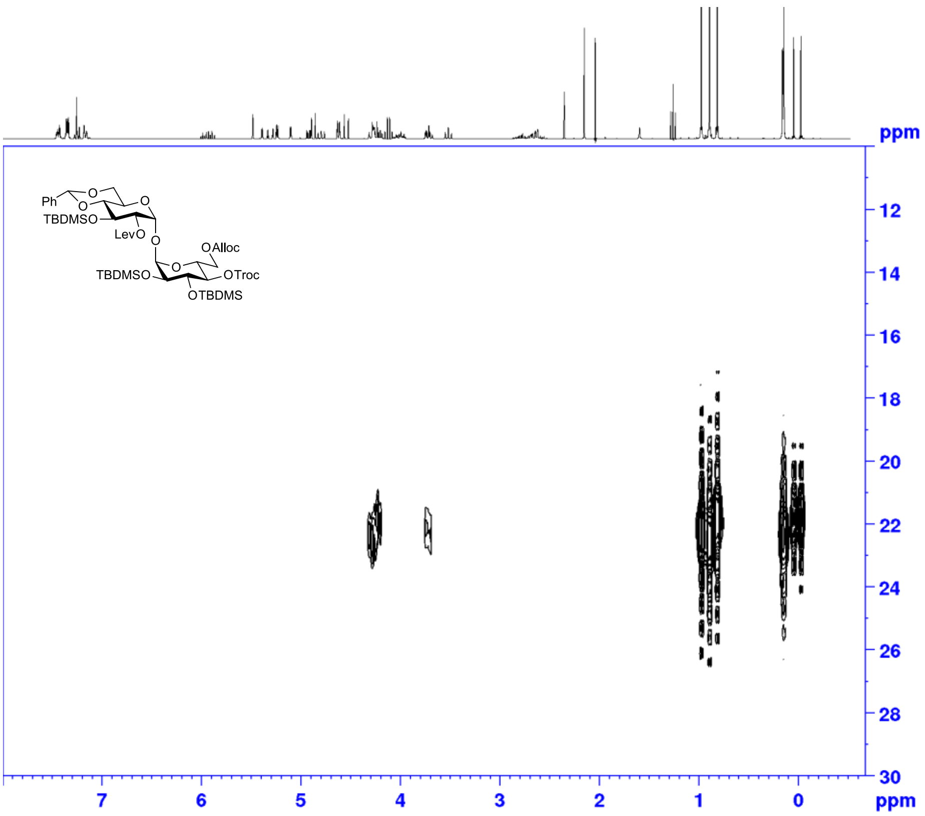

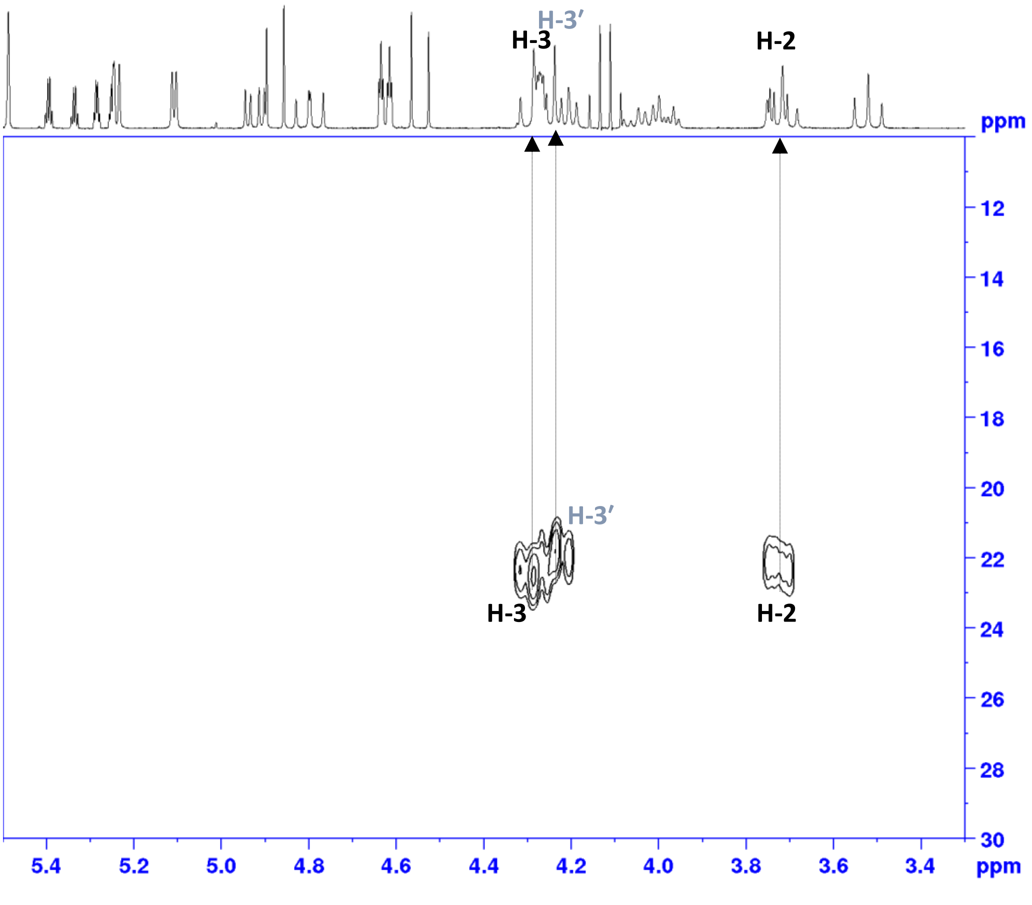

24 ^{1}H-NMR (600 MHz, MeOD)

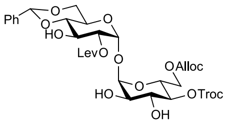

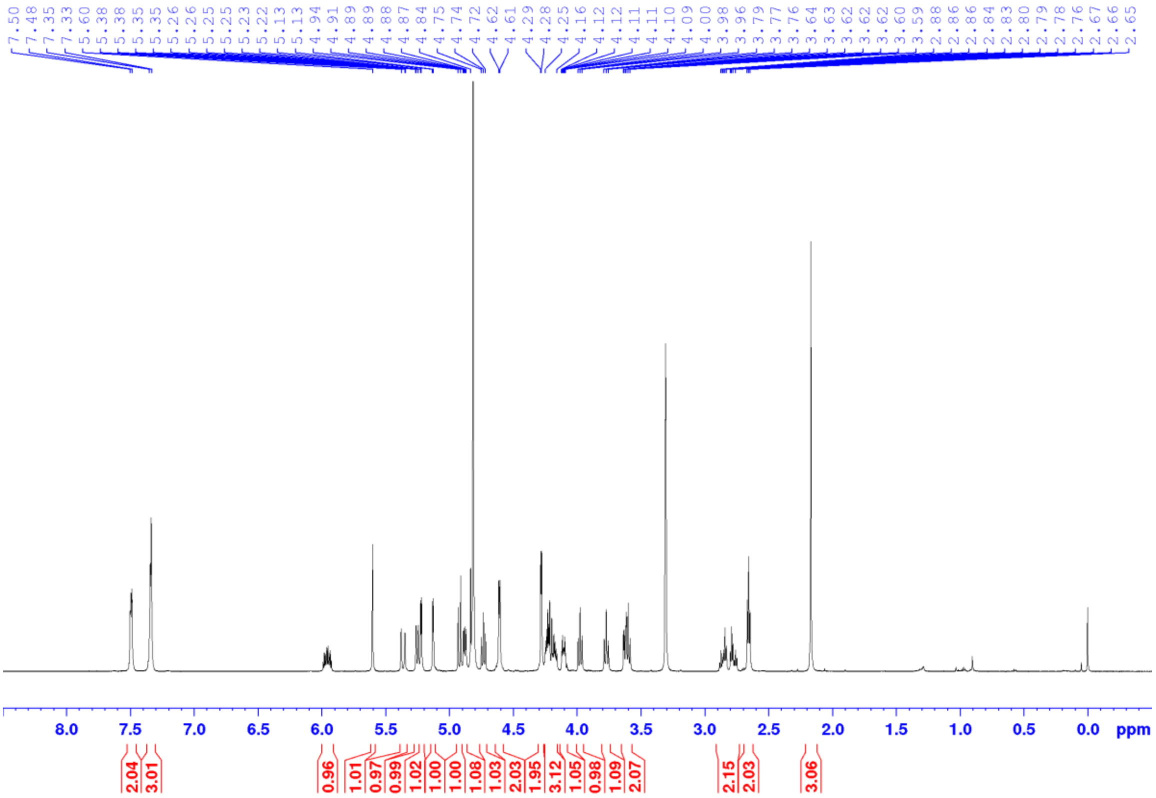

24 ^{13}C-NMR (151 MHz, MeOD)

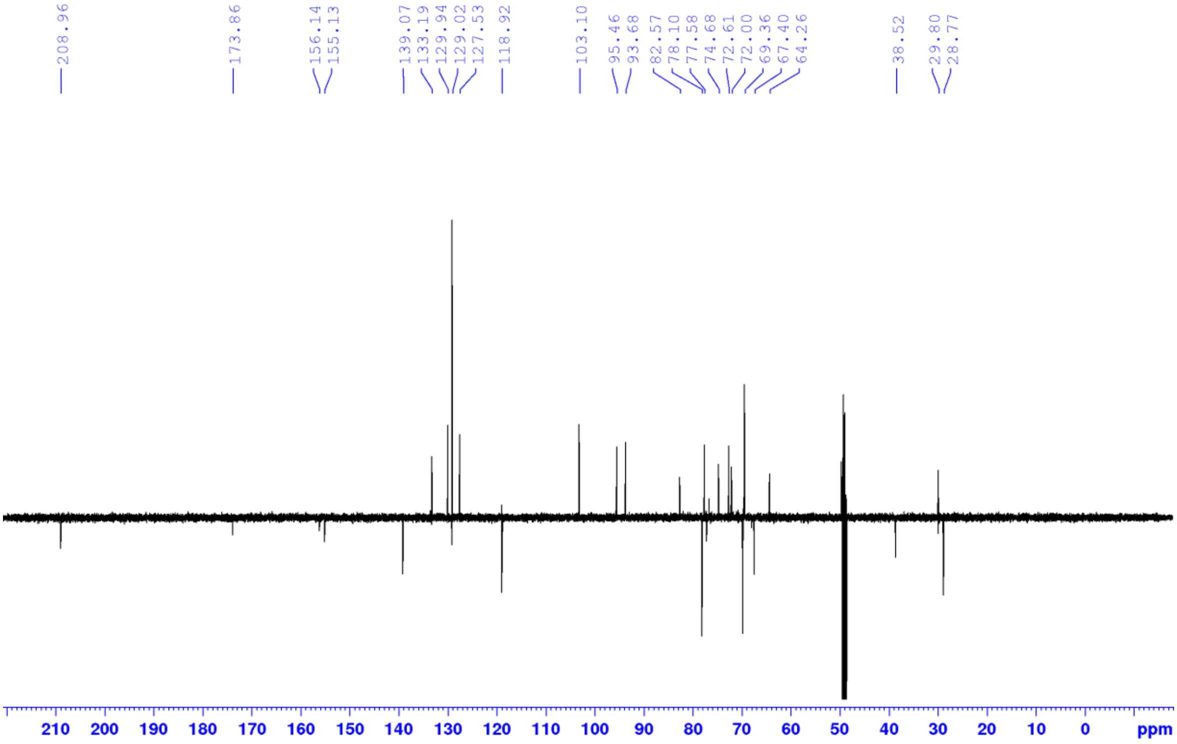

25 ^{1}H-NMR (600 MHz, $CDCl_3$)

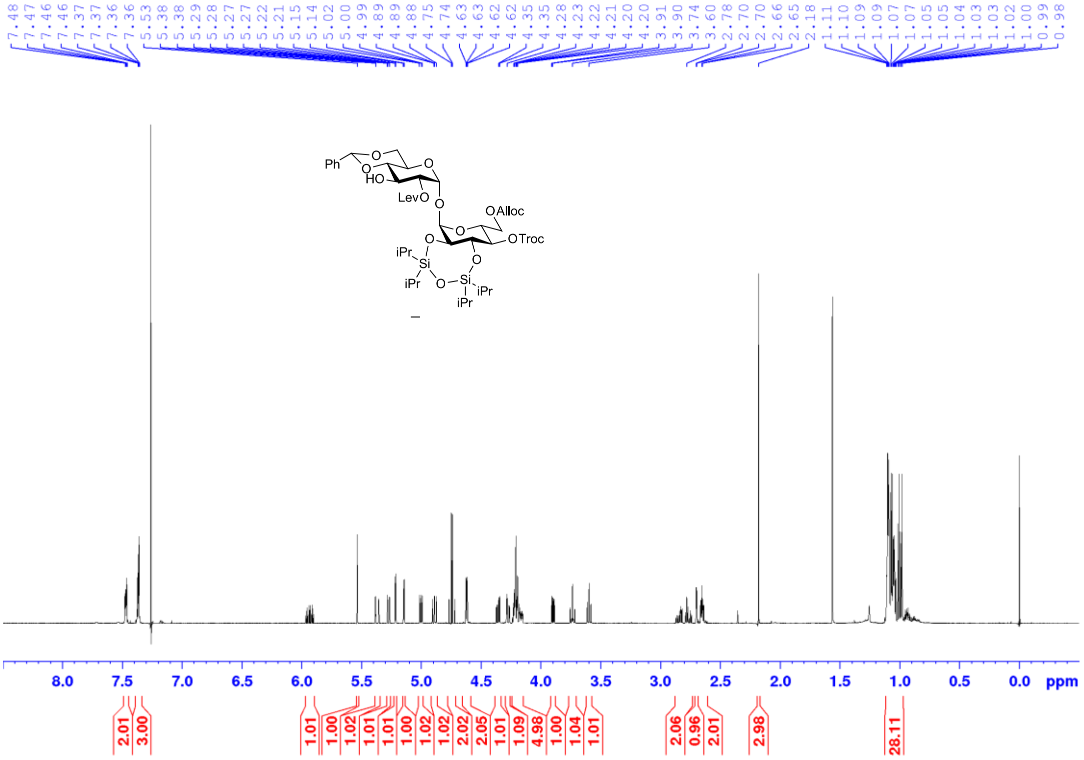

25 ^{13}C-NMR (151 MHz, $CDCl_3$)

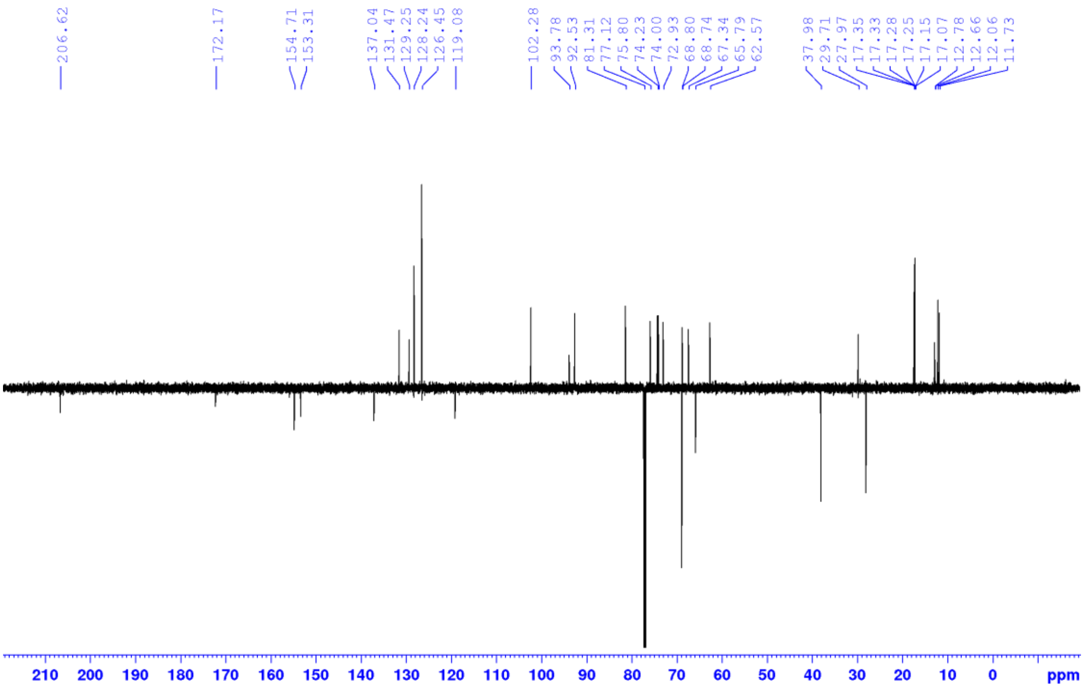

25 ^{1}H-^{29}Si NMR ($CDCl_3$)

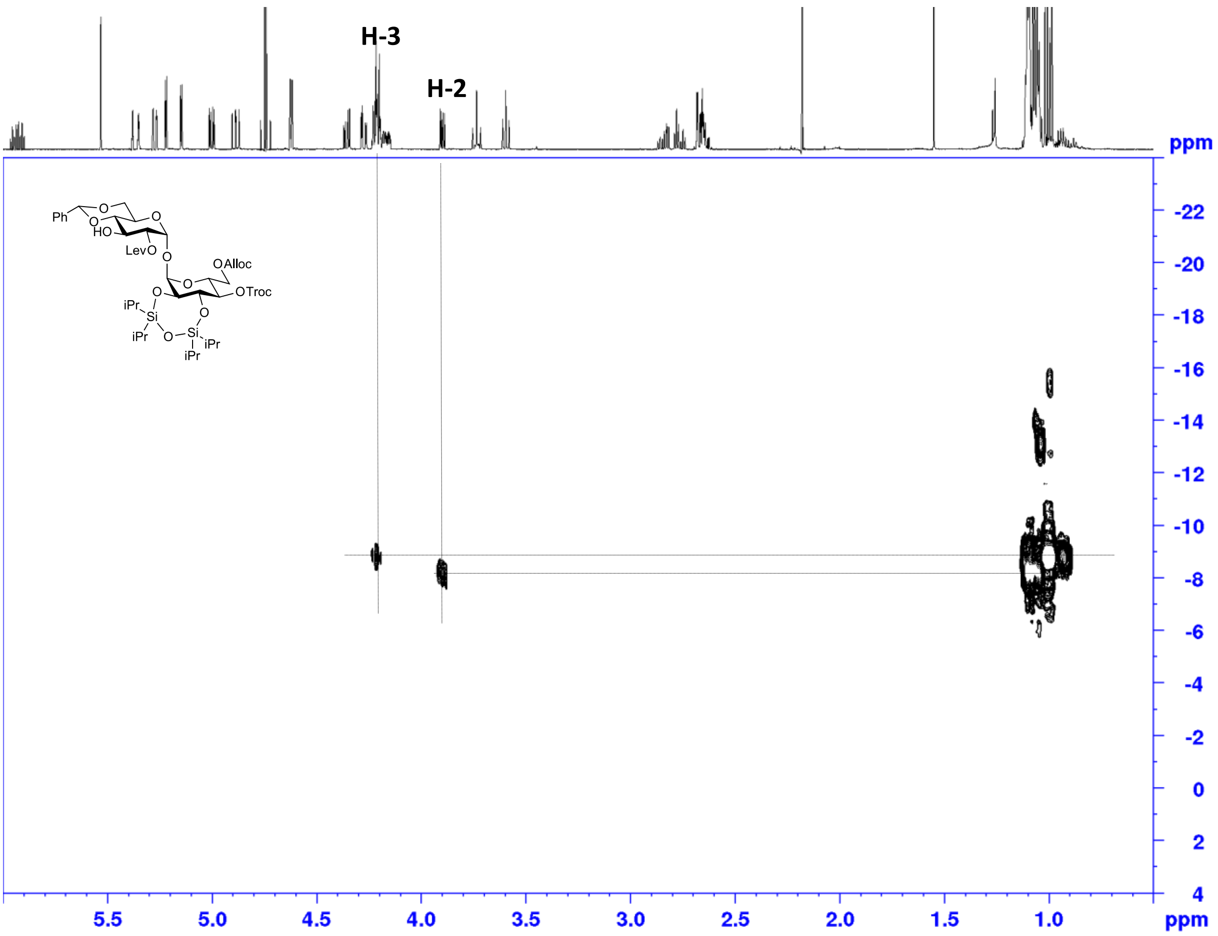

26 ^{1}H-NMR (600 MHz, $CDCl_3$)

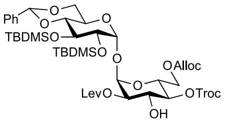

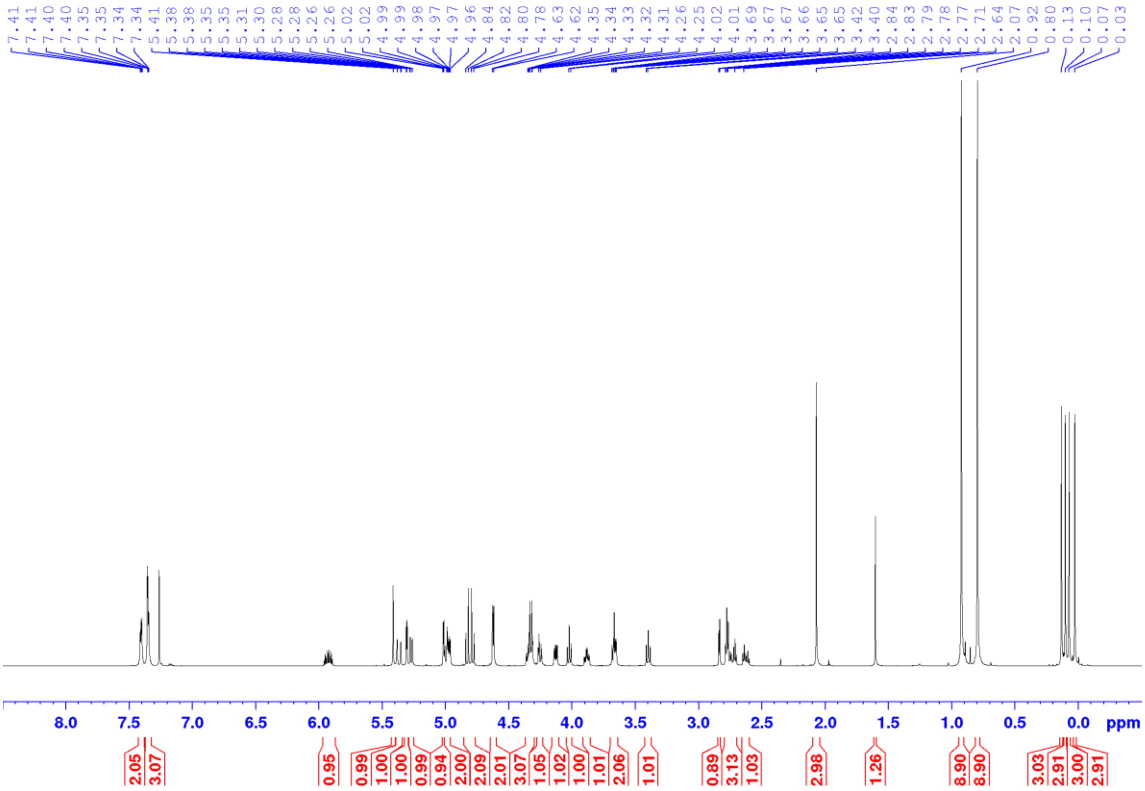

26 ^{13}C-NMR (151 MHz, $CDCl_3$)

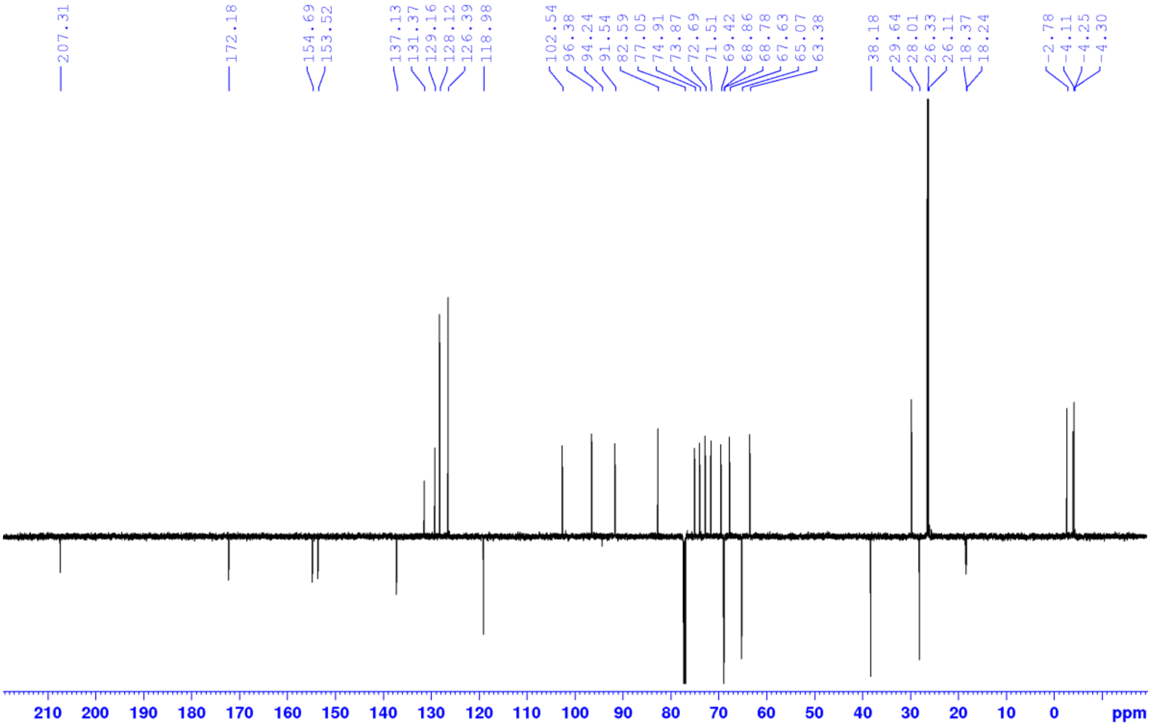

26 ^{1}H–^{29}Si HMBC (CDCl$_3$)

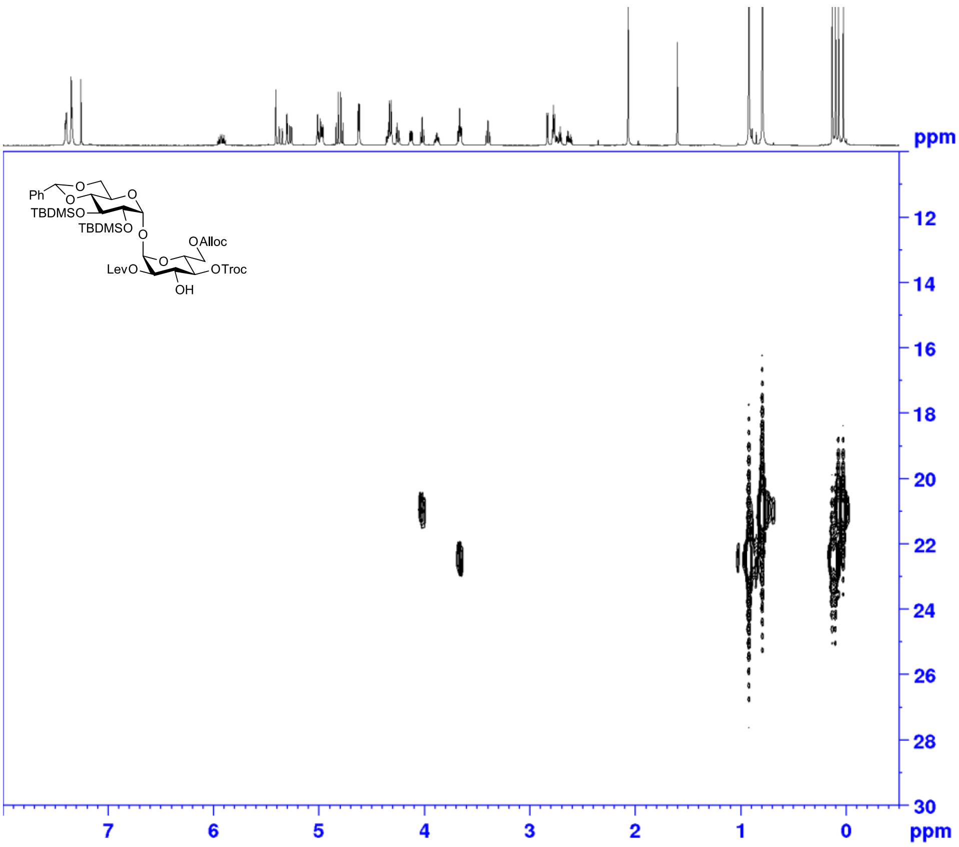

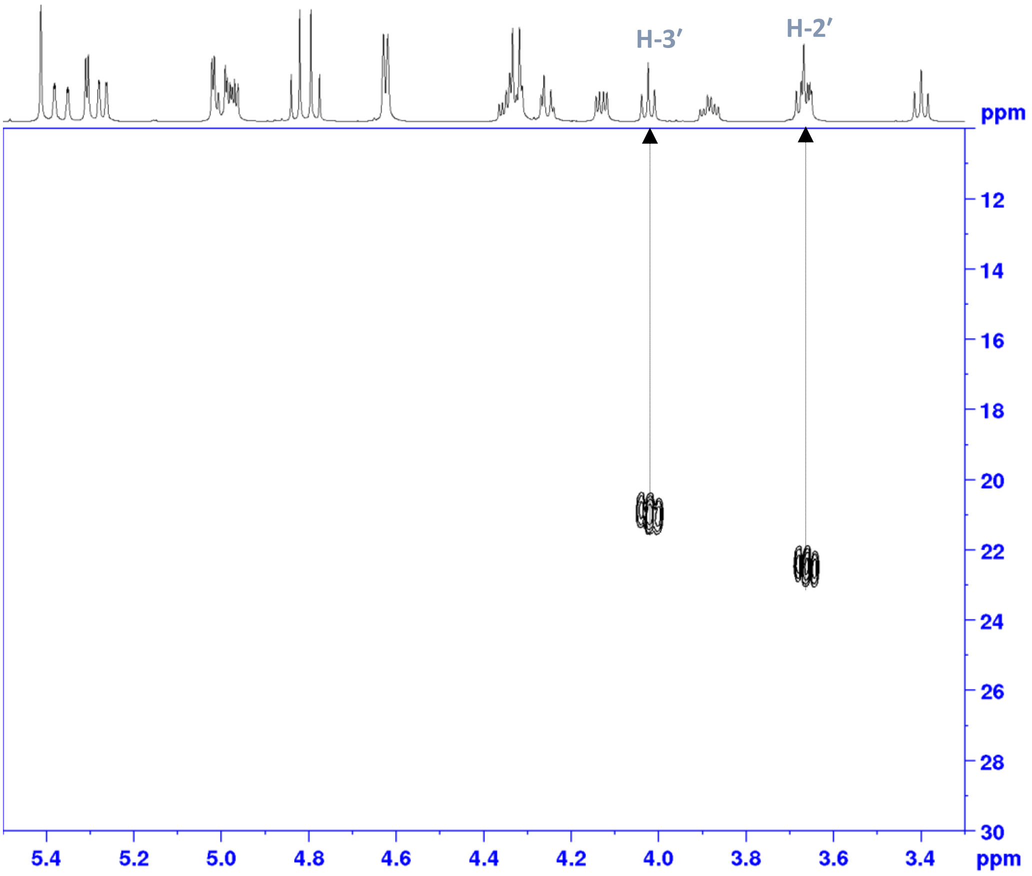

27 ^{1}H-NMR (600 MHz, $CDCl_3$)

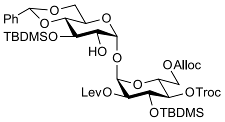

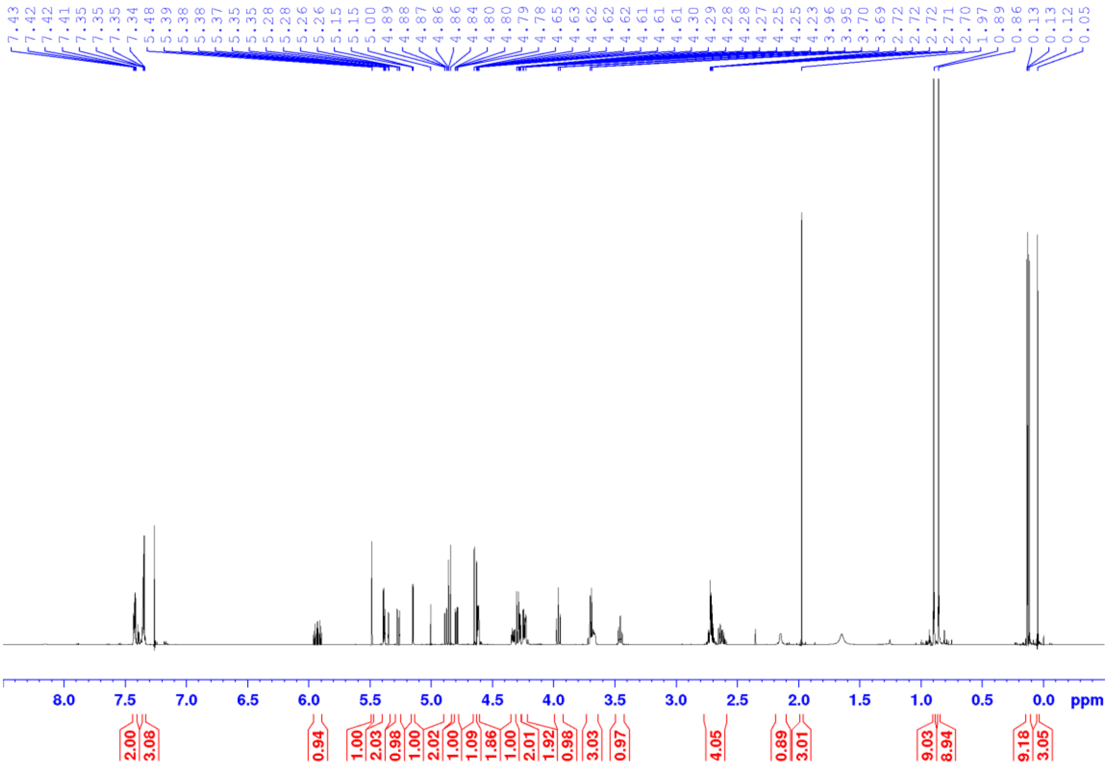

27 ^{13}C-NMR (151 MHz, $CDCl_3$)

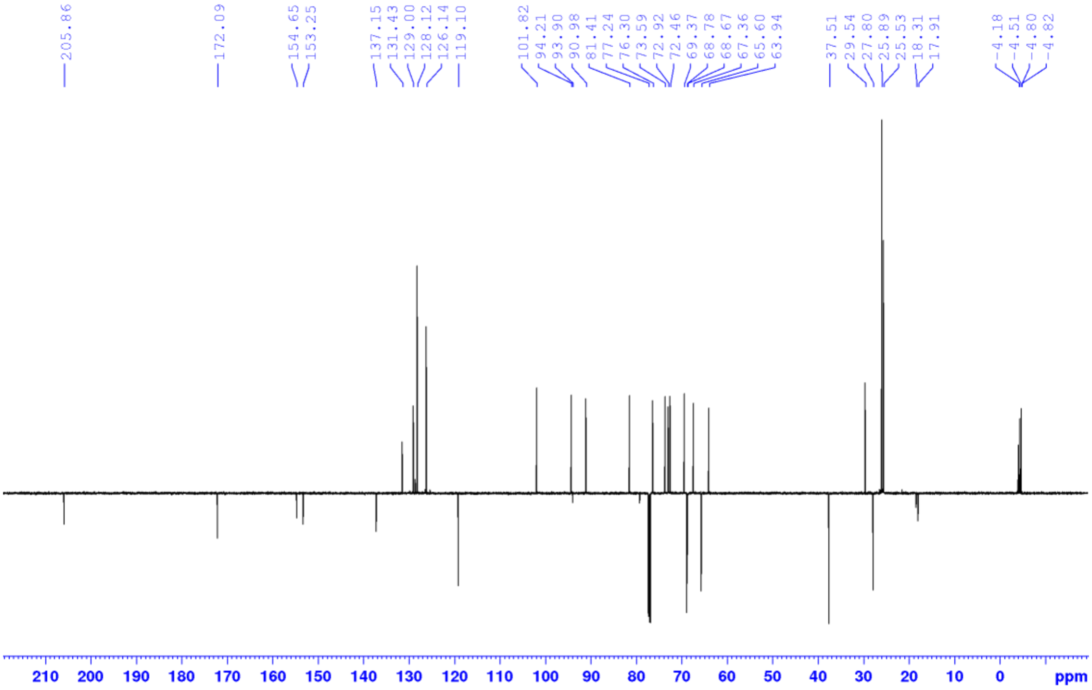

27 ^{1}H–^{29}Si HMBC ($CDCl_3$)

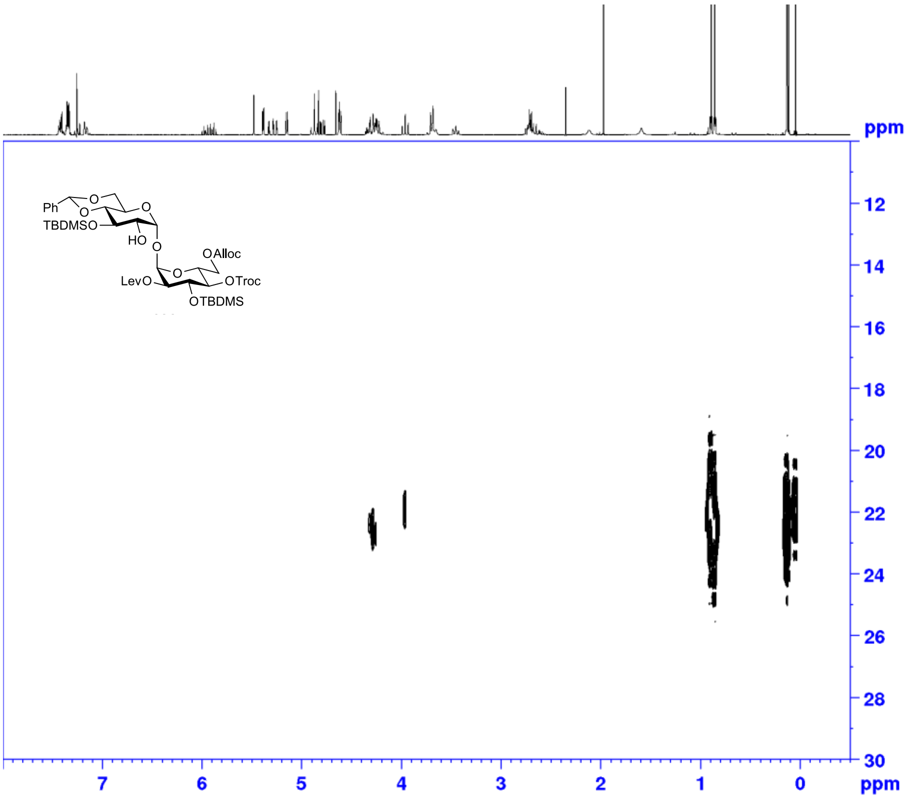

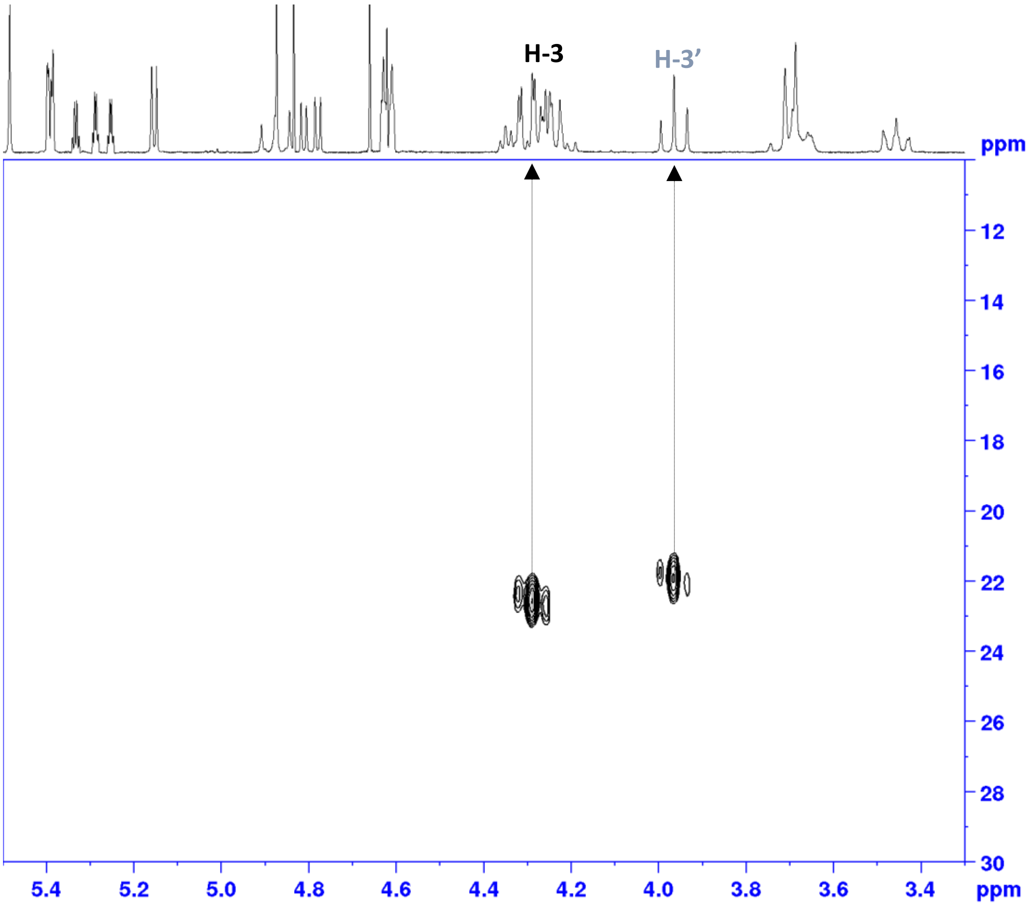

28 ^{1}H-NMR (600 MHz, $CDCl_3$)

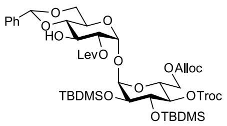

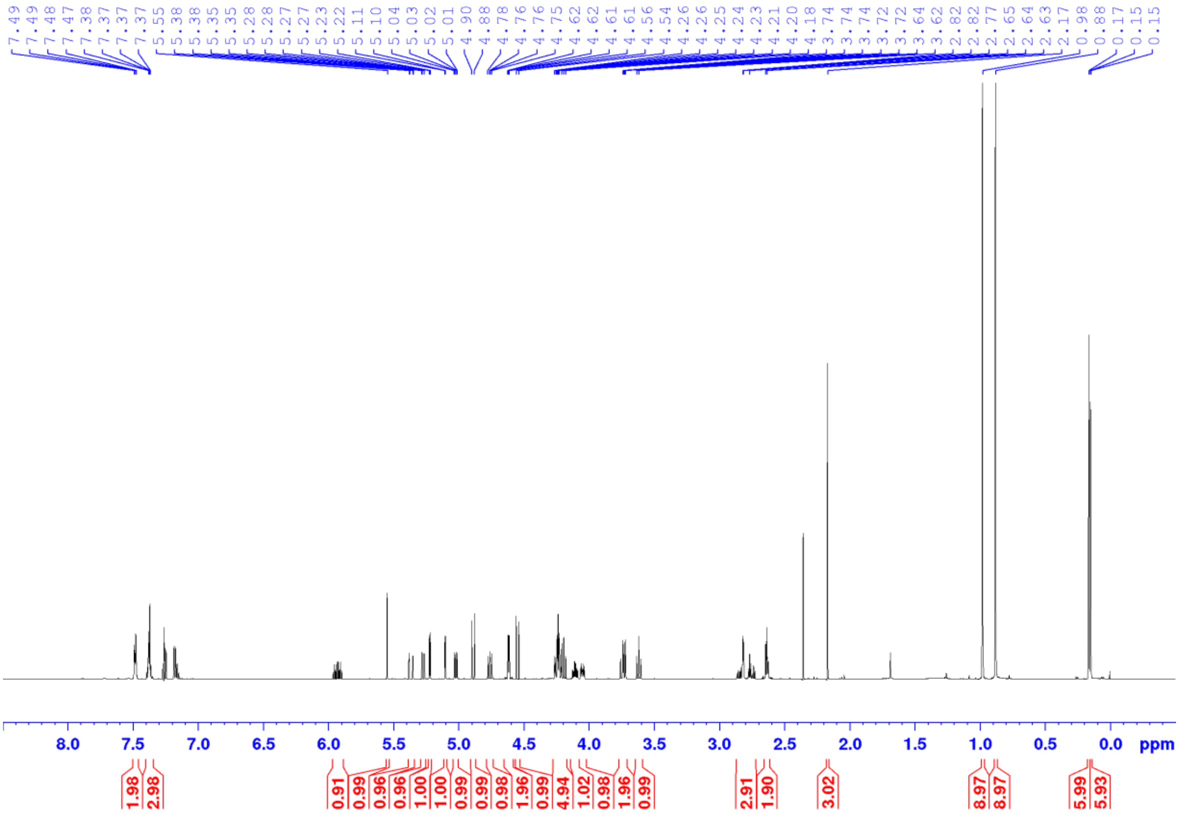

28 ^{13}C-NMR (151 MHz, $CDCl_3$)

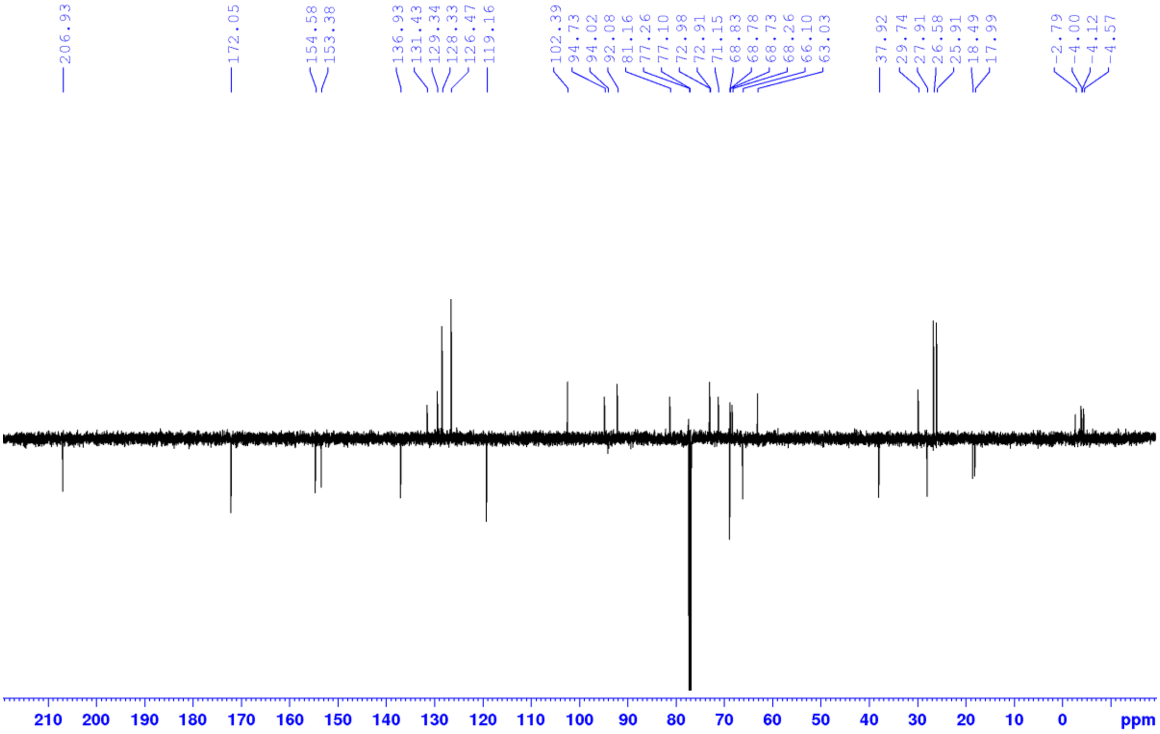

28 ^1H–^{29}Si HMBC ($CDCl_3$)

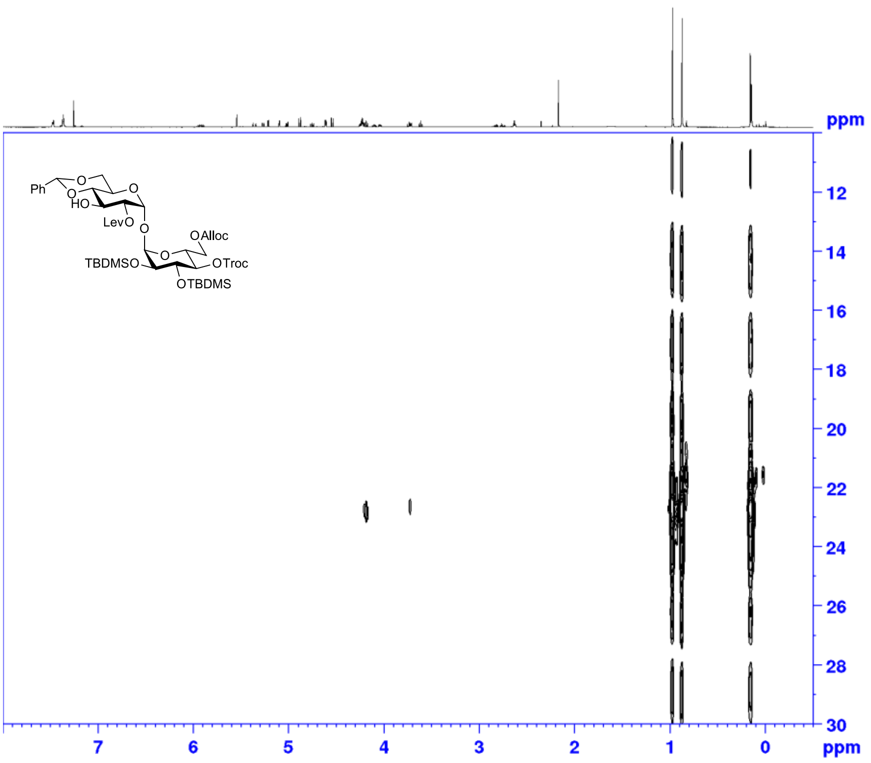

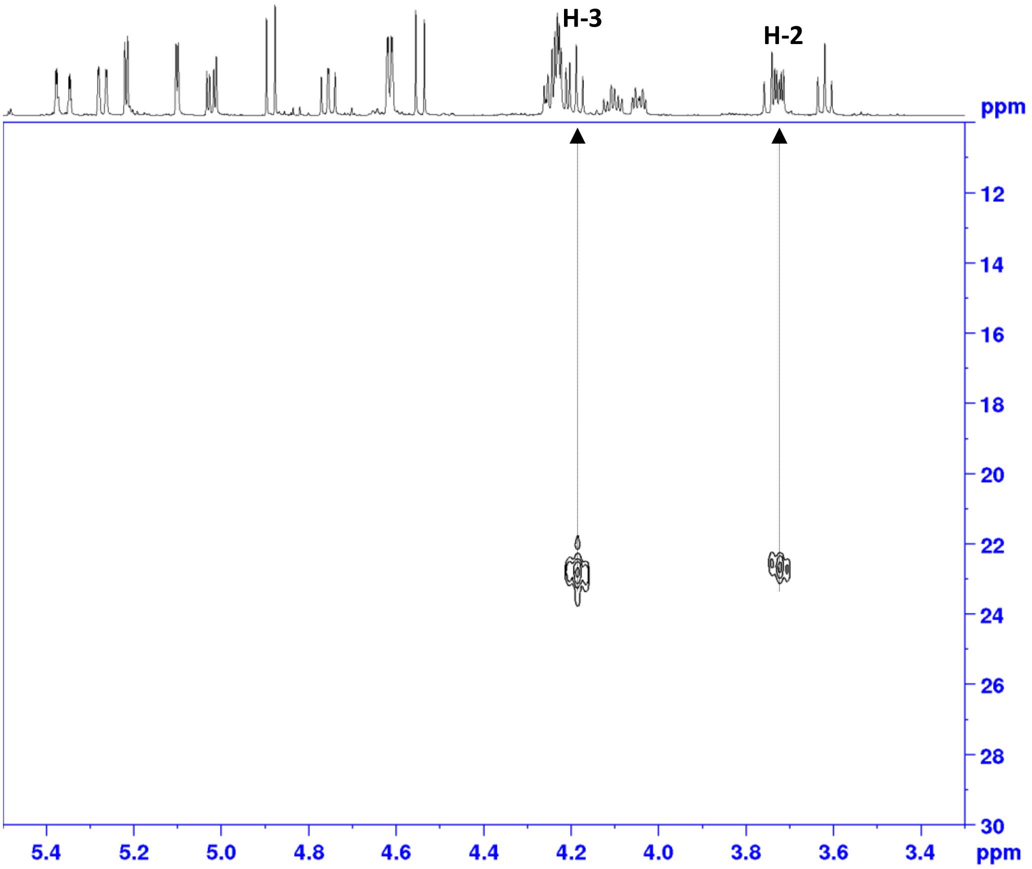

29 ^{1}H-NMR (600 MHz, $CDCl_3$)

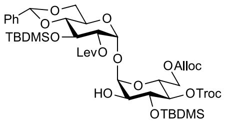

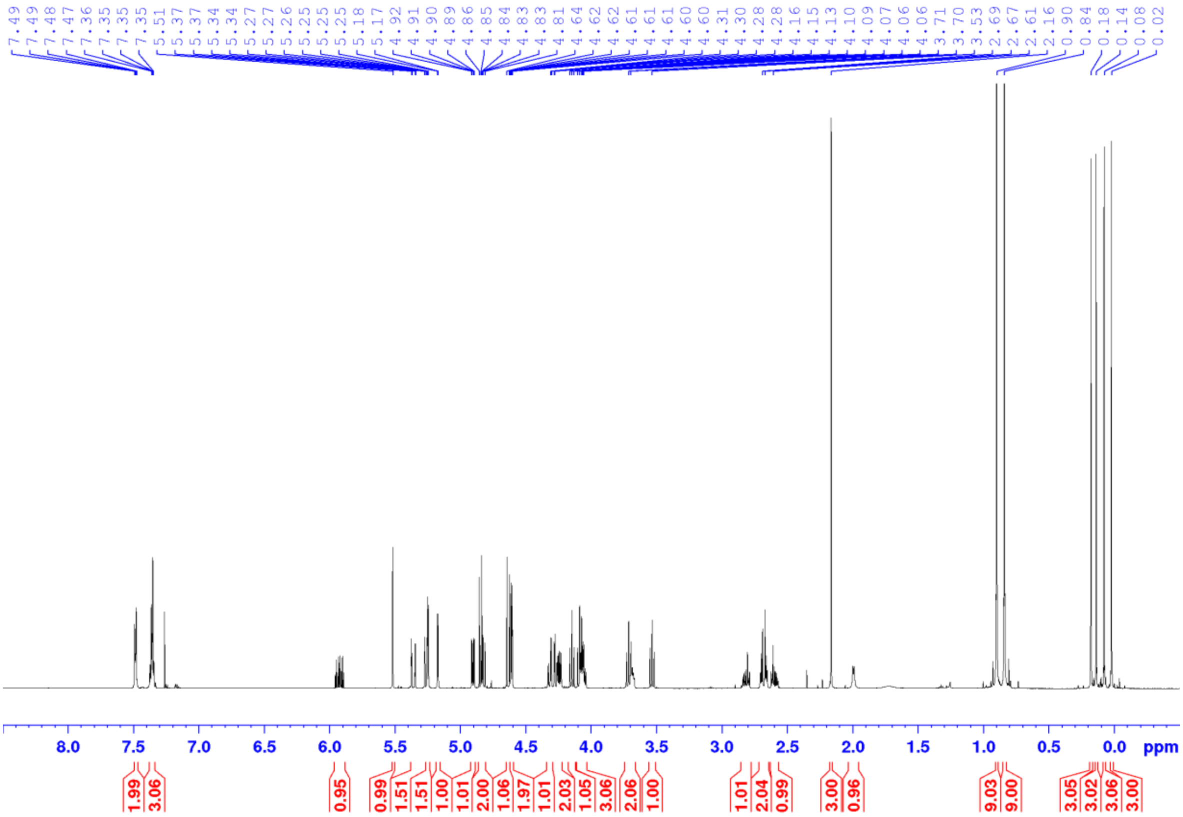

29 ^{13}C NMR (151 MHz, $CDCl_3$)

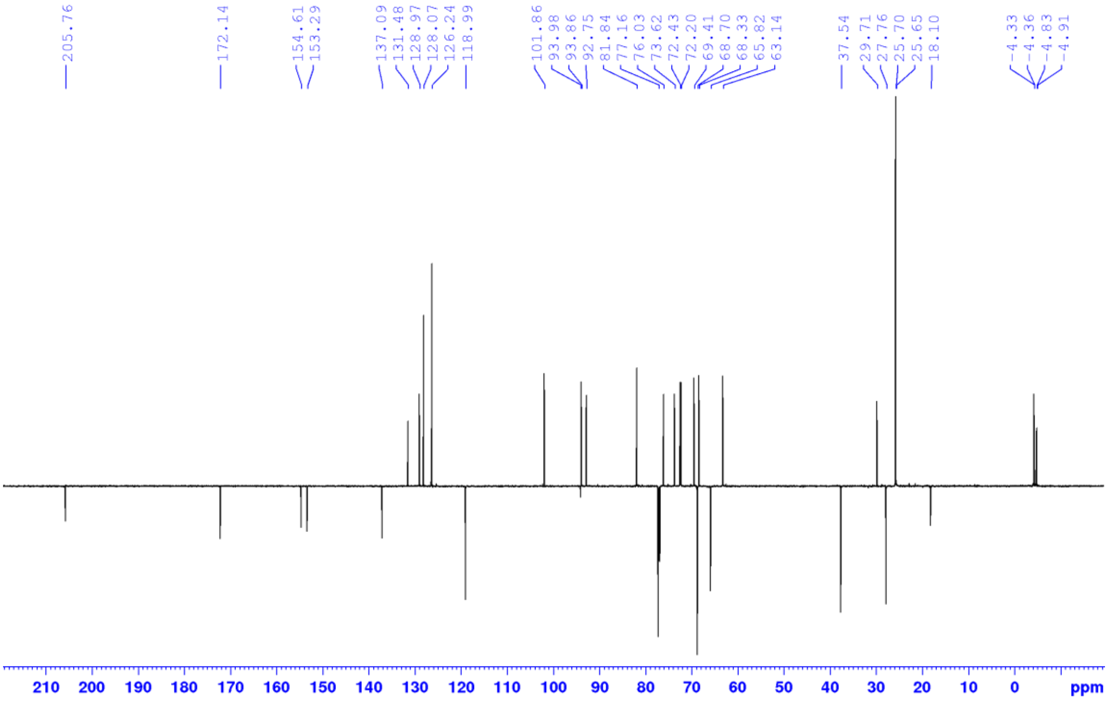

29 ^{1}H–^{29}Si HMBC (CDCl$_3$)

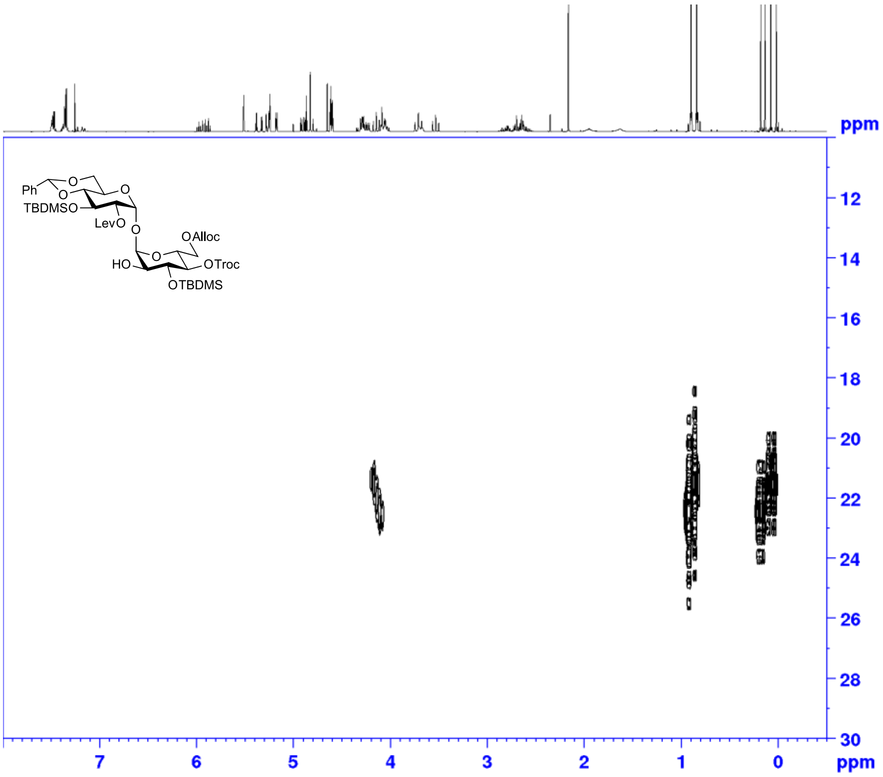

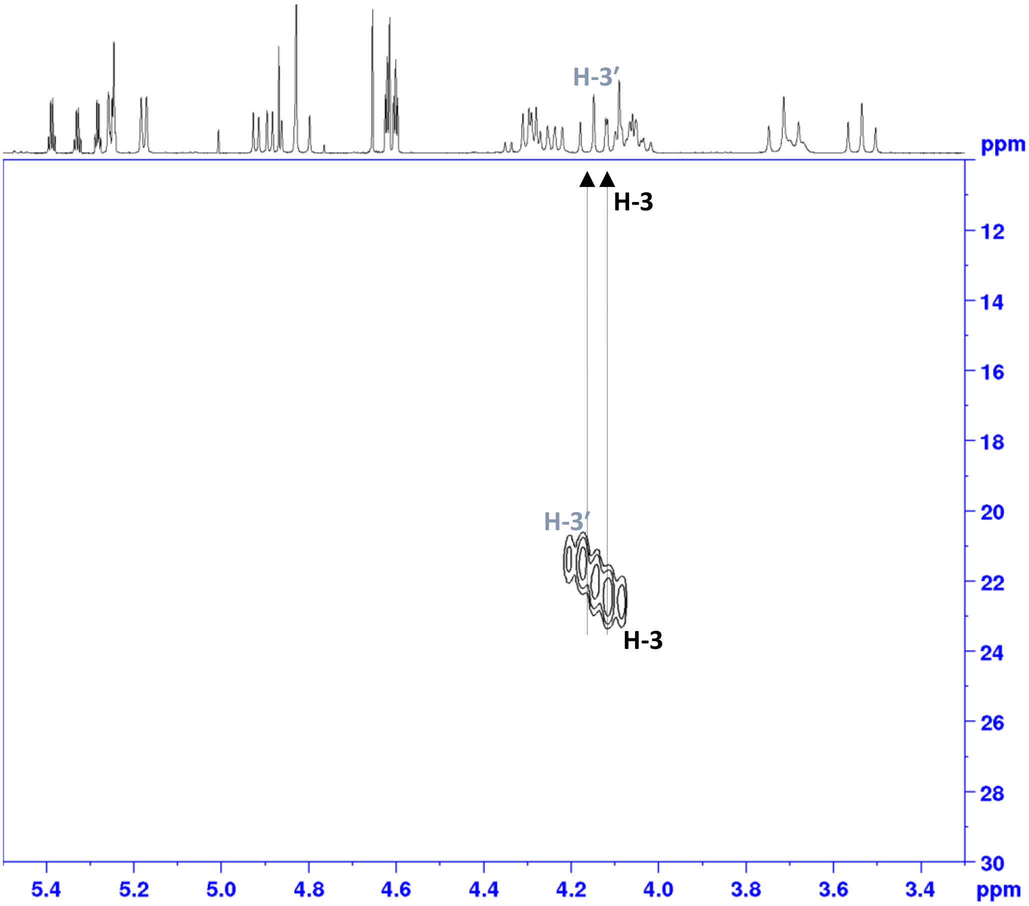

30 ^{1}H-NMR (600 MHz, $CDCl_3$)

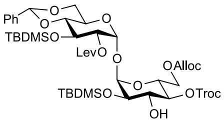

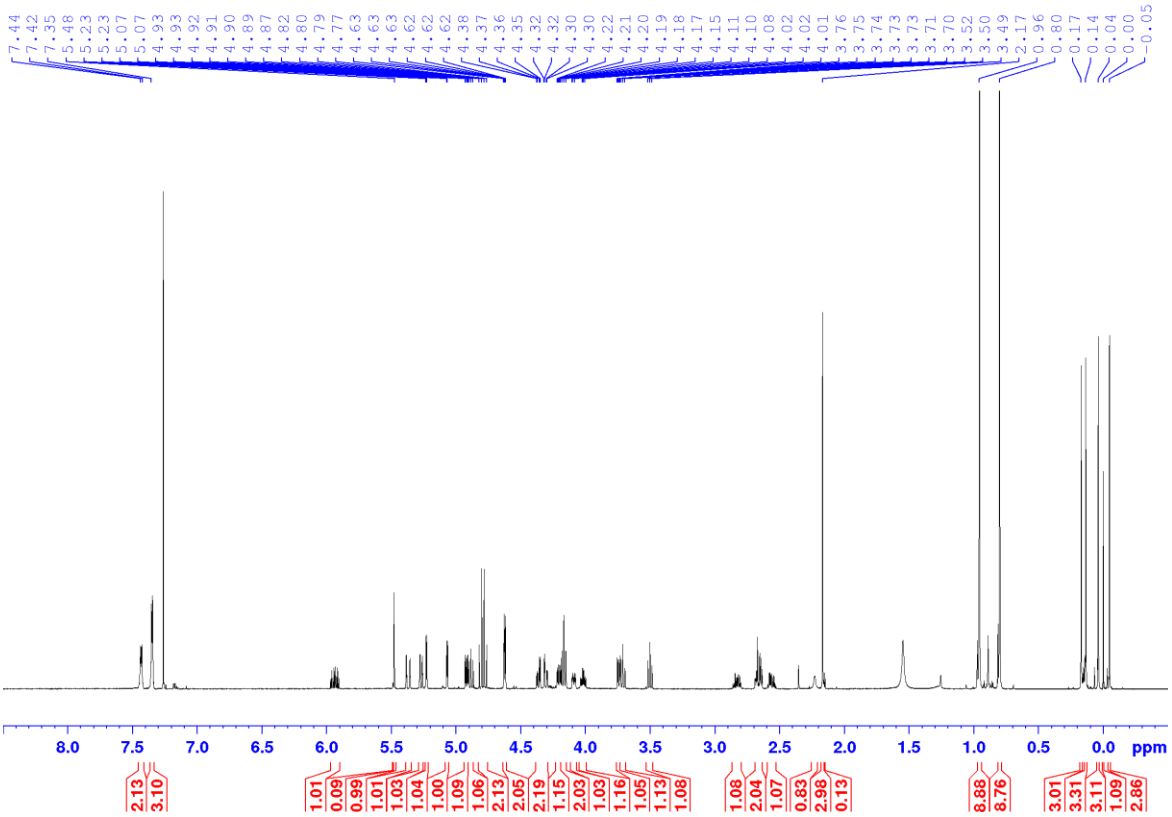

30 ^{13}C-NMR (151 MHz, $CDCl_3$)

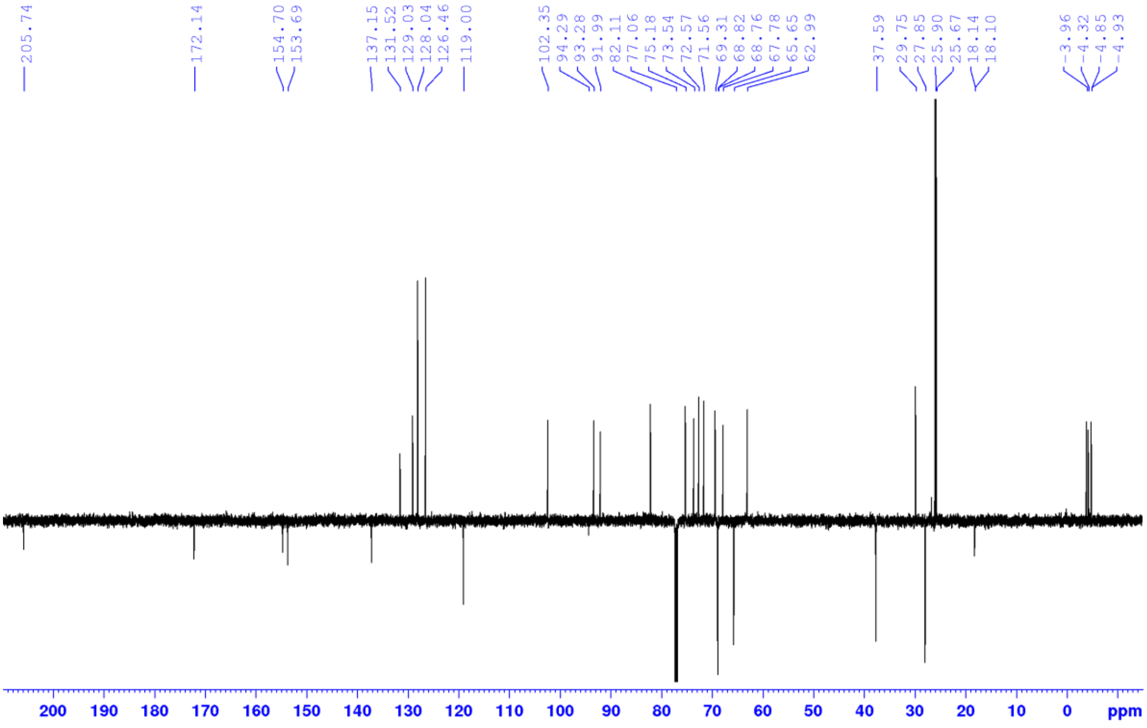

30 ^{1}H–^{29}Si HMBC ($CDCl_3$)

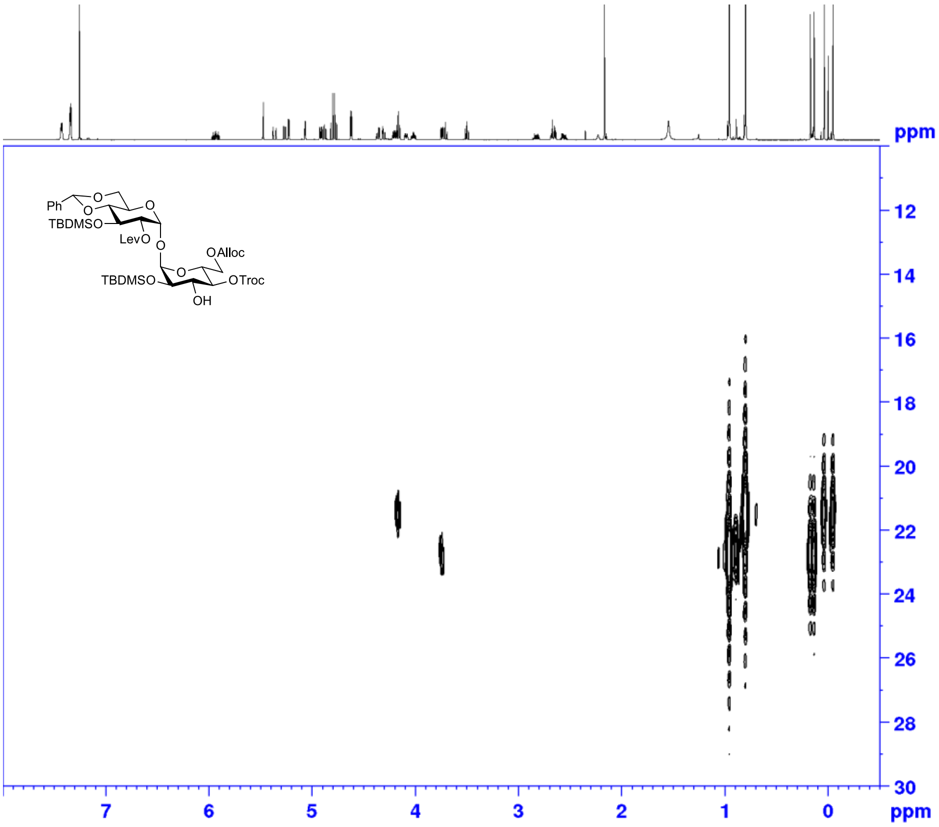

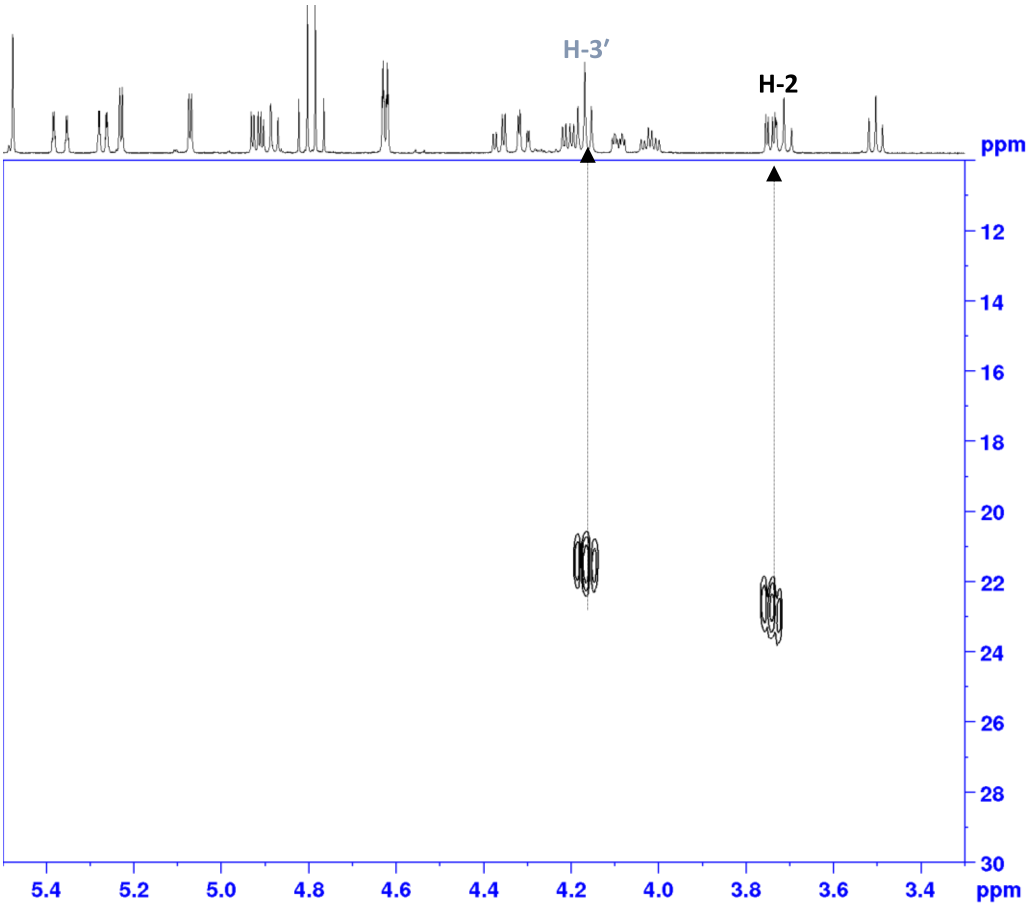

S2 ¹H-NMR (600 MHz, $CDCl_3$)

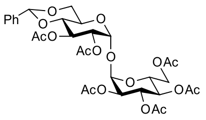

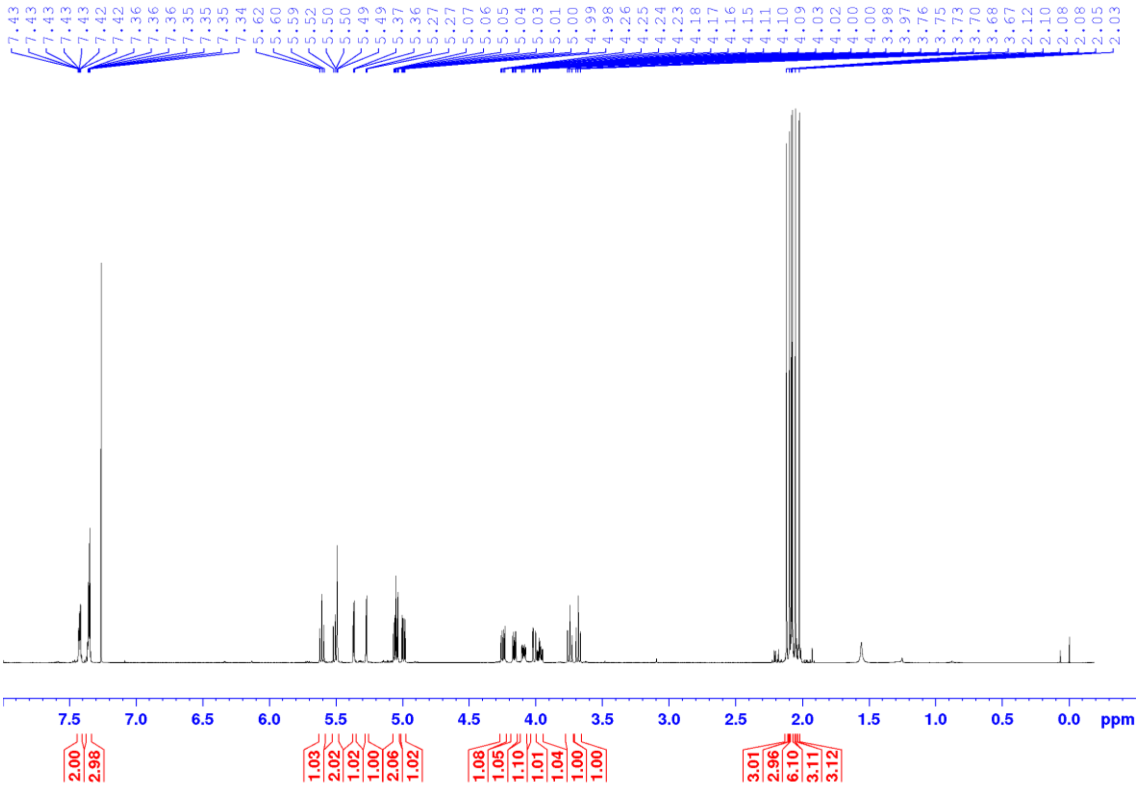

S2 ¹³C-NMR (151 MHz, $CDCl_3$)

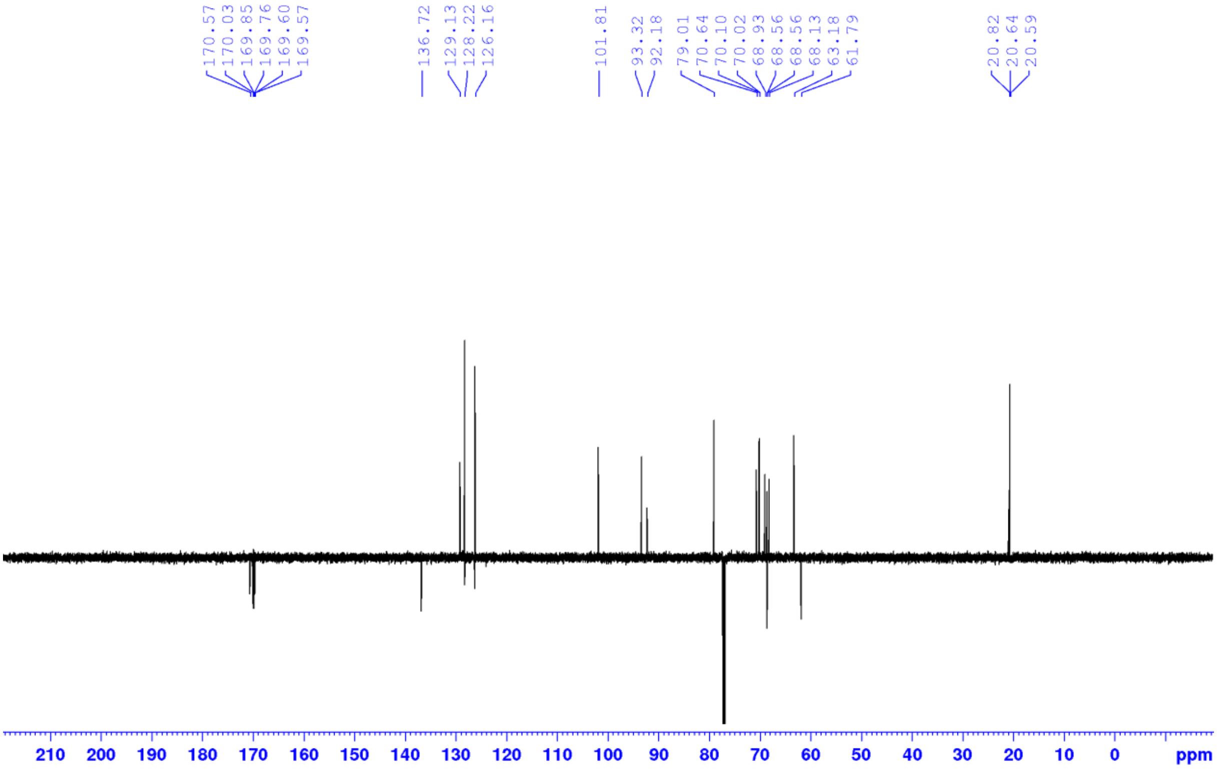

S4 ^{1}H-NMR (600 MHz, $CDCl_3$)

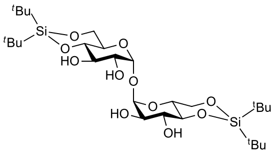

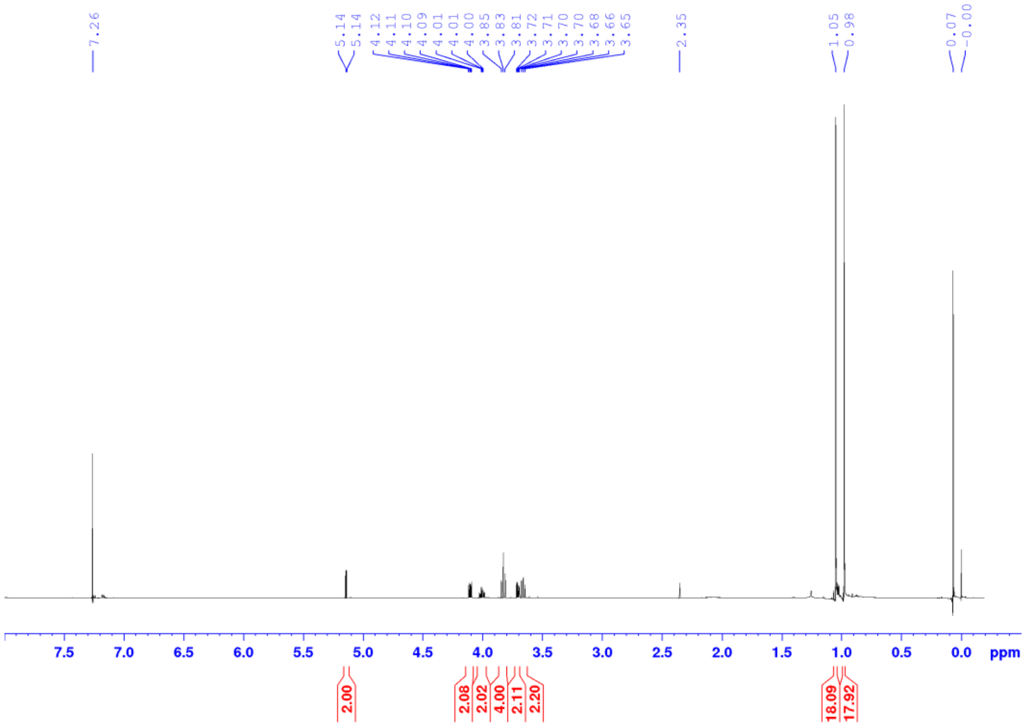

S4 ^{13}C-NMR (151 MHz, $CDCl_3$)

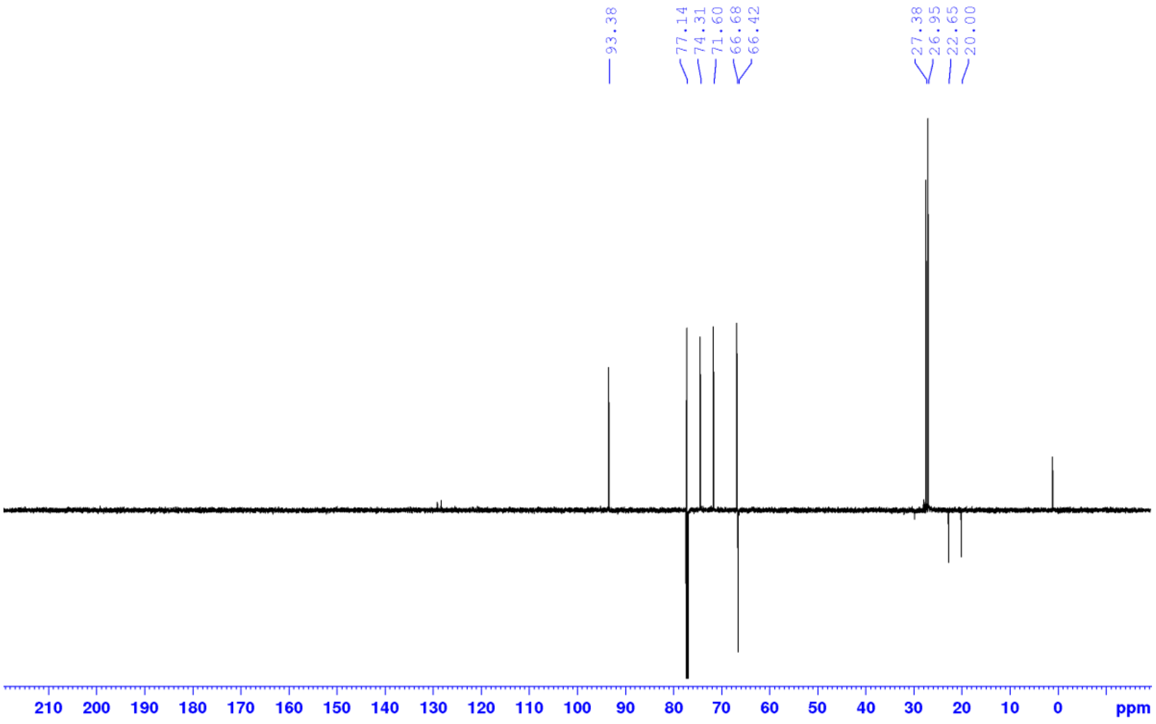

S5 ^{1}H-NMR (600 MHz, $CDCl_3$)

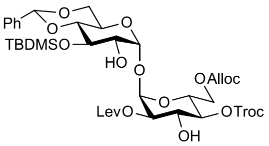

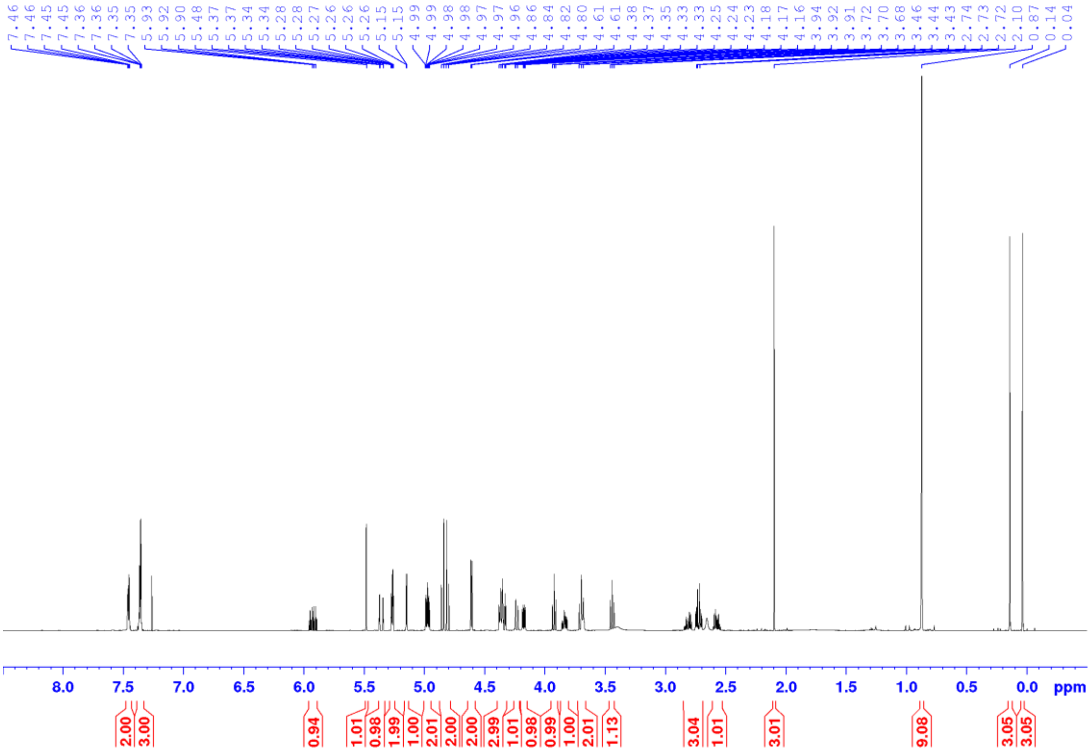

S5 ^{13}C-NMR (151 MHz, $CDCl_3$)

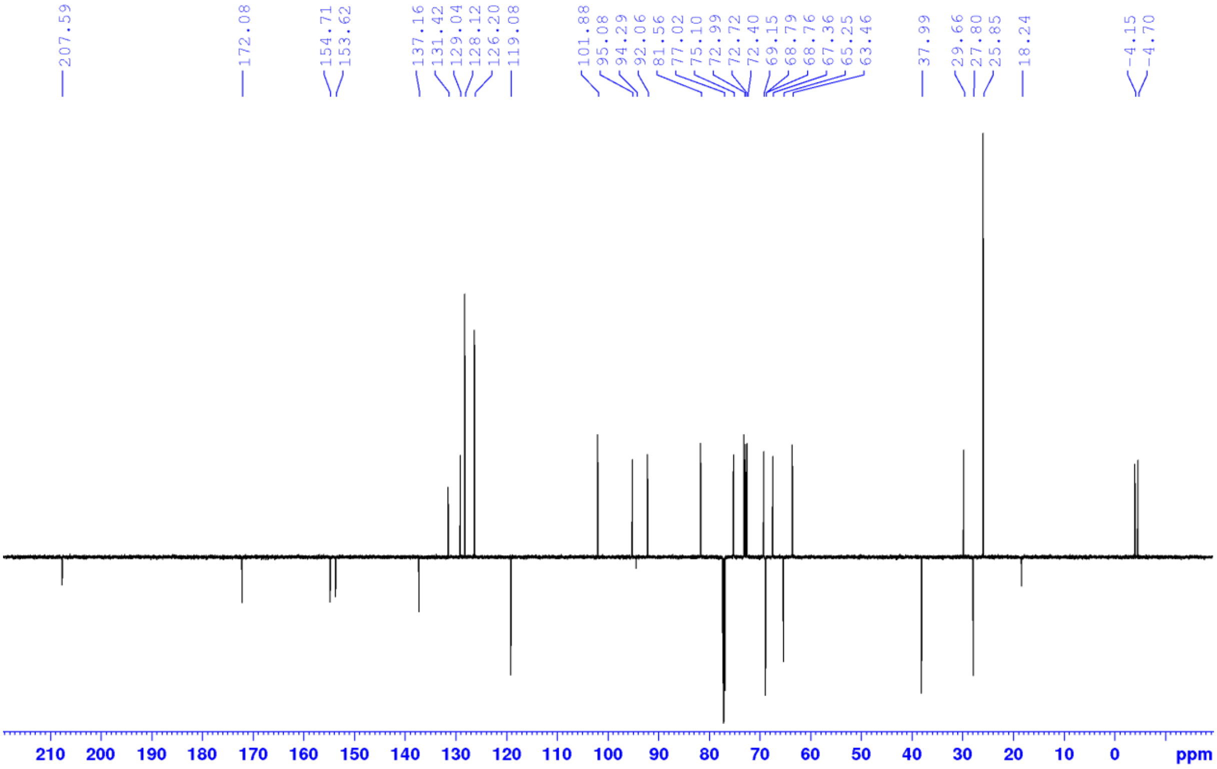

S5 ^1H–^{29}Si HMBC ($CDCl_3$)

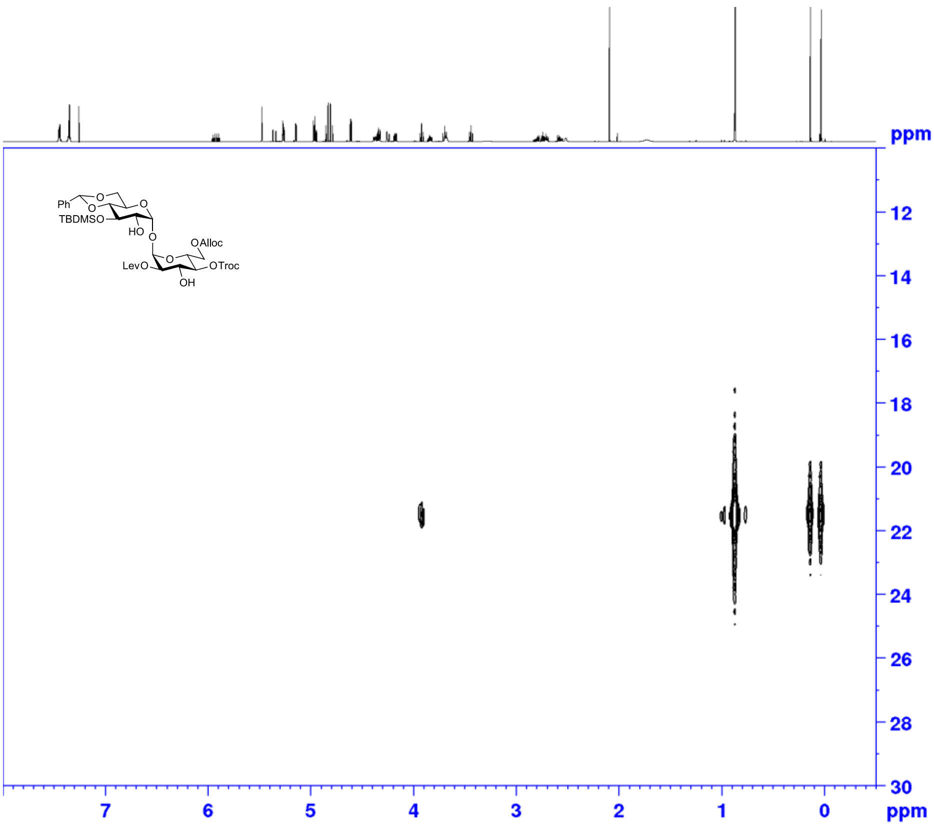

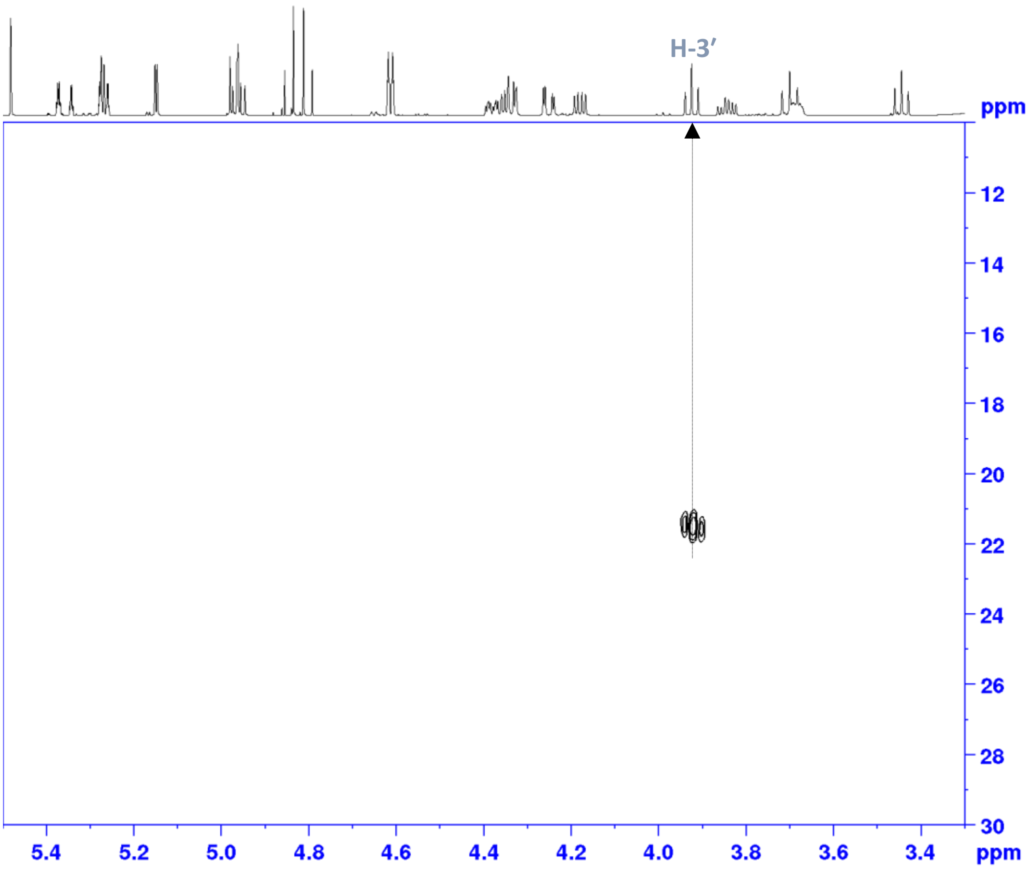

S6 ^{1}H-NMR (600 MHz, $CDCl_3$)

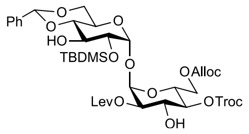

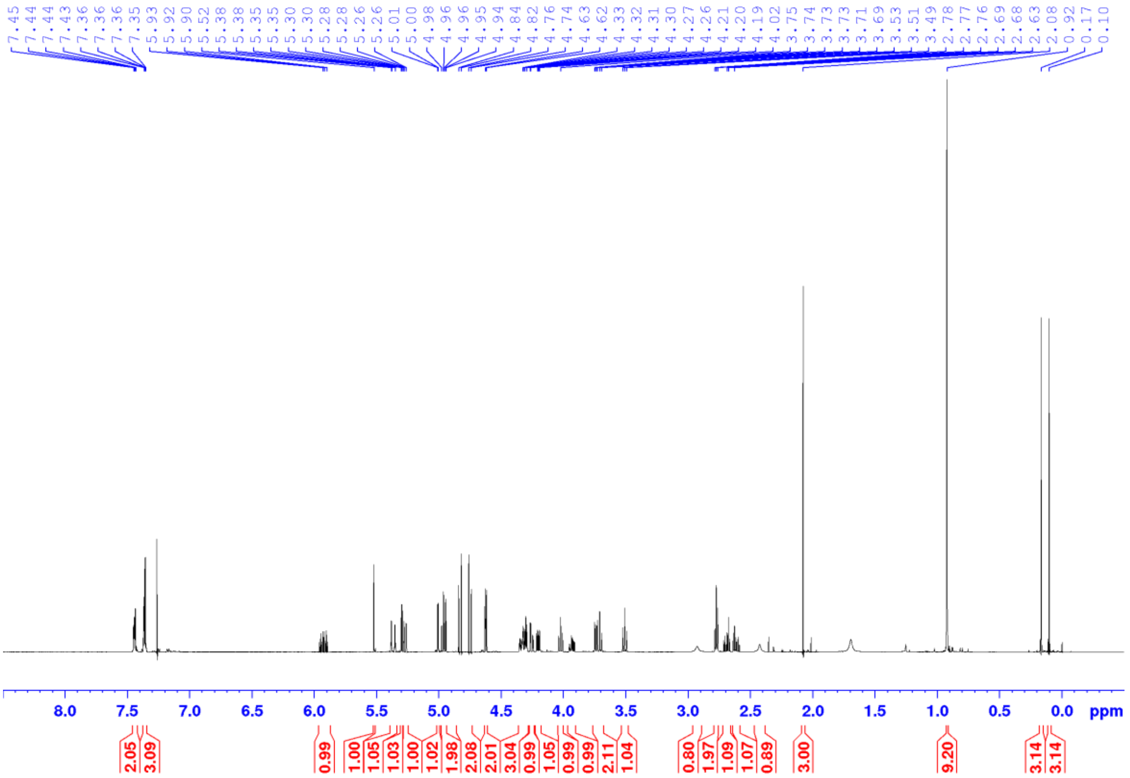

S6 ^{13}C NMR (151 MHz, $CDCl_3$)

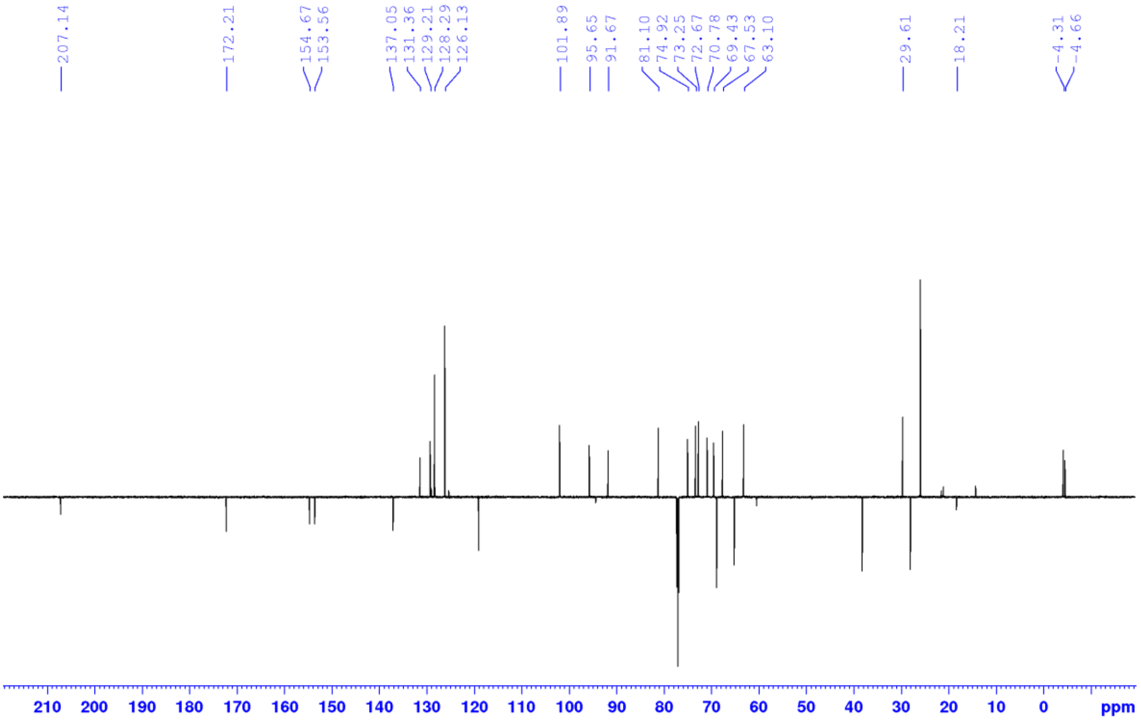

S6 ^{1}H–^{29}Si HMBC ($CDCl_3$)

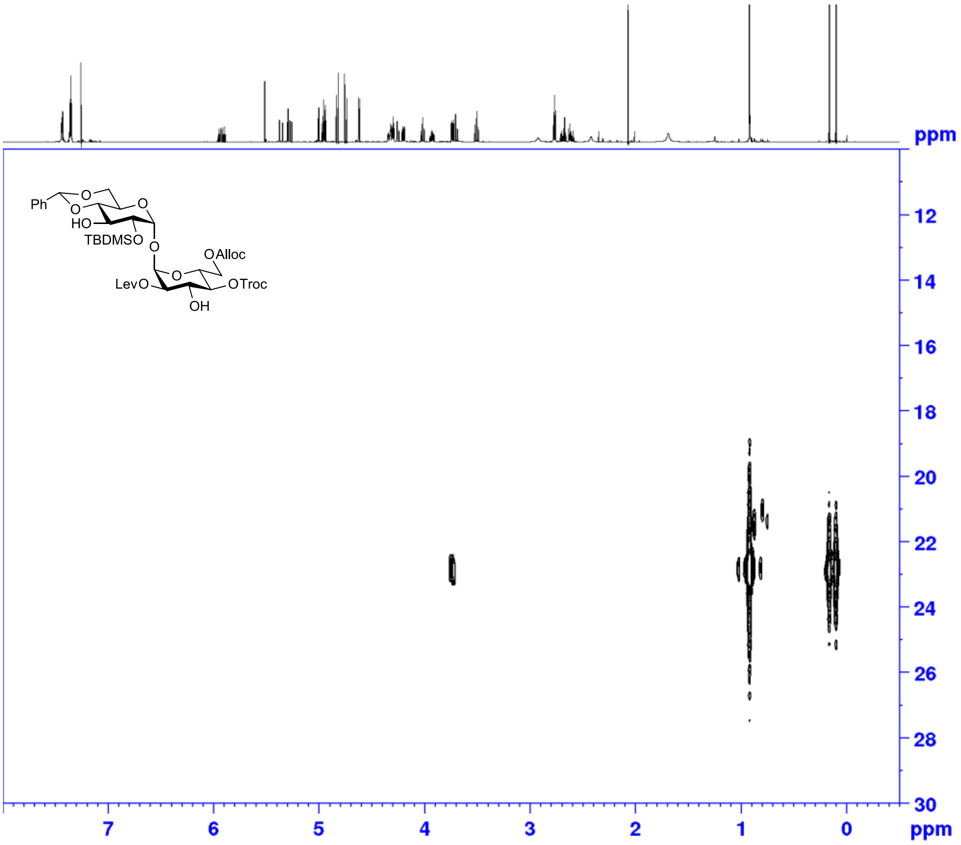

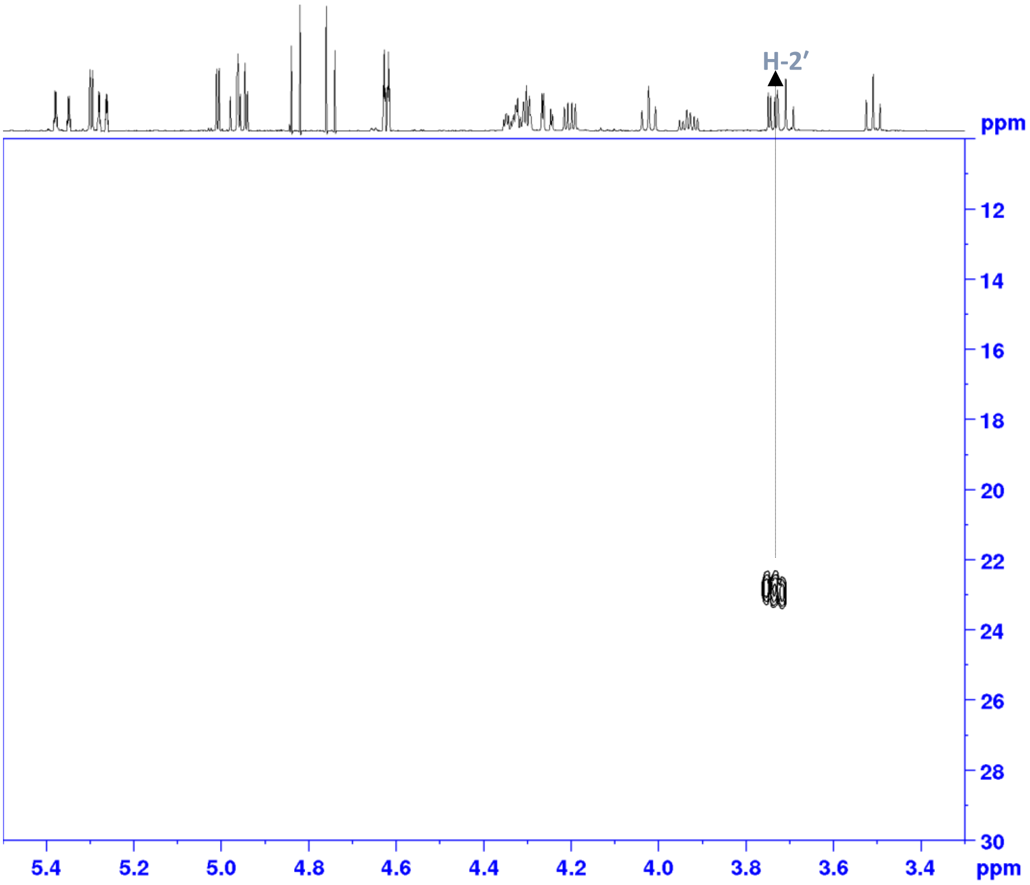

S7 ^{1}H-NMR (600 MHz, $CDCl_3$)

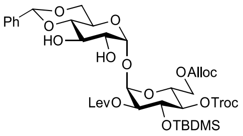

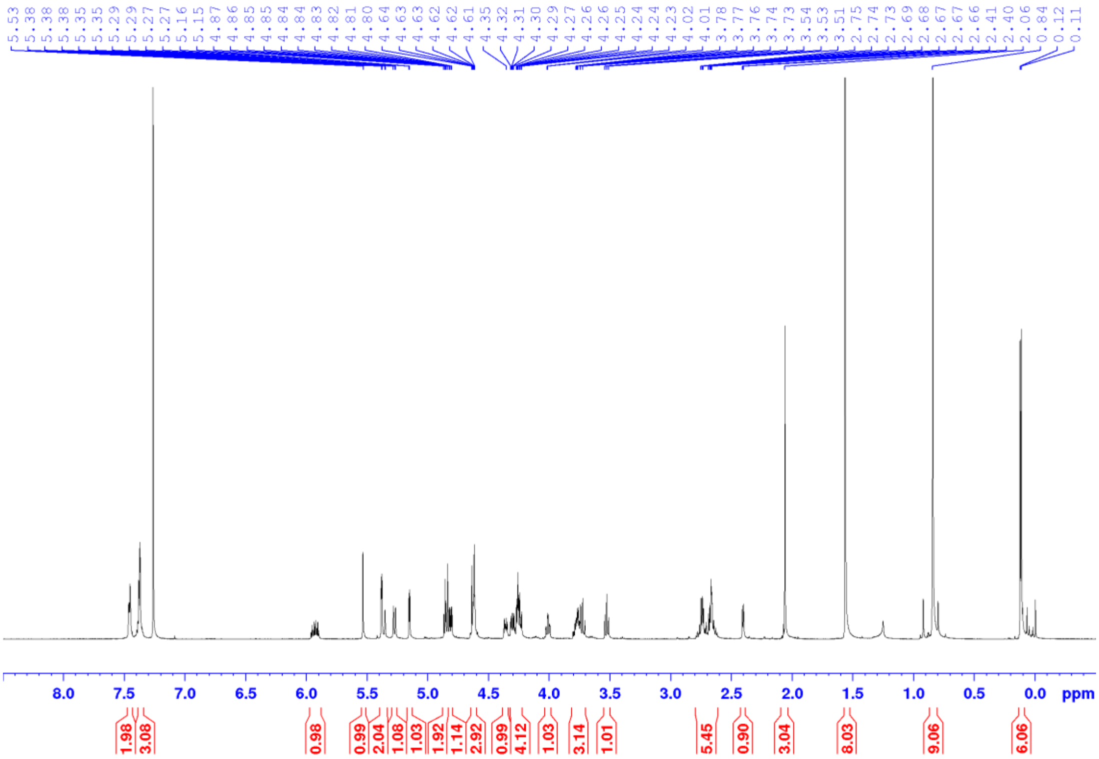

S7 ^{13}C NMR (151 MHz, $CDCl_3$)

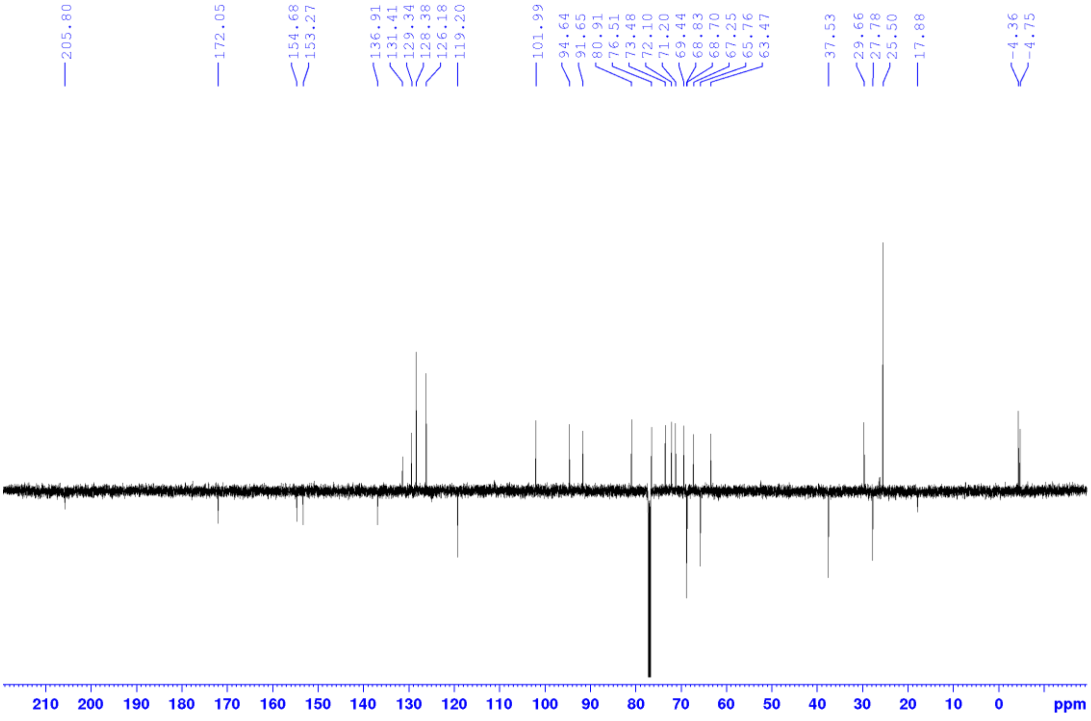

S7 ^{1}H–^{29}Si HMBC ($CDCl_3$)

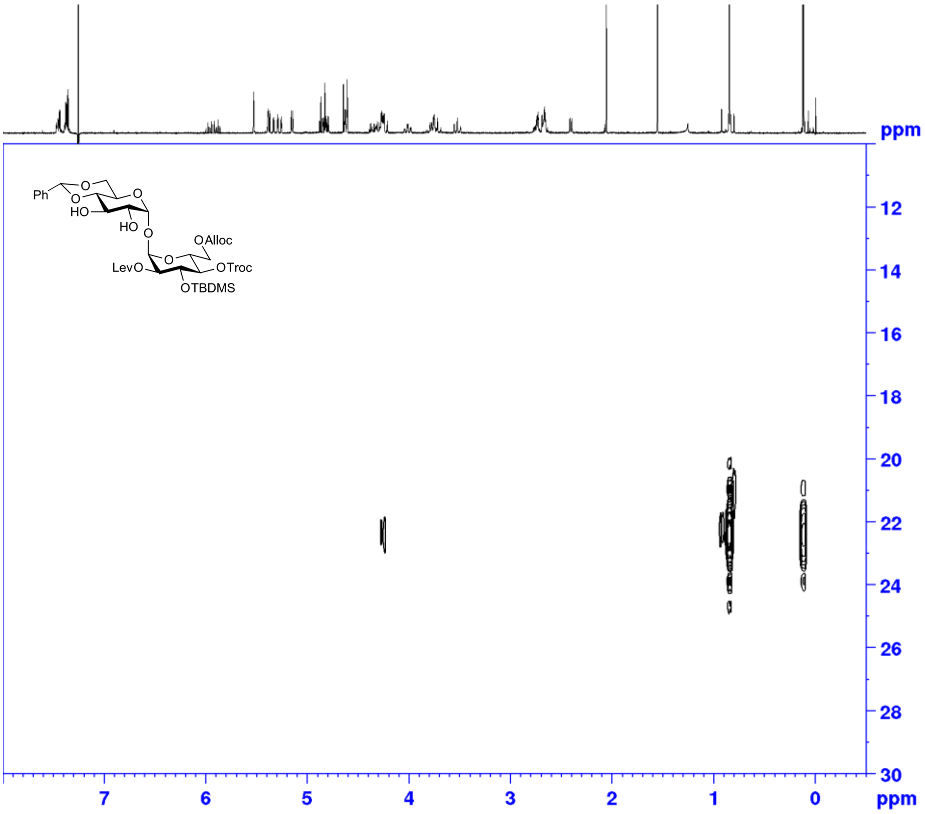

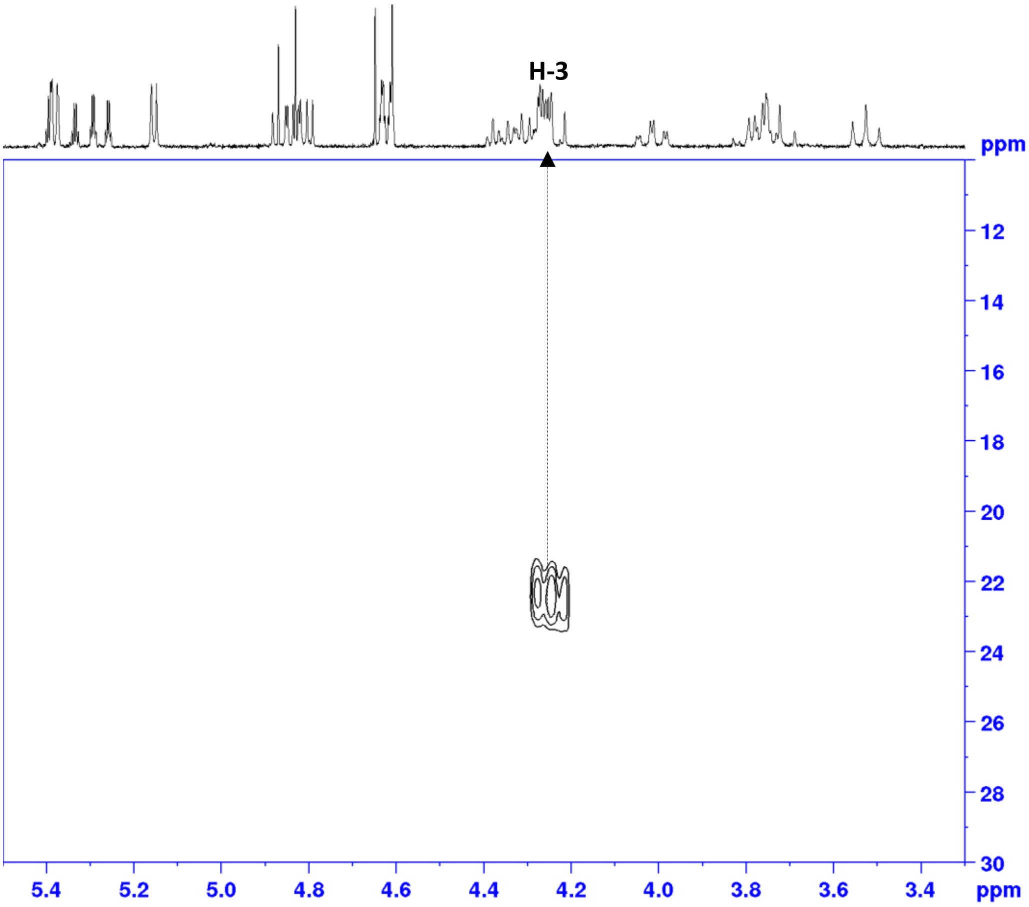

S8 ^{1}H NMR (600 MHz, $CDCl_3$)

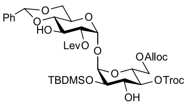

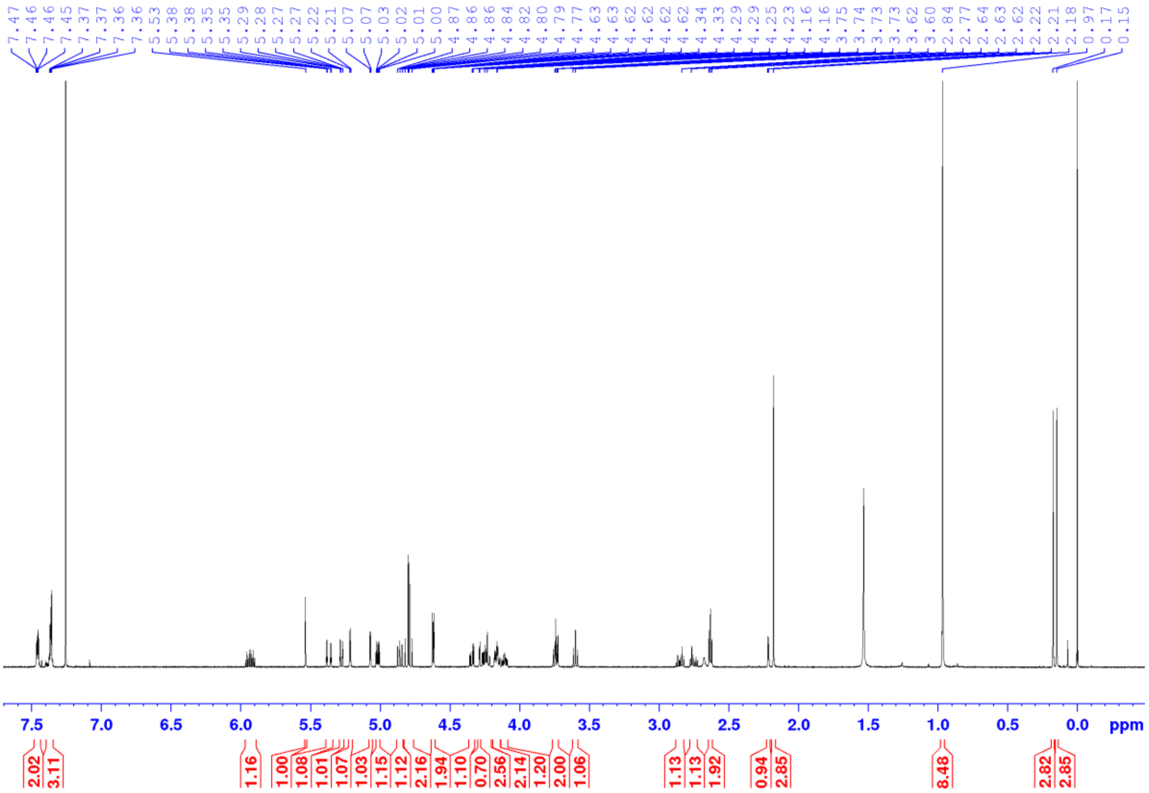

S8 ^{13}C NMR (151 MHz, $CDCl_3$)

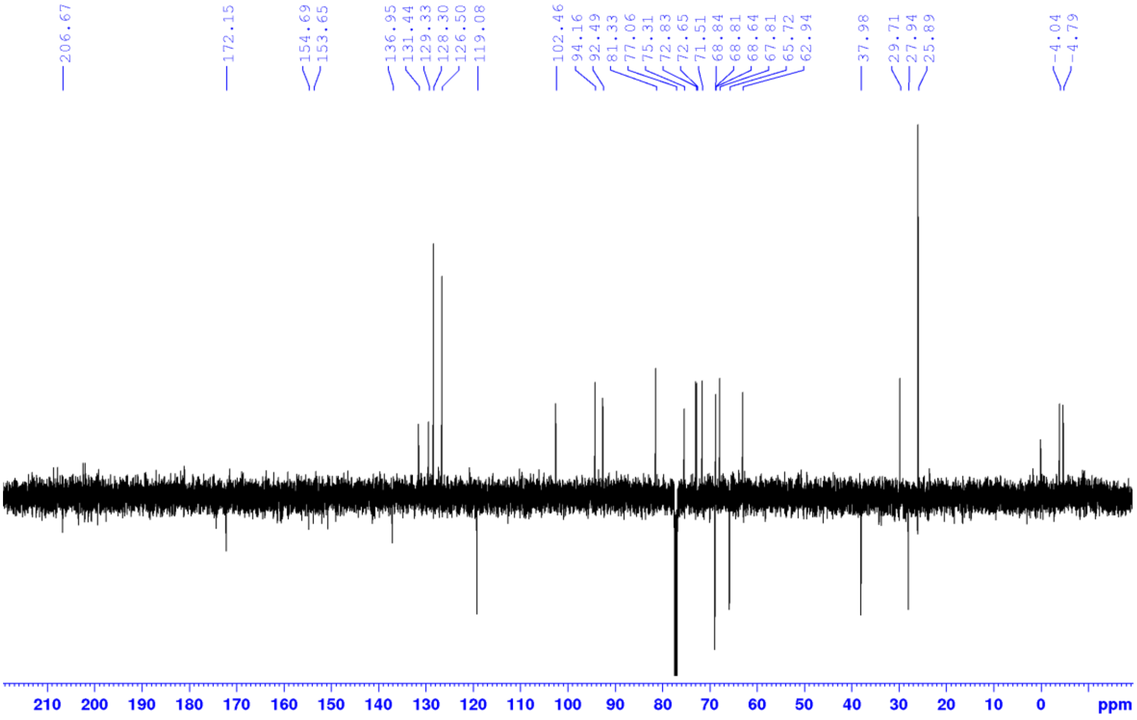

S8 $^{1}H–^{29}Si$ HMBC ($CDCl_3$)

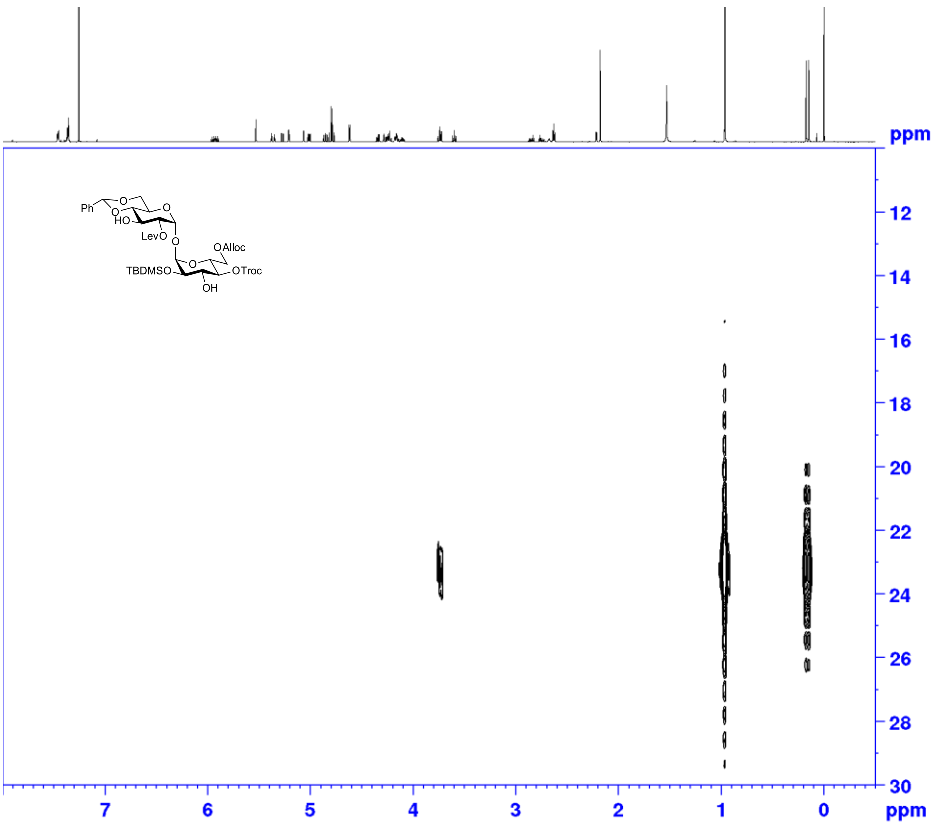

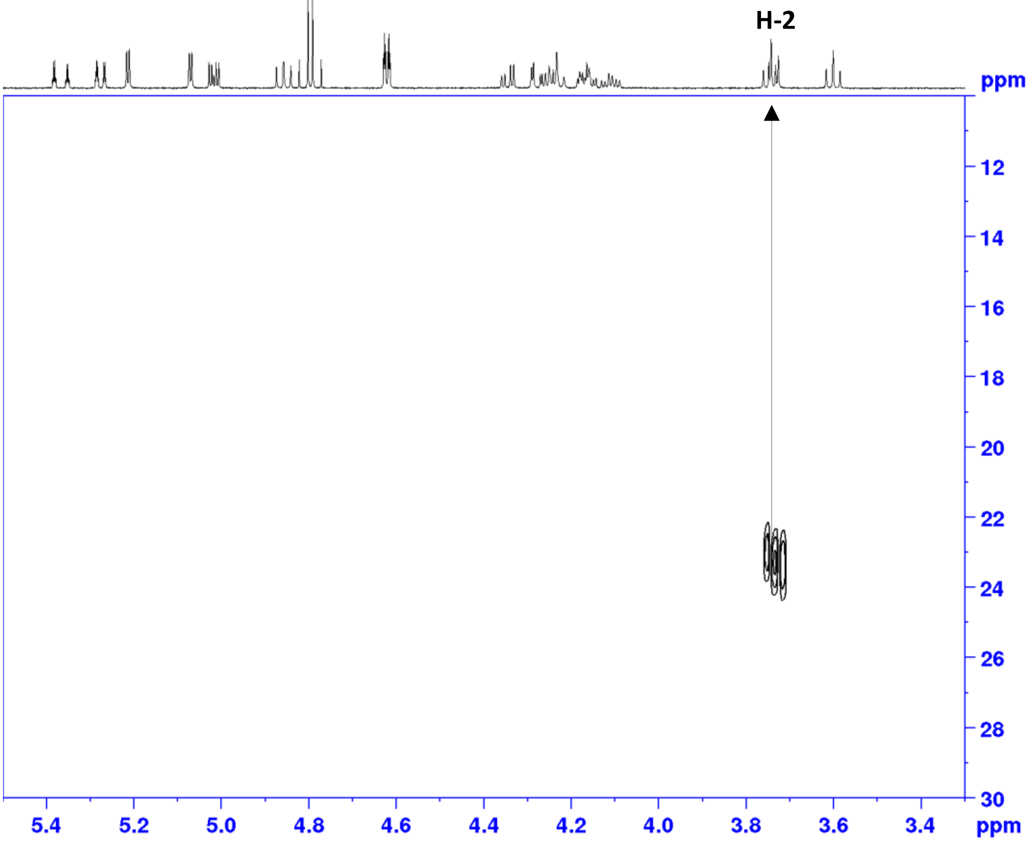

S9 ^{1}H NMR (600 MHz, $CDCl_3$)

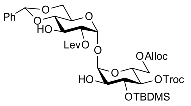

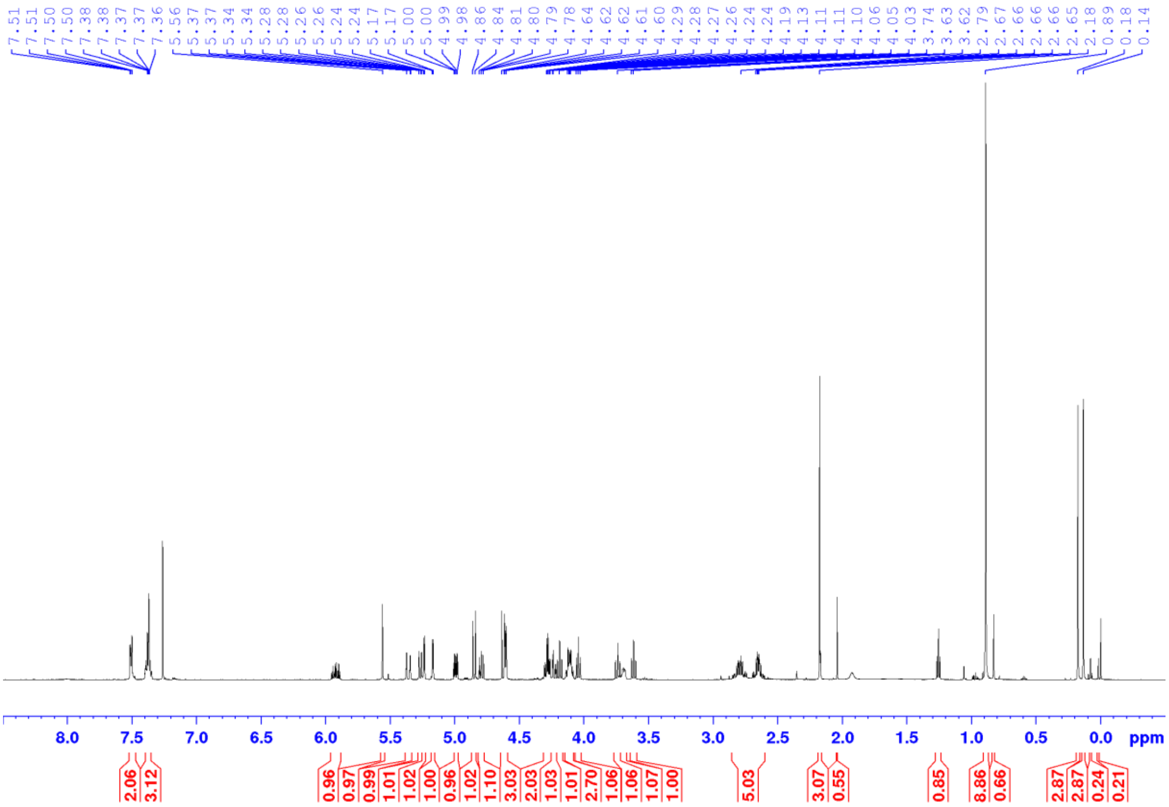

S9 ^{13}C NMR (151 MHz, $CDCl_3$)

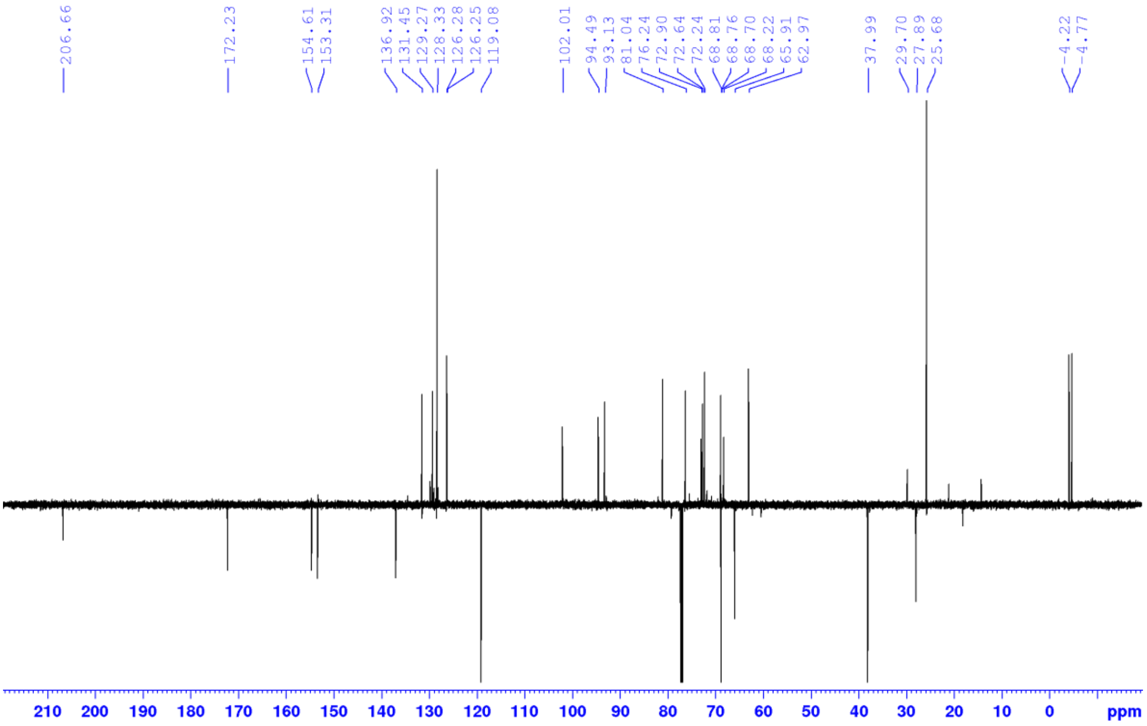

S9 ^{1}H–^{29}Si HMBC ($CDCl_3$)

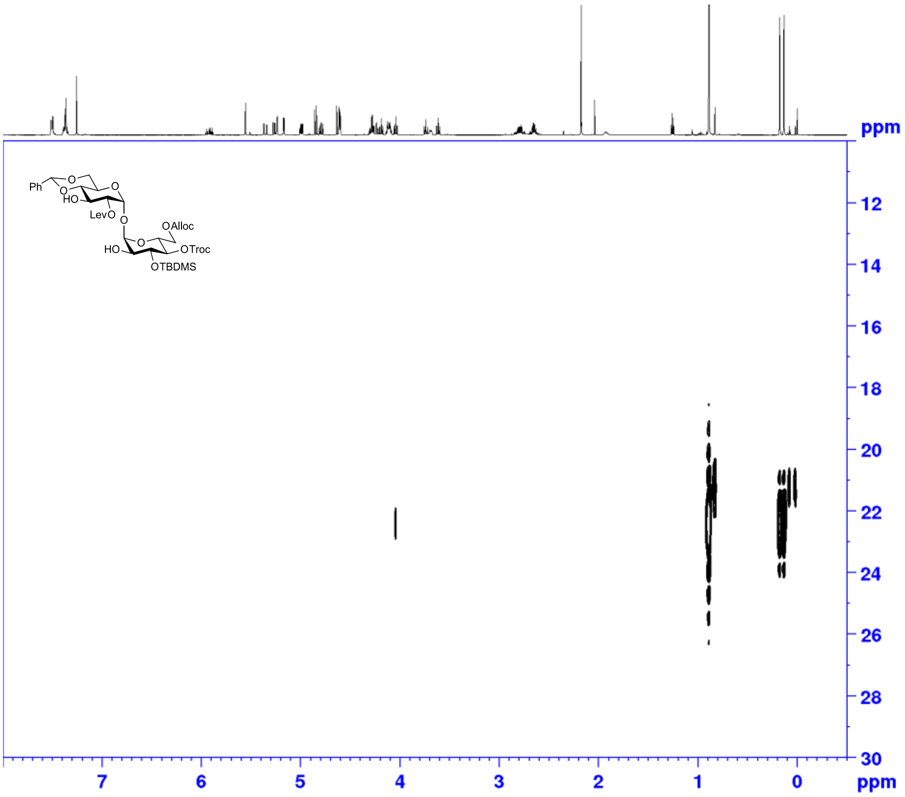

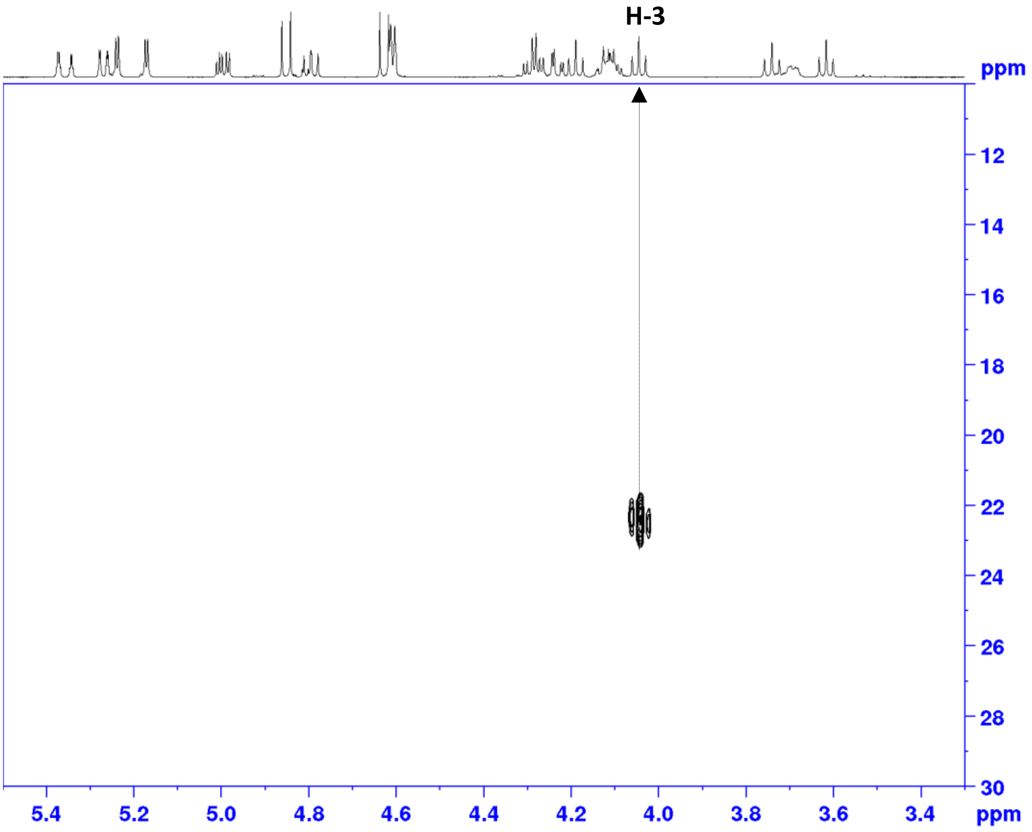

Supplement: Supplementary file 1 [file DataSheet1.pdf]
